# E3 ligase MG53 suppresses tumor growth by degrading cyclin D1

**DOI:** 10.1038/s41392-023-01458-9

**Published:** 2023-07-07

**Authors:** Meng Fang, Hong-Kun Wu, Yumeng Pei, Yan Zhang, Xiangyu Gao, Yanyun He, Gengjia Chen, Fengxiang Lv, Peng Jiang, Yumei Li, Wenwen Li, Peng Jiang, Lin Wang, Jiafu Ji, Xinli Hu, Rui-Ping Xiao

**Affiliations:** 1 State Key Laboratory of Membrane Biology, Institute of Molecular Medicine, College of Future Technology, Peking University, 100871 Beijing, China; 2 Peking-Tsinghua Center for Life Sciences, 100871 Beijing, China; 3 Department of Hepatobiliary and Pancreatic Surgery, the First Affiliated Hospital, Zhejiang University School of Medicine, 310003 Hangzhou, China; 4 Zhejiang Provincial Key Laboratory of Pancreatic Disease, 310003 Hangzhou, China; 5 Beijing City Key Laboratory of Cardiometabolic Molecular Medicine, Peking University, 100871 Beijing, China; 6 Key Laboratory of Carcinogenesis and Translational Research (Ministry of Education/Beijing), Gastrointestinal Tumor Center, Peking University Cancer Hospital & Institute, 100142 Beijing, China; 7 School of Life Sciences, Tsinghua University, 100084 Beijing, China; 8 Research Center for Tissue Engineering and Regenerative Medicine, Union Hospital, Tongji Medical College, Huazhong University of Science and Technology, 430022 Wuhan, China

**Keywords:** Cancer, Cell biology

## Abstract

Due to the essential role of cyclin D1 in regulating transition from G1 to S phase in cell cycle, aberrant cyclin D1 expression is a major oncogenic event in many types of cancers. In particular, the dysregulation of ubiquitination-dependent degradation of cyclin D1 contributes to not only the pathogenesis of malignancies but also the refractory to cancer treatment regiments with CDK4/6 inhibitors. Here we show that in colorectal and gastric cancer patients, MG53 is downregulated in more than 80% of tumors compared to the normal gastrointestinal tissues from the same patient, and the reduced MG53 expression is correlated with increased cyclin D1 abundance and inferior survival. Mechanistically, MG53 catalyzes the K48-linked ubiquitination and subsequent degradation of cyclin D1. Thus, increased expression of MG53 leads to cell cycle arrest at G1, and thereby markedly suppresses cancer cell proliferation in vitro as well as tumor growth in mice with xenograft tumors or AOM/DSS induced-colorectal cancer. Consistently, MG53 deficiency results in accumulation of cyclin D1 protein and accelerates cancer cell growth both in culture and in animal models. These findings define MG53 as a tumor suppressor via facilitating cyclin D1 degradation, highlighting the therapeutic potential of targeting MG53 in treating cancers with dysregulated cyclin D1 turnover.

## Introduction

Cyclin D1 plays multiple roles in tumorigenesis, including enabling cell cycle progression, promoting cell migration, facilitating DNA damage repair, and driving chromosome instability.^1^ The oncogenic effect of cyclin D1 can be attributed to mutations in its coding region, but is more often associated with cyclin D1 overexpression.^2,3^ The elevation in cyclin D1 level can be caused by genetic rearrangements,^4,5^ gene duplication,^6,7^ transcriptional upregulation,^8–10^ or aberrant degradation.^11,12^ In particular, the degradation of cyclin D1 is dictated by its polyubiquitination in normal cell cycle when the protein level of cyclin D1 fluctuates at different stages. The expression of cyclin D1 is induced by mitogenic stimulations. Then cyclin D1 enters nucleus and couples with CDK4/6 to inactivate retinoblastoma tumor suppressor (Rb) by phosphorylation, which enables the transcription of E2F-dependent genes. When cells enter S phase, cyclin D1 is phosphorylated and transported back to cytosol where it is ubiquitinated followed by degradation via proteasome. In addition, various stress conditions, such as radiation, DNA damage, drug treatment, or starvation, may also induce cyclin D1 degradation and subsequent cell cycle exit.^13^

Emerging evidence has pinpointed a pivotal role of dysfunctional E3 ubiquitin ligase in the cancer-associated cyclin D1 accumulation. Moreover, recent studies have implicated dysfunction in ubiquitination-dependent degradation of cyclin D1 as a key mechanism underlying insensitivity towards CDK4/6 inhibitors (CDK4/6is) in treating certain types of cancers.^14–16^ Thus, innate insensitivity or acquired resistance to CDK4/6is constitutes a major hindrance in the clinical application of these drugs. To date, two large E3 ligase complexes, S-phase kinase-associated protein 1-Cullin 1-F-box complex (SCF) and anaphase-promoting complex/cyclosome (APC/C), have been reported to mediate the degradation-related polyubiquitination of cyclin D1.^1^ SCF E3 ubiquitin ligase complexes contain different substrate-interacting proteins. Among them, SCF^FBX4/αB^ crystallin is a well-validated E3 ligase complex of cyclin D1. The mRNA levels of αB crystalline and FBX4 are downregulated in prostate, thyroid, and breast adenocarcinomas, as well as lymphomas.^11^ In addition, mutations of FBX4 were found in esophageal tumors.^17^ SCF^FBXW8^ is identified to facilitate cyclin D1 ubiquitination in HCT116 and SW480 colon cancer cells and T98G glioblastoma cells, although there is no direct evidence linking SCF^FBXW8^ to tumorigenesis in vivo.^18^ β-transducin repeat-containing protein (β-TrCP1) is another adaptor that interacts with cyclin D1 in response to the treatment of the peroxisome proliferator-activated receptor γ agonist STG28.^19^ Intriguingly, β-TrCP1 induces cyclin D1 degradation when prostate cancer cell LNCaP are challenged with glucose starvation, while other E3 ligases are not seem to be involved under this condition. Another E3 ubiquitin ligase complex APC/C mediates irradiation-induced degradation of cyclin D1 via its subunit Cdc27/APC3.^20,21^ It is noteworthy that the expression of Cdc27/APC3 is repressed in many types of cancer cells, but whether Cdc27/APC3 dysregulation promotes tumorigenesis via cyclin D1 is not reported. It is not uncommon that a protein has several E3 ligases, but the functions of the E3 ligases of cyclin D1 might be cancer-type and context dependent. Furthermore, it has been shown that the ablation or knockdown of the components of either SCF or APC/C E3 ligase complex does not reduce cyclin D1 abundance in vivo or in vitro.^22^ Therefore, it is important to identify other mechanisms contribute to the dysregulation of cyclin D1 protein turnover in the context of different cancer types.

MG53 (also named TRIM72) is an E3 ligase of insulin receptor and insulin receptor substrate 1. Thus, increases in MG53 expression contribute to metabolic disorders by impairing insulin signaling and glucose metabolism.^23,24^ On the other hand, MG53 facilitates membrane repair^25,26^ and activates survival signaling pathways,^25,27,28^ and thereby playing a protective role against acute damage of multiple organs.^29–31^ An unbiased screening has identified MG53 as one of the 4 essential regulators that limits the proliferation and metastasis of non-small cell lung cancer,^32^ however how MG53 functions as tumor suppressor is unclear. To delineate the underlying mechanism, we examined the function of MG53 in different cancer cell lines and searched for the cell cycle regulators that interacted with MG53 by proteomic analysis. We further validated our findings using MG53 transgenic and knockout mice, as well as samples from patients with colorectal and gastric cancers.

Specifically, we have demonstrated that the expression of MG53 is overtly decreased in more than 80% of the tumors examined relative to the normal tissues from the same patient. The downregulation of MG53 is strongly correlated with reduced overall survival of patients with colorectal or gastric cancers. Mechanistically, MG53 functions as an E3 ligase of cyclin D1, thereby suppressing tumor growth in mouse models with xenograft tumors as well as carcinogen-induced colorectal cancer. In contrast, depletion of MG53 has the opposite effects of accelerating cancer cell proliferation and exacerbating tumorigenesis in mice. Furthermore, adenoviral delivery of MG53 expression vector into xenograft tumors significantly retards tumor growth. These results reveal the mechanism of action of MG53 in suppressing tumor growth and demonstrate the potential of targeting MG53 in cancer therapy.

## Results

### Reduced MG53 abundance is associated with inferior survival in gastrointestinal cancer patients

**Fig. 1:**
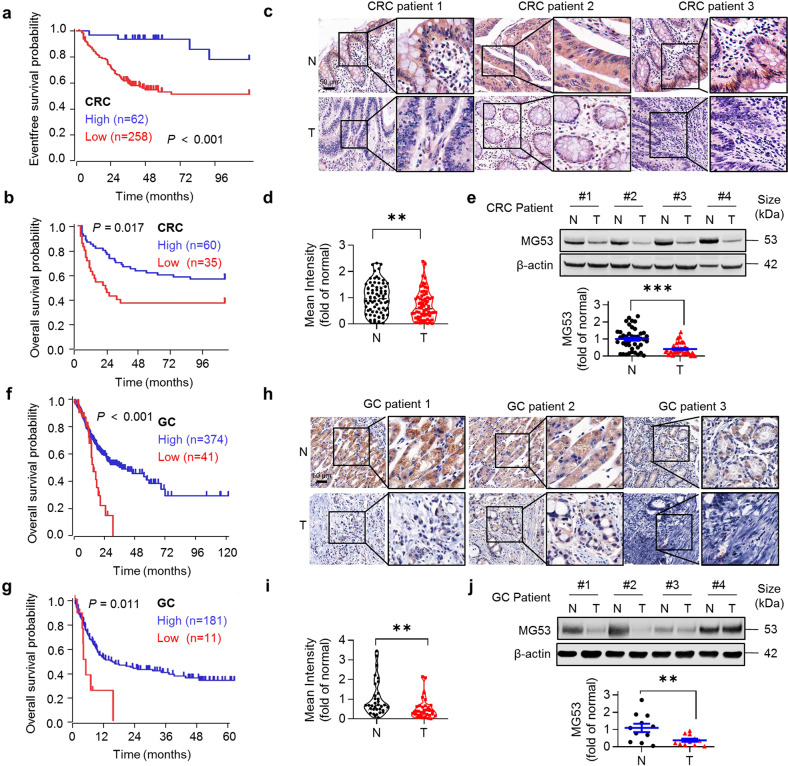
Reduced MG53 expression is associated with poorer clinical outcomes in gastrointestinal cancer patients. **a**, **b** Kaplan–Meier survival analysis of colorectal cancer patients from GEO databases (**a** GEO ID: GSE24551; **b** GEO ID: GSE30378). **c**, **d** Representative images (**c**) and averaged signal intensity (**d**) of immunohistochemical staining of MG53 (brown) in human colorectal tumor tissue microarrays; *n* = 71. **e** Representative western blots and averaged data showing the expression of MG53 in tumor tissues (T) compared to the corresponding adjacent normal tissues (N) from colorectal cancer patients; *n* = 46. **f**, **g** Kaplan–Meier survival analysis of gastric cancer patients from TCGA database (**f**, TCGA ID: STAD) and GEO database (**g**, GEO ID: GSE15459). **h**, **i** Representative images (**h**) and averaged signal intensity (**i**) of immunohistochemical staining of MG53 (brown) in human gastric tumor tissue microarrays; *n* = 33. **j** Representative western blots and averaged data showing the expression of MG53 in tumor tissues (T) compared to the corresponding adjacent normal tissues (N) from gastric cancer patients; *n* = 11. Data were presented as mean ± s.e.m. and analyzed using two-tailed paired *t* test. **P < 0.01, and ***P < 0.001 as compared with corresponding normal tissues. **a**, **b**, **f**, **g** Data was analyzed in *R2: Genomics Analysis and Visualization Platform* (http://r2platform.com) and auto select best cutoff was chosen in the analysis

To get mechanistic insights in the function of MG53 in cancer, we performed Kaplan–Meier survival analysis of two cohorts of colorectal cancer. Results showed that patients defined as having “high expression” of MG53 had significantly prolonged survival (Fig. 1a, b). Remarkably, immunohistochemical staining showed decreased signal intensity of MG53 in tumors as compared with the adjacent normal colorectal tissues (Fig. 1c, d). Consistently, we found decreased MG53 protein abundance in more than 80% of the tumor samples compared with the adjacent normal tissues (Fig. 1e). Likewise, in the samples of two cohorts of gastric cancer patients, high expression levels of MG53 were correlated with increased survival probability (Fig. 1f, g), and a reduction in MG53 protein abundance was observed in gastric tumors (Fig. 1h–j). These results indicate that MG53 is reduced in colorectal and gastric tumors and that the reduction of MG53 is associated with poorer clinical outcomes.

### MG53 suppresses gastrointestinal cancer cell proliferation via cell cycle arrest at G1 phase

To reveal the mechanism underlying the tumor-suppressive function of MG53, we firstly examined MG53 expression in several cancer cell lines, and found that colorectal cancer cell HCT116 had endogenous MG53 expression. Next, we overexpressed MG53 in HCT116 and found that upregulation of MG53 markedly attenuated (Supplementary Fig. 1a, b), whereas knockdown of MG53 promoted, cell proliferation (Supplementary Fig. 1c), consistent with the previous observations.^33^

**Fig. 2:**
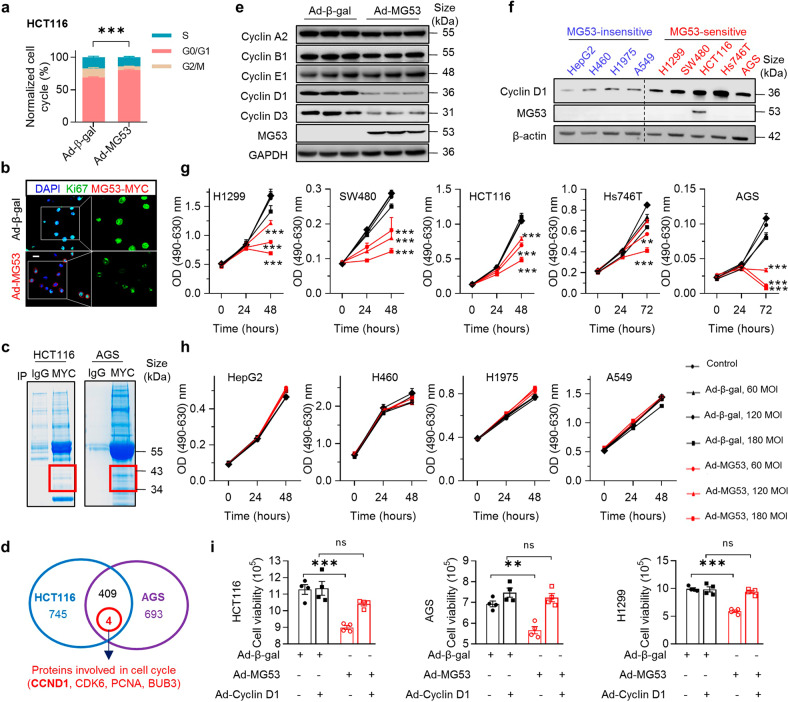
MG53 induces G1 arrest via repressing cyclin D1. **a** Averaged data of flow cytometry showing MG53 induced cell cycle arrest in G1 phase in HCT116 (*n* = 8). **b** Representative images of immunofluorescent signal of Ki67 in AGS cells with or without MG53 overexpression; *n* = 7. **c** HCT116 and AGS cell lysates were resolved on SDS-PAGE gel and stained with coomassie blue before mass spectrometry (MS) analysis. The red frame indicates the area on the SDS-PAGE gel that was excised for MS analysis. **d** Venn diagram showing the number of MG53-interacting proteins identified by MS. The blue circle represents the collection of proteins that interacted with MG53 in HCT116 cells, and the purple circle denotes the pool of proteins interacted with MG53 in AGS cells. The 409 proteins shared by these two collections were analyzed by *KOBAS (*http://kobas.cbi.pku.edu.cn/*)*, and four cell cycle regulation-related proteins were identified (in red circle). **e** Overexpression of MG53 resulted in downregulation of D cyclins, but not other types of cyclins in AGS cells; *n* = 3. **f** Representative western blots showing the protein levels of MG53 and cyclin D1 in MG53-sensitive and -insensitive cell lines; *n* = 3. **g**, **h** Results of MTT assay showing the proliferation of human cancer cell lines in the presence of MG53 overexpression; *n* = 6. **i** Results of cell viability assay showing that overexpression of cyclin D1 attenuated cell death induced by MG53; *n* = 4. Scale bar = 50 μm in (**b**). Data were presented as mean ± s.e.m., and were analyzed using two-tailed unpaired *t* test. ns not significant, **P < 0.01, and ***P < 0.001 as compared with the corresponding controls

In line with its inhibitory effect on cell proliferation, MG53 overexpressing resulted in G1 arrest in both HCT116 (with endogenous MG53 expression; Fig. 2a and Supplementary Fig. 1d) and gastric cancer AGS cells (without endogenous MG53 expression, Supplementary Fig. 1e, f). Consequently, when MG53 was overexpressed, fewer cells could enter S phase and initiate DNA synthesis, as evidenced by the reduced amount of Ki67-positive cells identified by immunofluorescent staining (Fig. 2b and Supplementary Fig. 1g).

### MG53 induces G1 arrest via repressing cyclin D1

To understand how MG53 induces G1 arrest, we searched for the MG53-interacting proteins that involve in cell cycle regulation by overexpressing MYC-tagged MG53 in HCT116 or AGS cells and analyzing the MG53-interacting proteins by mass spectrometry. 409 proteins potentially associated with MG53 in both HCT116 and AGS cells (Supplementary Table S1), and 4 of which were involved in cell cycle regulation based on KOBAS analysis (Fig. 2c, d).^34^ These included cyclin D1 (Supplementary Table S2) and CDK6, the key regulators of G1/S transition. Indeed, cyclin D1 and D3 abundance were reduced by overexpressing MG53, while other cyclins were not affected, suggesting that the regulatory effect of MG53 is specific towards D type cyclins (Fig. 2e). Interestingly, exogenous MG53 expression could dose-dependently repress the proliferation of cancer cells with relatively abundant cyclin D1 protein (Fig. 2f, g), but had very limited effects on cells with little cyclin D1 (Fig. 2f, h). Importantly, overexpressing cyclin D1 restored the cell proliferation suppressed by MG53 in multiple cancer cell lines (Fig. 2i). Collectively, these data indicate that MG53 may interfere cancer cell cycle progression via downregulating cyclin D1 protein abundance.

Cyclin D1 exerts its function by coupling with CDK4/6 to release the inhibitory effect of Rb on E2F family members by phosphorylation, enabling the expression of E2F target genes involved in DNA synthesis. Consistently, overexpressing MG53 attenuated phosphorylation of Rb, whereas specific shRNA-mediated inhibition of MG53 expression had the opposite effects (Supplementary Fig. 1h–k). Moreover, the expression of the E2F downstream targets, c-Myc and c-Jun, were decreased following MG53 overexpression, while increased when MG53 expression was knocked down (Supplementary Fig. 1h–k). Thus, the cell cycle arrest effect of MG53 might be mainly mediated by downregulating cyclin D1.

### MG53 facilitates cyclin D1 degradation via proteasome

**Fig. 3:**
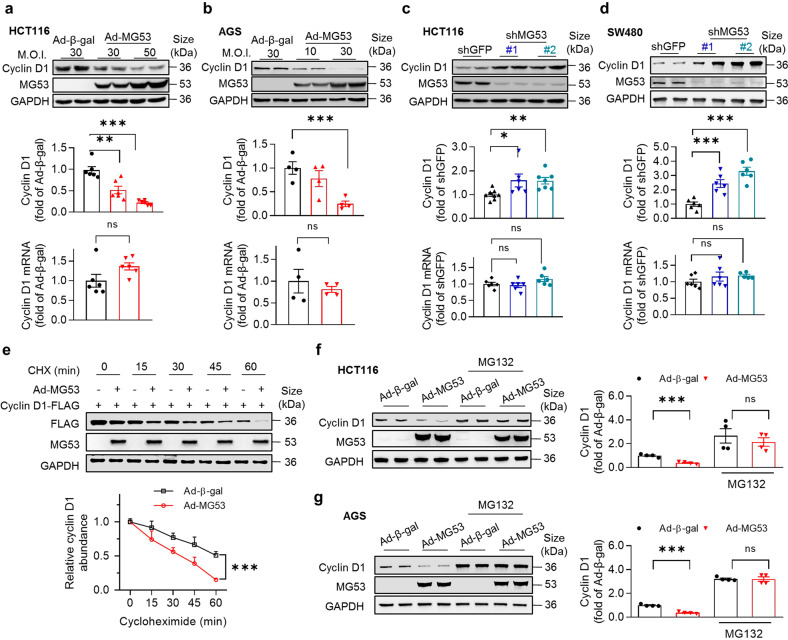
MG53 reduces cyclin D1 protein abundance and accelerates its turnover. **a**, **b** Results of western blots and RT-qPCR showing that overexpression of MG53 dose-dependently downregulated cyclin D1 at the protein levels without altering its mRNA levels in HCT116 (**a**) and AGS (**b**) cells; *n* ≥ 4. **c**, **d** Results of western blots and RT-qPCR showing cyclin D1 protein and mRNA levels in HCT116 (**c**) and SW480 (**d**) cells when MG53 expression was inhibited by specific shRNA; *n* = 6. **e** Representative western blots and statistical result showing that MG53 accelerated cyclin D1 protein turnover in HEK293 cells; *n* = 3. **f**, **g** Western blots showing that MG53-induced cyclin D1 degradation was blocked by proteasome inhibitor MG132 (10 μM, 12 h) in HCT116 (**f**) and AGS (**g**) cell lines; *n* = 6. Data are presented as mean ± s.e.m. and analyzed by two-tailed unpaired *t* test. ns, not significant, *P < 0.05, **P < 0.01, and ***P < 0.001 as compared to the corresponding controls

To further delineate the molecular mechanism responsible for MG53-induced downregulation of cyclin D1, we first overexpressed MG53 in HCT116 and AGS cells and observed dose-dependent decreases of cyclin D1 at the protein level (Fig. 3a, b), while shRNA-mediated inhibition of MG53 markedly increased cyclin D1 in the HCT116 and SW480 colorectal cancer cells (Fig. 3c, d). It is noteworthy that cyclin D1 mRNA levels were not altered with the changes in MG53 expression (Fig. 3a–d), whereas MG53 accelerated cyclin D1 protein turnover (Fig. 3e and Supplementary Fig. 2c, d). Moreover, the downregulation of cyclin D1 by MG53 was abolished by a proteasome inhibitor MG132 (Fig. 3f, g). These results suggest that MG53 may regulate cyclin D1 expression by post-transcriptional mechanisms, and the proteasome pathway is essentially involved.

### MG53 constitutes an E3 ligase of cyclin D1 and mediates its ubiquitination-dependent degradation

**Fig. 4:**
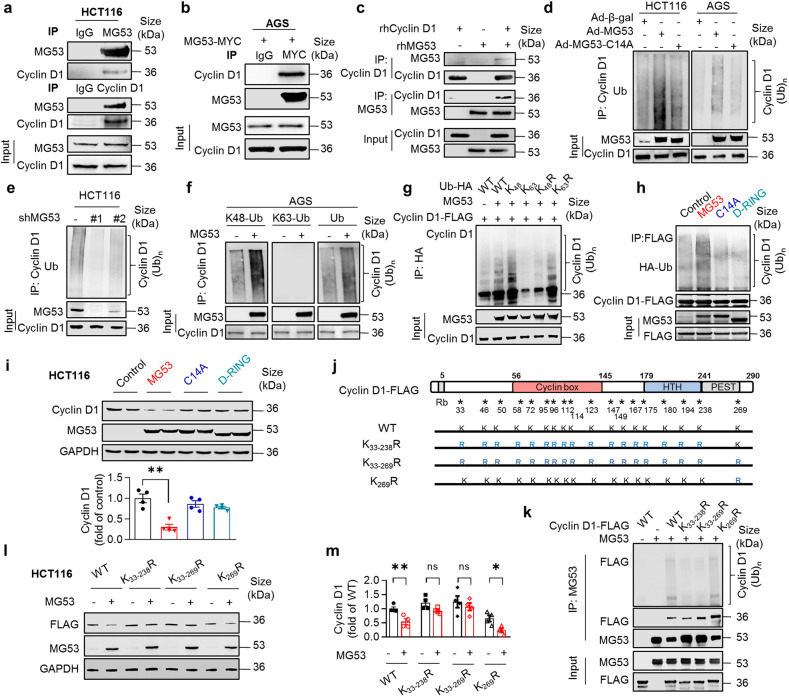
MG53 constitutes an E3 ligase targeting cyclin D1 for ubiquitination-dependent degradation. **a** Co-immunoprecipitation (co-IP) of endogenous MG53 with cyclin D1 in HCT116 cells. **b** Co-IP of MYC-tagged MG53 with endogenous cyclin D1 in AGS cells. **c** Co-IP of synthesized recombinant human MG53 (rhMG53) and cyclin D1 (rhCyclin D1) in vitro. **d** Ubiquitination of endogenous cyclin D1 was enhanced by overexpressing MG53 but not E3 ligase-inactive mutant MG53-C14A in HCT116 and AGS cells. **e** Ubiquitination of endogenous cyclin D1 was abated by silencing MG53 in HCT116 cells. **f** Representative western blots showing the K48-linked or K63-linked polyubiquitination of endogenous cyclin D1 in AGS cells. **g** Representative western blots of ubiquitination of exogenous cyclin D1 by MG53 with expressing wild type or mutant ubiquitin in HEK293 cells; WT, wild type ubiquitin; K_48_ or K_63_, ubiquitin mutant containing only one lysine at position 48 or 63 while the rest of the lysine residues were mutated to arginine; K_48_R or K_63_R, ubiquitin mutant with lysine 48 or lysine 63 mutated to arginine. **h** Western blots showing MG53 mutants devoid of E3 ligase activity could not mediate the ubiquitination of cyclin D1 in HEK293 cells. **i** Representative western blots and averaged data showing MG53 mutants devoid of E3 ligase activity failed to repress the expression of cyclin D1; *n* = 6. **j**, **k** Representative western blots (**k**) showing polyubiquitination of FLAG-tagged wild type (WT) and various lysine mutants of cyclin D1 (**j**) in HEK293 cells with or without MG53 expression. **l**, **m** Western blots (**l**) and averaged data (**m**) showing MG53 failed to downregulate cyclin D1 K_33-238_R and K_33-239_R mutants; *n* = 4. **a**–**h**, **k** Three independent experiments were performed with similar results. Data are presented as mean ± s.e.m. and analyzed by two-tailed unpaired *t* test. ns, not significant, *P < 0.05 and **P < 0.01 as compared to the corresponding controls

Studies have shown that MG53 is a bona fide E3 ubiquitin ligase.^23^ Co-immunoprecipitation (co-IP) assay revealed a strong interaction between MG53 and cyclin D1 in HEK293 cells when both proteins were overexpressed (Supplementary Fig. 3a), which was confirmed by co-IP of endogenous cyclin D1 with MG53 in HCT116 (Fig. 4a) and with exogenously overexpressed MG53 in AGS cells (Fig. 4b). The direct interaction between the two proteins was also evidenced by an in vitro co-IP of synthesized human recombinant proteins MG53 (rhMG53) and cyclin D1 (rhCyclin D1) (Fig. 4c). Using various truncations of cyclin D1 to perform co-IP with MG53, we further identified the cyclin box as the most crucial domain for cyclin D1 to bind to MG53 (Supplementary Fig. 3b–e).

By interacting with cyclin D1, MG53 catalyzed the endogenous cyclin D1 ubiquitination in HCT116 and AGS cells (Fig. 4d), as well as rhCyclin D1 in vitro (Supplementary Fig. 3f). Consistently, knockdown of MG53 reduced the amount of ubiquitinated cyclin D1 in HCT116 cells (Fig. 4e). Similar to cyclin D1, cyclin D3 was also a substrate of MG53 (Supplementary Fig. 3g–i). Importantly, the MG53-mediated polyubiquitination of cyclin D1 was K48-linked, which is mainly for targeting the modified protein for degradation; but not K63-linked (Fig. 4f, g). Moreover, E3 ligase-inactive MG53 mutants, including C14A and D-RING (lacking the entire RING domain), failed to catalyze the ubiquitination of cyclin D1 and D3 (Fig. 4d, h and Supplementary Fig. 3i). As a result, overexpression of MG53-C14A was not able to reduce protein abundance of cyclin D1, D3, or other cyclin proteins (Fig. 4i and Supplementary Fig. 4a), and thus did not disturb the growth of HCT116 or AGS cells (Supplementary Fig. 4b, c), or several other cell lines tested regardless of their sensitivity to MG53 overexpression (Supplementary Fig. 4d–f). Therefore, the MG53-induced degradation of cyclin D1 requires its E3 ligase activity.

It has been shown that mutation of all the lysine residues is required to remove ubiquitination of cyclin D1.^35^ Consistently, a cyclin D1 mutant with all the lysine residues substituted by arginine (K_33-269_R) abolished the ubiquitination by MG53, substantiating that MG53 is an E3 ligase of cyclin D1 (Fig. 4j, k). It has also been reported that K269 is critical for the ubiquitination-dependent degradation by SCF Fbx4/αB-crystallin^36^ and CRL4^AMBRA1^.^15^ Therefore, we examined whether K269 is required for MG53-mediated ubiquitination using mutants K_269_R and K_33-268_R. Surprisingly, K_269_R could not block the ubiquitination by MG53; while on the other hand, with only one lysine left at 269, K_33-268_R was not ubiquitinated (Fig. 4j, k). Consequently, the protein abundance of K_33-269_R or K_33-268_R was not affected, but K_269_R was markedly downregulated by MG53 (Fig. 4l, m). These results indicate that MG53 is different from the previously reported E3 ligases of cyclin D1.

### MG53 suppresses tumor growth in vivo and in vitro

**Fig. 5:**
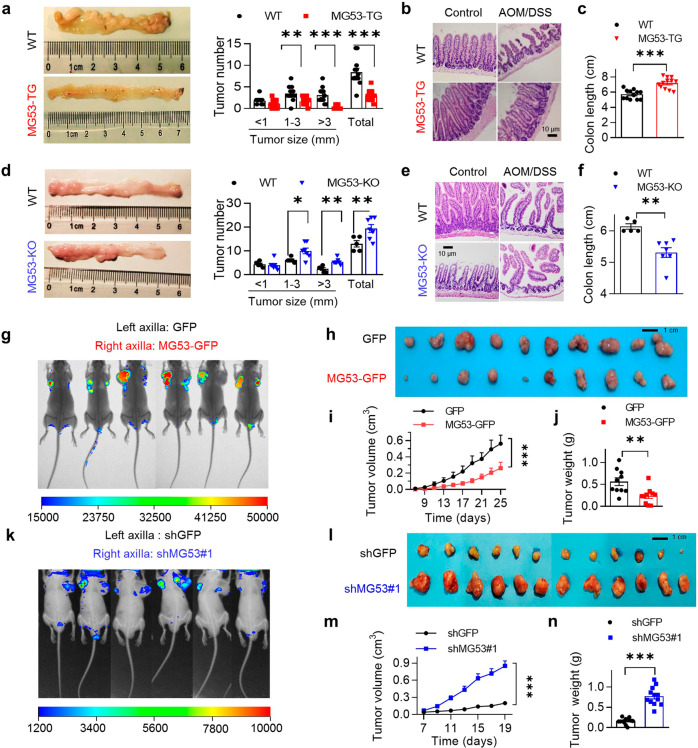
MG53 suppresses tumor growth in mouse models. **a**–**c** Representative images of colon and statistic result of tumor size and number (**a**), representative images of small intestines with H&E staining (**b**), and statistic result of colon length (**c**) of MG53 transgenic mice (MG53-TG, *n* = 12) or their wild type littermates (WT, *n* = 12) after treatment with AOM/DSS. **d**–**f** Representative images of colon and statistic result of tumor size and number (**d**), representative images of small intestines with H&E staining (**e**), and statistic result of colon length (**f**) of MG53 knockout mice (MG53-KO, *n* = 7) or their wild type littermates (WT, *n* = 5) after treatment with AOM/DSS. **g**–**j** Representative CT images of BALB/c nude mice (**g**), image of tumors (**h**), statistic results of volume (**i**) and weight (**j**) of the xenograft tumors derived from HCT116 cells expressing GFP or MG53-GFP; *n* = 10. **k**–**n** Representative CT images of BALB/c nude mice (**k**), image of tumors (**l**), statistic results of volume (**m**) and weight (**n**) of the xenograft tumors derived from HCT116 cells expressing shRNA targeting either GFP (shGFP) or MG53 (shMG53#1); *n* = 12. **b**, **e** Scale bar = 10 μm; **h**, **l** scale bars = 1 cm. Data were presented as mean ± s.e.m., and were analyzed using two-tailed unpaired *t* test. *P < 0.05, **P < 0.01, and ***P < 0.001 as compared with the corresponding controls

Since there is endogenous MG53 expression in colorectal cancer cell HCT116, we established a primary colorectal cancer model induced by azoxymethane/dextran sodium sulfate (AOM/DSS) treatment using MG53 overexpressing (MG53-TG) or deficient (MG53-KO) mice (Supplementary Fig. 5a) as well as their corresponding wild type littermates as controls. 10 weeks of AOM/DSS treatment inducted fewer and smaller tumors in MG53-TG mice (Fig. 5a). Moreover, the MG53-TG mice had better preserved jejunal villi (Fig. 5b) and longer colon (Fig. 5c), indicating that MG53-TG mice had improved intestinal structure and function. In contrast, MG53-KO mice developed more tumors, more seriously disrupted villi structure, and shortened colon length, as compared to their corresponding wild type controls (Fig. 5d–f). These results strongly suggest that MG53 can suppress carcinogenic stimuli-induced tumor growth in vivo.

We then transplanted HCT116 cells overexpressing MG53 into the axilla on one side of BALB/c nude mice and cells expressing GFP into the axilla on the other side of the same animal as a control. Overexpressing MG53 markedly retarded tumor growth as compared with expressing GFP (Fig. 5g). Twenty-five days after implantation, the MG53-overexpressing tumors were significantly smaller (Fig. 5h, i) and lighter (Fig. 5j). In stark contrast, the tumors derived from HCT116 cells with shRNA-mediated MG53 gene silencing displayed markedly accelerated growth (Fig. 5k). Autopsy at the end point (19 days) showed that MG53 deficiency resulted in profoundly increased tumor size and weight (Fig. 5l–n). Remarkably, administration of adenovirus expressing MG53 into HCT116 tumors significantly attenuated tumor growth in vivo, suggesting the therapeutic potential of MG53 (Supplementary Fig. 5b).

### Negative correlation between MG53 and cyclin D1 levels in vivo

**Fig. 6:**
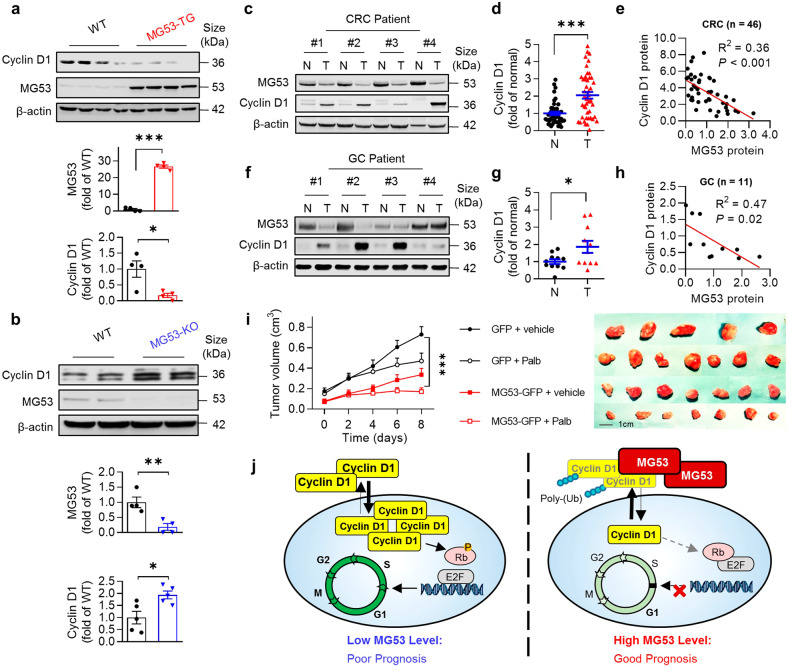
Downregulation of MG53 is associated with increased cyclin D1 protein abundance in tumors. **a**, **b** Representative western blots and averaged data showing the protein levels of MG53 and cyclin D1 in colon tissues from MG53 transgenic (MG53-TG, **a**) and MG53 knockout (MG53-KO, **b**) mice compared to their corresponding wild type littermates (WT) after treatment with AOM/DSS; *n* ≥ 4. **c**, **d** Representative western blots (**c**) and averaged data (**d**) showing the expression of cyclin D1 in tumor tissues (T) compared to the corresponding adjacent normal tissues (N) from colorectal cancer patients; *n* = 46. **e** Regression analysis showing the protein level of MG53 is negatively correlated with that of cyclin D1 in tumor tissues from colorectal cancer patients; *n* = 46. **f**, **g** Representative western blots (**f**) and averaged data (**g**) showing the expression of cyclin D1 in tumor tissues (T) compared to the corresponding adjacent normal tissues (N) from gastric cancer patients; *n* = 11. **h** Regression analysis showing the protein level of MG53 is negatively correlated with that of cyclin D1 in tumor tissues from gastric cancer patients; *n* = 11. **i** Statistical results of tumor size and images of xenograft tumors in BALB/c nude mice derived from HCT116 cells expressing GFP or MG53-GFP treated with vehicle or palbociclib started when xenograft MG53-overexpressing tumors reached an average size of 100 mm^3^; *n* ≥ 6. (Palb, palbociclib at 40 mg/kg administrated daily via oral gavage). **j** Schematic diagram showing E3 ligase MG53-mediated cyclin D1 degradation suppresses cancer growth. **i** GFP and MG53-GFP were HCT116-derived tumors expressing GFP and MG53-GFP, respectively using lentivirus infection. **a**, **b**, **d**, **g**, **i** Data were presented as mean ± s.e.m. and analyzed using two-tailed unpaired *t* test. *P < 0.05, **P < 0.01, and ***P < 0.001 as compared with corresponding normal tissues

In the AOM/DSS-induced mouse colorectal cancer model, cyclin D1 was decreased in the tumors from MG53-TG mice (Fig. 6a and Supplementary Fig. 5c), but increased in those from the MG53-KO mice (Fig. 6b), relative to their corresponding wild type littermates, reinforcing the role of MG53 in facilitating cyclin D1 degradation. Likewise, in the xenograft tumors derived from MG53-overexpressing HCT116, the immunofluorescent signal intensity of cyclin D1 was markedly reduced, while enhanced in the tumors with MG53 expression inhibited by specific shRNA (Supplementary Fig. 5d, e). More importantly, we found that decreased MG53 was associated with upregulation of cyclin D1 in colorectal tumors from patients (Fig. 6c, d and Supplementary Fig. 6a), and the amount of these two proteins were negatively correlated (Fig. 6e). Similarly, a reduction in MG53 protein abundance and negative correlation between MG53 and cyclin D1 levels were observed in gastric tumors (Fig. 6f–h and Supplementary Fig. 6b).

CDK4/6 are kinase partners of cyclin D1 and play important roles in regulating G1/S transition, and their abnormal activation is implicated in various kinds of malignancies. The inhibitors of CDK4/6, palbociclib,^37^ ribociclib,^38^ and abemaciclib,^39^ are FDA- approved drugs to treat breast cancer in combination with endocrine therapy. Their potential applications in other cancer types are also under evaluation in several clinical trials and pre-clinical studies.^40^ However, the innate or acquired resistance towards CDK4/6is limit their clinical implications. Recent studies have suggested that increased cyclin D1 level due to the dysregulation of its E3 ligase CRL4 leads to the resistance towards CDK4/6is.^14–16^ Since MG53 overexpression caused significant downregulation of cyclin D1 (Figs. 3a, b and 6a and Supplementary Fig. 7a), we speculated that ectopic expression of MG53 may sensitize cancer cells to palbociclib. As expected, overexpressing MG53 further inhibited cell proliferation on top of that with palbociclib treatment alone (Supplementary Fig. 7b). In particular, palbociclib failed to induce growth arrest in H1299, but demonstrated robust efficacy in the presence of exogenous MG53 expression. More importantly, MG53 and palbociclib synergistically repressed the growth of HCT116 xenograft tumors (Fig. 6i).

## Discussion

We have made three major findings in this study. First, in more than 80% colorectal and gastric cancer patients tested, the protein level of MG53 is markedly decreased in the tumors compared with that in the adjacent normal tissues, and that MG53 expression level is significantly correlated with the survival outcome of the gastrointestinal cancer patients. Second, in mouse models of xenograft tumor or carcinogen-induced colorectal cancer, overexpression of MG53 profoundly inhibits tumor growth, whereas MG53 deficiency has the opposite effects. Mechanistically, MG53 promotes the ubiquitination-dependent degradation of cyclin D1 via functioning as its E3 ligase, leading to G1 arrest in the cyclin D1-dependent cancer cells. These results indicate that MG53 is a potent tumor suppressor via accelerating cyclin D1 turnover (Fig. 6j).

Multiple lines of evidence have indicated that MG53 is an E3 ligase of cyclin D1 that facilitates its degradation. First, MG53 catalyzes K48-linked polyubiquitination of cyclin D1, while the E3 ligase activity-dead mutant C14A or truncation of MG53 fails to mediate this modification. Second, MG53 accelerates cyclin D1 turnover and represses cell proliferation in an E3 ligase enzyme activity-dependent manner. Third, mutation of lysine residues of cyclin D1 abolishes its ubiquitination by MG53. However, we have found that K_269_ is not required for MG53-mediated ubiquitination, suggesting that MG53 is different from the previously reported E3 ligase complexes of cyclin D1, such as Fbx4/αB-crystallin^36^ or CRL4^AMBRA1^.^15^

Several studies have demonstrated that MG53 is a tumor suppressor,^32,33,41–43^ and may even serve as a diagnostic biomarker.^44,45^ To date, some mechanisms have been proposed to explain the function of MG53 in tumor-suppression, which include promoting stress granule formation in lung cancer cells,^41^ attenuating PI3K/Akt signaling in tongue cancer cells,^33^ impairing lactate production and mTOR signaling downstream of PI3K/Akt in breast cancer cells,^43^ or inhibiting the activity of Ras-related C3 botulinum toxin substrate 1 (RAC1) via catalyzing its ubiquitination in hepatocellular carcinoma cells.^46^ In this study, we have shown that MG53 represses tumor growth via destabilizing cyclin D1. Thus, there was a negative correlation of the amount of MG53 and cyclin D1 in cultured cancer cells and tumors derived from animal models as well as patients with gastric or colorectal cancers. Moreover, MG53 induces cell cycle arrest at G1, and cyclin D1 is the key G1/S regulator that interacts directly with MG53. Most importantly, supplement of cyclin D1 can restore the cell proliferation attenuated by MG53 overexpression. Although we cannot fully exclude other potential mechanisms, our results strongly suggest that the anti-cancer effect of MG53 is largely mediated by facilitating cyclin D1’s ubiquitination and subsequent degradation in multiple cancer cell types that depend on cyclin D1 for cell proliferation. Therefore, understanding the detailed mechanism(s) of action of MG53 in each particular cancer type is fundamental for implementation of precision medicine in cancer treatment.

MG53 has originally been reported to play an important role in membrane repair,^25,26^ which has inspired the application of recombinant MG53 protein (rhMG53) in treating acute injury of a variety of organs.^30,31,47–49^ In a recent study using mouse xenograft model of colorectal cancer cell SW620/AD300, injection of rhMG53 inhibits tumor growth and displays synergistic antitumor effect with doxorubicin.^42^ We showed significant therapeutic effects of adenoviral delivery of MG53 in delaying HCT116 tumor progression in vivo. Moreover, as the dysfunction of cyclin D1 E3 ligases is involved in desensitizing of cancer cells towards CDK4/6is in clinical practice,^14–16^ we utilized MG53 in combination with palbociclib and successfully enhanced the sensitivity of cancer cells to palbociclib and further repressed tumor growth. These proof-of-concept tests demonstrate the possible application scenarios of MG53 in cancer therapy.

In summary, we have shown that MG53 is a tumor suppressor that targets cyclin D1 for ubiquitination-dependent degradation. Upregulation of MG53 is sufficient to suppress tumor growth. Most importantly, increased expression of MG53 not only inhibits tumor growth in animal models, but also is associated with markedly improved survival probability of cancer patients. These findings highlight the therapeutic potential of MG53 in treating cancers with high cyclin D1 abundance and improving the efficacy of CDK4/6is.

## Materials and methods

### Research subject

Patient samples were obtained from Beijing Cancer Hospital and Institute, Beijing, China; and Wuhan Union Hospital, Hubei, China (for detailed patient’s information, see (Supplementary Table S3). All the donors were informed and giving consent before tissue samples were collected. The procedures were approved by the Human Ethics Committees of Beijing Cancer Hospital & Institute and Wuhan Union Hospital and performed in compliance with standards of research involving human subjects. Human tissue microarrays were purchased from US Biomax (CO801a, CO803a, and ST1004a) and analyzed by Alena Biotechnology (Xi’an, China).

### Animal models

All animal study protocols were approved by the Institutional Animal Care and Use Committee of Peking University. All the experiments were carried out in accordance with the Guide for the Care and Use of Laboratory Animals by Association for Assessment and Accreditation of Laboratory Animal Care (AAALAC). The animals were maintained in the AAALAC-accredited Laboratory Animal Center at Peking University, Beijing, China. Male mice were randomly assigned to experiment groups for treatments. MG53 overexpressing transgenic mice (MG53-TG) and MG53 knockout mice (MG53-KO) were generated as described previously.^23^

For the xenograft mouse model, male BALB/c nude mice at 6 weeks of age were injected with 3 × 10^6^ HCT116 cells expressing either MG53-GFP or MG53-specific shRNA (shMG53) into one axilla, and cells expressing GFP or GFP-specific shRNA (shGFP) were transplanted into the axilla on the other side of the same animal as a control. Once palpable tumors were established, a caliper was used to measure their sizes every 2 days. The formula (A × B^2^)/2 was used to calculate the volume of a tumor, where A and B were the tumor length and width, respectively. After the indicated time course, mice were imaged. The xenograft tumors were dissected and weighed. For tests of palbociclib treatments, tumors were monitored till the average size of xenograft MG53-overexpressing tumors reached 100 mm^3^, at which point mice were randomly assigned to treatment group of either vehicle or palbociclib (40 mg/kg) via oral gavage for 7 consecutive days. For evaluating therapeutic potential of MG53, 3 × 10^6^ HCT116 cells were xenoplanted into male 6-week-old BALB/c nude mice. Once the average tumor size was around 200 mm^3^, the mice were randomized into two groups and injected with either adenovirus expressing β-gal or MYC-tagged MG53. The adenovirus was injected very slowly into the tumor at two different sites once every 2 days (1 × 10^7^ viral particles per mouse). Mice were sacrificed after they received five doses of adenovirus.

For AOM/DSS-induced colorectal carcinogenesis, mice were injected with AOM intraperitoneally (10 mg/kg, Sigma-Aldrich, #A5486). 7 days after AOM injection, 2.5% DSS (MP Biomedicals, #0216011090) was administered via drinking water for 7 consecutive days, then replaced with normal drinking water for another 14 days. At the end of 10 weeks after repeating this treatment scheme for 3 times, the mice were sacrificed. Colons were collected, and feces were washed off with Ringer’s solution (115 mM NaCl, 1.2 mM MgCl_2_, 1.2 mM CaCl_2_, 25 mM NaHCO_3_, 2.4 mM K_2_HPO_4_, 0.4 mM KH_2_PO_4_, and 2% PMSF, pH 7.4). Colons were slit open longitudinally and the number of tumors were counted. The snap-frozen small intestine, colon, and tumors were stored at −80 °C for further analysis.

### Reagents and materials

Unless specifically indicated, all the chemicals used were from Sigma-Aldrich. The antibodies used in this study are listed in Supplementary Table S4. MG53 recombinant protein was purified as described previously.^50^ Cyclin D1 recombinant protein (#230-00261-10) was from Raybiotech. Palbociclib (#S1116) was from Selleck Chemicals. Cycloheximide (CHX, #A8244) was from APExBio.

### Constructs

The expression vector of HA-Ubiquitin was from Addgene (RRID: Addgene_18712). The full-length MG53 cDNA was amplified from a human ORFeome v8.1 Entry library by PCR and the expression vector was generated using pcDNA4/TO/myc-HisB Expression Vector (Invitrogen, #V86320). Cyclin D-FLAG expression vectors were also constructed by inserting PCR product of the human ORFeome library into the vector c-FLAG pcDNA3 (RRID: Addgene_20011). The constructs expressing MG53 D-RING and truncated cyclin D1 were generated from their corresponding wild type full-length expression vectors. All mutant and truncation expression vectors were generated using a Q5 Site-Directed Mutagenesis Kit (NEB, #E0554S) as described by the manual.

### Cell culture, cell counting, adenoviral or lentivirus infection, and plasmid transfection

All the cells, including HEK293 (Cat# CRL-1573, RRID: CVCL_0045), HepG2 (Cat# HB-8065, RRID: CVCL_0027), H460 (Cat# HTB-177, RRID: CVCL_0459), H1975 (Cat#CRL-5908, RRID: CVCL_1511), A549 (Cat#CCL-185, RRID: CVCL_0023), H1299 (Cat#CRL-5803, RRID: CVCL_0060), AGS (Cat#CRL-1739, RRID: CVCL_0139), Hs746T (Cat#HTB-135, RRID: CVCL_0333), SW480 (Cat#CCL-228, RRID: CVCL_0546), and HCT116 (Cat#CCL-247, RRID: CVCL_0291) cells, were from ATCC. HEK293, HCT116, HepG2, and Hs746T cells were cultured in Dulbecco’s modified Eagle’s medium (Gibco, #12800-017). H1975, A549, H1299, and AGS cells were maintained in RPMI1640 (Sigma-Aldrich, R4130), all of which were supplied with 10% fetal bovine serum (Sigma-Aldrich, F8687). SW480 cells were cultured in Leibovitz’s L-15 medium (Gibco, #11415-064) with 15% fetal bovine serum. Cell number was determined by Cellmeter Auto T4 (Nexcelom). When cells reached 90% confluency, gene transfer was performed by adenoviral or lentiviral infection (Suzhou GenePharma), or plasmid transfection using Lipofectamine 3000TM (Invitrogen, L3000015). The sequences of the MG53-specific shRNAs are as follows:

shMG53#1: 5′-GACTGAGTTCCTCATGAAATA-3′,

shMG53#2: 5′-GGGTTGAAGCTTAGGTCTCCT-3′.

### Cell viability and cell death

Cells were seeded in 24-well plates at 4 × 10^4^ per well and infected with the indicated adenoviruses. Cell proliferation was assessed with MTT assay (Amresco, #0793). Specifically, cells were incubated with MTT at 37 °C for 4 h. The medium was then replaced with dimethyl sulfoxide and cells were solubilized. MTT cleavage was quantified using a spectrophotometer to measure absorption at 490 nm with absorption at 630 nm subtracted as background. CellTiter-Glo^®^ Luminescent Cell Viability Assay (Promega, #G7570) was utilized to assess cell viability. For monitoring cell proliferation at real-time, 4 × 10^4^ cells in a well of 24-well plate were subjected to the measurement by an impedance-based real-time instrument system (xCELLigence RTCA DP, ACEA Biosciences Inc.).

### Fluorescence-activated cell sorter analysis

Cells were synchronized using transient serum starvation and infected with the indicated adenovirus. Twenty-four hours after infections, cells were fixed at 4 °C overnight in 75% ethanol, then washed in PBS before stained in 1 mL PI staining solution (50 μg/ml, Solarbio #C0080) containing RNase A (20 μg/mL final concentration, TransGen Biotech #GE101-01) in the dark at room temperature for 30 min. For each sample, around 10,000 PI-stained cells were evaluated with a FACScan flow cytometer (Becton Dickinson).

### Real-time PCR

Total RNA was extracted for reverse transcription and real-time PCR reactions.^23^ The relative mRNA level was determined by normalizing to the level of 18S rRNA. Each real-time PCR (LightCycler 96, Roche) experiment was performed in triplicate, and the data were presented as the average of at least three independent experiments. Primers for real-time PCR are:

18S forward: 5′-GTAACCCGTTGAACCCCATT-3′;

18S reverse: 5′-CCATCCAATCGGTAGTAGCG-3′.

Human cyclin D1 forward: 5′-TGCATGTTCGTGGCCTCTAA-3′;

Human cyclin D1 reverse: 5′-GAACTTCACATCTGTGGCAC-3′.

### Confocal microscopy, co-immunoprecipitation and western blotting

After transfection with the indicated plasmids or infection with the indicated adenovirus, cells were stained with indicated antibodies, and the immunofluorescent images were examined using a confocal microscope (A1RSi+).

Tissue or cell lysate was homogenized in lysis buffer (30 mM HEPES at pH 7.6, 100 mM NaCl, 0.5% Nonidet P-40, and protease inhibitor cocktail (Roche, #04693132001) and lysed on ice for 10 min. The supernatant was collected after 10 min centrifugation at 13,000 rpm and used for western blotting and co-immunoprecipitation. All original and uncropped images of western blots were provided in the supplementary materials (Supplementary Fig. 8).

### Mass spectrometry

Cells were infected with adenovirus expressing c-terminus MYC-tagged MG53. Upon harvest, cell lysate was prepared by centrifugation at 13,000 rpm for 10 min. MG53 was immunoprecipitated with anti-MYC antibody or IgG on protein A Sepharose 4 Fast Flow (GE healthcare #17-5280-02). The non-specific bindings were removed with ice-cold lysis buffer and then loading buffer (BIO-RAD #1610737) was added to elute immunoprecipitated protein complex. The eluent was resolved by SDS–polyacrylamide gel electrophoresis (BIO-RAD #1610183) and stained with Coomassie blue. The specific bands were cut out for mass spectrometry analysis, and the IgG control lane was used to subtract background hit in the MS.

For LC-MS/MS analysis, peptides were separated using a Thermo-Dionex Ultimate 3000 HPLC system with a 120 min gradient elution at a flow rate of 0.300 μL/min. The analytical column was homemade by packing C-18 resin (300 A, 5 μm; Varian, Lexington, MA, USA) in a fused silica capillary column (75 μm ID, 150 mm length; Upchurch, Oak Harbor, WA, USA). Mobile phase A and B are 0.1% formic acid, and 100% acetonitrile and 0.1% formic acid, respectively. Xcalibur3.0 software was used to control and process data obtained from an Orbitrap Fusion mass spectrometer operating in a data-dependent acquisition mode. A single Orbitrap scan across full mass spectrum (350–1550 *m/z*, 120,000 resolution) is followed by 3 s data-dependent MS/MS scans in an Ion Routing Multipole at 30% normalized collision energy (HCD). The MS/MS spectra from each LC-MS/MS run were identified using Proteome Discovery searching algorithm (version 1.4) to search against the selected database.

### Ubiquitination assay

Cells were incubated with MG132 (10 μM, Sigma-Aldrich, #474790) for 12 h and then harvested in ice-cold PBS. Then cells were re-suspended in RIPA buffer (200 mM NaCl, 20 mM Tris-HCl at pH 8.0, 1 mM EDTA, 1 mM EGTA, 1% Nonidet P-40, 0.5% sodium deoxycholate, 0.1% SDS, 2.5 mM sodium pyrophosphate, 1 mM β-glycerol phosphate, 1 mM Na_3_VO_4_, protease inhibitor cocktail, and 10 μM MG132) and lysed on ice for 10 min. The ubiquitinated proteins were immunoprecipitated with indicated antibodies from the cell lysate and resolved on SDS-PAGE for western blotting. The in vitro ubiquitination assay of cyclin D1 was carried out using an ubiquitination kit from Enzo Life Science (cat. no. BML-UW9920-0001) and UBE2H was used in ubiquitination reactions.

### Subcellular fractionation

Cytosolic and nuclear fractionation of cells was done using MinuteTM cytoplasmic and nuclear extraction kit (Invent Biotechnologies, #sc-003) following manufacturer’s protocol.

### Kaplan–Meier survival curve analysis

Data was analyzed in *R2: Genomics Analysis and Visualization Platform* (http://r2platform.com). Patients were ranked based on the expression levels of MG53 in their tumors obtained from the TCGA RNAseq and GEO databases. Kaplan–Meier survival analyses and log-rank significance tests were used to compare the survival outcome.

### Statistical analysis

Statistical analyses were performed using GraphPad Prism 8. Data were presented as mean ± s.e.m. The statistical significance of differences between groups were examined using two-tailed unpaired *t* test or paired *t* test. *P* < 0.05 was considered as statistically significant. Reproducibility and statistics were described in detail in the figure legends.

## Supplementary information

Supplementary Materials

Supplementary Table S1

## Data Availability

All data will be available upon reasonable request.
