# Supplementary material for: E3 ligase MG53 suppresses tumor growth by degrading cyclin D1
Source: Signal Transduct Target Ther. 2023 Jul 7;8:263. doi: 10.1038/s41392-023-01458-9 (PMC10326024; doi:10.1038/s41392-023-01458-9)
Supplement: Supplementary file 1 — Supplementary Materials [file 41392_2023_1458_MOESM1_ESM.docx]

Supplementary Materials for

E3 ligase MG53 Suppresses Tumor Growth by Degrading Cyclin D1

Meng Fang^1,2†^, Hong-Kun Wu^3,4†^, Yumeng Pei^1,2†^, Yan Zhang^1,5^, Xiangyu Gao^6^, Yanyun He^1,2^, Gengjia Chen^1^, Fengxiang Lv^1,5^, Peng Jiang^1^, Yumei Li^1^, Wenwen Li^1^, Peng Jiang^7^, Lin Wang^8^, Jiafu Ji^6^*, Xinli Hu^1,3^*, Rui-Ping Xiao^1,2,4^*

Correspondence to: xiaor@pku.edu.cn (R-P. X), huxxx025@pku.edu.cn (X.H.), jijiafu@hsc.pku.edu.cn (J.J.)

**This PDF file includes:**

Figures. S1 to S8

Tables S1 to S4

Supplementary Figures


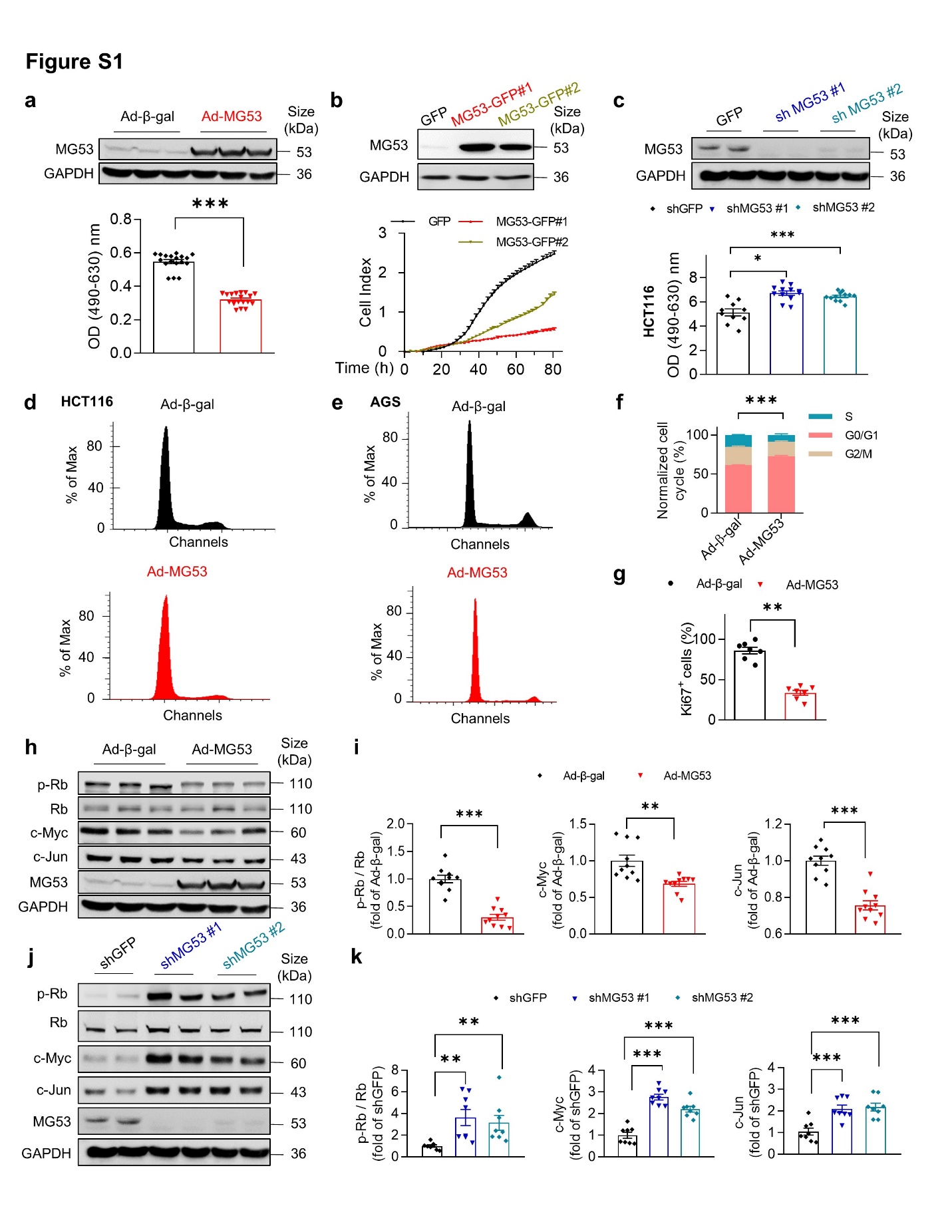


**Fig. S1. MG53 induces aberrant cell cycle progression and G1 arrest. a** Cell proliferation was decreased after overexpressing MG53 in HCT116 cells; *n* = 18. **b** Real time recording of the growth curves of two HCT116 clones overexpressing MG53-GFP and one clone expressing GFP over 80 hr using xCELLigence® RTCA DP (ACEA Biosciences Inc.). Lentiviruses used in these experiments were validated by western blot. **c** Cell proliferation was promoted after knocking down MG53 expression in HCT116 (*n* = 10). **d-f** Flow cytometry and averaged data showing MG53 induced cell cycle arrest in G1 phase in HCT116 (**d**; *n* = 8) and AGS cells (**e**, **f**; *n* = 6). **g** Averaged data of immunofluorescent signal intensity of Ki67 in AGS cells with or without MG53 overexpression; *n* = 7. **h-k** Representative western blots and averaged data showing the protein levels of phosphorylated Rb (p-Rb) at S780, c-Myc, and c-Jun after overexpressing (**h, i**) or knocking down MG53 (**j, k**) in HCT116 cells. In (**a)** and (**d-i**), Ad-β-gal and Ad-MG53, cells infected with adenovirus expressing β-gal and MG53, respectively. In (**b**), GFP and MG53-GFP were HCT116 cells expressing GFP and MG53-GFP, respectively. In (**c**, **j-k**) shGFP and shMG53 were cells expressing shRNA targeting GFP and MG53, respectively. Data are presented as mean ± s.e.m and analyzed by two-tailed unpaired *t*-test. **P* < 0.05, ***P* < 0.01, and ****P* < 0.001 as compared to the corresponding controls.

**
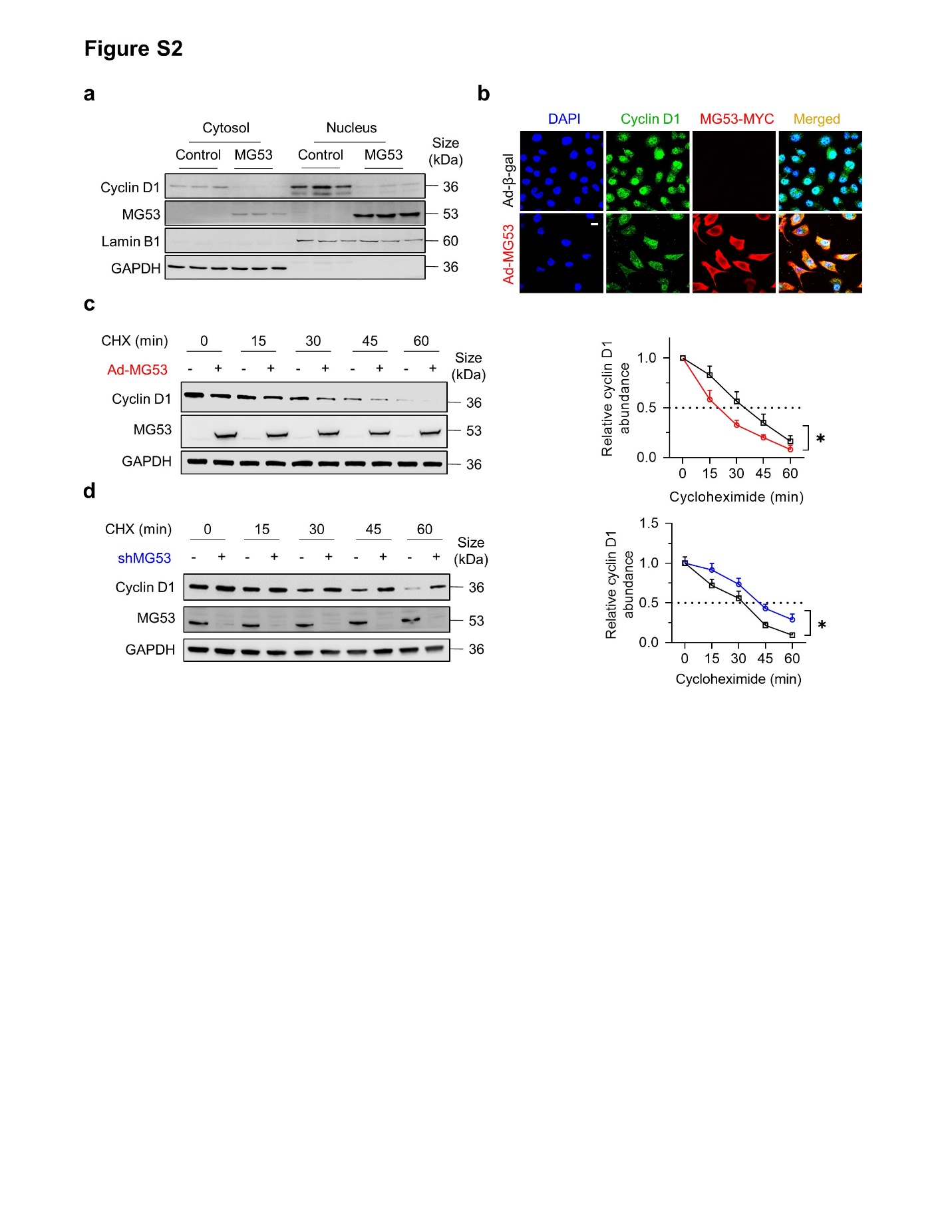
**

**Fig. S2. MG53 negatively regulates cyclin D1 protein abundance** **and accelerates its turnover. a** Western blots of cyclin D1 in cytosolic and nuclear fractions of AGS cells with or without MG53 overexpression; *n* = 3. **b** Confocal images of immunofluorescent staining of cyclin D1 and MYC-tagged MG53 in AGS cells; *n* ≥ 3. **c**, **d** Representative western blots and averaged data of endogenous cyclin D1 protein turnover by overexpressing MG53 (**c**) or silencing MG53 (**d**) in HCT116 cells. Scale bar = 10 μm in **b**. Control and MG53 were cells transfected with empty vector or vectors expressing MG53. Ad-β-gal and Ad-MG53, cells infected with adenovirus expressing β-gal, MYC-tagged MG53, respectively. shGFP and shMG53 were HCT116 cells expressing shRNAs targeting GFP or MG53, respectively using lentiviral infection. Data are presented as mean ± s.e.m and analyzed by two-tailed unpaired *t*-test. **P* < 0.05 as compared to the corresponding controls.


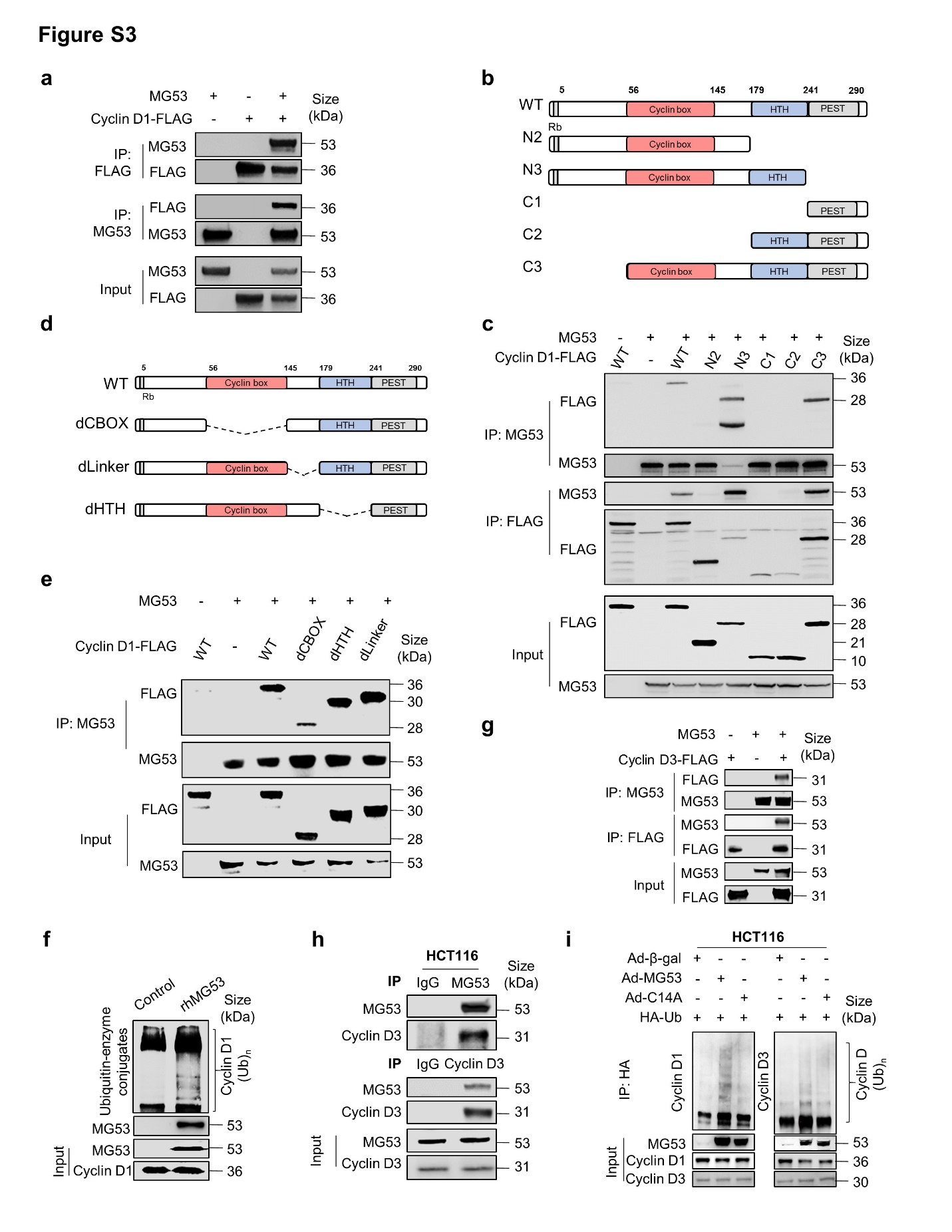


**Fig. S3. MG53 interacts with cyclin D1 and facilitates its ubiquitination. a** Co-IP and reverse co-IP of MG53 with cyclin D1 in HEK293 cells. **b-e** Co-IP (**c**, **e**) of MG53 with different cyclin D1 truncations (**b, d**) in HEK293 cells. **f** Ubiquitination of rhCyclin D1 by rhMG53 *in vitro*. **g** Co-IP and reverse co-IP of MG53 with cyclin D3 in HEK293 cells. **h** Co-IP and reverse co-IP of endogenous MG53 with cyclin D3 in HCT116 cells. **i** Ubiquitination levels of endogenous cyclin D3 after overexpression of MG53 or MG53-C14A in HCT116 cells. In panel (**a-e** and **g**), cells were transfected with vectors expressing MG53and FLAG-tagged cyclin D protein or its different truncations. In panel (**i**), Ad-β-gal, Ad-MG53, and Ad-C14A, cells infected with adenovirus expressing β-gal, MYC-tagged MG53, and MYC-tagged MG53-C14A mutant, respectively. For all the panels with blots, 3 independent experiments were performed with similar results.


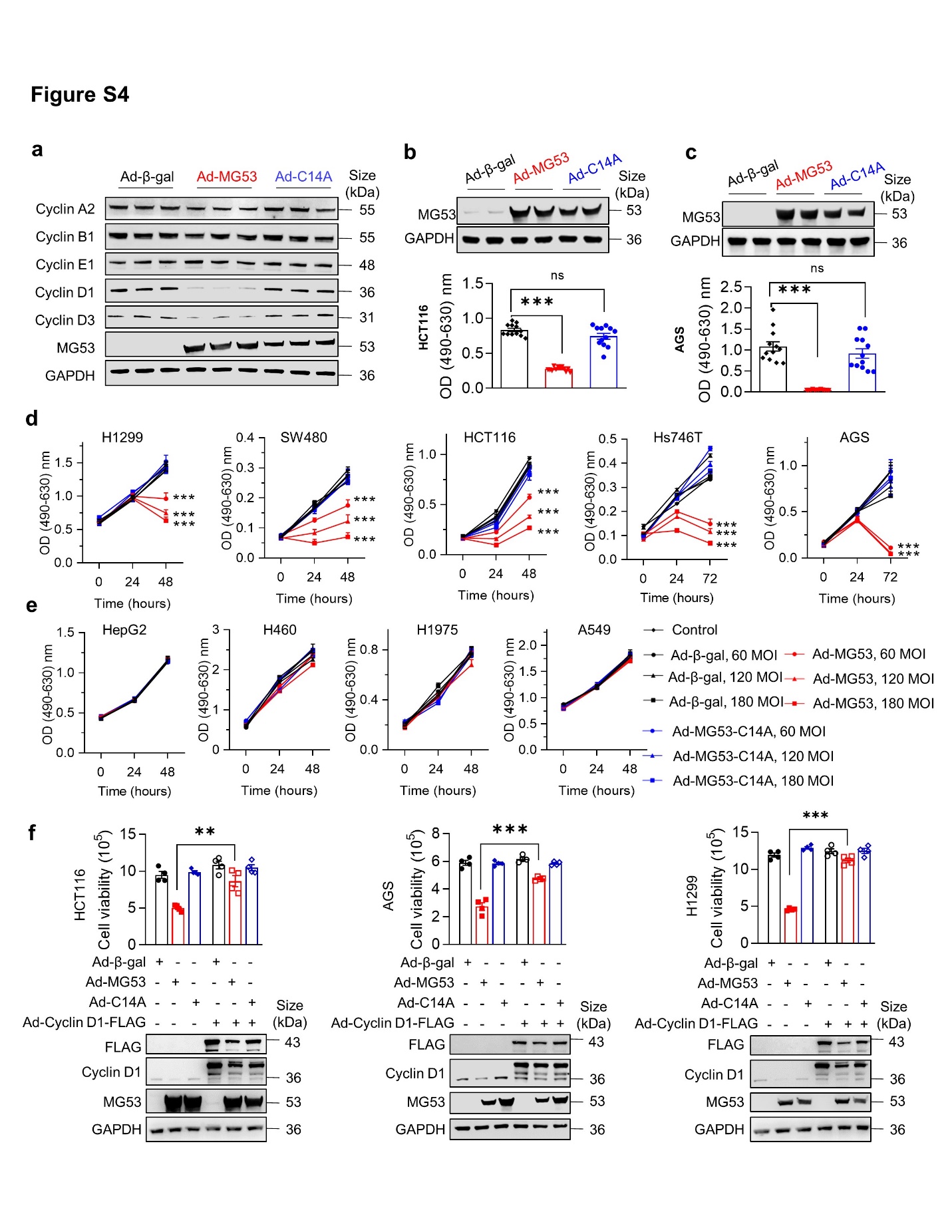


**Fig. S4.** **MG53 C14A mutant fails to repress cyclin D1 expression and cancer cell proliferation. a** Western blots indicating that MG53 E3 ligase activity-dead mutant C14A could not reduce the protein level of cyclin D or other cyclins in AGS cells; *n* = 3. **b, c** Representative western blots and results of MTT assay showing that overexpression of MG53-C14A in HCT116 had no effect on cell proliferation in HCT116 (**b**) or AGS (**c**) cells; *n* = 12. **d**, **e** Results of MTT assay showing the proliferation of human cancer cell lines with the overexpression of MG53 or MG53-C14A; *n* = 8. **f** Representative western blots and results of cell viability assays showing that MG53-C14A had no effect on cell death regardless of cyclin D1 overexpression in HCT116, AGS, or H1299 cells; *n* = 4. Ad-β-gal, Ad-Cyclin D1-FLAG, Ad-MG53, and Ad-C14A, cells infected with adenovirus expressing β-gal, FLAG-tagged cyclin D1, MYC-tagged MG53, and MYC-tagged MG53-C14A mutant, respectively. Data were presented as mean ± s.e.m, and were analyzed using two-tailed unpaired *t*-test. ns, not significant, ***P* < 0.01, and ****P* < 0.001 as compared with the corresponding controls.


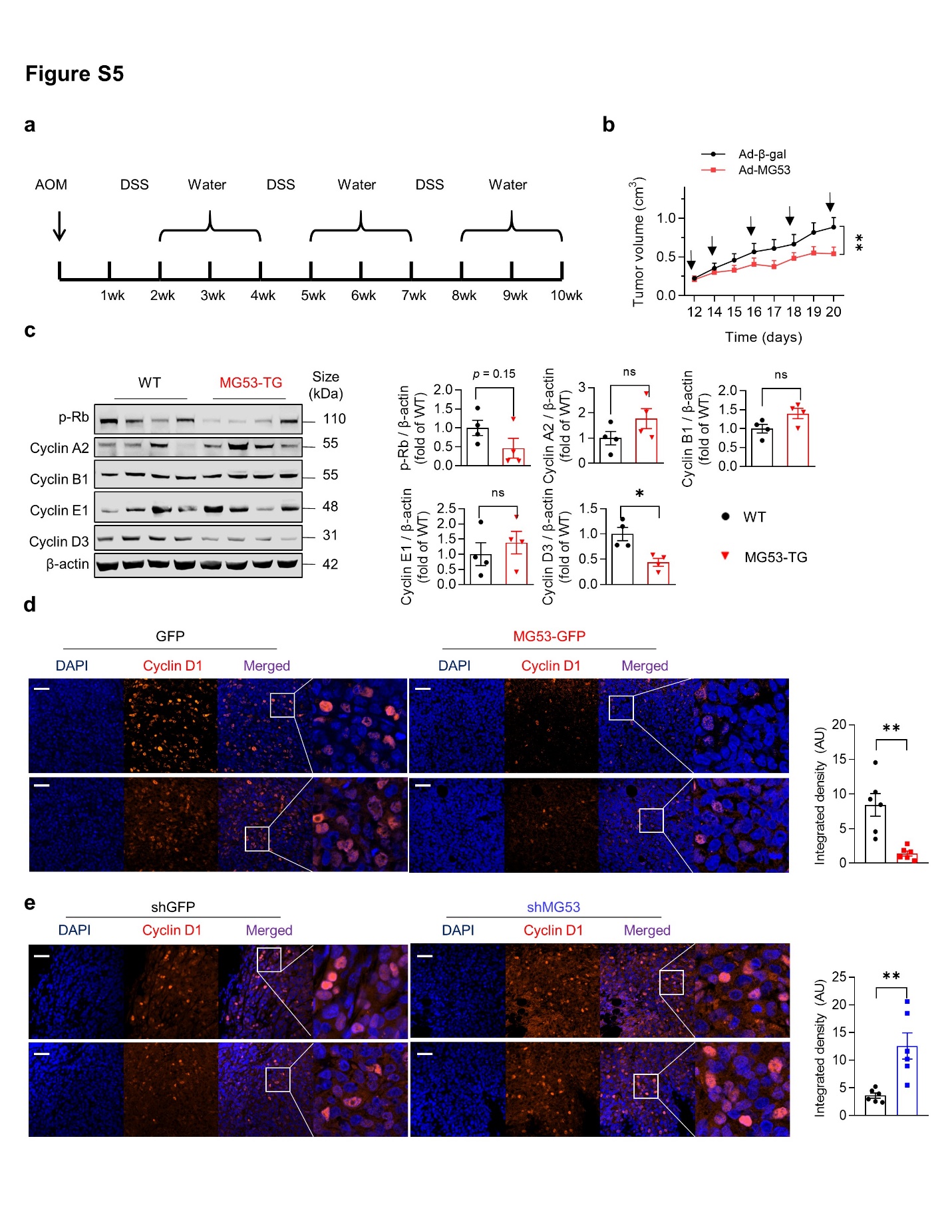


**Fig. S5. MG53 downregulates cyclin D1 in tumors. a** Schematic diagram showing the experiment procedure of AOM/DSS treatment to induce colon cancer in mice. **b** Statistic result of tumor volume of the xenograft HCT116 tumors showing that intratumoral injection of adenovirus expressing MG53 repressed tumor growth *in vivo*. The arrows indicated the time point of intratumor administration of adenovirus expressing β-gal or MYC-tagged MG53 (1×10^7^ viral particles per mouse) after implantation of HCT116; *n* = 6 for each group. **c** Representative western blots and averaged data of cyclin proteins in the colon tissue from MG53 transgenic mice (MG53-TG) compared to their wild type littermates (WT) after treatment with AOM/DSS; *n* = 4. **d, e** Immunofluorescent staining and statistic results of integrated density of cyclin D1 in tumors derived from HCT116 cells expressing GFP or MG53-GFP (**d**), and shGFP or shMG53 (**e**). Scale bar = 50 μm in **d** and **e**. For panels (**b**) to (**e**), data are presented as mean ± s.e.m and analyzed using two-tailed unpaired *t*-test. ns, not significant, **P* < 0.05, ***P* < 0.01 as compared to the corresponding controls.


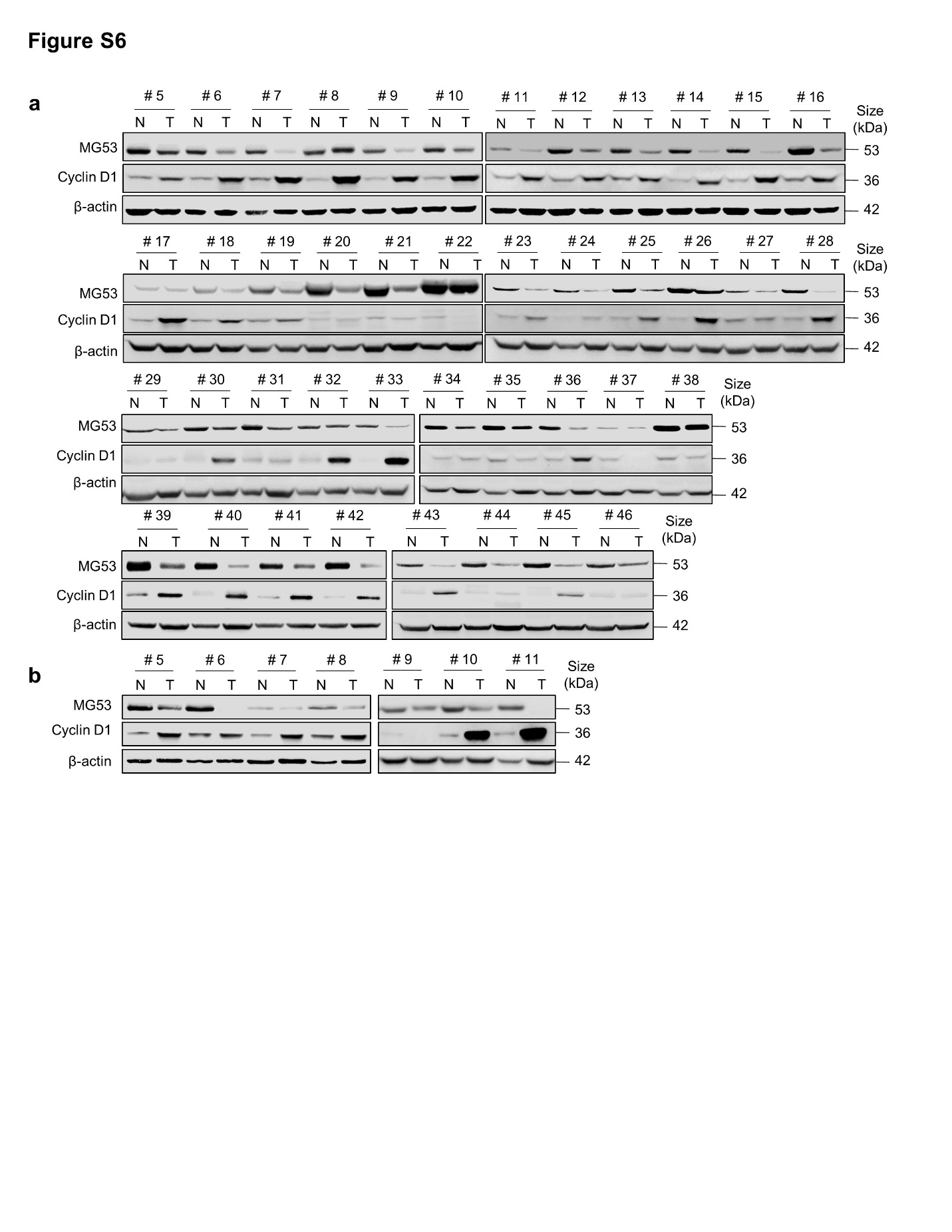


**Fig. S6. Decreased MG53 is associated with increased cyclin D1 in tumor samples from colorectal and gastric cancer patients. a**, **b** Representative western blots showing MG53 and cyclin D1 protein levels in tumor tissue (T) compared to the corresponding adjacent normal tissue (N) from the same colorectal cancer patient (**a**) or gastric cancer patient (**b**).


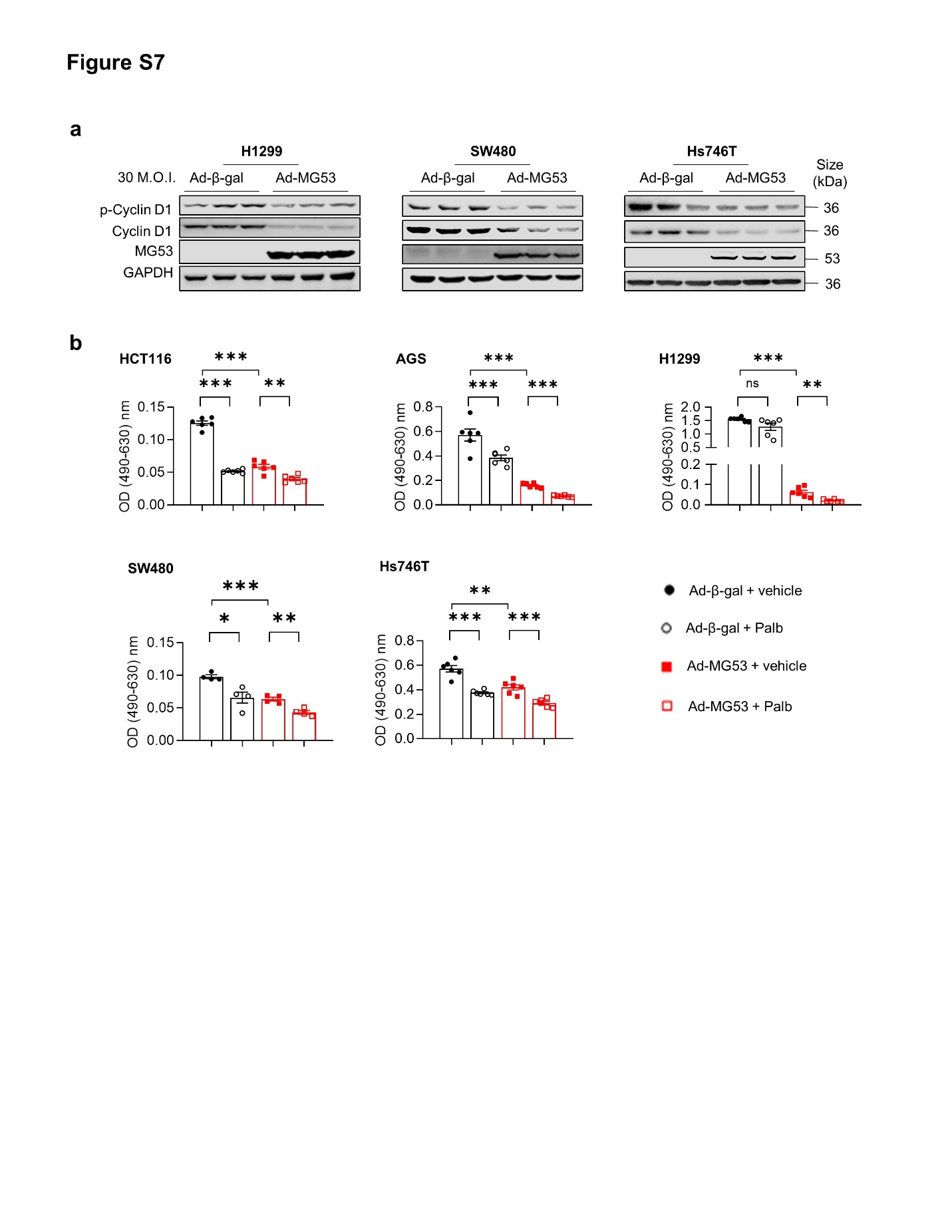


**Fig. S7. Ectopic expression of MG53 sensitizes cancer cells to palbociclib. a** Representative western blots showing protein levels of phosphorylated and total cyclin D1 after overexpressing MG53 in H1299, SW480, and HS746T cells; *n* = 3. **b** The synergistic effect of MG53 and palbociclib (Palb, 1 μM, 48 h) in HCT116, AGS, H1299, SW480, and HS746T cells; *n* ≥ 5. Ad-β-gal and Ad-MG53, cells infected with adenovirus expressing β-gal, MYC-tagged MG53, respectively. Data are presented as mean ± s.e.m and analyzed using two-tailed unpaired *t*-test. **P* < 0.05, ***P* < 0.01, and ****P* < 0.001 as compared to the corresponding controls.


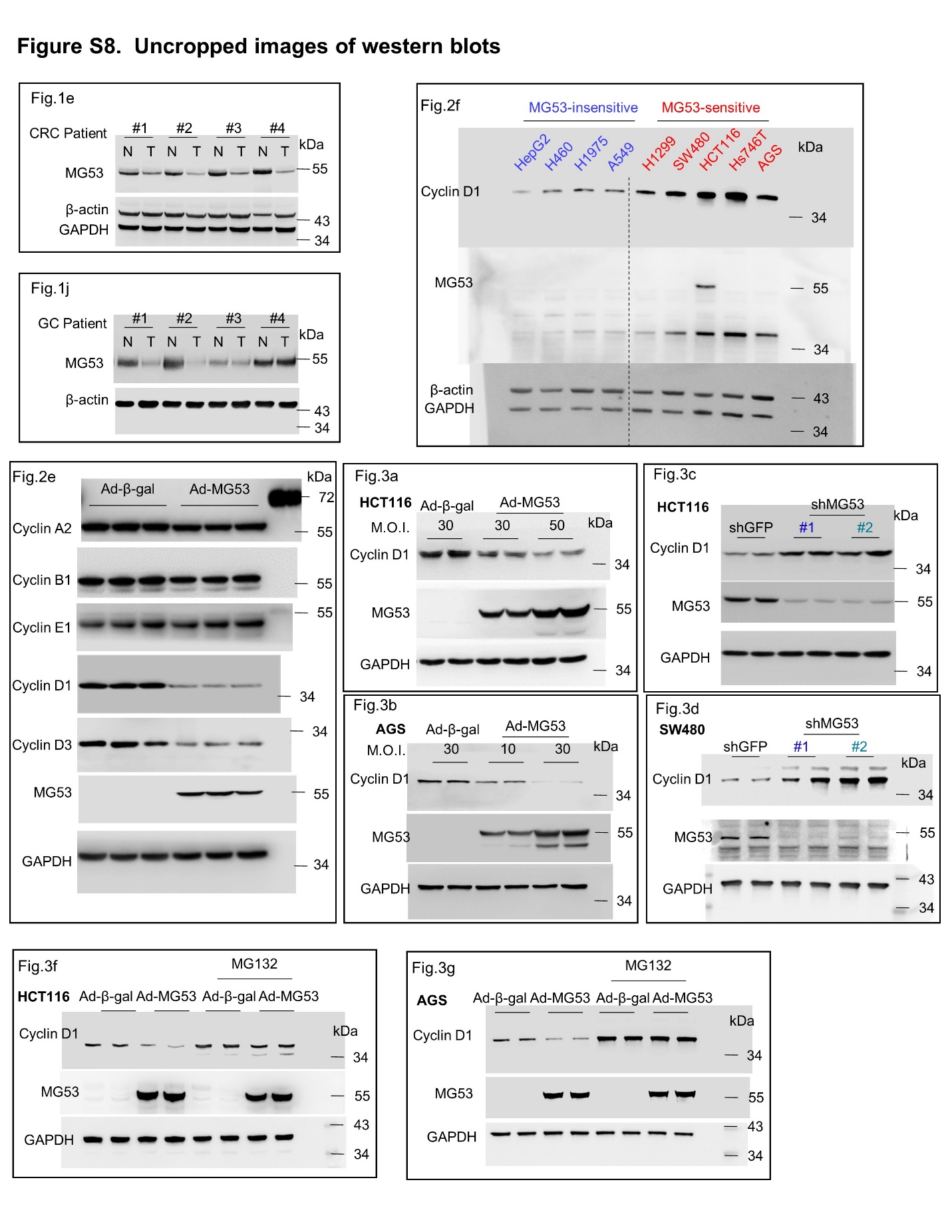

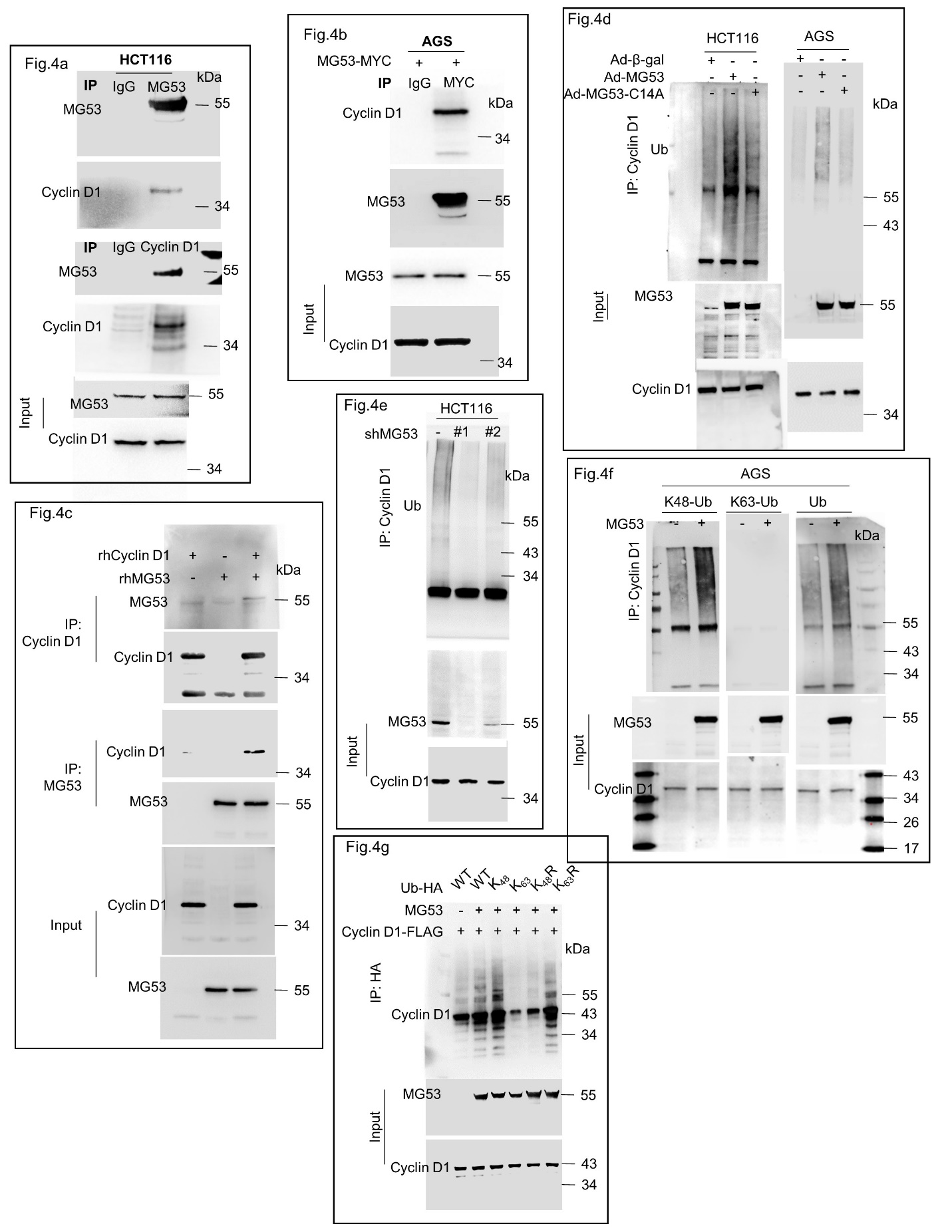

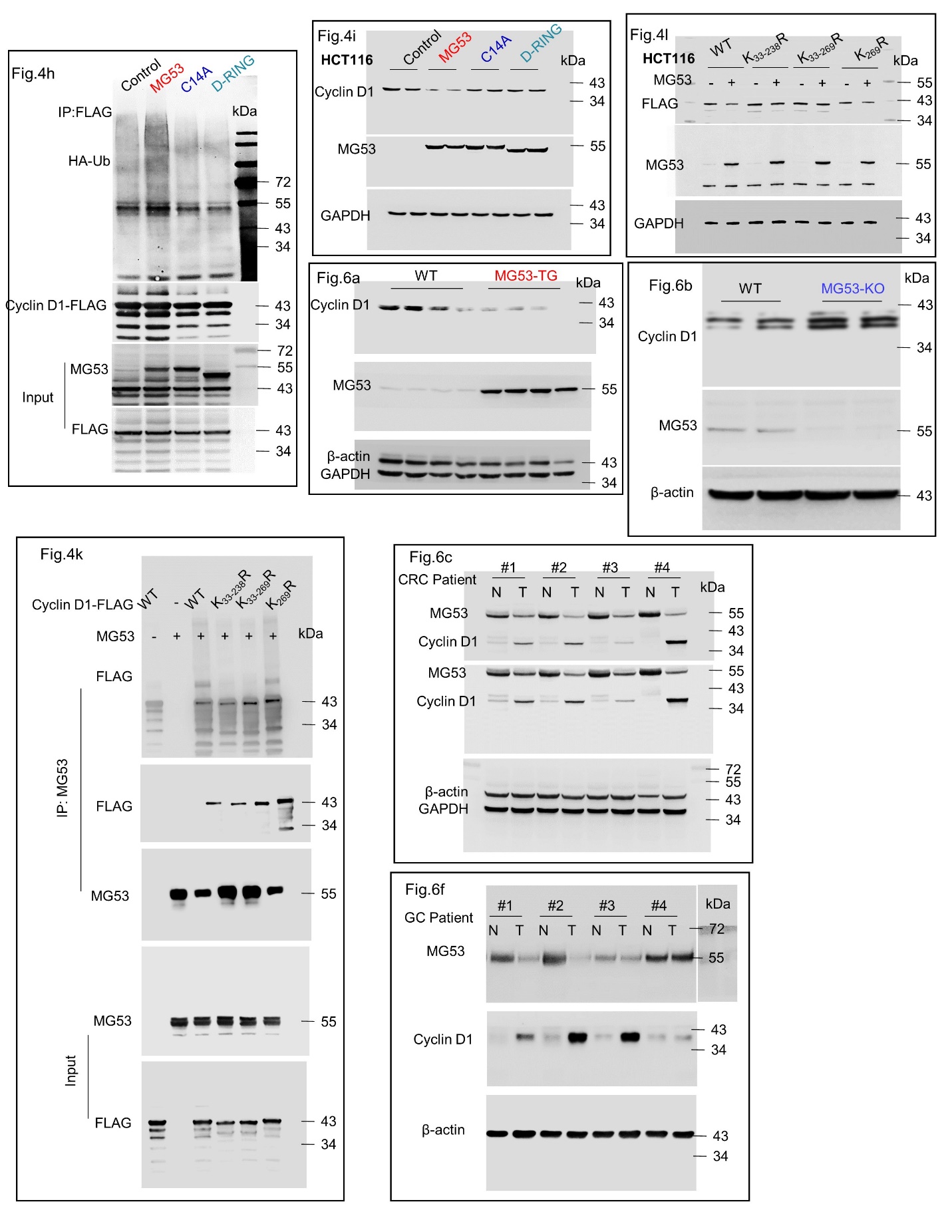

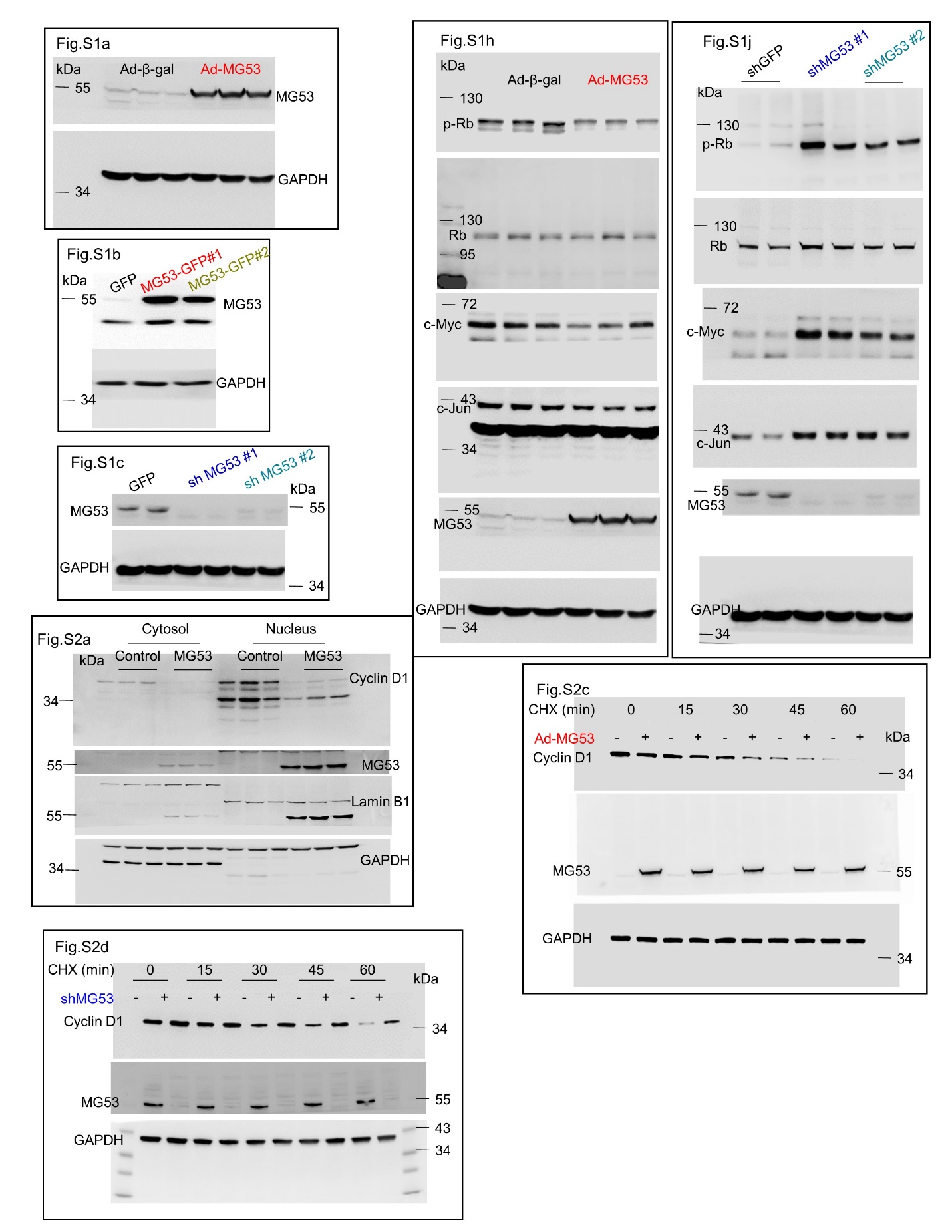

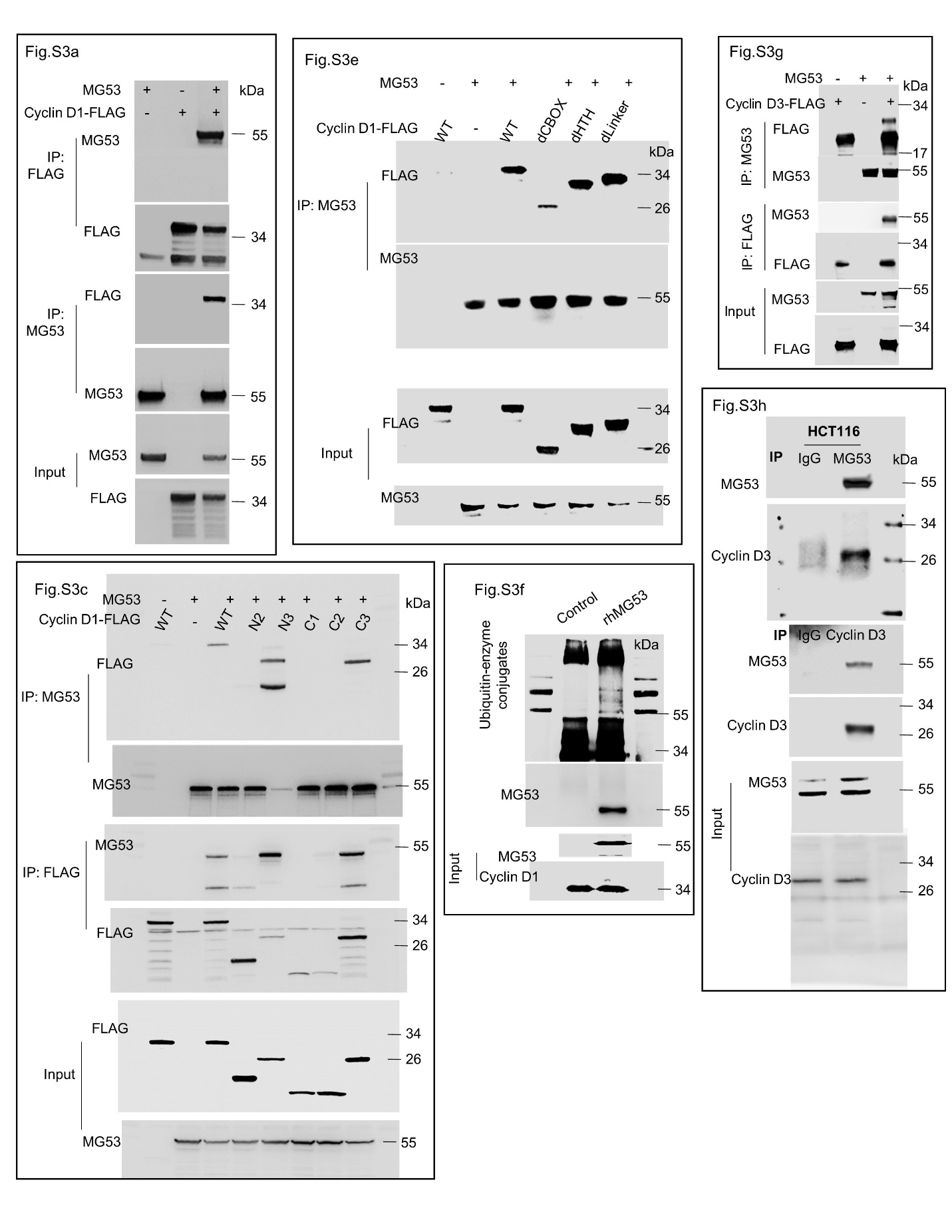

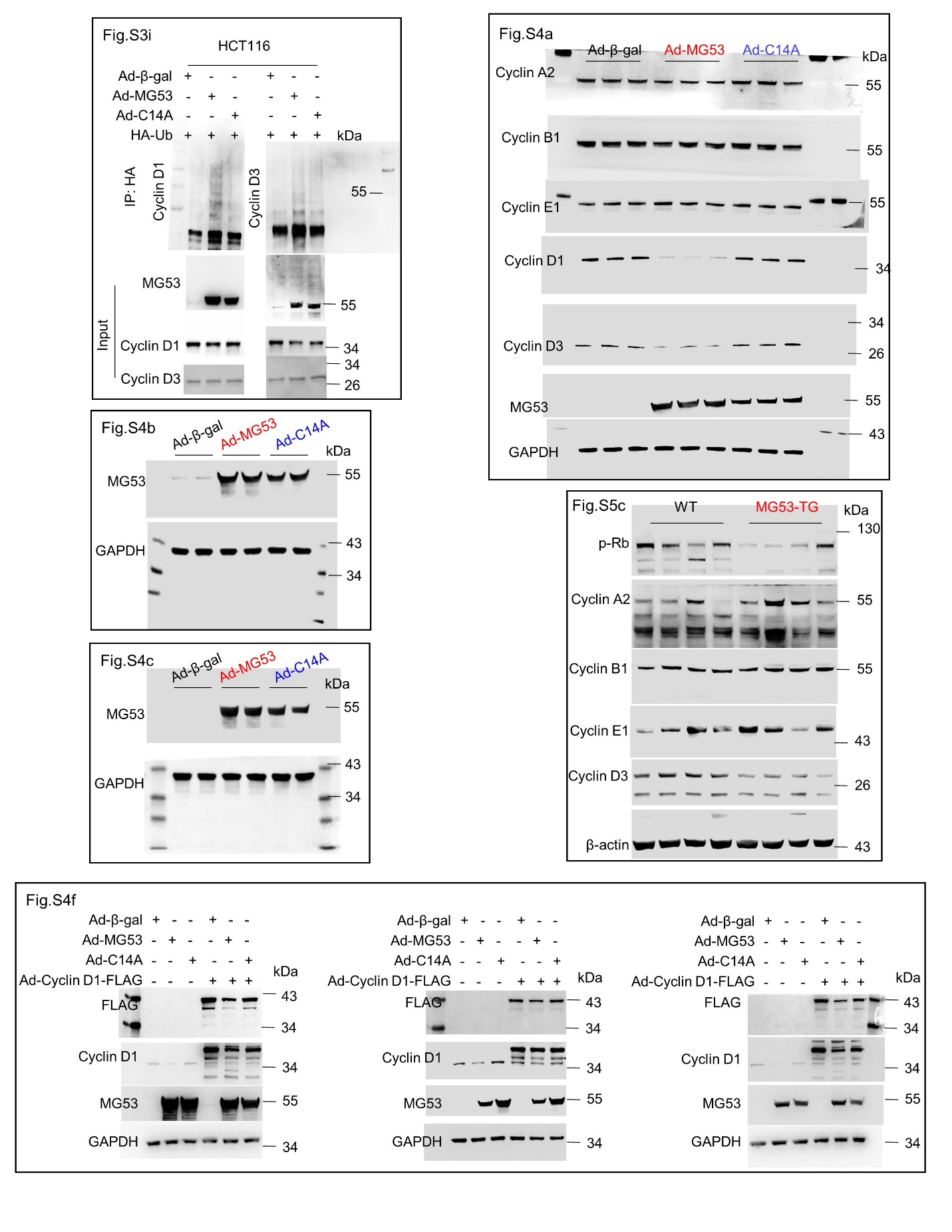

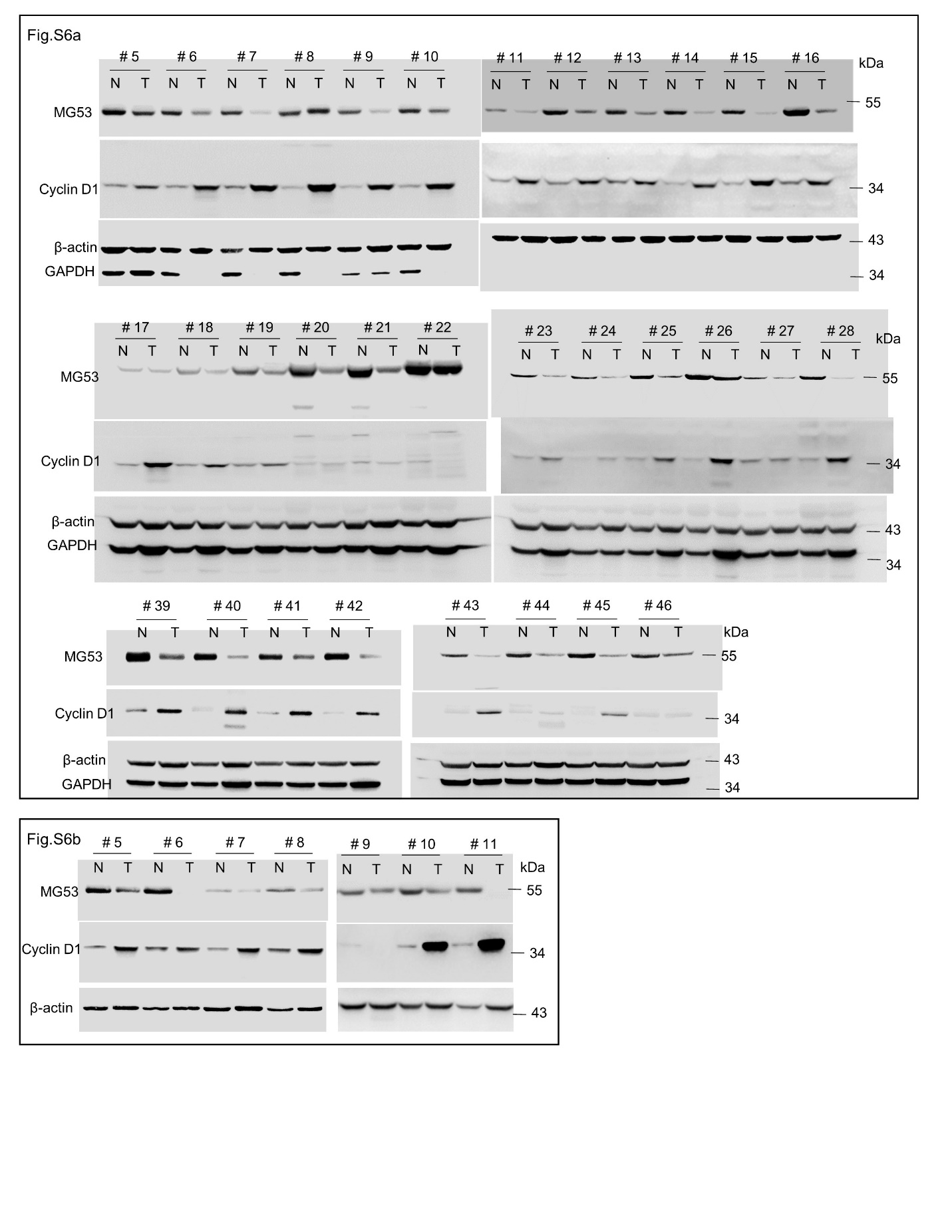

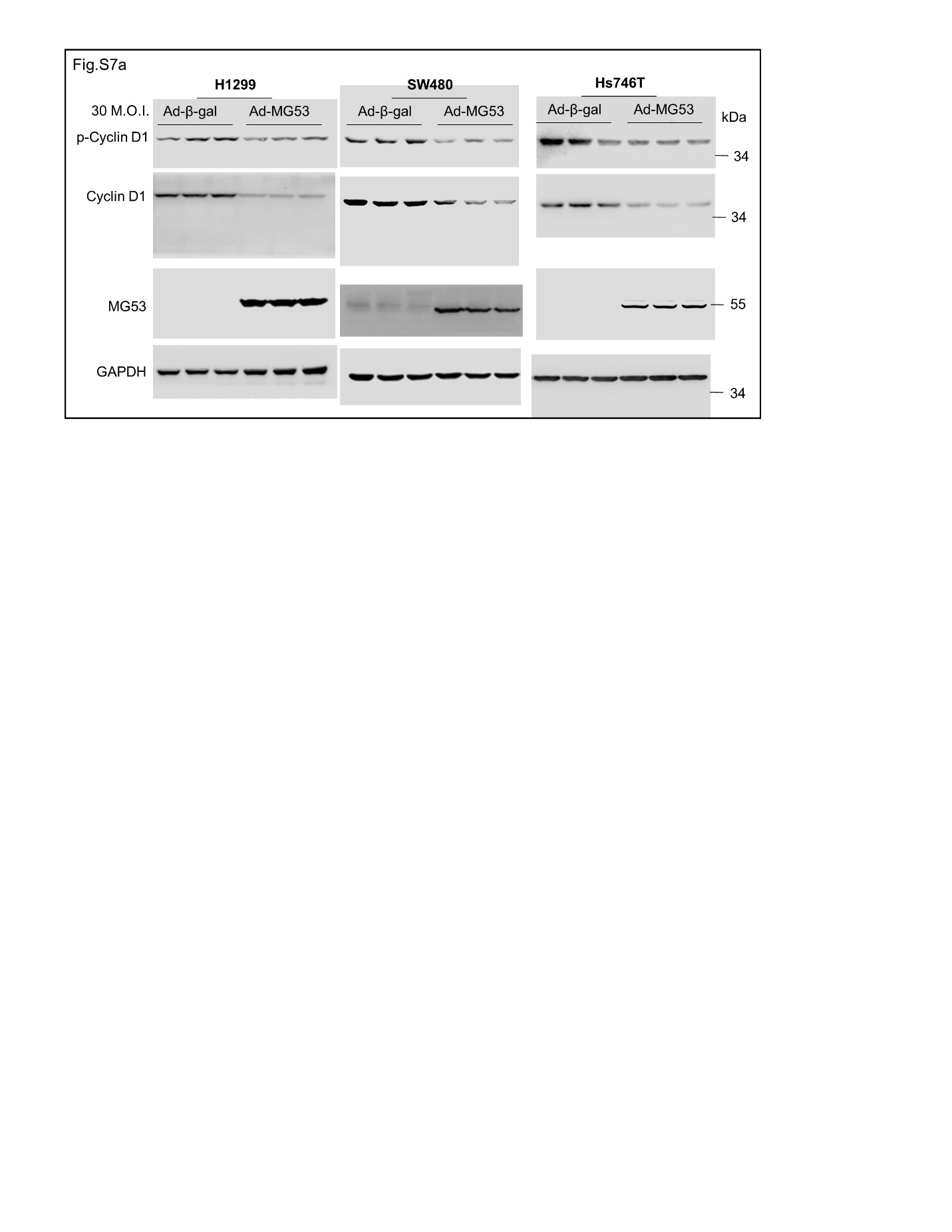


**Supplementary Tables**

**Table S1. Results of MS and KORAS analysis (separate file).**

**Table S2. CCND1 PSM counts and peptide information for the IP-MS.**


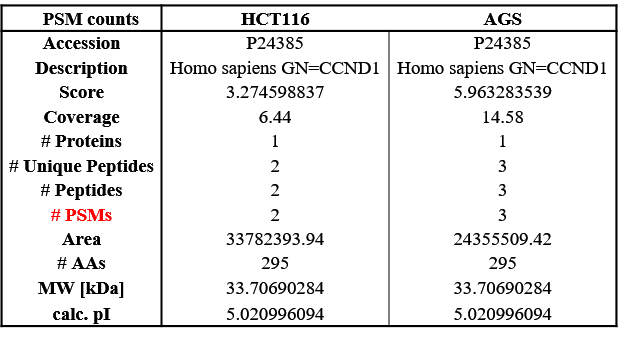

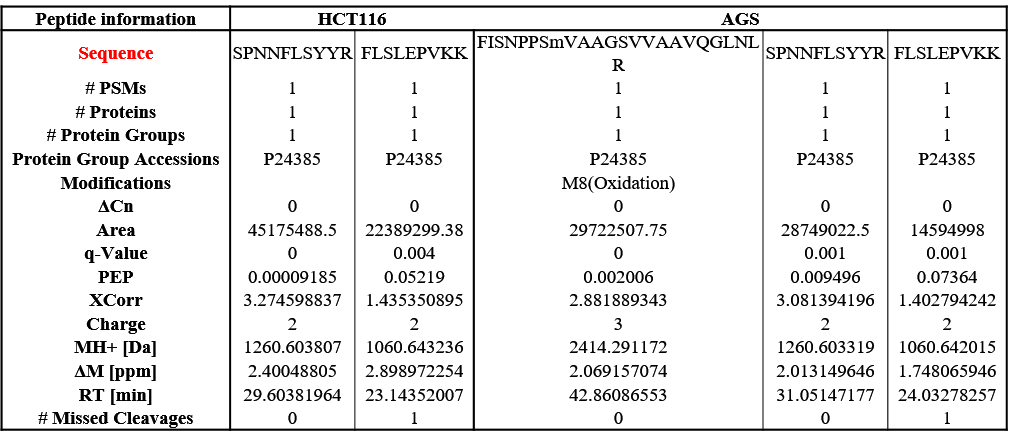


**Table S3. Patient information.**

| CRC sample from Beijing, China | | | |
| --- | --- | --- | --- |
| Sex | **Age** | **Organ(Anatomic Site)** | **TNM** |
| M | 72 | Colon Adenocarcinoma | T3N0M0 |
| F | 52 | Colon Adenocarcinoma | T3N1M0 |
| F | 58 | Colon Adenocarcinoma | T3N1M1 |
| F | 51 | Colon Adenocarcinoma | T3N1M0 |
| F | 35 | Colon Adenocarcinoma | T3N0M0 |
| F | 84 | Colon Adenocarcinoma | T4aN0M0 |
| M | 44 | Colon Adenocarcinoma | T4aN0M0 |
| F | 75 | Colon Adenocarcinoma | T3N0M0 |
| M | 75 | Colon Adenocarcinoma | T4aN0M0 |
| M | 76 | Colon Adenocarcinoma | T3N1M0 |
| F | 70 | Colon Adenocarcinoma | T4aN2M0 |
| F | 82 | Colon Adenocarcinoma | T3N0M0 |
| F | 81 | Colon Adenocarcinoma | T3N0M0 |
| F | 40 | Colon Adenocarcinoma | T3N1bM0 |
| M | 62 | Colon Adenocarcinoma | T3N0M0 |
| M | 59 | Colon Adenocarcinoma | T4bN0M0 |
| M | 49 | Colon Adenocarcinoma | T3N0M0 |
| M | 74 | Colon Adenocarcinoma | T3N0M0 |
| M | 75 | Colon Adenocarcinoma | T3N0M0 |
| M | 66 | Colon Adenocarcinoma | T3N0M0 |
| M | 49 | Colon Adenocarcinoma | T3N0Mx |
| M | 72 | Colon Adenocarcinoma | T3N1cMx |
| F | 73 | Colon Adenocarcinoma | T3N0Mx |
| M | 66 | Colon Adenocarcinoma | T3N1Mx |
| F | 77 | Colon Adenocarcinoma | T3N0M0 |
| M | 73 | Colon Adenocarcinoma | T3N0M0 |
| F | 53 | Colon Adenocarcinoma | pT3N0Mx |
| M | 85 | Colon Adenocarcinoma | pT4bN2aM1 |
| F | 65 | Colon Adenocarcinoma | pT3N2Mx |
| M | 67 | Colon Adenocarcinoma | pT3N1b |
| F | 49 | Colon Adenocarcinoma | pT3N2b |
| M | 62 | Colon Adenocarcinoma | pT3N0Mx |
| M | 54 | Colon Adenocarcinoma | pT3N1 |
| F | 75 | Colon Adenocarcinoma | pT3N1 |
| M | 77 | Colon Adenocarcinoma | pT3N1a |
| M | 66 | Colon Adenocarcinoma | pT3N0Mx |
| F | 63 | Colon Adenocarcinoma | PT3N1aMx |
| CRC sample from Wuhan, China | | | |
| Sex | **Age** | **Organ(Anatomic Site)** | **Pathological differentiation** |
| F | 75 | Colon Adenocarcinoma | Moderately differentiated |
| F | 72 | Colon Adenocarcinoma | Moderately differentiated |
| M | 58 | Colon Adenocarcinoma | Moderately differentiated |
| M | 43 | Colon Adenocarcinoma | Poorly differentiated |
| M | 63 | Colon Adenocarcinoma | Moderately differentiated |
| M | 67 | Colon Adenocarcinoma | Moderately differentiated |
| F | 51 | Colon Adenocarcinoma | Moderately differentiated |
| F | 62 | Colon Adenocarcinoma | Poorly differentiated |
| F | 46 | Colon Adenocarcinoma | Moderately differentiated |
| GC sample from Beijing, China | | | |
| Sex | **Age** | **Organ(Anatomic Site)** | **TNM** |
| M | 77 | Gastric cancer | T3n1m0 |
| M | 59 | Gastric cancer | T2n0m0 |
| M | 51 | Gastric cancer | T3N3bm0 |
| M | 57 | Gastric cancer | T3N0M0 |
| M | 66 | Gastric cancer | T3n3bM0 |
| F | 69 | Gastric cancer | T3N0M0 |
| M | 45 | Gastric cancer | T3N3aM0 |
| M | 68 | Gastric cancer | T4aN0M0 |
| F | 69 | Gastric cancer | T3N3bm0 |
| M | 64 | Gastric cancer | T3N2M0 |
| M | 57 | Gastric cancer | T3n1m0 |

**Table S4. Antibodies used in this study.**

| Antibodies | Origin | Identifier |
| --- | --- | --- |
| Mouse monoclonal anti-FLAG, CloneName/M2 | Sigma-Aldrich | Cat# F1804,  RRID: AB_262044 |
| Mouse monoclonal anti-MYC, CloneName/9E10 | Sigma-Aldrich | Cat# SAB4700447, |
| Rabbit monoclonal anti-HA, CloneName/C29F4 | [Cell Signaling Technology](https://www.cellsignal.com/) | Cat# 3724,  RRID: AB_1549585 |
| Mouse monoclonal anti-Ki67, CloneName/8D5 | [Cell Signaling Technology](https://www.cellsignal.com/) | Cat# 9449,  RRID: AB_2715512 |
| Rabbit monoclonal anti-phospho-Cyclin D1 (Thr286), CloneName/D29B3 | Cell Signaling Technology | Cat# 3300,  RRID: AB_2070561 |
| Rabbit monoclonal anti-Cyclin D1, CloneName/92G2 | [Cell Signaling Technology](https://www.cellsignal.com/) | Cat# 2978,  RRID: AB_2259616 |
| Rabbit polyclonal anti- c-Myc,  CloneName/D84C12 | Cell Signaling Technology | Cat# 5605,  RRID: AB_1903938 |
| Rabbit polyclonal anti- c-Jun,  CloneName/60A8 | [Cell Signaling Technology](https://www.cellsignal.com/) | Cat# 9165,  RRID: AB_2130165 |
| Rabbit monoclonal anti-phospho-Rb (Ser780), CloneName/D59B7 | [Cell Signaling Technology](https://www.cellsignal.com/) | Cat# 8180,  RRID: AB_10950972 |
| Mouse monoclonal anti-Rb, CloneName/4H1 | [Cell Signaling Technology](https://www.cellsignal.com/) | Cat# 9309,  RRID: AB_823629 |
| Mouse monoclonal anti-Lamin B1, CloneName/ 3C6 | Bioworld | Cat# MB8006, |
| Mouse monoclonal anti-Ubiquitin, CloneName/P4D1 | [Cell Signaling Technology](https://www.cellsignal.com/) | Cat# 3936,  RRID: AB_331292 |
| Rabbit polyclonal anti- K48-linkage specific polyubiquitin,  CloneName/D9D5 | [Cell Signaling Technology](https://www.cellsignal.com/) | Cat#12805,  RRID: AB_2798031 |
| Rabbit polyclonal anti- K63-linkage specific polyubiquitin,  CloneName/D7A11 | [Cell Signaling Technology](https://www.cellsignal.com/) | Cat#5621,  RRID: AB_10827985 |
| Mouse monoclonal anti-GAPDH, CloneName/2B8 | EASYBIO | Cat# BE0023, |
| Mouse monoclonal anti-β-actin, CloneName/6B8 | EASYBIO | Cat# BE0021 |
| Rabbit polyclonal anti-Cyclin A2,  CloneName/E1D9T | [Cell Signaling Technology](https://www.cellsignal.com/) | Cat# 91500 |
| Rabbit polyclonal anti-Cyclin B1,  CloneName/D5C10 | [Cell Signaling Technology](https://www.cellsignal.com/) | Cat# 12231,  RRID: AB_2783553 |
| Mouse monoclonal anti-Cyclin D3,  CloneName/DCS22 | [Cell Signaling Technology](https://www.cellsignal.com/) | Cat# 2936,  RRID: AB_2070801 |
| Mouse monoclonal anti-Cyclin D3 | Abcam | Cat# ab289546 |
| Mouse monoclonal anti-Cyclin E1,  CloneName/HE12 | [Cell Signaling Technology](https://www.cellsignal.com/) | Cat# 4129,  RRID: AB_2071200 |
| Goat anti-rabbit IgG (light chain) HPR conjugated | EASYBIO | Cat# BE0107,  Lot# 80910716 |
| Goat anti-mouse IgG (light chain) HPR conjugated | EASYBIO | Cat# BE0105,  Lot# 80910911 |
| Goat anti-rabbit IgG (H+L) HPR conjugated | EASYBIO | Cat# BE0101,  Lot# 8091220 |
| Goat anti-mouse IgG (H+L) HPR conjugated | EASYBIO | Cat# BE0102,  Lot# 80910708 |
| Goat anti-mouse IgG FITC conjugated | ZSGB-BIO. | Cat# ZF-0312,  Lot# 139415 |
| Goat anti-rabbit IgG TRITC conjugated | ZSGB-BIO. | Cat# ZF-0316,  Lot# 138669 |
| Control rabbit IgG | Santa Cruz Biotechnology | Cat# sc-2027,  RRID: AB_737197 |
| Rabbit monoclonal anti-MG53 | Wu, Hong-Kun, et al. (2019) | N/A |
