# Supplementary material for: E3 ligase MG53 suppresses tumor growth by degrading cyclin D1
Source: Signal Transduct Target Ther. 2023 Jul 7;8:263. doi: 10.1038/s41392-023-01458-9 (PMC10326024; doi:10.1038/s41392-023-01458-9)
Supplement: Supplementary file 2 — Supplementary Table S1 [file 41392_2023_1458_MOESM2_ESM.pdf]

HCT116

Accession	Description	Score	Coverage	# Proteins	# Unique Peptides	# Peptides	# PSMs	Area	# AAs	MW [kDa]	calc. pI
Q6ZMU5	Tripartite motif-containing protein 72 OS=Homo sapiens GN=TRIM7	1311.83	79.87	1	47	47	463	1.066E11	477	52.7	6.48
O15372	Eukaryotic translation initiation factor 3 subunit H OS=Homo sapiens	320.03	75.28	9	29	29	114	2.975E9	352	39.9	6.54
P22626	Heterogeneous nuclear ribonucleoproteins A2/B1 OS=Homo sapiens	312.62	74.50	2	29	34	113	4.467E9	353	37.4	8.95
P05388	60S acidic ribosomal protein P0 OS=Homo sapiens GN=RPLP0 PE=1	283.60	67.19	13	23	23	92	1.443E10	317	34.3	5.97
F8W6I7	Heterogeneous nuclear ribonucleoprotein A1 OS=Homo sapiens GN=	282.47	61.89	8	23	28	93	3.844E9	307	33.1	9.13
Q13347	Eukaryotic translation initiation factor 3 subunit I OS=Homo sapiens	237.13	86.15	3	24	24	77	3.155E9	325	36.5	5.64
Q02878	60S ribosomal protein L6 OS=Homo sapiens GN=RPL6 PE=1 SV=3 -	226.92	64.58	7	31	31	75	7.362E9	288	32.7	10.58
Q7L2H7	Eukaryotic translation initiation factor 3 subunit M OS=Homo sapiens	205.85	68.98	7	25	25	72	2.227E9	374	42.5	5.63
P04264	Keratin, type II cytoskeletal 1 OS=Homo sapiens GN=KRT1 PE=1 SV	172.77	55.90	16	32	38	60	1.410E9	644	66.0	8.12
E7EU96	Casein kinase II subunit alpha OS=Homo sapiens GN=CSNK2A1 PE=	146.57	59.74	9	20	22	52	1.206E9	385	45.3	7.94
Q96AG4	Leucine-rich repeat-containing protein 59 OS=Homo sapiens GN=LR	142.82	57.65	2	21	21	48	2.271E9	307	34.9	9.57
P05198	Eukaryotic translation initiation factor 2 subunit 1 OS=Homo sapiens	140.46	73.02	3	31	31	48	9.461E8	315	36.1	5.08
P46777	60S ribosomal protein L5 OS=Homo sapiens GN=RPL5 PE=1 SV=3 -	129.39	65.66	3	25	25	51	1.467E9	297	34.3	9.72
P51991	Heterogeneous nuclear ribonucleoprotein A3 OS=Homo sapiens GN=	128.87	43.39	2	20	22	48	1.083E9	378	39.6	9.01
P19784	Casein kinase II subunit alpha' OS=Homo sapiens GN=CSNK2A2 PE=	125.67	63.14	6	20	22	41	7.169E8	350	41.2	8.56
P07355	Annexin A2 OS=Homo sapiens GN=ANXA2 PE=1 SV=2 - [ANXA2_HU	122.48	59.59	24	24	24	43	6.103E8	339	38.6	7.75
P04406	Glyceraldehyde-3-phosphate dehydrogenase OS=Homo sapiens GN=	121.87	79.70	2	21	21	37	1.910E9	335	36.0	8.46
Q02218	2-oxoglutarate dehydrogenase, mitochondrial OS=Homo sapiens GN	120.45	30.30	6	18	26	39	7.846E8	1023	115.9	6.86
P62136	Serine/threonine-protein phosphatase PP1-alpha catalytic subunit OS	112.02	63.33	7	5	22	40	7.348E8	330	37.5	6.33
P36957	Dihydrolipoyllysine-residue succinyltransferase component of 2-oxog	110.62	47.46	5	18	18	38	9.883E8	453	48.7	8.95
Q99988	Growth/differentiation factor 15 OS=Homo sapiens GN=GDF15 PE=1	104.94	60.06	2	15	15	39	3.380E9	308	34.1	9.66
Q15366	Poly(rC)-binding protein 2 OS=Homo sapiens GN=PCBP2 PE=1 SV=1	102.22	56.71	21	1	13	33	1.616E9	365	38.6	6.79
H3BRU6	Poly(rC)-binding protein 2 (Fragment) OS=Homo sapiens GN=PCBP2	101.96	69.10	21	1	13	33	1.616E9	301	31.7	8.44
P06748	Nucleophosmin OS=Homo sapiens GN=NPM1 PE=1 SV=2 - [NPM_H	101.28	51.02	8	16	16	34	1.473E9	294	32.6	4.78
P35527	Keratin, type I cytoskeletal 9 OS=Homo sapiens GN=KRT9 PE=1 SV=	100.26	54.90	2	23	23	37	8.325E8	623	62.0	5.24
P51665	26S proteasome non-ATPase regulatory subunit 7 OS=Homo sapiens	100.01	54.01	4	15	15	31	6.577E8	324	37.0	6.77
P13645	Keratin, type I cytoskeletal 10 OS=Homo sapiens GN=KRT10 PE=1 S	94.75	45.03	5	25	26	34	8.602E8	584	58.8	5.21
P35908	Keratin, type II cytoskeletal 2 epidermal OS=Homo sapiens GN=KRT	93.26	54.30	12	16	25	32	9.245E8	639	65.4	8.00
Q15717	ELAV-like protein 1 OS=Homo sapiens GN=ELAVL1 PE=1 SV=2 - [EL	91.86	57.67	10	15	15	28	7.791E8	326	36.1	9.17
F8VYE8	Serine/threonine-protein phosphatase OS=Homo sapiens GN=PPP1C	89.91	55.59	8	1	18	32	5.633E8	304	34.9	5.26
Q07021	Complement component 1 Q subcomponent-binding protein, mitoch	89.40	63.12	3	11	11	31	2.064E9	282	31.3	4.84
Q14C86	GTPase-activating protein and VPS9 domain-containing protein 1 OS	88.91	12.86	6	22	22	31	6.676E8	1478	164.9	5.22
D6R9P3	Heterogeneous nuclear ribonucleoprotein A/B OS=Homo sapiens GN	87.58	42.86	4	16	17	33	1.245E9	280	30.3	7.91
P62995	Transformer-2 protein homolog beta OS=Homo sapiens GN=TRA2B	81.76	45.49	3	13	14	29	1.588E9	288	33.6	11.25
P61962	DDB1- and CUL4-associated factor 7 OS=Homo sapiens GN=DCAF7	79.38	48.83	2	14	14	29	1.290E9	342	38.9	5.52
O15479	Melanoma-associated antigen B2 OS=Homo sapiens GN=MAGEB2 PE	76.19	50.78	5	19	19	32	6.804E8	319	35.3	8.76
P62140	Serine/threonine-protein phosphatase PP1-beta catalytic subunit OS=	74.56	55.35	6	5	18	28	5.304E8	327	37.2	6.19
Q9Y3F4	Serine-threonine kinase receptor-associated protein OS=Homo sapie	72.32	59.14	2	18	18	27	4.597E8	350	38.4	5.12
P22087	rRNA 2'-O-methyltransferase fibrillarin OS=Homo sapiens GN=FBL PI	71.07	60.12	10	14	14	22	3.672E8	321	33.8	10.18
P61247	40S ribosomal protein S3a OS=Homo sapiens GN=RPS3A PE=1 SV=	70.38	60.23	12	19	19	30	5.139E8	264	29.9	9.73
A0A087WUK2	Heterogeneous nuclear ribonucleoprotein D-like OS=Homo sapiens G	68.84	30.30	2	12	14	28	8.556E8	363	40.0	9.96
Q9BQE3	Tubulin alpha-1C chain OS=Homo sapiens GN=TUBA1C PE=1 SV=1	68.82	36.75	23	1	12	25	3.372E8	449	49.9	5.10
Q12796	Proline-rich nuclear receptor coactivator 1 OS=Homo sapiens GN=PN	67.77	65.14	2	16	16	26	5.180E8	327	35.2	10.51
G3V2Q1	Heterogeneous nuclear ribonucleoproteins C1/C2 OS=Homo sapiens	66.49	44.59	19	8	14	26	1.529E9	305	33.6	5.08
P68104	Elongation factor 1-alpha 1 OS=Homo sapiens GN=EEF1A1 PE=1 SV	65.93	26.84	7	12	12	21	1.047E9	462	50.1	9.01
O43684	Mitotic checkpoint protein BUB3 OS=Homo sapiens GN=BUB3 PE=1	65.60	65.24	3	17	17	25	7.226E8	328	37.1	6.84
Q9Y295	Developmentally-regulated GTP-binding protein 1 OS=Homo sapiens	65.36	64.31	7	19	19	25	4.678E8	367	40.5	8.90
F8VZJ2	Nascent polypeptide-associated complex subunit alpha OS=Homo sa	64.11	52.94	9	7	7	18	2.500E9	136	15.0	4.91
P48729	Casein kinase I isoform alpha OS=Homo sapiens GN=CSNK1A1 PE=	62.55	47.48	8	5	16	25	4.568E8	337	38.9	9.57
K7ES61	39S ribosomal protein L4, mitochondrial (Fragment) OS=Homo sapie	61.82	53.00	7	11	11	20	3.672E8	300	33.8	9.83

Q99496	E3 ubiquitin-protein ligase RING2 OS=Homo sapiens GN=RNF2 PE=	61.67	33.33	3	10	10	19	5.514E8	336	37.6	6.84
P08779	Keratin, type I cytoskeletal 16 OS=Homo sapiens GN=KRT16 PE=1 S	61.43	40.59	8	13	18	23	2.763E8	473	51.2	5.05
J3KPX7	Prohibitin-2 OS=Homo sapiens GN=PHB2 PE=1 SV=2 - [J3KPX7_HU	61.37	66.44	8	17	17	26	5.442E8	298	33.2	9.83
P50750	Cyclin-dependent kinase 9 OS=Homo sapiens GN=CDK9 PE=1 SV=3	60.97	38.44	67	15	16	23	4.554E8	372	42.8	8.79
P46736	Lys-63-specific deubiquitinase BRCC36 OS=Homo sapiens GN=BRCC	58.83	59.49	6	16	16	23	4.336E8	316	36.0	5.92
Q9NYK5	39S ribosomal protein L39, mitochondrial OS=Homo sapiens GN=MR	58.58	51.48	2	17	17	24	3.788E8	338	38.7	7.65
Q13151	Heterogeneous nuclear ribonucleoprotein A0 OS=Homo sapiens GN=	58.23	40.66	1	11	13	23	6.926E8	305	30.8	9.29
Q71U36	Tubulin alpha-1A chain OS=Homo sapiens GN=TUBA1A PE=1 SV=1	58.03	36.59	18	1	12	22	3.372E8	451	50.1	5.06
P11940	Polyadenylate-binding protein 1 OS=Homo sapiens GN=PABPC1 PE=	57.82	39.62	23	12	19	21	2.041E8	636	70.6	9.50
Q15365	Poly(rC)-binding protein 1 OS=Homo sapiens GN=PCBP1 PE=1 SV=2	57.08	48.03	13	8	12	19	7.934E8	356	37.5	7.09
O60506	Heterogeneous nuclear ribonucleoprotein Q OS=Homo sapiens GN=S	56.55	28.09	3	13	15	19	1.901E8	623	69.6	8.59
Q9H9J2	39S ribosomal protein L44, mitochondrial OS=Homo sapiens GN=MR	55.96	39.16	1	14	14	22	3.256E8	332	37.5	8.40
G5E9W7	28S ribosomal protein S22, mitochondrial OS=Homo sapiens GN=MR	55.70	52.04	7	14	14	22	2.481E8	319	36.8	6.81
E9PEX6	Dihydrolipoyl dehydrogenase OS=Homo sapiens GN=DLD PE=1 SV=	55.12	38.89	2	15	15	22	2.747E8	486	51.8	7.96
P04259	Keratin, type II cytoskeletal 6B OS=Homo sapiens GN=KRT6B PE=1	54.91	33.87	12	1	20	24	5.546E8	564	60.0	8.00
B3KPJ4	Polyhomeotic-like protein 2 OS=Homo sapiens GN=PHC2 PE=1 SV=	54.89	29.74	5	14	14	20	3.644E8	464	50.4	8.05
Q5JP53	Tubulin beta chain OS=Homo sapiens GN=TUBB PE=1 SV=1 - [Q5JP	53.76	38.73	18	3	14	19	2.200E8	426	47.7	4.81
P48668	Keratin, type II cytoskeletal 6C OS=Homo sapiens GN=KRT6C PE=1	53.73	35.46	15	2	21	23	3.152E8	564	60.0	8.00
P52907	F-actin-capping protein subunit alpha-1 OS=Homo sapiens GN=CAPZ	53.38	63.99	1	8	11	19	8.457E8	286	32.9	5.69
H0YA96	Heterogeneous nuclear ribonucleoprotein D0 (Fragment) OS=Homo	53.01	47.14	6	5	12	23	9.718E8	210	23.9	9.58
Q96DV4	39S ribosomal protein L38, mitochondrial OS=Homo sapiens GN=MR	51.38	39.47	1	13	13	22	2.903E8	380	44.6	7.53
B3KTM8	Mortality factor 4-like protein 1 OS=Homo sapiens GN=MORF4L1 PE	50.29	55.17	10	13	15	20	1.790E8	348	40.0	9.11
P51398	28S ribosomal protein S29, mitochondrial OS=Homo sapiens GN=DA	49.84	37.44	11	15	15	21	2.692E8	398	45.5	8.88
P46976	Glycogenin-1 OS=Homo sapiens GN=GYG1 PE=1 SV=4 - [GLYG_HU	49.24	32.29	5	13	13	22	7.734E8	350	39.4	5.53
P68371	Tubulin beta-4B chain OS=Homo sapiens GN=TUBB4B PE=1 SV=1 -	48.62	43.15	21	4	15	20	1.827E8	445	49.8	4.89
O75822	Eukaryotic translation initiation factor 3 subunit J OS=Homo sapiens	47.15	41.86	3	14	14	19	1.613E9	258	29.0	4.83
O00487	26S proteasome non-ATPase regulatory subunit 14 OS=Homo sapier	46.84	50.32	2	11	11	16	5.483E8	310	34.6	6.52
P13647	Keratin, type II cytoskeletal 5 OS=Homo sapiens GN=KRT5 PE=1 SV	46.47	27.97	16	6	19	21	2.755E8	590	62.3	7.74
P35249	Replication factor C subunit 4 OS=Homo sapiens GN=RFC4 PE=1 SV	45.85	50.96	9	15	15	20	2.641E8	363	39.7	8.02
F5H265	Polyubiquitin-C (Fragment) OS=Homo sapiens GN=UBC PE=1 SV=1	45.62	63.09	23	6	6	22	2.993E9	149	16.8	6.58
P82933	28S ribosomal protein S9, mitochondrial OS=Homo sapiens GN=MRF	45.50	42.93	1	15	15	19	2.063E8	396	45.8	9.51
P13995	Bifunctional methylenetetrahydrofolate dehydrogenase/cyclohydrolas	44.81	57.71	3	12	12	17	3.227E8	350	37.9	8.73
Q9UNM6	26S proteasome non-ATPase regulatory subunit 13 OS=Homo sapier	44.10	42.82	7	14	14	18	1.970E8	376	42.9	5.81
A0A0G2JLR5	Ras association domain-containing protein 7 OS=Homo sapiens GN=	43.52	48.66	7	12	12	15	3.382E8	337	36.3	6.06
P40937	Replication factor C subunit 5 OS=Homo sapiens GN=RFC5 PE=1 SV	43.08	55.00	6	13	13	18	2.520E8	340	38.5	7.20
Q13243	Serine/arginine-rich splicing factor 5 OS=Homo sapiens GN=SRSF5 F	42.22	29.78	4	9	10	15	9.285E8	272	31.2	11.59
P60709	Actin, cytoplasmic 1 OS=Homo sapiens GN=ACTB PE=1 SV=1 - [AC1	42.03	41.33	28	11	11	16	3.252E8	375	41.7	5.48
P09001	39S ribosomal protein L3, mitochondrial OS=Homo sapiens GN=MRF	41.48	37.64	6	10	10	15	2.216E8	348	38.6	9.48
D6REM4	Casein kinase I isoform alpha OS=Homo sapiens GN=CSNK1A1 PE=	41.42	54.66	2	1	12	18	4.090E8	236	27.7	9.44
Q12904	Aminoacyl tRNA synthase complex-interacting multifunctional protein	41.35	43.91	2	9	9	13	2.417E8	312	34.3	8.43
Q13283	Ras GTPase-activating protein-binding protein 1 OS=Homo sapiens G	40.63	30.04	1	11	11	19	2.491E8	466	52.1	5.52
Q5JRI1	Serine/arginine-rich-splicing factor 10 OS=Homo sapiens GN=SRSF1	40.49	44.77	5	10	10	14	2.937E8	172	20.9	10.48
O43464	Serine protease HTRA2, mitochondrial OS=Homo sapiens GN=HTRA	38.91	34.93	3	10	10	13	2.039E8	458	48.8	10.07
C9J9K3	40S ribosomal protein SA (Fragment) OS=Homo sapiens GN=RPSA F	38.05	44.87	4	10	10	13	5.064E8	263	29.4	5.25
Q9UNQ2	Probable dimethyladenosine transferase OS=Homo sapiens GN=DIM	37.71	45.37	3	12	12	15	2.952E8	313	35.2	9.99
P47755	F-actin-capping protein subunit alpha-2 OS=Homo sapiens GN=CAPZ	37.63	45.45	4	6	9	14	4.603E8	286	32.9	5.85
Q13247	Serine/arginine-rich splicing factor 6 OS=Homo sapiens GN=SRSF6 F	37.11	25.87	4	8	9	14	6.309E8	344	39.6	11.43
B1ANR0	Polyadenylate-binding protein OS=Homo sapiens GN=PABPC4 PE=1	36.40	32.20	9	7	14	16	1.045E8	615	67.9	9.45
P08727	Keratin, type I cytoskeletal 19 OS=Homo sapiens GN=KRT19 PE=1 S	36.29	34.00	6	8	12	13	2.431E8	400	44.1	5.14
P54105	Methylosome subunit pICln OS=Homo sapiens GN=CLNS1A PE=1 SV	36.22	59.49	5	7	7	14	3.147E8	237	26.2	4.11
A2A2S5	Ribosome-binding protein 1 (Fragment) OS=Homo sapiens GN=RRB	36.04	39.00	4	8	8	13	3.601E8	300	31.7	9.89
Q8WVM0	Dimethyladenosine transferase 1, mitochondrial OS=Homo sapiens G	35.01	54.34	1	14	14	18	1.791E8	346	39.5	9.26
P67809	Nuclease-sensitive element-binding protein 1 OS=Homo sapiens GN=	35.00	41.36	4	4	8	14	3.268E8	324	35.9	9.88

Q9NPH2	Inositol-3-phosphate synthase 1 OS=Homo sapiens GN=ISYNA1 PE=	34.06	21.68	3	9	9	13	3.023E8	558	61.0	5.76
Q9Y2S7	Polymerase delta-interacting protein 2 OS=Homo sapiens GN=POLD	33.74	47.01	2	13	13	15	9.269E7	368	42.0	8.63
P0DMV8	Heat shock 70 kDa protein 1A OS=Homo sapiens GN=HSPA1A PE=1	33.72	21.53	6	8	10	14	1.340E8	641	70.0	5.66
P07951	Tropomyosin beta chain OS=Homo sapiens GN=TPM2 PE=1 SV=1 -	33.67	30.28	22	6	11	13	1.261E8	284	32.8	4.70
P61964	WD repeat-containing protein 5 OS=Homo sapiens GN=WDR5 PE=1	33.52	44.31	4	11	11	13	2.087E8	334	36.6	8.27
P62714	Serine/threonine-protein phosphatase 2A catalytic subunit beta isofo	33.51	33.98	8	7	9	11	2.983E8	309	35.6	5.43
D6RBQ9	Heterogeneous nuclear ribonucleoprotein D0 (Fragment) OS=Homo	33.44	37.42	2	1	8	17	6.896E8	155	15.6	4.91
P02533	Keratin, type I cytoskeletal 14 OS=Homo sapiens GN=KRT14 PE=1 S	33.37	34.75	7	7	14	17	2.763E8	472	51.5	5.16
Q13595	Transformer-2 protein homolog alpha OS=Homo sapiens GN=TRA2A	33.18	32.27	1	10	11	15	6.738E8	282	32.7	11.27
Q15014	Mortality factor 4-like protein 2 OS=Homo sapiens GN=MORF4L2 PE	32.87	48.26	5	9	11	12	1.209E8	288	32.3	9.72
P82673	28S ribosomal protein S35, mitochondrial OS=Homo sapiens GN=MF	32.60	40.25	2	10	10	14	1.299E8	323	36.8	8.24
F8VRH0	Poly(rC)-binding protein 2 (Fragment) OS=Homo sapiens GN=PCBP2	32.30	33.55	1	1	5	9	5.430E8	310	32.0	8.07
Q7L5D6	Golgi to ER traffic protein 4 homolog OS=Homo sapiens GN=GET4 P	32.19	32.42	4	9	9	16	3.231E8	327	36.5	5.41
Q96QR8	Transcriptional activator protein Pur-beta OS=Homo sapiens GN=PU	31.85	40.06	4	11	12	16	3.345E8	312	33.2	5.43
Q9UH17	DNA dC->dU-editing enzyme APOBEC-3B OS=Homo sapiens GN=AP	31.38	35.34	5	9	11	15	1.482E8	382	45.9	6.06
A0A0G2JPF8	Uncharacterized protein OS=Homo sapiens PE=4 SV=1 - [A0A0G2JP	30.51	18.09	6	1	7	14	1.089E9	293	32.0	5.68
P17535	Transcription factor jun-D OS=Homo sapiens GN=JUND PE=1 SV=3	30.22	29.39	2	6	6	11	3.005E8	347	35.2	7.37
Q9UN81	LINE-1 retrotransposable element ORF1 protein OS=Homo sapiens G	29.92	30.47	1	10	10	11	1.866E8	338	40.0	9.51
B4DHE8	RNA-binding protein Musashi homolog 2 OS=Homo sapiens GN=MSI	29.82	28.40	6	8	9	10	2.964E8	324	34.8	8.88
P11142	Heat shock cognate 71 kDa protein OS=Homo sapiens GN=HSPA8 P	29.76	24.15	14	10	12	12	7.307E7	646	70.9	5.52
O96008	Mitochondrial import receptor subunit TOM40 homolog OS=Homo sa	29.66	34.63	3	8	8	11	1.309E8	361	37.9	7.25
P40926	Malate dehydrogenase, mitochondrial OS=Homo sapiens GN=MDH2	29.42	32.54	2	8	8	9	1.353E8	338	35.5	8.68
P16989	Y-box-binding protein 3 OS=Homo sapiens GN=YBX3 PE=1 SV=4 -	28.99	13.98	3	1	5	10	2.090E8	372	40.1	9.77
A6NLN1	Polypyrimidine tract binding protein 1, isoform CRA_b OS=Homo sap	28.48	20.87	11	9	9	12	1.852E8	527	56.5	9.38
Q9ULD0	2-oxoglutarate dehydrogenase-like, mitochondrial OS=Homo sapiens	28.23	7.62	1	1	9	11	3.168E8	1010	114.4	6.65
Q99613	Eukaryotic translation initiation factor 3 subunit C OS=Homo sapiens	28.16	9.97	5	9	9	11	1.553E8	913	105.3	5.68
Q07820	Induced myeloid leukemia cell differentiation protein Mcl-1 OS=Hom	27.97	23.71	3	8	8	10	2.341E8	350	37.3	5.66
Q3ZCQ8	Mitochondrial import inner membrane translocase subunit TIM50 OS	27.55	29.75	10	10	10	11	1.898E8	353	39.6	8.37
Q9BWM7	Sideroflexin-3 OS=Homo sapiens GN=SFXN3 PE=1 SV=3 - [SFXN3_	27.51	34.89	3	8	8	11	7.616E7	321	35.5	9.10
Q00577	Transcriptional activator protein Pur-alpha OS=Homo sapiens GN=PU	27.17	32.61	3	6	7	11	6.123E8	322	34.9	6.44
Q9BYG3	MKI67 FHA domain-interacting nucleolar phosphoprotein OS=Homo	27.01	43.00	4	10	10	11	1.384E8	293	34.2	9.88
P35250	Replication factor C subunit 2 OS=Homo sapiens GN=RFC2 PE=1 SV	26.83	39.55	10	10	10	10	1.774E8	354	39.1	6.44
P07195	L-lactate dehydrogenase B chain OS=Homo sapiens GN=LDHB PE=1	26.14	36.83	11	9	10	11	1.789E8	334	36.6	6.05
Q9BWF3	RNA-binding protein 4 OS=Homo sapiens GN=RBM4 PE=1 SV=1 - [	26.03	36.26	8	9	9	10	2.493E8	364	40.3	7.08
Q9Y262	Eukaryotic translation initiation factor 3 subunit L OS=Homo sapiens	26.00	22.87	7	12	12	12	1.752E8	564	66.7	6.34
Q9GZY8	Mitochondrial fission factor OS=Homo sapiens GN=MFF PE=1 SV=1	25.76	52.05	7	8	8	10	1.141E8	342	38.4	8.95
P05787	Keratin, type II cytoskeletal 8 OS=Homo sapiens GN=KRT8 PE=1 SV	25.51	19.05	24	5	11	12	2.728E8	483	53.7	5.59
Q96B36	Proline-rich AKT1 substrate 1 OS=Homo sapiens GN=AKT1S1 PE=1	25.42	33.98	2	6	6	9	7.079E8	256	27.4	4.75
Q6P2E9	Enhancer of mRNA-decapping protein 4 OS=Homo sapiens GN=EDC	25.30	11.71	2	11	11	12	7.301E7	1401	151.6	5.86
Q9P015	39S ribosomal protein L15, mitochondrial OS=Homo sapiens GN=MF	25.21	44.59	3	10	10	11	8.960E7	296	33.4	10.01
P28562	Dual specificity protein phosphatase 1 OS=Homo sapiens GN=DUSP	25.16	18.80	1	5	5	8	1.087E8	367	39.3	7.20
O14908	PDZ domain-containing protein GIPC1 OS=Homo sapiens GN=GIPC1	24.88	30.63	6	7	7	10	4.627E7	333	36.0	6.28
P82675	28S ribosomal protein S5, mitochondrial OS=Homo sapiens GN=MRI	24.84	18.84	1	7	7	9	1.114E8	430	48.0	9.92
P0DN76	Splicing factor U2AF 35 kDa subunit-like protein OS=Homo sapiens G	24.77	33.33	5	6	6	7	4.996E8	240	27.9	8.81
A0A140T933	HLA class I histocompatibility antigen, A-3 alpha chain (Fragment) O	24.70	36.79	39	3	8	8	2.651E8	299	34.2	6.71
P12004	Proliferating cell nuclear antigen OS=Homo sapiens GN=PCNA PE=1	24.49	52.11	1	9	9	10	3.293E8	261	28.8	4.69
E7ER27	Peroxisomal multifunctional enzyme type 2 OS=Homo sapiens GN=H	24.14	19.00	5	9	9	10	8.734E7	500	53.9	6.33
Q16795	NADH dehydrogenase [ubiquinone] 1 alpha subcomplex subunit 9, m	24.07	35.54	4	10	10	12	7.571E7	377	42.5	9.80
Q96C36	Pyrroline-5-carboxylate reductase 2 OS=Homo sapiens GN=PYCR2 P	24.05	30.94	7	7	8	10	1.615E8	320	33.6	7.77
K7EIJ0	WW domain-binding protein 2 (Fragment) OS=Homo sapiens GN=W	23.95	43.71	9	7	7	8	2.448E8	167	18.2	7.90
E7EMC7	Sequestosome-1 OS=Homo sapiens GN=SQSTM1 PE=1 SV=1 - [E7E	23.73	26.46	7	5	5	8	1.180E8	378	41.0	7.52
O43837	Isocitrate dehydrogenase [NAD] subunit beta, mitochondrial OS=Ho	23.69	20.00	4	6	6	8	7.211E7	385	42.2	8.46
P61978	Heterogeneous nuclear ribonucleoprotein K OS=Homo sapiens GN=H	23.59	20.52	2	6	6	8	6.475E7	463	50.9	5.54

P35226	Polycomb complex protein BMI-1 OS=Homo sapiens GN=BMI1 PE=1	23.59	25.15	8	8	8	10	1.486E8	326	36.9	8.63
Q92665	28S ribosomal protein S31, mitochondrial OS=Homo sapiens GN=MR	23.56	31.90	1	13	13	14	1.404E8	395	45.3	9.29
P28482	Mitogen-activated protein kinase 1 OS=Homo sapiens GN=MAPK1 P	23.02	33.89	7	11	11	13	1.396E8	360	41.4	6.98
O75367	Core histone macro-H2A.1 OS=Homo sapiens GN=H2AFY PE=1 SV=	22.92	28.76	3	7	7	8	9.094E7	372	39.6	9.79
Q9BYD6	39S ribosomal protein L1, mitochondrial OS=Homo sapiens GN=MR	22.90	28.00	2	8	8	9	1.099E8	325	36.9	8.78
Q04695	Keratin, type I cytoskeletal 17 OS=Homo sapiens GN=KRT17 PE=1 S	22.88	20.37	9	3	9	10	2.314E8	432	48.1	5.02
P04075	Fructose-bisphosphate aldolase A OS=Homo sapiens GN=ALDOA PE	22.54	33.24	9	10	10	11	1.452E8	364	39.4	8.09
F5H6M0	Cleavage and polyadenylation-specificity factor subunit 7 (Fragment)	22.35	40.64	14	8	8	9	1.628E8	219	24.4	5.22
Q96EY1	DnaJ homolog subfamily A member 3, mitochondrial OS=Homo sapi	22.27	21.04	2	7	7	10	1.712E8	480	52.5	9.26
P25685	DnaJ homolog subfamily B member 1 OS=Homo sapiens GN=DNAJB	21.97	28.53	8	8	9	12	2.405E8	340	38.0	8.63
P35637	RNA-binding protein FUS OS=Homo sapiens GN=FUS PE=1 SV=1 -	21.81	15.40	4	6	6	9	1.626E8	526	53.4	9.36
Q96DI7	U5 small nuclear ribonucleoprotein 40 kDa protein OS=Homo sapien	21.76	36.69	2	8	8	10	6.416E7	357	39.3	8.10
Q5QPL9	RNA-binding protein Raly (Fragment) OS=Homo sapiens GN=RALY P	21.31	43.04	9	9	9	9	1.673E8	237	24.7	10.49
Q99848	Probable rRNA-processing protein EBP2 OS=Homo sapiens GN=EBN	21.21	28.76	2	6	6	9	9.195E7	306	34.8	10.10
P46734	Dual specificity mitogen-activated protein kinase kinase 3 OS=Homo	20.85	33.72	5	9	9	9	1.634E8	347	39.3	7.43
Q9UBS4	DnaJ homolog subfamily B member 11 OS=Homo sapiens GN=DNAJ	20.69	19.27	2	6	6	7	1.411E8	358	40.5	6.18
A0A1W2PRV5	Survival motor neuron protein OS=Homo sapiens GN=SMN2 PE=1 S	20.61	24.82	8	6	6	10	3.073E8	282	30.4	5.73
A0A087WWU8	Tropomyosin alpha-3 chain OS=Homo sapiens GN=TPM3 PE=1 SV=	20.54	17.18	13	1	6	8	5.796E7	227	26.4	4.78
P23396	40S ribosomal protein S3 OS=Homo sapiens GN=RPS3 PE=1 SV=2 -	20.15	53.50	12	11	11	11	1.600E8	243	26.7	9.66
J3KPS0	DnaJ (Hsp40) homolog, subfamily B, member 12, isoform CRA_c OS	20.11	22.25	4	8	9	9	8.831E7	409	45.5	9.36
P26651	mRNA decay activator protein ZFP36 OS=Homo sapiens GN=ZFP36	20.09	19.33	4	5	5	7	3.137E8	326	34.0	8.48
O00743	Serine/threonine-protein phosphatase 6 catalytic subunit OS=Homo	20.06	23.93	2	5	5	6	1.140E8	305	35.1	5.69
Q92890	Ubiquitin recognition factor in ER-associated degradation protein 1 O	19.82	30.29	4	8	8	8	1.403E8	307	34.5	6.70
Q96L58	Beta-1,3-galactosyltransferase 6 OS=Homo sapiens GN=B3GALT6 PE	19.49	13.68	1	4	4	6	9.317E7	329	37.1	9.66
O75821	Eukaryotic translation initiation factor 3 subunit G OS=Homo sapiens	19.31	19.06	5	7	7	8	1.058E8	320	35.6	6.13
Q9H2G9	Golgin-45 OS=Homo sapiens GN=BLZF1 PE=1 SV=2 - [GO45_HUMA	18.98	21.75	3	7	7	8	5.176E7	400	44.9	8.54
P29966	Myristoylated alanine-rich C-kinase substrate OS=Homo sapiens GN=	18.70	15.66	1	6	6	9	8.570E8	332	31.5	4.45
P36551	Oxygen-dependent coproporphyrinogen-III oxidase, mitochondrial O	18.34	19.60	3	7	7	8	1.243E8	454	50.1	8.25
Q969G5	Caveolae-associated protein 3 OS=Homo sapiens GN=CAVIN3 PE=1	18.33	27.20	2	6	6	8	6.444E7	261	27.7	6.43
P49411	Elongation factor Tu, mitochondrial OS=Homo sapiens GN=TUFM PE	18.32	18.58	1	7	7	8	9.259E7	452	49.5	7.61
Q9UL40	Zinc finger protein 346 OS=Homo sapiens GN=ZNF346 PE=1 SV=1 -	18.24	17.35	6	4	4	6	1.302E8	294	32.9	9.09
Q9NXS2	Glutaminyl-peptide cyclotransferase-like protein OS=Homo sapiens G	18.05	27.49	2	9	9	10	1.255E8	382	42.9	9.82
G3XAN4	Translocating chain-associated membrane protein 1 OS=Homo sapie	18.01	19.79	2	6	6	7	7.681E7	288	33.4	9.89
G3V2D5	mRNA decay activator protein ZFP36L1 (Fragment) OS=Homo sapien	17.79	32.95	5	6	6	7	8.560E7	176	19.5	9.66
P50402	Emerin OS=Homo sapiens GN=EMD PE=1 SV=1 - [EMD_HUMAN]	17.70	29.92	2	6	6	7	1.081E8	254	29.0	5.50
P78345	Ribonuclease P protein subunit p38 OS=Homo sapiens GN=RPP38 P	17.61	28.62	3	7	7	8	6.739E7	283	31.8	9.92
A0A087WX29	TAR DNA-binding protein 43 (Fragment) OS=Homo sapiens GN=TAR	17.43	30.86	20	5	5	6	5.242E7	243	26.7	7.77
Q9NZJ7	Mitochondrial carrier homolog 1 OS=Homo sapiens GN=MTCH1 PE=	17.19	21.08	3	7	7	8	1.183E8	389	41.5	9.32
P40938	Replication factor C subunit 3 OS=Homo sapiens GN=RFC3 PE=1 SV	17.18	23.88	2	7	7	7	1.873E8	356	40.5	8.34
A0A0C4DFV9	Protein SET OS=Homo sapiens GN=SET PE=1 SV=1 - [A0A0C4DFV9	17.07	33.08	4	6	6	6	7.114E7	266	31.1	4.23
O75477	Erlin-1 OS=Homo sapiens GN=ERLIN1 PE=1 SV=1 - [ERLN1_HUMAN	17.03	18.79	5	6	6	7	1.149E8	346	38.9	7.87
O43255	E3 ubiquitin-protein ligase SIAH2 OS=Homo sapiens GN=SIAH2 PE=	16.84	13.27	3	7	7	7	2.838E8	324	34.6	7.12
Q8TDN6	Ribosome biogenesis protein BRX1 homolog OS=Homo sapiens GN=	16.67	18.98	1	6	6	8	4.033E7	353	41.4	9.92
P19387	DNA-directed RNA polymerase II subunit RPB3 OS=Homo sapiens G	16.66	34.18	2	8	8	10	9.856E7	275	31.4	4.92
Q9NZL4	Hsp70-binding protein 1 OS=Homo sapiens GN=HSPBP1 PE=1 SV=1	16.54	15.47	4	5	5	6	6.955E7	362	39.4	5.21
Q9Y617	Phosphoserine aminotransferase OS=Homo sapiens GN=PSAT1 PE=	16.43	19.19	1	7	7	7	1.026E8	370	40.4	7.66
P15880	40S ribosomal protein S2 OS=Homo sapiens GN=RPS2 PE=1 SV=2 -	16.39	25.60	9	6	6	7	8.008E7	293	31.3	10.24
P60842	Eukaryotic initiation factor 4A-I OS=Homo sapiens GN=EIF4A1 PE=1	16.33	15.27	22	6	6	6	7.769E7	406	46.1	5.48
E9PK01	Elongation factor 1-delta (Fragment) OS=Homo sapiens GN=EEF1D	16.25	46.36	22	9	9	10	1.380E8	261	28.8	5.02
Q9UG56	Phosphatidylserine decarboxylase proenzyme, mitochondrial OS=Ho	16.18	20.05	4	7	7	8	4.854E7	409	46.6	9.42
Q9NQ29	Putative RNA-binding protein Luc7-like 1 OS=Homo sapiens GN=LU	15.87	21.56	7	3	8	8	5.247E7	371	43.7	9.92
P43307	Translocon-associated protein subunit alpha OS=Homo sapiens GN=	15.86	20.98	6	4	4	6	5.217E8	286	32.2	4.49
Q92820	Gamma-glutamyl hydrolase OS=Homo sapiens GN=GGH PE=1 SV=2	15.82	16.35	1	4	4	6	6.508E7	318	35.9	7.11

P60228	Eukaryotic translation initiation factor 3 subunit E OS=Homo sapiens	15.74	21.80	6	10	10	11	9.508E7	445	52.2	6.04
Q13155	Aminoacyl tRNA synthase complex-interacting multifunctional protein	15.66	25.62	3	6	6	7	1.663E8	320	35.3	8.22
P00338	L-lactate dehydrogenase A chain OS=Homo sapiens GN=LDHA PE=1	15.22	20.78	18	6	7	8	1.841E8	332	36.7	8.27
Q01085	Nucleolysin TIAR OS=Homo sapiens GN=TIAL1 PE=1 SV=1 - [TIAR_	15.21	19.73	11	6	6	6	3.205E7	375	41.6	7.74
Q96C57	Uncharacterized protein C12orf43 OS=Homo sapiens GN=C12orf43 F	15.20	29.01	6	6	6	6	4.149E7	262	28.2	9.42
E7EVY0	Mitochondrial inner membrane protein OXA1L OS=Homo sapiens GN	15.13	18.87	5	7	7	7	8.178E7	408	45.1	9.17
P10319	HLA class I histocompatibility antigen, B-58 alpha chain OS=Homo s	15.09	20.72	43	0	5	5	1.748E8	362	40.3	6.30
P11021	78 kDa glucose-regulated protein OS=Homo sapiens GN=HSPA5 PE=	14.98	16.21	1	6	8	9	6.798E7	654	72.3	5.16
Q9Y314	Nitric oxide synthase-interacting protein OS=Homo sapiens GN=NOS	14.97	23.59	5	5	5	6	6.256E7	301	33.2	8.82
Q9BSV6	tRNA-splicing endonuclease subunit Sen34 OS=Homo sapiens GN=T	14.96	31.29	5	7	7	7	5.941E7	310	33.6	8.43
E9PKG1	Protein arginine N-methyltransferase 1 OS=Homo sapiens GN=PRMT	14.95	21.54	8	5	5	6	6.109E7	325	37.7	6.15
E9PCY7	Heterogeneous nuclear ribonucleoprotein H OS=Homo sapiens GN=I	14.73	13.99	21	2	4	5	1.849E8	429	47.1	6.34
F8VNY5	Thyroid transcription factor 1-associated protein 26 (Fragment) OS=	14.72	25.41	2	5	5	5	5.680E7	181	21.3	9.48
Q6P4A7	Sideroflexin-4 OS=Homo sapiens GN=SFXN4 PE=1 SV=1 - [SFXN4_I	14.64	31.75	2	7	7	8	7.109E7	337	38.0	9.19
J3KTA4	Probable ATP-dependent RNA helicase DDX5 OS=Homo sapiens GN=	14.53	9.45	13	4	5	6	4.865E7	614	69.0	8.85
P29084	Transcription initiation factor IIE subunit beta OS=Homo sapiens GN	14.45	29.55	4	7	7	7	1.108E8	291	33.0	9.66
Q15024	Exosome complex component RRP42 OS=Homo sapiens GN=EXOSC	14.42	18.90	1	4	4	5	8.622E7	291	31.8	5.19
Q9UH62	Armadillo repeat-containing X-linked protein 3 OS=Homo sapiens GN	14.39	21.64	1	6	6	7	1.098E8	379	42.5	8.37
Q9H1E5	Thioredoxin-related transmembrane protein 4 OS=Homo sapiens GN	14.35	18.62	2	5	5	6	8.483E7	349	38.9	4.37
Q8IVS2	Malonyl-CoA-acyl carrier protein transacylase, mitochondrial OS=Hor	14.12	21.28	1	5	5	5	5.559E7	390	42.9	8.72
Q9BU76	Multiple myeloma tumor-associated protein 2 OS=Homo sapiens GN	14.05	27.76	1	6	6	6	4.806E7	263	29.4	10.02
Q9Y383	Putative RNA-binding protein Luc7-like 2 OS=Homo sapiens GN=LUC	13.90	15.82	2	2	6	7	5.128E7	392	46.5	10.01
G3V150	Galactosylgalactosylxylosylprotein 3-beta-glucuronosyltransferase OS	13.88	34.80	3	7	7	7	7.027E7	319	34.9	7.85
Q9H2D1	Mitochondrial folate transporter/carrier OS=Homo sapiens GN=SLC2	13.72	34.60	4	7	7	8	7.939E7	315	35.4	9.45
O75190	DnaJ homolog subfamily B member 6 OS=Homo sapiens GN=DNAJE	13.70	31.90	10	7	7	7	1.435E8	326	36.1	9.16
O75569	Interferon-inducible double-stranded RNA-dependent protein kinase	13.61	17.57	4	5	5	5	7.884E7	313	34.4	8.41
P81605	Dermcidin OS=Homo sapiens GN=DCD PE=1 SV=2 - [DCD_HUMAN]	13.60	35.45	1	3	3	4	1.122E8	110	11.3	6.54
P39023	60S ribosomal protein L3 OS=Homo sapiens GN=RPL3 PE=1 SV=2 -	13.48	20.35	6	6	6	6	5.438E7	403	46.1	10.18
P08238	Heat shock protein HSP 90-beta OS=Homo sapiens GN=HSP90AB1 F	13.40	9.39	9	3	6	7	2.946E7	724	83.2	5.03
Q8IUX4	DNA dC->dU-editing enzyme APOBEC-3F OS=Homo sapiens GN=AP	13.36	24.66	2	6	8	8	9.088E7	373	45.0	7.23
Q14493	Histone RNA hairpin-binding protein OS=Homo sapiens GN=SLBP PE	13.33	15.19	5	4	4	5	9.889E7	270	31.3	7.47
P46109	Crk-like protein OS=Homo sapiens GN=CRKL PE=1 SV=1 - [CRKL_H	13.33	29.37	1	6	6	7	1.030E8	303	33.8	6.74
K7EIE8	Methyl-CpG binding domain protein 3, isoform CRA_b OS=Homo sap	13.24	16.17	5	4	4	5	5.351E7	235	26.3	4.84
Q92667	A-kinase anchor protein 1, mitochondrial OS=Homo sapiens GN=AKA	13.16	6.64	5	5	5	6	4.698E7	903	97.3	4.94
Q9BTV4	Transmembrane protein 43 OS=Homo sapiens GN=TMEM43 PE=1 S	13.11	23.50	1	5	5	6	5.171E7	400	44.8	8.13
K7EQ02	DAZ-associated protein 1 (Fragment) OS=Homo sapiens GN=DAZAP	12.94	14.07	4	3	3	5	7.311E7	327	35.0	7.85
O00401	Neural Wiskott-Aldrich syndrome protein OS=Homo sapiens GN=WA	12.73	19.21	1	7	7	8	6.826E7	505	54.8	7.93
Q95604	HLA class I histocompatibility antigen, Cw-17 alpha chain OS=Homo	12.53	19.09	5	1	4	4	2.599E8	372	41.2	6.80
A0A0B4J1Z1	Serine/arginine-rich-splicing factor 7 OS=Homo sapiens GN=SRSF7 F	12.32	37.23	6	5	5	5	1.367E8	137	15.8	9.80
Q6UX07	Dehydrogenase/reductase SDR family member 13 OS=Homo sapiens	12.25	13.26	1	5	5	5	5.805E7	377	40.8	7.69
P41091	Eukaryotic translation initiation factor 2 subunit 3 OS=Homo sapiens	12.24	10.59	3	3	3	3	9.688E7	472	51.1	8.40
Q9UJZ1	Stomatin-like protein 2, mitochondrial OS=Homo sapiens GN=STOM	12.24	26.40	3	5	5	5	6.776E7	356	38.5	7.39
Q9UMY1	Nucleolar protein 7 OS=Homo sapiens GN=NOL7 PE=1 SV=2 - [NOL	12.19	14.79	2	5	5	5	6.357E7	257	29.4	9.67
P05412	Transcription factor AP-1 OS=Homo sapiens GN=JUN PE=1 SV=2 - [	12.16	14.50	1	3	3	3	1.464E8	331	35.7	8.76
O76071	Probable cytosolic iron-sulfur protein assembly protein CIAO1 OS=Ho	12.02	21.83	1	6	6	6	5.961E7	339	37.8	4.97
H3BV22	Serine/threonine-protein phosphatase (Fragment) OS=Homo sapiens	11.99	12.81	4	1	3	4	5.074E7	203	23.1	5.12
A0A087X271	Calponin (Fragment) OS=Homo sapiens GN=CNN2 PE=1 SV=1 - [A0	11.98	27.93	10	5	5	5	8.101E7	179	19.7	8.79
P20742	Pregnancy zone protein OS=Homo sapiens GN=PZP PE=1 SV=4 - [P	11.98	2.29	2	1	3	5	7.634E7	1482	163.8	6.38
Q9C004	Protein sprouty homolog 4 OS=Homo sapiens GN=SPRY4 PE=1 SV=	11.86	11.71	3	2	2	4	2.014E8	299	32.5	7.88
A0A0G2JNZ2	Protein scribble homolog OS=Homo sapiens GN=SCRIB PE=1 SV=1	11.85	3.25	6	5	5	5	2.051E7	1630	174.8	5.07
P30464	HLA class I histocompatibility antigen, B-15 alpha chain OS=Homo s	11.84	21.55	57	1	5	5	1.748E8	362	40.4	6.30
Q5JVF3	PCI domain-containing protein 2 OS=Homo sapiens GN=PCID2 PE=	11.77	20.30	1	7	7	8	5.683E7	399	46.0	8.53
P01023	Alpha-2-macroglobulin OS=Homo sapiens GN=A2M PE=1 SV=3 - [A	11.45	3.46	1	3	5	6	1.373E8	1474	163.2	6.46

P35998	26S proteasome regulatory subunit 7 OS=Homo sapiens GN=PSMC2	11.31	13.39	3	5	5	6	2.217E7	433	48.6	5.95
P78406	mRNA export factor OS=Homo sapiens GN=RAE1 PE=1 SV=1 - [RAE	11.26	14.95	5	5	5	5	4.530E7	368	40.9	7.83
A2IDA3	DNA-3-methyladenine glycosylase (Fragment) OS=Homo sapiens GN	11.21	20.32	2	4	4	4	6.144E7	251	27.3	9.03
Q8TF74	WAS/WASL-interacting protein family member 2 OS=Homo sapiens (	11.11	15.68	3	6	6	6	8.676E7	440	46.3	10.93
P04818	Thymidylate synthase OS=Homo sapiens GN=TYMS PE=1 SV=3 - [T	11.07	30.35	1	6	6	7	4.904E7	313	35.7	7.01
Q9BW04	Specifically androgen-regulated gene protein OS=Homo sapiens GN=	11.06	19.30	1	6	6	6	7.007E7	601	63.9	8.62
Q00534	Cyclin-dependent kinase 6 OS=Homo sapiens GN=CDK6 PE=1 SV=1	10.98	13.50	56	3	4	6	1.613E8	326	36.9	6.46
O43709	Probable 18S rRNA (guanine-N(7))-methyltransferase OS=Homo sap	10.86	17.79	2	3	3	4	1.261E8	281	31.9	8.73
O95433	Activator of 90 kDa heat shock protein ATPase homolog 1 OS=Homo	10.83	20.71	6	4	4	4	6.610E7	338	38.3	5.53
P61160	Actin-related protein 2 OS=Homo sapiens GN=ACTR2 PE=1 SV=1 -	10.82	14.72	4	5	5	7	5.743E7	394	44.7	6.74
A0A0C4DGR2	Prostate tumor overexpressed gene 1, isoform CRA_d OS=Homo sap	10.69	17.38	7	4	4	4	8.933E7	374	41.5	10.24
P11177	Pyruvate dehydrogenase E1 component subunit beta, mitochondrial	10.60	16.71	3	6	6	6	5.991E7	359	39.2	6.65
F8VYY9	5'-AMP-activated protein kinase subunit gamma-1 OS=Homo sapiens	10.60	23.93	10	5	5	5	5.586E7	280	31.9	8.57
Q5T440	Putative transferase CAF17, mitochondrial OS=Homo sapiens GN=IB	10.54	16.85	1	4	4	4	5.035E7	356	38.1	9.83
Q9UBM7	7-dehydrocholesterol reductase OS=Homo sapiens GN=DHCR7 PE=1	10.50	11.16	8	4	4	4	1.126E8	475	54.5	8.70
P15407	Fos-related antigen 1 OS=Homo sapiens GN=FOSL1 PE=1 SV=1 - [F	10.43	8.12	1	2	2	3	1.015E8	271	29.4	8.02
P31942	Heterogeneous nuclear ribonucleoprotein H3 OS=Homo sapiens GN=	10.36	12.72	1	4	4	5	3.227E7	346	36.9	6.87
Q6P087	RNA pseudouridylate synthase domain-containing protein 3 OS=Hon	10.31	15.10	6	5	5	5	3.363E7	351	38.4	10.32
Q7Z7K6	Centromere protein V OS=Homo sapiens GN=CENPV PE=1 SV=1 - [	10.25	25.45	2	4	4	6	4.771E7	275	29.9	9.73
P36578	60S ribosomal protein L4 OS=Homo sapiens GN=RPL4 PE=1 SV=5 -	10.21	11.71	4	5	5	5	5.984E7	427	47.7	11.06
Q8TBX8	Phosphatidylinositol 5-phosphate 4-kinase type-2 gamma OS=Homo	10.20	15.91	4	6	6	6	3.492E7	421	47.3	6.84
K7EQL4	Troponin T, slow skeletal muscle (Fragment) OS=Homo sapiens GN=	10.13	22.86	17	4	4	6	8.306E7	210	25.3	9.66
F8VVM2	Phosphate carrier protein, mitochondrial OS=Homo sapiens GN=SLC	10.12	13.27	5	4	4	5	5.989E7	324	36.1	9.26
O15143	Actin-related protein 2/3 complex subunit 1B OS=Homo sapiens GN=	10.09	14.25	8	4	4	4	3.166E7	372	40.9	8.35
E9PHS0	LanC-like protein 1 (Fragment) OS=Homo sapiens GN=LANCL1 PE=1	10.05	33.67	3	5	5	5	8.921E7	196	22.0	8.15
Q5JR04	Mov10, Moloney leukemia virus 10, homolog (Mouse), isoform CRA_	9.95	4.75	2	4	4	4	2.293E7	947	107.1	8.79
K7ELV2	Nucleoporin SEH1 (Fragment) OS=Homo sapiens GN=SEH1L PE=1 S	9.92	22.81	5	4	4	4	4.636E7	263	29.5	7.80
J3KT86	Aurora kinase B (Fragment) OS=Homo sapiens GN=AURKB PE=1 SV	9.90	16.12	14	4	4	5	1.886E7	242	28.0	9.39
P63096	Guanine nucleotide-binding protein G(i) subunit alpha-1 OS=Homo s	9.87	18.93	27	5	5	6	7.539E7	354	40.3	5.97
Q9H7B2	Ribosome production factor 2 homolog OS=Homo sapiens GN=RPF2	9.84	26.14	4	7	7	8	2.039E7	306	35.6	9.99
Q9H4I3	TraB domain-containing protein OS=Homo sapiens GN=TRABD PE=	9.83	14.63	2	4	4	5	3.368E8	376	42.3	8.00
E7EP32	Guanine nucleotide-binding protein G(I)/G(S)/G(T) subunit beta-2 O	9.67	12.84	17	4	4	4	8.919E7	296	32.4	6.15
P19474	E3 ubiquitin-protein ligase TRIM21 OS=Homo sapiens GN=TRIM21 F	9.64	9.47	1	5	5	6	6.324E7	475	54.1	6.38
P50479	PDZ and LIM domain protein 4 OS=Homo sapiens GN=PDLIM4 PE=1	9.64	25.45	2	6	6	8	7.661E7	330	35.4	7.91
H7BY36	RNA-binding protein EWS (Fragment) OS=Homo sapiens GN=EWSR	9.62	9.42	5	2	2	3	6.185E7	308	32.2	9.82
Q8TB36	Ganglioside-induced differentiation-associated protein 1 OS=Homo s	9.62	12.57	1	3	3	3	2.626E7	358	41.3	8.34
P12236	ADP/ATP translocase 3 OS=Homo sapiens GN=SLC25A6 PE=1 SV=4	9.41	13.42	3	1	4	4	3.958E7	298	32.8	9.74
P01857	Immunoglobulin heavy constant gamma 1 OS=Homo sapiens GN=IG	9.36	13.64	6	4	4	5	7.762E7	330	36.1	8.19
P05141	ADP/ATP translocase 2 OS=Homo sapiens GN=SLC25A5 PE=1 SV=7	9.27	13.42	3	1	4	4	5.382E7	298	32.8	9.69
Q7Z417	Nuclear fragile X mental retardation-interacting protein 2 OS=Homo	9.15	12.23	1	6	6	6	3.490E7	695	76.1	8.70
A0A087WXC5	NADH dehydrogenase [ubiquinone] 1 alpha subcomplex subunit 10,	9.04	15.49	8	4	4	4	5.485E7	355	40.8	8.48
Q8NBN7	Retinol dehydrogenase 13 OS=Homo sapiens GN=RDH13 PE=1 SV=	9.01	17.52	2	4	4	4	2.870E7	331	35.9	8.10
F5GYN4	Ubiquitin thioesterase OTUB1 OS=Homo sapiens GN=OTUB1 PE=1 S	8.99	28.22	6	6	6	6	3.978E7	241	28.0	5.29
Q96J01	THO complex subunit 3 OS=Homo sapiens GN=THOC3 PE=1 SV=1 -	8.97	8.55	4	4	4	4	6.027E7	351	38.7	6.09
P45954	Short/branched chain specific acyl-CoA dehydrogenase, mitochondri	8.91	18.06	1	5	5	6	3.731E7	432	47.5	6.99
O00767	Acyl-CoA desaturase OS=Homo sapiens GN=SCD PE=1 SV=2 - [ACO	8.91	9.19	1	4	4	4	6.795E7	359	41.5	9.00
P56470	Galectin-4 OS=Homo sapiens GN=LGALS4 PE=1 SV=1 - [LEG4_HUM	8.79	11.46	2	3	3	4	2.592E7	323	35.9	9.16
Q9NUQ2	1-acyl-sn-glycerol-3-phosphate acyltransferase epsilon OS=Homo sa	8.73	16.48	3	4	4	4	7.381E7	364	42.0	9.10
A0A087WTZ5	UBX domain-containing protein 1 OS=Homo sapiens GN=UBXN1 PE=	8.72	29.83	5	4	4	4	7.441E7	238	27.0	7.96
P12956	X-ray repair cross-complementing protein 6 OS=Homo sapiens GN=X	8.64	8.05	2	4	4	4	2.210E7	609	69.8	6.64
O15371	Eukaryotic translation initiation factor 3 subunit D OS=Homo sapiens	8.62	6.93	3	3	3	3	2.719E7	548	63.9	6.05
Q6IN84	rRNA methyltransferase 1, mitochondrial OS=Homo sapiens GN=MR	8.62	19.83	2	5	5	5	6.803E7	353	38.6	7.94
H0YKB1	Tight junction protein 1 (Zona occludens 1), isoform CRA_d OS=Hor	8.57	15.14	6	4	4	4	3.281E7	383	42.4	9.51

K7EML3	Chromosome 19 open reading frame 66 (Fragment) OS=Homo sapie	8.56	40.85	2	2	2	3	1.189E8	71	8.1	4.83
F5H6G4	Protein RMD5 homolog B OS=Homo sapiens GN=RMND5B PE=1 SV=	8.47	8.42	2	2	2	4	3.370E7	380	42.7	6.92
B4DY09	Interleukin enhancer-binding factor 2 OS=Homo sapiens GN=ILF2 P	8.45	18.18	4	4	4	5	3.855E7	352	38.9	4.94
D6RAD4	Cyclin-dependent kinase 7 OS=Homo sapiens GN=CDK7 PE=1 SV=1	8.22	14.24	4	3	3	3	4.705E7	309	34.6	8.28
P52597	Heterogeneous nuclear ribonucleoprotein F OS=Homo sapiens GN=H	8.15	9.40	4	1	3	4	1.074E8	415	45.6	5.58
A0A087WVC4	cAMP-dependent protein kinase catalytic subunit beta OS=Homo sap	8.10	9.17	11	1	4	4	3.870E7	338	39.2	8.82
Q9NX31	Oxidative stress-responsive serine-rich protein 1 OS=Homo sapiens (	8.09	13.70	1	3	3	3	3.882E7	292	31.8	8.48
C9J384	Protein CMSS1 (Fragment) OS=Homo sapiens GN=CMSS1 PE=1 SV=	8.04	20.00	3	4	4	4	5.481E7	225	26.2	9.88
F5GWT4	Serine/threonine-protein kinase WNK1 OS=Homo sapiens GN=WNK1	8.00	4.26	3	7	7	7	6.738E7	2134	225.4	6.44
O00165	HCLS1-associated protein X-1 OS=Homo sapiens GN=HAX1 PE=1 SV	8.00	11.47	3	3	3	3	2.744E7	279	31.6	4.92
K7EMD6	Small glutamine-rich tetratricopeptide repeat-containing protein alph	7.99	33.95	3	4	4	5	1.501E7	162	17.8	4.75
J3KQL8	Apolipoprotein L2 OS=Homo sapiens GN=APOL2 PE=1 SV=2 - [J3KQ	7.87	6.24	3	2	2	3	3.726E7	449	48.9	6.00
Q99612	Krueppel-like factor 6 OS=Homo sapiens GN=KLF6 PE=1 SV=3 - [KL	7.86	8.48	22	2	2	3	8.600E7	283	31.8	6.92
P55884	Eukaryotic translation initiation factor 3 subunit B OS=Homo sapiens	7.74	3.93	1	3	3	3	5.219E7	814	92.4	5.00
Q9HBH5	Retinol dehydrogenase 14 OS=Homo sapiens GN=RDH14 PE=1 SV=	7.73	11.61	1	3	3	3	1.452E7	336	36.8	8.79
Q9ULR0	Pre-mRNA-splicing factor ISY1 homolog OS=Homo sapiens GN=ISY1	7.64	12.98	4	3	3	3	3.452E7	285	33.0	5.17
Q8ND90	Paraneoplastic antigen Ma1 OS=Homo sapiens GN=PNMA1 PE=1 SV	7.60	7.65	1	2	2	3	1.904E7	353	39.7	4.83
P04004	Vitronectin OS=Homo sapiens GN=VTN PE=1 SV=1 - [VTNC_HUMAN	7.55	4.81	1	2	2	3	1.790E8	478	54.3	5.80
P55735	Protein SEC13 homolog OS=Homo sapiens GN=SEC13 PE=1 SV=3 -	7.53	18.01	2	4	4	4	5.550E7	322	35.5	5.48
P19525	Interferon-induced, double-stranded RNA-activated protein kinase O	7.51	6.17	3	3	3	3	1.308E7	551	62.1	8.40
P06702	Protein S100-A9 OS=Homo sapiens GN=S100A9 PE=1 SV=1 - [S10A	7.51	24.56	1	2	2	3	4.251E7	114	13.2	6.13
Q6PK04	Coiled-coil domain-containing protein 137 OS=Homo sapiens GN=CC	7.50	11.76	2	3	3	3	2.647E7	289	33.2	10.93
Q9UBV7	Beta-1,4-galactosyltransferase 7 OS=Homo sapiens GN=B4GALT7 PE	7.44	14.07	3	4	4	4	2.355E7	327	37.4	8.98
B4DQT1	Macrophage erythroblast attacher OS=Homo sapiens GN=MAEA PE=	7.38	8.91	5	3	3	3	2.985E7	348	39.8	8.53
C9JRD2	DnaJ homolog subfamily B member 2 (Fragment) OS=Homo sapiens	7.37	20.18	6	3	3	3	3.783E7	228	25.4	5.73
Q96QE5	Transcription elongation factor, mitochondrial OS=Homo sapiens GN	7.25	8.61	2	4	4	4	2.363E7	360	41.6	9.32
Q15018	BRISC complex subunit Abraxas 2 OS=Homo sapiens GN=ABRAXAS2	7.19	14.70	1	5	5	5	6.573E7	415	46.9	6.21
J3QL05	Serine/arginine-rich-splicing factor 2 (Fragment) OS=Homo sapiens (	7.18	27.69	3	2	3	3	7.374E7	130	15.1	10.96
P17612	cAMP-dependent protein kinase catalytic subunit alpha OS=Homo sa	7.09	8.83	4	1	4	4	3.812E7	351	40.6	8.79
Q9Y679	Ancient ubiquitous protein 1 OS=Homo sapiens GN=AUP1 PE=1 SV=	7.04	13.24	1	5	5	6	2.005E7	476	53.0	8.09
A4D1E9	GTP-binding protein 10 OS=Homo sapiens GN=GTPBP10 PE=1 SV=1	7.02	14.73	4	5	5	5	6.142E7	387	42.9	9.03
P49770	Translation initiation factor eIF-2B subunit beta OS=Homo sapiens G	6.97	16.81	4	5	5	5	2.200E7	351	39.0	6.16
Q17RN3	Protein FAM98C OS=Homo sapiens GN=FAM98C PE=2 SV=1 - [FA98	6.92	9.46	3	2	2	2	1.885E7	349	37.3	7.18
Q9UBQ7	Glyoxylate reductase/hydroxypyruvate reductase OS=Homo sapiens	6.91	13.11	2	3	3	3	2.360E7	328	35.6	7.39
Q9UBP9	PTB domain-containing engulfment adapter protein 1 OS=Homo sap	6.84	11.18	2	4	4	4	2.303E7	304	34.5	7.90
O60547	GDP-mannose 4,6 dehydratase OS=Homo sapiens GN=GMDS PE=1	6.82	15.59	1	4	4	4	3.078E7	372	41.9	7.31
O15427	Monocarboxylate transporter 4 OS=Homo sapiens GN=SLC16A3 PE=	6.79	7.31	5	3	3	3	2.196E7	465	49.4	7.96
Q15233	Non-POU domain-containing octamer-binding protein OS=Homo sap	6.77	7.43	5	3	3	3	2.308E7	471	54.2	8.95
Q8TCE1	Antithrombin-III OS=Homo sapiens GN=SERPINC1 PE=1 SV=1 - [Q8	6.76	4.25	2	1	1	2	5.807E7	259	29.1	8.81
F5H1F6	Vacuolar protein sorting-associated protein 37B (Fragment) OS=Hon	6.71	29.89	3	3	3	4	3.936E7	184	20.6	6.19
P53597	Succinate--CoA ligase [ADP/GDP-forming] subunit alpha, mitochondr	6.66	11.27	1	2	2	2	7.127E7	346	36.2	8.79
Q7Z2W4	Zinc finger CCCH-type antiviral protein 1 OS=Homo sapiens GN=ZC3	6.53	5.76	3	4	4	4	2.845E7	902	101.4	8.40
H3BPZ1	Very-long-chain (3R)-3-hydroxyacyl-CoA dehydratase OS=Homo sap	6.52	10.39	5	3	3	3	1.276E7	337	40.0	8.97
Q96MX6	WD repeat-containing protein 92 OS=Homo sapiens GN=WDR92 PE	6.52	6.44	1	2	2	3	3.610E7	357	39.7	8.09
O00622	Protein CYR61 OS=Homo sapiens GN=CYR61 PE=1 SV=1 - [CYR61_	6.51	7.35	1	3	3	3	4.791E7	381	42.0	8.21
O00154	Cytosolic acyl coenzyme A thioester hydrolase OS=Homo sapiens GN	6.48	7.11	2	2	2	2	4.787E7	380	41.8	8.54
Q9NWB6	Arginine and glutamate-rich protein 1 OS=Homo sapiens GN=ARGLU	6.43	13.19	2	5	5	5	3.156E7	273	33.2	10.35
H3BSK9	Ataxin-2-like protein (Fragment) OS=Homo sapiens GN=ATXN2L PE=	6.37	14.58	5	4	4	5	2.154E7	336	37.1	7.24
P13747	HLA class I histocompatibility antigen, alpha chain E OS=Homo sapie	6.37	10.61	19	2	3	3	1.166E8	358	40.1	5.95
Q8NBU5	ATPase family AAA domain-containing protein 1 OS=Homo sapiens (	6.25	5.54	1	2	2	2	4.358E7	361	40.7	6.90
Q9Y2P8	RNA 3'-terminal phosphate cyclase-like protein OS=Homo sapiens GI	6.17	12.60	4	4	4	4	2.699E7	373	40.8	9.26
E7EX73	Eukaryotic translation initiation factor 4 gamma 1 OS=Homo sapiens	6.15	3.13	5	3	3	4	8.224E7	1436	158.5	5.21
Q9H5Q4	Dimethyladenosine transferase 2, mitochondrial OS=Homo sapiens (	6.14	8.33	1	3	3	3	4.135E7	396	45.3	9.19

H7C3X5	Syntaxin-5 (Fragment) OS=Homo sapiens GN=STX5 PE=1 SV=1 - [H	6.11	24.05	3	2	2	2	4.699E7	158	17.6	7.06
Q96CB9	5-methylcytosine rRNA methyltransferase NSUN4 OS=Homo sapiens	6.11	11.72	2	3	3	3	2.898E7	384	43.1	8.18
O00303	Eukaryotic translation initiation factor 3 subunit F OS=Homo sapiens	6.09	13.17	2	3	3	3	3.630E7	357	37.5	5.45
H0YMU3	Isocitrate dehydrogenase [NAD] subunit alpha, mitochondrial (Fragn	6.04	18.86	6	3	3	3	3.121E7	175	19.2	9.47
P10909	Clusterin OS=Homo sapiens GN=CLU PE=1 SV=1 - [CLUS_HUMAN]	6.00	10.02	10	3	3	3	9.873E7	449	52.5	6.27
J3QKT4	Pyrroline-5-carboxylate reductase (Fragment) OS=Homo sapiens GN	5.89	9.92	5	1	2	2	3.793E7	242	25.6	6.61
Q9Y3A4	Ribosomal RNA-processing protein 7 homolog A OS=Homo sapiens (	5.78	9.29	1	2	2	2	3.998E7	280	32.3	9.58
C9JZR2	Catenin delta-1 OS=Homo sapiens GN=CTNND1 PE=1 SV=2 - [C9JZ	5.77	3.73	2	3	3	3	1.748E7	938	104.8	6.87
P53004	Biliverdin reductase A OS=Homo sapiens GN=BLVRA PE=1 SV=2 - [	5.68	13.85	2	3	3	3	2.557E7	296	33.4	6.44
H0Y2N6	Casein kinase I isoform delta (Fragment) OS=Homo sapiens GN=CSI	5.55	20.83	9	2	2	2	3.779E7	120	13.8	9.26
G3V2J8	Heat shock protein HSP 90-alpha (Fragment) OS=Homo sapiens GN=	5.55	29.31	5	1	4	4	1.391E7	174	20.1	4.63
E9PLA9	Caprin-1 (Fragment) OS=Homo sapiens GN=CAPRIN1 PE=1 SV=1 -	5.52	12.37	3	2	2	2	4.346E7	186	20.2	7.40
C9JP00	Muscleblind-like protein 1 OS=Homo sapiens GN=MBNL1 PE=1 SV=	5.46	8.62	7	2	2	2	3.772E7	348	37.9	8.75
F8VUC8	2'-5'-oligoadenylate synthase 1 OS=Homo sapiens GN=OAS1 PE=1 S	5.37	16.95	4	2	2	2	2.161E7	177	19.9	7.69
Q9BYB4	Guanine nucleotide-binding protein subunit beta-like protein 1 OS=H	5.31	12.84	1	3	3	3	2.030E7	327	35.6	7.97
P35237	Serpin B6 OS=Homo sapiens GN=SERPINB6 PE=1 SV=3 - [SPB6_HU	5.26	11.97	3	3	3	3	2.016E7	376	42.6	5.27
O43390	Heterogeneous nuclear ribonucleoprotein R OS=Homo sapiens GN=I	5.22	5.21	2	1	3	3	1.284E8	633	70.9	8.13
E5RIU6	Cyclin-dependent kinase 1 (Fragment) OS=Homo sapiens GN=CDK1	5.21	9.52	59	1	2	2	2.164E8	189	21.7	8.51
Q8NBS9	Thioredoxin domain-containing protein 5 OS=Homo sapiens GN=TXI	5.14	6.71	1	2	2	2	5.383E7	432	47.6	5.97
Q5JW28	Double-stranded RNA-binding protein Staufen homolog 1 (Fragment	5.14	10.55	4	2	2	2	5.068E7	199	22.5	9.95
R4GND3	Pleckstrin homology-like domain family A member 1 OS=Homo sapie	5.13	6.15	2	2	2	2	5.965E7	260	29.6	9.20
M0R0I0	BRISC and BRCA1-A complex member 1 (Fragment) OS=Homo sapie	5.12	29.33	9	3	3	3	3.018E7	225	24.6	4.61
Q01650	Large neutral amino acids transporter small subunit 1 OS=Homo sap	5.08	4.93	6	2	2	2	1.117E8	507	55.0	7.72
Q9NYT0	Pleckstrin-2 OS=Homo sapiens GN=PLEK2 PE=1 SV=1 - [PLEK2_HU	5.01	14.45	4	4	4	4	3.122E7	353	39.9	9.41
A6NP24	Quinone oxidoreductase (Fragment) OS=Homo sapiens GN=CRYZ PI	4.95	15.23	3	2	2	2	2.341E7	243	26.0	8.88
O43427	Acidic fibroblast growth factor intracellular-binding protein OS=Hom	4.93	10.99	4	3	3	3	1.614E7	364	41.9	6.48
Q96IZ0	PRKC apoptosis WT1 regulator protein OS=Homo sapiens GN=PAWF	4.89	8.53	1	2	2	2	1.455E7	340	36.5	5.41
H3BQQ6	Protein FAM192A (Fragment) OS=Homo sapiens GN=FAM192A PE=1	4.87	26.04	14	2	2	2	3.355E7	96	11.8	4.75
O95900	Probable tRNA pseudouridine synthase 2 OS=Homo sapiens GN=TRI	4.83	7.25	1	2	2	2	2.874E7	331	36.7	8.98
C9J050	Choline-phosphate cytidylyltransferase A (Fragment) OS=Homo sapi	4.78	7.51	6	2	2	2	2.435E7	293	33.7	6.74
Q5T3N1	Annexin (Fragment) OS=Homo sapiens GN=ANXA1 PE=1 SV=1 - [Q	4.78	20.10	3	3	3	3	1.864E7	204	22.7	5.53
Q15293	Reticulocalbin-1 OS=Homo sapiens GN=RCN1 PE=1 SV=1 - [RCN1_I	4.77	13.29	2	3	3	3	3.273E7	331	38.9	5.00
P53985	Monocarboxylate transporter 1 OS=Homo sapiens GN=SLC16A1 PE=	4.73	6.40	3	2	2	3	4.751E7	500	53.9	8.66
F8VZY9	Keratin, type I cytoskeletal 18 OS=Homo sapiens GN=KRT18 PE=1 S	4.73	5.37	2	1	2	2	6.892E7	391	43.7	5.35
H0YKU1	Tropomodulin-3 (Fragment) OS=Homo sapiens GN=TMOD3 PE=1 SV	4.72	16.58	3	2	2	2	4.066E7	187	20.9	7.43
P62917	60S ribosomal protein L8 OS=Homo sapiens GN=RPL8 PE=1 SV=2 -	4.71	13.23	5	3	3	3	3.119E7	257	28.0	11.03
P00734	Prothrombin OS=Homo sapiens GN=F2 PE=1 SV=2 - [THRB_HUMAN	4.66	2.41	2	2	2	2	4.251E7	622	70.0	5.90
P62753	40S ribosomal protein S6 OS=Homo sapiens GN=RPS6 PE=1 SV=1 -	4.65	14.86	3	3	3	4	2.293E7	249	28.7	10.84
Q53EU6	Glycerol-3-phosphate acyltransferase 3 OS=Homo sapiens GN=GPAT	4.63	5.99	1	2	2	2	2.499E7	434	48.7	8.87
Q6PK18	2-oxoglutarate and iron-dependent oxygenase domain-containing pr	4.60	13.17	2	3	3	3	1.946E7	319	35.6	8.18
O00257	E3 SUMO-protein ligase CBX4 OS=Homo sapiens GN=CBX4 PE=1 SV	4.58	5.36	2	3	3	3	1.836E7	560	61.3	9.36
O75436	Vacuolar protein sorting-associated protein 26A OS=Homo sapiens G	4.58	6.12	2	2	2	2	2.430E7	327	38.1	6.57
H3BM30	Enoyl-[acyl-carrier-protein] reductase, mitochondrial (Fragment) OS=	4.58	6.60	2	1	1	1	9.434E7	212	22.9	5.91
Q96BW9	Phosphatidate cytidylyltransferase, mitochondrial OS=Homo sapiens	4.55	11.06	5	3	3	3	2.054E7	452	51.0	7.94
Q8TB40	Protein ABHD4 OS=Homo sapiens GN=ABHD4 PE=1 SV=1 - [ABHD4	4.55	8.48	2	2	2	2	2.896E7	342	38.8	7.59
Q9H9L3	Interferon-stimulated 20 kDa exonuclease-like 2 OS=Homo sapiens (	4.52	6.80	1	2	2	2	1.323E7	353	39.1	9.94
P24752	Acetyl-CoA acetyltransferase, mitochondrial OS=Homo sapiens GN=/	4.52	11.24	3	3	3	3	2.575E7	427	45.2	8.85
Q6NZI2	Caveolae-associated protein 1 OS=Homo sapiens GN=CAVIN1 PE=1	4.52	7.44	1	2	2	2	9.480E6	390	43.4	5.60
Q9Y3B9	RRP15-like protein OS=Homo sapiens GN=RRP15 PE=1 SV=2 - [RRF	4.45	7.80	1	2	2	2	3.284E7	282	31.5	5.52
Q96MG7	Non-structural maintenance of chromosomes element 3 homolog OS	4.44	10.53	1	3	3	3	1.562E7	304	34.3	9.28
P56937	3-keto-steroid reductase OS=Homo sapiens GN=HSD17B7 PE=1 SV=	4.40	6.16	2	2	2	2	1.037E7	341	38.2	8.10
X6RLL4	Ribonuclease P protein subunit p40 OS=Homo sapiens GN=RPP40 P	4.40	16.17	4	3	3	4	3.438E7	303	34.7	5.60
Q5VY09	Immediate early response gene 5 protein OS=Homo sapiens GN=IEI	4.38	10.40	1	3	3	3	1.080E7	327	33.7	4.96

Q9Y2C4	Nuclease EXOG, mitochondrial OS=Homo sapiens GN=EXOG PE=1 S	4.37	7.61	4	2	2	2	2.743E7	368	41.1	8.27
A0A0A0MRR5	Uracil phosphoribosyltransferase homolog OS=Homo sapiens GN=UP	4.37	7.12	3	2	2	2	4.854E7	281	30.7	6.15
O95159	Zinc finger protein-like 1 OS=Homo sapiens GN=ZFPL1 PE=1 SV=2 -	4.28	8.39	4	2	2	3	4.940E7	310	34.1	8.07
H7C1I0	Solute carrier family 35 member E1 (Fragment) OS=Homo sapiens G	4.21	13.37	2	3	3	3	1.431E7	344	38.2	9.92
A0A0B4J207	Ribose-phosphate pyrophosphokinase 3 OS=Homo sapiens GN=PRP	4.19	9.43	8	2	2	2	2.132E7	318	34.8	6.35
Q86W42	THO complex subunit 6 homolog OS=Homo sapiens GN=THOC6 PE=	4.16	17.60	1	3	3	3	3.392E7	341	37.5	7.43
K7ER96	Thioredoxin-like protein 1 (Fragment) OS=Homo sapiens GN=TXNL1	4.15	7.83	3	2	2	2	3.394E7	281	31.4	4.83
Q96GY0	Zinc finger C2HC domain-containing protein 1A OS=Homo sapiens G	4.06	15.38	2	4	4	4	1.543E7	325	35.1	9.82
Q14978	Nucleolar and coiled-body phosphoprotein 1 OS=Homo sapiens GN=	4.05	2.43	2	2	2	2	5.490E7	699	73.6	9.47
Q06609	DNA repair protein RAD51 homolog 1 OS=Homo sapiens GN=RAD51	4.04	6.19	1	2	2	2	1.215E7	339	36.9	5.60
Q8WU90	Zinc finger CCCH domain-containing protein 15 OS=Homo sapiens G	4.04	4.69	1	2	2	2	2.827E7	426	48.6	5.31
Q8N5P1	Zinc finger CCCH domain-containing protein 8 OS=Homo sapiens GN	4.03	8.59	2	2	2	2	5.473E7	291	33.6	8.28
P29992	Guanine nucleotide-binding protein subunit alpha-11 OS=Homo sapi	4.03	5.57	4	2	2	2	1.441E7	359	42.1	5.69
Q9BXW7	Haloacid dehalogenase-like hydrolase domain-containing 5 OS=Hom	3.97	4.26	1	2	2	2	1.775E7	423	46.3	8.13
H0YE89	RalBP1-associated Eps domain-containing protein 1 (Fragment) OS=	3.95	26.23	5	1	1	1	3.401E7	61	6.7	10.27
A0A087X1D8	Farnesyl pyrophosphate synthase (Fragment) OS=Homo sapiens GN	3.93	12.64	3	2	2	2	1.874E7	174	20.0	6.19
M0QX65	SUMO-activating enzyme subunit 1 (Fragment) OS=Homo sapiens G	3.91	9.27	7	2	2	2	1.478E7	205	22.6	7.33
Q8N6M0	OTU domain-containing protein 6B OS=Homo sapiens GN=OTUD6B	3.89	11.95	3	2	2	2	3.275E7	293	33.8	6.05
Q13123	Protein Red OS=Homo sapiens GN=IK PE=1 SV=3 - [RED_HUMAN]	3.88	3.23	3	2	2	2	7.494E6	557	65.6	6.64
O43347	RNA-binding protein Musashi homolog 1 OS=Homo sapiens GN=MSI	3.86	6.91	2	1	2	2	5.400E7	362	39.1	7.85
P35241	Radixin OS=Homo sapiens GN=RDX PE=1 SV=1 - [RADI_HUMAN]	3.86	7.20	7	5	5	5	4.951E7	583	68.5	6.37
Q6UXN9	WD repeat-containing protein 82 OS=Homo sapiens GN=WDR82 PE	3.77	10.54	1	3	3	3	1.843E7	313	35.1	7.69
Q96B26	Exosome complex component RRP43 OS=Homo sapiens GN=EXOSC	3.72	9.06	1	2	2	2	4.459E7	276	30.0	5.30
Q53HC0	Coiled-coil domain-containing protein 92 OS=Homo sapiens GN=CCD	3.60	5.44	4	2	2	2	1.390E7	331	36.9	8.90
P17275	Transcription factor jun-B OS=Homo sapiens GN=JUNB PE=1 SV=1	3.60	7.78	1	2	2	2	1.661E7	347	35.9	9.22
P69905	Hemoglobin subunit alpha OS=Homo sapiens GN=HBA1 PE=1 SV=2	3.59	16.90	2	2	2	2	3.176E7	142	15.2	8.68
P56962	Syntaxin-17 OS=Homo sapiens GN=STX17 PE=1 SV=2 - [STX17_HU	3.59	6.62	1	1	1	1	4.372E7	302	33.4	6.57
H7BZK6	Ubiquitin carboxyl-terminal hydrolase 46 OS=Homo sapiens GN=USP	3.54	7.67	3	2	2	2	2.713E7	339	39.5	7.36
Q99836	Myeloid differentiation primary response protein MyD88 OS=Homo s	3.53	7.09	4	1	1	1	2.363E7	296	33.2	6.15
V9GYR2	Sodium/potassium-transporting ATPase subunit beta (Fragment) OS	3.53	19.23	2	2	2	3	9.918E7	130	15.1	6.16
H0YHC3	Nucleosome assembly protein 1-like 1 (Fragment) OS=Homo sapiens	3.53	14.14	15	2	2	2	1.644E7	198	23.4	4.81
P07477	Trypsin-1 OS=Homo sapiens GN=PRSS1 PE=1 SV=1 - [TRY1_HUMA	3.50	11.34	12	2	2	2	1.018E8	247	26.5	6.51
H3BPG5	RNA binding protein S1, serine-rich domain, isoform CRA_c OS=Hom	3.48	11.72	6	1	1	1	5.026E7	128	15.1	11.87
P20042	Eukaryotic translation initiation factor 2 subunit 2 OS=Homo sapiens	3.44	4.50	1	2	2	2	8.287E6	333	38.4	5.80
E9PQW0	Peroxisomal membrane protein PEX16 OS=Homo sapiens GN=PEX16	3.40	26.67	4	2	2	2	1.741E7	60	6.8	8.69
F5H6X0	General transcription factor IIH subunit 3 (Fragment) OS=Homo sap	3.30	8.11	6	1	1	1	4.731E7	148	16.4	7.56
Q9Y5J1	U3 small nucleolar RNA-associated protein 18 homolog OS=Homo sa	3.29	2.34	1	1	1	1	3.815E7	556	62.0	8.76
P24385	G1/S-specific cyclin-D1 OS=Homo sapiens GN=CCND1 PE=1 SV=1 -	3.27	6.44	1	2	2	2	3.378E7	295	33.7	5.02
H3BRL9	Endonuclease III-like protein 1 (Fragment) OS=Homo sapiens GN=N	3.22	7.80	2	1	1	1	2.980E7	218	23.8	9.73
H0YIZ6	HAUS augmin-like complex subunit 4 (Fragment) OS=Homo sapiens	3.21	15.28	7	2	2	2	1.824E7	144	16.6	5.36
A0A0D9SFB3	ATP-dependent RNA helicase DDX3X OS=Homo sapiens GN=DDX3X	3.21	2.97	8	1	2	2	2.441E7	640	70.8	7.36
H7C0S9	WD repeat domain-containing protein 83 (Fragment) OS=Homo sapi	3.18	18.92	3	1	1	1	1.975E7	74	8.0	9.38
P48651	Phosphatidylserine synthase 1 OS=Homo sapiens GN=PTDSS1 PE=1	3.17	2.75	1	1	1	1	2.678E7	473	55.5	8.43
H0Y9P0	Receptor of-activated protein C kinase 1 (Fragment) OS=Homo sapie	3.15	19.28	9	1	1	1	2.969E7	83	8.8	4.42
H0YJ92	CDK-activating kinase assembly factor MAT1 (Fragment) OS=Homo s	3.15	13.02	3	1	1	1	1.386E7	169	19.4	5.58
M0R1Z5	Lysophospholipid acyltransferase 7 (Fragment) OS=Homo sapiens G	3.15	12.15	3	1	1	1	4.575E7	181	19.8	9.03
C9J9W2	LIM and SH3 domain protein 1 (Fragment) OS=Homo sapiens GN=L	3.14	7.83	2	1	1	1	8.213E7	166	19.0	9.01
Q92522	Histone H1x OS=Homo sapiens GN=H1FX PE=1 SV=1 - [H1X_HUMA	3.11	4.69	1	1	1	1	3.245E7	213	22.5	10.76
Q00403	Transcription initiation factor IIB OS=Homo sapiens GN=GTF2B PE=	3.03	4.43	1	1	1	1	3.553E7	316	34.8	8.35
B4DNK4	Pyruvate kinase OS=Homo sapiens GN=PKM PE=1 SV=1 - [B4DNK4	3.02	7.22	6	3	3	3	2.836E7	457	49.9	7.83
F8W733	BRISC and BRCA1-A complex member 2 (Fragment) OS=Homo sapie	3.01	4.93	2	1	1	1	3.134E7	284	32.0	4.89
Q9Y371	Endophilin-B1 OS=Homo sapiens GN=SH3GLB1 PE=1 SV=1 - [SHLB	3.00	4.11	2	1	1	1	1.757E7	365	40.8	6.04
Q8NFH4	Nucleoporin Nup37 OS=Homo sapiens GN=NUP37 PE=1 SV=1 - [NU	3.00	4.91	1	1	1	1	5.266E7	326	36.7	5.92

F8WJN3	Cleavage and polyadenylation-specificity factor subunit 6 OS=Homo	2.99	2.93	2	1	1	1	4.957E7	478	52.2	6.43
B1AN99	Trypsin-3 (Fragment) OS=Homo sapiens GN=PRSS3 PE=1 SV=8 - [B	2.98	7.34	2	1	1	1	1.097E8	177	19.3	6.07
Q9UGR2	Zinc finger CCCH domain-containing protein 7B OS=Homo sapiens G	2.91	1.41	1	1	1	1	1.653E7	993	111.5	7.17
P31689	DnaJ homolog subfamily A member 1 OS=Homo sapiens GN=DNAJA	2.90	4.79	4	2	2	2	2.483E7	397	44.8	7.08
P62701	40S ribosomal protein S4, X isoform OS=Homo sapiens GN=RPS4X F	2.89	3.42	1	1	1	1	1.754E7	263	29.6	10.15
H0Y698	Acyl-coenzyme A thioesterase 8 (Fragment) OS=Homo sapiens GN=	2.88	21.62	7	1	1	1	4.556E7	74	8.4	9.10
G3V4X1	26S proteasome regulatory subunit 4 (Fragment) OS=Homo sapiens	2.87	14.29	10	1	1	1	4.829E7	84	9.1	4.61
Q13751	Laminin subunit beta-3 OS=Homo sapiens GN=LAMB3 PE=1 SV=1 -	2.82	0.94	1	1	1	1	2.309E7	1172	129.5	7.21
O94992	Protein HEXIM1 OS=Homo sapiens GN=HEXIM1 PE=1 SV=1 - [HEXI	2.78	4.74	1	1	1	1	4.140E7	359	40.6	4.89
P62266	40S ribosomal protein S23 OS=Homo sapiens GN=RPS23 PE=1 SV=	2.77	7.69	1	1	1	1	8.552E6	143	15.8	10.49
Q13115	Dual specificity protein phosphatase 4 OS=Homo sapiens GN=DUSP	2.77	6.85	1	2	2	2	3.252E7	394	42.9	7.36
M0R2P8	PIH1 domain-containing protein 1 (Fragment) OS=Homo sapiens GN	2.76	26.32	6	2	2	2	5.149E7	114	13.2	5.26
I3L2R3	Nuclear distribution protein nudE homolog 1 (Fragment) OS=Homo s	2.75	8.76	10	1	1	1	1.030E7	137	14.2	9.69
P22492	Histone H1t OS=Homo sapiens GN=HIST1H1T PE=2 SV=4 - [H1T_H	2.74	5.31	5	1	1	1	2.110E7	207	22.0	11.71
F2Z2Y4	Pyridoxal kinase OS=Homo sapiens GN=PDXK PE=1 SV=1 - [F2Z2Y4	2.72	6.25	2	1	1	1	3.942E7	272	30.6	6.65
M0QYK9	DNA-binding death effector domain-containing protein 2 (Fragment)	2.71	9.40	3	1	1	1	9.135E6	117	13.2	5.52
P56589	Peroxisomal biogenesis factor 3 OS=Homo sapiens GN=PEX3 PE=1 S	2.71	2.68	1	1	1	1	2.791E7	373	42.1	8.15
A0A087WWT3	Serum albumin OS=Homo sapiens GN=ALB PE=1 SV=1 - [A0A087W	2.71	3.79	7	1	1	1	1.103E8	396	45.1	6.10
F2Z3J9	Prostaglandin reductase 1 (Fragment) OS=Homo sapiens GN=PTGR1	2.67	8.47	2	1	1	1	8.658E6	118	13.4	5.55
Q99626	Homeobox protein CDX-2 OS=Homo sapiens GN=CDX2 PE=1 SV=3	2.67	6.07	68	1	2	2	2.799E7	313	33.5	9.63
Q5JPU2	Pyruvate dehydrogenase E1 component subunit alpha, somatic form	2.67	10.74	5	1	1	1	4.724E7	121	13.8	10.18
H3BSQ0	Enhancer of mRNA-decapping protein 3 (Fragment) OS=Homo sapie	2.67	7.36	3	1	1	1	3.166E7	163	18.5	6.05
H3BNC1	RNA-binding motif protein, X chromosome OS=Homo sapiens GN=R	2.66	35.14	7	1	1	1	5.325E7	37	4.0	5.11
H7C1W2	Isocitrate dehydrogenase [NAD] subunit gamma, mitochondrial (Fra	2.64	5.03	5	1	1	1	1.780E7	199	22.4	8.18
F5H8H2	Mevalonate kinase OS=Homo sapiens GN=MVK PE=1 SV=1 - [F5H8	2.64	3.49	3	1	1	1	1.061E7	344	37.1	7.08
A0A0C4DGC5	Prelamin-A/C (Fragment) OS=Homo sapiens GN=LMNA PE=1 SV=1	2.63	5.38	3	1	1	1	1.911E7	260	27.5	9.69
A0A1W2PQ16	Cyclin-T1 OS=Homo sapiens GN=CCNT1 PE=1 SV=1 - [A0A1W2PQ1	2.63	6.52	2	1	1	1	1.586E7	184	21.2	7.18
Q96DP5	Methionyl-tRNA formyltransferase, mitochondrial OS=Homo sapiens	2.61	4.11	1	1	1	1	3.472E7	389	43.8	9.66
Q5TBH0	Serine/threonine-protein kinase Nek6 (Fragment) OS=Homo sapiens	2.61	4.00	7	1	1	1	1.439E7	200	22.6	7.03
J3KSR8	Serine/arginine-rich-splicing factor 1 (Fragment) OS=Homo sapiens	2.58	6.99	3	1	1	1	3.614E7	143	16.4	11.44
Q96J88	Epithelial-stromal interaction protein 1 OS=Homo sapiens GN=EPSTI	2.57	3.46	1	1	1	1	1.652E7	318	36.8	9.89
O76003	Glutaredoxin-3 OS=Homo sapiens GN=GLRX3 PE=1 SV=2 - [GLRX3_	2.57	5.07	1	1	1	1	7.928E6	335	37.4	5.39
Q92747	Actin-related protein 2/3 complex subunit 1A OS=Homo sapiens GN=	2.56	2.70	1	1	1	1		370	41.5	8.18
E9PH82	Protein FAM98A OS=Homo sapiens GN=FAM98A PE=1 SV=1 - [E9PH	2.56	3.85	2	1	1	1	5.330E6	312	34.4	8.28
Q5T8U3	60S ribosomal protein L7a (Fragment) OS=Homo sapiens GN=RPL7A	2.55	24.61	3	4	4	4	1.821E7	191	21.5	11.02
Q9BRL6	Serine/arginine-rich splicing factor 8 OS=Homo sapiens GN=SRSF8 F	2.54	8.87	1	1	2	2	1.605E8	282	32.3	11.72
B9A035	Cytosolic 5'-nucleotidase 3A OS=Homo sapiens GN=NT5C3A PE=1 S	2.50	5.85	3	1	1	1	1.095E7	205	23.5	7.75
Q8IYB3	Serine/arginine repetitive matrix protein 1 OS=Homo sapiens GN=SF	2.50	3.76	2	1	1	1		904	102.3	11.84
C9J2I1	Armadillo repeat-containing protein 8 (Fragment) OS=Homo sapiens	2.49	5.40	5	1	1	1	2.387E7	352	39.9	7.85
Q9UJX0	Oxidative stress-induced growth inhibitor 1 OS=Homo sapiens GN=(	2.45	2.68	1	1	1	1	1.020E7	560	60.8	7.42
E9PHA9	E3 ubiquitin-protein ligase CBL-B OS=Homo sapiens GN=CBLB PE=1	2.45	6.60	3	1	1	1	1.781E7	197	21.4	8.18
A0A1B0GW95	Protein IWS1 homolog (Fragment) OS=Homo sapiens GN=IWS1 PE=	2.44	6.76	2	1	1	1	6.508E7	207	24.0	10.35
G3V4X8	SNW domain-containing protein 1 OS=Homo sapiens GN=SNW1 PE=	2.44	2.67	3	1	1	1	1.858E7	374	43.3	9.70
O14828	Secretory carrier-associated membrane protein 3 OS=Homo sapiens	2.42	16.43	1	3	3	3	4.444E7	347	38.3	7.64
Q9BX95	Sphingosine-1-phosphate phosphatase 1 OS=Homo sapiens GN=SGI	2.40	2.95	1	1	1	1	8.976E6	441	49.1	8.82
K7EJR8	SWI/SNF-related matrix-associated actin-dependent regulator of chr	2.40	6.52	3	1	1	1	2.154E7	184	20.5	10.05
K7EII7	Galactokinase (Fragment) OS=Homo sapiens GN=GALK1 PE=1 SV=1	2.39	11.96	2	1	1	1	2.629E7	184	20.1	7.06
D6RF48	Syntaxin-18 OS=Homo sapiens GN=STX18 PE=1 SV=1 - [D6RF48_H	2.38	2.60	2	1	1	1	1.115E7	308	35.4	5.55
B9A008	U3 small nucleolar ribonucleoprotein protein IMP4 (Fragment) OS=H	2.37	5.81	4	1	1	1	2.187E7	172	19.6	9.70
B1AP15	CD55 antigen, decay accelerating factor for complement (Cromer blc	2.37	7.57	5	1	1	1	4.136E7	317	34.0	6.38
Q14106	Protein Tob2 OS=Homo sapiens GN=TOB2 PE=1 SV=2 - [TOB2_HU	2.36	10.17	2	2	2	2	3.810E7	344	36.6	6.93
P26196	Probable ATP-dependent RNA helicase DDX6 OS=Homo sapiens GN=	2.36	3.11	1	1	1	1	1.890E7	483	54.4	8.66
Q9BVG4	Protein PBDC1 OS=Homo sapiens GN=PBDC1 PE=1 SV=1 - [PBDC1_	2.35	4.29	1	1	1	1	1.993E7	233	26.0	4.79

S4R3W8	Calcium uniporter protein, mitochondrial OS=Homo sapiens GN=MCU	2.35	16.67	4	1	1	1	1.308E7	54	5.3	11.82
Q5T911	Mediator of RNA polymerase II transcription subunit 4 (Fragment) O	2.34	5.88	2	1	1	1	3.093E7	238	27.1	5.63
H0Y2V1	Microtubule-associated protein (Fragment) OS=Homo sapiens GN=M	2.34	3.67	6	1	1	1	2.490E7	463	48.6	10.11
Q9Y5P6	Mannose-1-phosphate guanyltransferase beta OS=Homo sapiens GN	2.34	3.06	1	1	1	1	4.072E7	360	39.8	6.61
H7BY16	Nucleolin (Fragment) OS=Homo sapiens GN=NCL PE=1 SV=8 - [H7B	2.33	10.81	2	2	2	2	3.297E7	296	32.4	5.36
Q5TDE7	E3 ubiquitin-protein ligase RNF220 (Fragment) OS=Homo sapiens GI	2.32	5.41	3	1	1	1	2.102E7	259	29.3	5.53
Q8WWH5	Probable tRNA pseudouridine synthase 1 OS=Homo sapiens GN=TRU	2.32	3.15	1	1	1	1	1.722E7	349	37.2	8.25
Q13303	Voltage-gated potassium channel subunit beta-2 OS=Homo sapiens	2.31	2.72	1	1	1	1	9.131E6	367	41.0	9.00
O00625	Pirin OS=Homo sapiens GN=PIR PE=1 SV=1 - [PIR_HUMAN]	2.30	3.10	1	1	1	1	1.379E7	290	32.1	6.92
A6NHN2	Roquin-2 OS=Homo sapiens GN=RC3H2 PE=1 SV=2 - [A6NHN2_HU	2.30	2.77	3	1	1	1	4.623E6	506	56.8	7.69
K7ELB8	Pleckstrin homology domain-containing family F member 1 (Fragmer	2.30	17.19	2	1	1	1	9.313E6	128	14.5	9.13
Q9BWD1	Acetyl-CoA acetyltransferase, cytosolic OS=Homo sapiens GN=ACAT	2.29	2.77	1	1	1	1	1.943E7	397	41.3	6.92
Q15628	Tumor necrosis factor receptor type 1-associated DEATH domain pro	2.29	3.21	1	1	1	1	1.128E7	312	34.2	6.27
D6R918	OCIA domain-containing protein 1 (Fragment) OS=Homo sapiens GN	2.27	24.66	8	1	1	1	4.550E7	73	8.3	5.83
E9PJD9	60S ribosomal protein L27a OS=Homo sapiens GN=RPL27A PE=1 SV	2.27	12.09	4	1	1	1	9.104E6	91	10.1	10.21
P30419	Glycylpeptide N-tetradecanoyltransferase 1 OS=Homo sapiens GN=N	2.27	2.22	1	1	1	1	2.805E7	496	56.8	7.80
Q9UHC7	E3 ubiquitin-protein ligase makorin-1 OS=Homo sapiens GN=MKRN1	2.25	2.07	2	1	1	1	7.826E6	482	53.3	5.14
I3L387	Serine/threonine-protein kinase PLK1 OS=Homo sapiens GN=PLK1 P	2.24	10.98	2	1	1	1	1.888E7	173	18.7	8.92
Q15327	Ankyrin repeat domain-containing protein 1 OS=Homo sapiens GN=A	2.24	3.76	1	1	1	1	2.515E7	319	36.2	7.50
E5RIL5	F-box only protein 16 (Fragment) OS=Homo sapiens GN=FBXO16 PE	2.24	5.98	4	1	1	1	4.616E7	184	21.3	9.66
Q92604	Acyl-CoA:lysophosphatidylglycerol acyltransferase 1 OS=Homo sapie	2.24	8.65	1	2	2	2	2.036E7	370	43.1	8.92
Q8TAP8	Protein phosphatase 1 regulatory subunit 35 OS=Homo sapiens GN=	2.22	7.11	1	1	1	2	1.708E7	253	27.9	8.68
A0A0D9SEU5	Ran-binding protein 10 OS=Homo sapiens GN=RANBP10 PE=1 SV=1	2.22	1.77	3	1	1	1	1.406E7	620	67.2	6.84
E9PIZ4	Cysteine and histidine-rich domain-containing protein 1 OS=Homo sa	2.22	13.79	4	1	1	1	2.261E7	116	13.1	8.29
P13639	Elongation factor 2 OS=Homo sapiens GN=EEF2 PE=1 SV=4 - [EF2_	2.21	3.38	1	2	2	2	1.829E7	858	95.3	6.83
H0YJH7	Lamina-associated polypeptide 2, isoforms beta/gamma (Fragment)	2.21	13.50	4	2	2	2	1.443E7	237	26.9	9.31
A0A0M3HER1	LIM and senescent cell antigen-like-containing domain protein 1 (Fra	2.21	5.74	3	1	1	1	2.491E7	209	24.4	9.00
H0YJN9	Legumain (Fragment) OS=Homo sapiens GN=LGMN PE=1 SV=1 - [H	2.19	18.68	4	1	1	1	3.423E7	91	9.8	8.51
F8WE04	Heat shock protein beta-1 OS=Homo sapiens GN=HSPB1 PE=1 SV=	2.19	5.38	2	1	1	1	3.363E7	186	20.4	9.06
O14556	Glyceraldehyde-3-phosphate dehydrogenase, testis-specific OS=Hom	2.18	2.70	1	1	1	1	1.710E7	408	44.5	8.19
P61026	Ras-related protein Rab-10 OS=Homo sapiens GN=RAB10 PE=1 SV=	2.18	5.50	1	1	1	1	4.797E6	200	22.5	8.38
Q9UDT1	Rhomboid domain-containing protein 2 OS=Homo sapiens GN=WUG	2.17	20.25	2	1	1	1	5.602E7	79	8.4	7.87
Q9UHR4	Brain-specific angiogenesis inhibitor 1-associated protein 2-like prote	2.17	2.15	1	1	1	1		511	56.8	8.68
G3V1C1	1,5-anhydro-D-fructose reductase OS=Homo sapiens GN=AKR1E2 PE	2.16	3.94	3	1	1	1	2.270E7	203	23.1	6.20
E7EX54	Mitogen-activated protein kinase 14 (Fragment) OS=Homo sapiens G	2.16	17.42	28	2	2	2	1.331E7	132	15.4	5.68
Q8TBM8	DnaJ homolog subfamily B member 14 OS=Homo sapiens GN=DNAJ	2.15	2.90	1	1	1	1	5.025E7	379	42.5	8.59
P14091	Cathepsin E OS=Homo sapiens GN=CTSE PE=1 SV=2 - [CATE_HUM	2.15	2.00	1	1	1	1	1.093E8	401	43.3	4.86
F8WFC6	Large subunit GTPase 1 homolog OS=Homo sapiens GN=LSG1 PE=1	2.15	6.18	2	1	1	1	9.522E6	178	21.0	8.78
A0A0A0MSI0	Peroxiredoxin-1 (Fragment) OS=Homo sapiens GN=PRDX1 PE=1 SV	2.14	12.87	5	2	2	2	1.697E7	171	19.0	6.92
Q5T2N8	ATPase family AAA domain-containing protein 3C OS=Homo sapiens	2.14	1.95	4	1	1	1	9.257E6	411	46.4	9.31
Q9H871	Protein RMD5 homolog A OS=Homo sapiens GN=RMND5A PE=1 SV=	2.14	3.32	1	1	1	1	1.157E7	391	44.0	6.06
Q5D862	Filaggrin-2 OS=Homo sapiens GN=FLG2 PE=1 SV=1 - [FILA2_HUMA	2.13	0.46	1	1	1	1	1.150E7	2391	247.9	8.31
I3L1Q5	Pre-rRNA-processing protein TSR1 homolog OS=Homo sapiens GN=T	2.13	7.88	2	2	2	2	1.910E7	368	41.4	9.50
A2A2L6	Protein RTF2 homolog OS=Homo sapiens GN=RTFDC1 PE=1 SV=1 -	2.13	4.35	4	1	1	1	1.072E7	230	26.1	9.07
Q9NUD5	Zinc finger CCHC domain-containing protein 3 OS=Homo sapiens GN	2.13	7.43	3	4	4	4	1.017E7	404	43.6	8.53
P23610	Factor VIII intron 22 protein OS=Homo sapiens GN=F8A1 PE=1 SV=	2.11	3.50	1	1	1	1	1.258E7	371	39.1	6.84
Q13177	Serine/threonine-protein kinase PAK 2 OS=Homo sapiens GN=PAK2	2.11	1.72	3	1	1	1	1.313E7	524	58.0	5.96
C9JUG1	5'-AMP-activated protein kinase subunit gamma-2 (Fragment) OS=H	2.11	6.75	5	1	1	1	1.086E7	163	19.0	7.05
Q9Y512	Sorting and assembly machinery component 50 homolog OS=Homo	2.11	2.35	1	1	1	1	2.035E7	469	51.9	6.90
Q7Z7A3	Cytoplasmic tRNA 2-thiolation protein 1 OS=Homo sapiens GN=CTU	2.11	6.03	1	1	1	1	1.679E7	348	36.4	9.20
H7BY94	Bystin (Fragment) OS=Homo sapiens GN=BYSL PE=1 SV=1 - [H7BY	2.10	4.92	2	1	1	1	8.573E6	183	21.0	9.25
Q3SXM5	Inactive hydroxysteroid dehydrogenase-like protein 1 OS=Homo sap	2.10	4.85	3	2	2	2	1.805E7	330	37.0	8.72
Q96S59	Ran-binding protein 9 OS=Homo sapiens GN=RANBP9 PE=1 SV=1 -	2.10	1.92	1	1	1	1	4.500E7	729	77.8	6.79

Q8WXF1	Paraspeckle component 1 OS=Homo sapiens GN=PSPC1 PE=1 SV=1	2.10	2.68	1	1	1	1	1.979E7	523	58.7	6.67
K7EP82	Regulation of nuclear pre-mRNA domain-containing protein 1A (Frag	2.10	14.63	3	1	1	1	6.936E6	123	13.7	4.86
P05109	Protein S100-A8 OS=Homo sapiens GN=S100A8 PE=1 SV=1 - [S10A	2.09	11.83	1	1	1	1	2.161E7	93	10.8	7.03
P78318	Immunoglobulin-binding protein 1 OS=Homo sapiens GN=IGBP1 PE	2.08	2.65	1	1	1	1	2.591E7	339	39.2	5.38
E9PPT8	Coiled-coil domain-containing protein 84 OS=Homo sapiens GN=CCD	2.07	6.76	3	1	1	1	1.960E7	148	17.2	9.13
Q5T0Z8	Uncharacterized protein C6orf132 OS=Homo sapiens GN=C6orf132 F	2.06	1.18	1	1	1	2	3.804E7	1188	124.0	9.45
Q96QD9	UAP56-interacting factor OS=Homo sapiens GN=FYTTD1 PE=1 SV=3	2.06	5.03	2	2	2	2	1.796E7	318	35.8	11.78
H0YJE9	26S proteasome regulatory subunit 10B OS=Homo sapiens GN=PSM	2.06	20.00	5	1	1	1	1.081E7	70	8.3	6.11
C9IYF5	Exostosin-like 2 (Fragment) OS=Homo sapiens GN=EXTL2 PE=1 SV=	2.03	9.40	4	1	1	1	1.208E7	117	13.2	9.99
A0A1W2PPZ5	Transcription elongation factor A protein 1 OS=Homo sapiens GN=T	2.02	16.28	13	4	4	4	2.077E7	301	33.9	8.38
M0R248	Delta(3,5)-Delta(2,4)-dienoyl-CoA isomerase, mitochondrial (Fragme	2.02	2.62	2	1	1	1	9.069E6	267	29.2	8.00
Q9UEW8	STE20/SPS1-related proline-alanine-rich protein kinase OS=Homo sa	2.01	8.81	3	3	3	3	3.451E7	545	59.4	6.29
H3BQC6	Ubiquitin carboxyl-terminal hydrolase 10 (Fragment) OS=Homo sapi	2.01	12.06	2	1	1	1	1.019E7	141	15.1	4.21
P13861	cAMP-dependent protein kinase type II-alpha regulatory subunit OS=	2.01	5.20	3	2	2	2	8.288E6	404	45.5	5.07
P29279	Connective tissue growth factor OS=Homo sapiens GN=CTGF PE=1	2.01	3.72	1	1	1	1	2.272E7	349	38.1	8.00
Q9BRQ8	Apoptosis-inducing factor 2 OS=Homo sapiens GN=AIFM2 PE=1 SV=	2.01	3.49	1	1	1	1	1.274E7	373	40.5	9.11
Q7LG56	Ribonucleoside-diphosphate reductase subunit M2 B OS=Homo sapie	2.00	3.70	2	1	1	1	2.086E7	351	40.7	4.97
Q5T457	Ankyrin repeat domain-containing protein 2 OS=Homo sapiens GN=A	1.99	5.00	3	1	1	1	1.403E7	300	33.4	6.02
H7BXR2	Phosphatidylinositol 3,4,5-trisphosphate 5-phosphatase 2 (Fragment	1.99	9.40	3	1	1	1		149	16.5	5.06
K7EPP7	DnaJ homolog subfamily C member 7 OS=Homo sapiens GN=DNAJC	1.99	3.95	4	1	1	1	3.689E6	253	28.7	8.48
A0A1W2PRW5	Adaptin ear-binding coat-associated protein 1 (Fragment) OS=Homo	1.98	15.05	8	1	1	1	1.461E7	93	9.3	9.41
B5MCP9	40S ribosomal protein S7 OS=Homo sapiens GN=RPS7 PE=1 SV=1 -	1.98	6.42	2	1	1	1	4.826E6	187	21.3	10.27
Q6DCA0	AMMECR1-like protein OS=Homo sapiens GN=AMMECR1L PE=1 SV=	1.97	3.23	2	1	1	1	1.637E7	310	34.5	8.98
P35548	Homeobox protein MSX-2 OS=Homo sapiens GN=MSX2 PE=1 SV=3	1.97	5.24	69	1	2	2	2.525E7	267	28.9	9.67
Q96PK6	RNA-binding protein 14 OS=Homo sapiens GN=RBM14 PE=1 SV=2 -	1.96	2.24	1	1	1	1	1.487E7	669	69.4	9.67
H7C547	Sodium/potassium-transporting ATPase subunit beta-3 (Fragment) O	1.96	32.56	3	1	1	1	4.723E7	43	4.9	9.77
F2Z2A4	Deoxynucleotidyltransferase terminal-interacting protein 1 (Fragmen	1.96	17.93	5	2	2	2	5.255E7	145	16.4	9.61
H7C5D5	Procollagen C-endopeptidase enhancer 2 (Fragment) OS=Homo sapi	1.96	4.29	5	1	1	1	2.057E7	140	15.3	9.52
J3QKY4	Proline-rich protein 11 (Fragment) OS=Homo sapiens GN=PRR11 PE	1.95	9.03	3	1	1	1	2.077E7	144	16.8	10.29
F5GY15	BTB/POZ domain-containing adapter for CUL3-mediated RhoA degra	1.95	6.73	8	1	1	1	2.478E7	104	11.7	5.92
Q6P0N6	DST protein OS=Homo sapiens GN=DST PE=1 SV=1 - [Q6P0N6_HU	1.94	1.05	6	1	1	1	2.483E8	1143	132.2	5.48
H0Y894	Mitogen-activated protein kinase kinase kinase 6 (Fragment) OS=Ho	1.94	0.88	2	1	1	1	4.131E7	798	88.7	6.83
B4DLR8	NAD(P)H dehydrogenase [quinone] 1 OS=Homo sapiens GN=NQO1	1.93	4.95	3	1	1	1	1.525E7	202	22.8	8.50
B7Z8R2	Pentatricopeptide repeat-containing protein 2, mitochondrial OS=Ho	1.93	4.35	2	1	1	1	2.083E7	161	18.3	9.17
Q5T712	Monoacylglycerol lipase ABHD12 (Fragment) OS=Homo sapiens GN=	1.93	4.28	2	1	1	1	1.060E7	187	21.2	8.25
F5H282	T-complex protein 1 subunit alpha OS=Homo sapiens GN=TCP1 PE=	1.92	3.31	3	1	1	1	7.713E6	332	36.4	7.02
Q9BZE1	39S ribosomal protein L37, mitochondrial OS=Homo sapiens GN=MR	1.91	1.89	2	1	1	1	7.601E6	423	48.1	8.59
Q8WVY7	Ubiquitin-like domain-containing CTD phosphatase 1 OS=Homo sapi	1.91	4.40	1	1	1	1	6.179E6	318	36.8	6.46
Q1W6H9	Protein FAM110C OS=Homo sapiens GN=FAM110C PE=1 SV=2 - [F1	1.89	5.30	1	1	1	1	2.036E7	321	33.8	9.91
O94907	Dickkopf-related protein 1 OS=Homo sapiens GN=DKK1 PE=1 SV=1	1.88	3.76	1	1	1	1	7.460E6	266	28.7	8.40
E5RJG9	PIN2/TERF1-interacting telomerase inhibitor 1 OS=Homo sapiens GN	1.88	10.45	4	1	1	1	1.037E7	134	15.4	8.97
E9PNW8	Fatty acyl-CoA reductase (Fragment) OS=Homo sapiens GN=FAR1 P	1.88	3.88	2	1	1	1	1.004E7	335	37.5	6.92
E9PEK1	TRAF family member-associated NF-kappa-B activator OS=Homo sap	1.87	22.22	11	1	1	1	1.053E9	36	4.2	6.54
Q96PG2	Membrane-spanning 4-domains subfamily A member 10 OS=Homo s	1.86	3.37	1	1	1	1	2.639E7	267	29.7	8.95
H0YLA4	Sorbitol dehydrogenase OS=Homo sapiens GN=SORD PE=1 SV=1 -	1.85	8.04	2	2	2	2	6.664E6	336	36.2	7.81
Q08211	ATP-dependent RNA helicase A OS=Homo sapiens GN=DHX9 PE=1 S	1.83	0.63	1	1	1	1	3.003E7	1270	140.9	6.84
Q9Y5W3	Krueppel-like factor 2 OS=Homo sapiens GN=KLF2 PE=1 SV=2 - [KL	1.83	2.25	1	1	1	1	6.996E6	355	37.4	8.79
E9PN19	Galectin OS=Homo sapiens GN=LGALS8 PE=1 SV=1 - [E9PN19_HUN	1.82	6.55	6	2	2	2	1.684E7	290	32.6	7.23
C9JBY0	Bromodomain-containing protein 9 (Fragment) OS=Homo sapiens GI	1.82	3.11	2	1	1	1	6.826E7	225	25.5	6.54
P50995	Annexin A11 OS=Homo sapiens GN=ANXA11 PE=1 SV=1 - [ANX11_	1.81	3.17	1	1	1	1		505	54.4	7.65
E7EU85	Fragile X mental retardation syndrome-related protein 1 OS=Homo s	1.80	1.76	9	1	1	1	4.534E7	454	51.0	7.27
Q9UJK0	Ribosome biogenesis protein TSR3 homolog OS=Homo sapiens GN=	1.80	10.58	1	3	3	3	1.633E7	312	33.6	6.87
D6RHI7	Cyclin-H OS=Homo sapiens GN=CCNH PE=1 SV=1 - [D6RHI7_HUMA	1.79	3.21	3	1	1	1	8.558E6	249	28.9	5.92

K7ERU8	Deoxyhypusine hydroxylase (Fragment) OS=Homo sapiens GN=DOH	1.78	16.92	3	2	2	2	7.822E6	195	20.8	4.70
Q86UE4	Protein LYRIC OS=Homo sapiens GN=MTDH PE=1 SV=2 - [LYRIC_H	1.78	2.75	1	1	1	1	2.371E7	582	63.8	9.32
A2RQR6	Transcription elongation factor A (SII)-like 4 variant 1 OS=Homo sap	1.77	4.84	3	1	1	1	1.427E7	186	21.5	5.86
Q8IZV5	Retinol dehydrogenase 10 OS=Homo sapiens GN=RDH10 PE=1 SV=	1.76	2.35	1	1	1	1	8.505E6	341	38.1	7.40
K7EJC1	26S proteasome non-ATPase regulatory subunit 8 OS=Homo sapiens	1.76	4.65	4	1	1	1	9.674E6	172	19.8	6.93
P11766	Alcohol dehydrogenase class-3 OS=Homo sapiens GN=ADH5 PE=1 S	1.75	2.14	1	1	1	1	5.282E6	374	39.7	7.49
D6RIU4	Vesicular integral-membrane protein VIP36 (Fragment) OS=Homo sa	1.73	5.24	4	1	1	1	2.313E7	191	21.8	6.52
H3BLU7	Aflatoxin B1 aldehyde reductase member 2 (Fragment) OS=Homo sa	1.73	6.05	2	1	1	1	1.404E7	314	34.7	7.18
A0A0A0MRK6	Metaxin 1, isoform CRA_b OS=Homo sapiens GN=MTX1 PE=1 SV=1	1.73	3.00	2	1	1	1	2.276E7	466	51.4	9.79
M0QZR0	Coiled-coil domain-containing protein 9 (Fragment) OS=Homo sapie	1.70	21.43	3	1	1	1	7.830E6	56	6.5	6.77
Q3MIR4	Cell cycle control protein 50B OS=Homo sapiens GN=TMEM30B PE=	1.70	2.28	2	1	1	1	1.225E7	351	38.9	7.88
Q9H649	tRNA (cytosine(34)-C(5))-methyltransferase, mitochondrial OS=Hom	1.69	3.24	1	1	1	1		340	38.2	8.87
H0YBM4	Arf-GAP with SH3 domain, ANK repeat and PH domain-containing pr	1.69	2.69	4	1	1	1		483	52.9	6.02
Q9UDY4	DnaJ homolog subfamily B member 4 OS=Homo sapiens GN=DNAJB	1.67	12.76	2	2	4	4	2.991E7	337	37.8	8.50
Q3KP66	Uncharacterized protein C1orf106 OS=Homo sapiens GN=C1orf106 F	1.67	2.11	1	1	1	1	2.590E7	663	72.9	9.31
P78356	Phosphatidylinositol 5-phosphate 4-kinase type-2 beta OS=Homo sa	1.67	5.53	5	2	2	2	1.224E7	416	47.3	7.33
Q9P258	Protein RCC2 OS=Homo sapiens GN=RCC2 PE=1 SV=2 - [RCC2_HU	1.67	6.32	1	3	3	3	5.366E6	522	56.0	8.78
Q92526	T-complex protein 1 subunit zeta-2 OS=Homo sapiens GN=CCT6B P	1.67	2.26	1	1	1	1	8.209E6	530	57.8	7.24
E7EM64	COP9 signalosome complex subunit 6 OS=Homo sapiens GN=COPS6	1.66	6.75	2	2	2	2	2.587E7	326	36.0	5.73
Q86YZ3	Hornerin OS=Homo sapiens GN=HRNR PE=1 SV=2 - [HORN_HUMA	1.66	1.23	1	1	1	1		2850	282.2	10.04
C9JCN8	Fos-related antigen 2 (Fragment) OS=Homo sapiens GN=FOSL2 PE=	1.65	8.91	2	1	1	1	1.516E7	202	22.2	8.78
Q96CM3	RNA pseudouridylate synthase domain-containing protein 4 OS=Hon	1.65	13.00	1	3	3	3	2.078E7	377	42.2	9.88
Q5LJB0	Ubiquitin carboxyl-terminal hydrolase (Fragment) OS=Homo sapiens	1.64	13.18	5	3	3	3	1.230E7	258	29.1	5.03
A8MUF7	Hemoglobin subunit epsilon (Fragment) OS=Homo sapiens GN=HBE	1.63	11.49	10	1	1	1	1.377E7	87	9.5	9.13
Q6PKG0	La-related protein 1 OS=Homo sapiens GN=LARP1 PE=1 SV=2 - [LA	1.62	0.55	1	1	1	1	8.524E6	1096	123.4	8.82
A0A075B6F6	Minor histocompatibility antigen H13 (Fragment) OS=Homo sapiens	1.62	6.99	2	2	2	2	1.336E7	272	30.1	7.88
A0A0C4DGA2	Enoyl-CoA delta isomerase 2, mitochondrial OS=Homo sapiens GN=I	1.62	1.92	2	1	1	1	6.288E6	364	40.2	8.76
B1AK81	GPI-anchor transamidase OS=Homo sapiens GN=PIGK PE=1 SV=1 -	1.61	3.99	3	1	1	1	2.249E7	301	34.3	5.81
Q9BQA1	Methylosome protein 50 OS=Homo sapiens GN=WDR77 PE=1 SV=1	1.61	3.51	1	1	1	1	2.788E7	342	36.7	5.17
E5RGE1	14-3-3 protein zeta/delta (Fragment) OS=Homo sapiens GN=YWHAZ	1.60	15.69	22	1	1	1	1.260E7	51	5.8	4.78
Q8N4T4	Rho guanine nucleotide exchange factor 39 OS=Homo sapiens GN=/	0.00	6.87	1	1	1	1	1.719E7	335	38.3	9.64
O43150	Arf-GAP with SH3 domain, ANK repeat and PH domain-containing pr	0.00	0.80	1	1	1	1		1006	111.6	6.68
Q5U4P2	Aspartate beta-hydroxylase domain-containing protein 1 OS=Homo s	0.00	6.67	1	1	1	1	5.762E7	390	41.1	8.56
Q6ZUV0	Putative cytosolic acyl coenzyme A thioester hydrolase-like OS=Hom	0.00	6.35	1	1	1	1		252	28.1	7.15
Q9UNE7	E3 ubiquitin-protein ligase CHIP OS=Homo sapiens GN=STUB1 PE=1	0.00	3.63	1	1	1	1	5.423E7	303	34.8	5.87
P01024	Complement C3 OS=Homo sapiens GN=C3 PE=1 SV=2 - [CO3_HUM	0.00	1.20	1	1	1	2	5.365E7	1663	187.0	6.40
Q9H1X3	DnaJ homolog subfamily C member 25 OS=Homo sapiens GN=DNAJ	0.00	7.22	1	1	1	1	1.908E7	360	42.4	9.13
O75319	RNA/RNP complex-1-interacting phosphatase OS=Homo sapiens GN=	0.00	10.61	3	2	2	3	1.917E7	330	38.9	9.41
P26641	Elongation factor 1-gamma OS=Homo sapiens GN=EEF1G PE=1 SV=	0.00	2.29	1	1	1	1	5.691E6	437	50.1	6.67
P12259	Coagulation factor V OS=Homo sapiens GN=F5 PE=1 SV=4 - [FA5_H	0.00	0.58	2	1	1	1	1.987E7	2224	251.5	6.05
Q6UN15	Pre-mRNA 3'-end-processing factor FIP1 OS=Homo sapiens GN=FIP1	0.00	2.19	1	1	1	1	1.199E7	594	66.5	5.59
Q96I24	Far upstream element-binding protein 3 OS=Homo sapiens GN=FUB	0.00	1.57	3	1	1	1	6.251E6	572	61.6	8.38
Q9UN86	Ras GTPase-activating protein-binding protein 2 OS=Homo sapiens (	0.00	3.53	1	1	1	1	4.574E7	482	54.1	5.55
Q8TDV5	Glucose-dependent insulinotropic receptor OS=Homo sapiens GN=G	0.00	4.18	1	1	1	1	1.384E7	335	36.9	8.82
Q9P0J7	E3 ubiquitin-protein ligase KCMF1 OS=Homo sapiens GN=KCMF1 PE	0.00	4.72	1	1	1	1	2.816E6	381	41.9	5.66
Q6PID8	Kelch domain-containing protein 10 OS=Homo sapiens GN=KLHDC1(	0.00	5.43	1	2	2	2	2.018E7	442	49.1	9.38
A6PVS8	Leucine-rich repeat and IQ domain-containing protein 3 OS=Homo s	0.00	2.72	1	1	1	1	2.043E8	624	73.6	9.72
Q9NU22	Midasin OS=Homo sapiens GN=MDN1 PE=1 SV=2 - [MDN1_HUMAN	0.00	0.16	1	1	1	1	8.458E7	5596	632.4	5.68
Q9H4K7	Mitochondrial ribosome-associated GTPase 2 OS=Homo sapiens GN=	0.00	3.94	2	2	2	2	8.797E6	406	43.9	9.45
Q9BYG5	Partitioning defective 6 homolog beta OS=Homo sapiens GN=PARD6	0.00	3.23	1	1	1	1	7.226E6	372	41.2	5.58
Q08752	Peptidyl-prolyl cis-trans isomerase D OS=Homo sapiens GN=PPID PE	0.00	3.24	1	1	1	1	8.297E6	370	40.7	7.21
Q5T8P6	RNA-binding protein 26 OS=Homo sapiens GN=RBM26 PE=1 SV=3 -	0.00	0.79	2	1	1	1	1.041E7	1007	113.5	9.16
Q86XZ4	Spermatogenesis-associated serine-rich protein 2 OS=Homo sapiens	0.00	2.20	1	1	1	1	1.905E7	545	59.5	8.90

P41250	Glycine--tRNA ligase OS=Homo sapiens GN=GARS PE=1 SV=3 - [GA	0.00	1.08	1	1	1	1		739	83.1	7.03
Q8WUA8	Tsukushin OS=Homo sapiens GN=TSKU PE=2 SV=3 - [TSK_HUMAN	0.00	4.25	1	1	1	1	6.674E6	353	37.8	6.87
P40222	Alpha-taxilin OS=Homo sapiens GN=TXLNA PE=1 SV=3 - [TXLNA_H	0.00	3.48	1	1	1	1	1.760E7	546	61.9	6.52
Q9Y2W2	WW domain-binding protein 11 OS=Homo sapiens GN=WBP11 PE=	0.00	2.18	1	1	1	1		641	70.0	8.38
P13010	X-ray repair cross-complementing protein 5 OS=Homo sapiens GN=	0.00	1.23	1	1	1	1	5.170E6	732	82.7	5.81
Q7L3S4	Zinc finger protein 771 OS=Homo sapiens GN=ZNF771 PE=1 SV=1	0.00	3.79	1	1	1	1	9.576E6	317	35.7	8.79
E7ETY4	Non-specific serine/threonine protein kinase OS=Homo sapiens GN=	0.00	2.43	3	1	1	1	7.670E6	699	78.4	9.54
G5E9U9	Poly (ADP-ribose) polymerase family, member 12, isoform CRA_a OS	0.00	2.86	2	1	1	1	7.477E5	420	47.5	8.56
J3KRL8	Pleckstrin homology domain-containing family M member 1 (Fragme	0.00	8.57	4	1	1	1	1.051E7	140	15.4	4.96
V9GYL1	Protein-L-isoaspartate O-methyltransferase domain-containing protei	0.00	9.57	5	1	1	1	6.441E6	94	10.5	4.46
Q5SQT6	Inorganic pyrophosphatase OS=Homo sapiens GN=PPA1 PE=1 SV=	0.00	5.06	2	1	1	1	2.537E7	178	20.0	5.25
A0A024R7W5	YTH domain family, member 3, isoform CRA_a OS=Homo sapiens G	0.00	4.31	6	2	2	2	1.684E7	534	58.3	9.23
M0R1N9	NAD-dependent protein deacetylase sirtuin-6 OS=Homo sapiens GN	0.00	7.39	2	1	1	1	6.201E6	176	19.1	7.69
Q5TBH9	Chromosome 1 open reading frame 131, isoform CRA_a OS=Homo s	0.00	4.82	3	1	1	1	1.036E7	249	27.9	9.54
A0A0A0MRE9	A-kinase anchor protein 9 OS=Homo sapiens GN=AKAP9 PE=1 SV=	0.00	0.80	3	1	1	1	1.593E7	3126	362.4	4.89
K7EM90	Alpha-enolase (Fragment) OS=Homo sapiens GN=ENO1 PE=1 SV=1	0.00	5.13	2	1	1	1	1.336E7	195	21.0	8.88
I3L4G9	Phosphoribosyl pyrophosphate synthase-associated protein 2 (Fragm	0.00	9.79	7	1	1	1	1.528E7	143	15.8	4.89
A0A087WU62	39S ribosomal protein L45, mitochondrial OS=Homo sapiens GN=MF	0.00	3.52	4	1	1	1	5.163E7	256	29.3	8.91
Q5SY74	Kinetochore-associated protein NSL1 homolog OS=Homo sapiens GN	0.00	8.72	2	1	1	1	1.037E7	172	19.5	4.98
A0A087WYC0	Golgin subfamily A member 2 OS=Homo sapiens GN=GOLGA2 PE=1	0.00	2.80	2	1	1	1	2.354E7	286	31.9	5.05
K7EJY5	Coiled-coil and C2 domain-containing protein 1A (Fragment) OS=Ho	0.00	2.91	2	1	1	1		378	39.2	9.26
H0YJR8	Dehydrogenase/reductase SDR family member 7 (Fragment) OS=Ho	0.00	6.60	4	1	1	1	8.986E6	212	23.5	8.90
C9JE01	WD repeat domain phosphoinositide-interacting protein 4 (Fragment	0.00	8.23	8	1	1	1	3.469E6	158	18.0	7.94
C9JDR0	Sterol-4-alpha-carboxylate 3-dehydrogenase, decarboxylating (Fragr	0.00	5.12	2	1	1	1	1.251E7	254	28.1	6.54
H0Y9D9	Cytoplasmic polyadenylation element-binding protein 2 (Fragment) (	0.00	9.58	8	1	1	1	8.186E6	167	18.6	7.24
E7EVK2	Mitochondrial GTPase 1 OS=Homo sapiens GN=MTG1 PE=1 SV=1 -	0.00	6.71	4	1	1	1	1.988E7	283	31.7	8.91
H0Y9T5	m7GpppN-mRNA hydrolase (Fragment) OS=Homo sapiens GN=DCP	0.00	3.98	2	2	2	2	1.745E7	402	44.8	9.47
M0QX52	Microtubule-associated protein RP/EB family member 2 (Fragment) (	0.00	9.45	4	1	1	1	1.024E7	127	14.9	7.87
C9J7M8	Transcription initiation factor TFIID subunit 8 OS=Homo sapiens GN	0.00	16.26	3	1	1	1		123	13.2	8.76
H0Y641	La-related protein 4B (Fragment) OS=Homo sapiens GN=LARP4B PE	0.00	4.21	3	1	1	1	8.387E7	190	20.4	8.27
E9PPH5	Acidic leucine-rich nuclear phosphoprotein 32 family member E (Frag	0.00	12.28	2	1	1	1	1.357E7	114	13.1	4.22
F5H2X8	5'-AMP-activated protein kinase subunit beta-1 OS=Homo sapiens G	0.00	25.00	4	2	2	2	7.540E6	104	11.7	7.91
C9J0E9	26S proteasome non-ATPase regulatory subunit 6 (Fragment) OS=H	0.00	5.75	2	1	1	1	5.188E6	226	26.6	5.47
F2Z3K5	Dolichyl-diphosphooligosaccharide--protein glycosyltransferase subu	0.00	7.41	4	1	1	1	6.042E6	162	17.8	5.15
E5RGJ2	ER membrane protein complex subunit 2 (Fragment) OS=Homo sapi	0.00	16.96	2	1	1	1	1.116E7	112	13.5	5.19
H0Y6D8	TATA-box-binding protein (Fragment) OS=Homo sapiens GN=TBP P	0.00	14.85	3	1	1	1	2.810E7	101	11.6	9.83
E9PLG2	26S proteasome regulatory subunit 6A (Fragment) OS=Homo sapien	0.00	8.15	6	1	1	1	3.441E7	184	20.5	5.07
Q5VU10	Ribonuclease P protein subunit p30 (Fragment) OS=Homo sapiens G	0.00	4.72	3	1	1	1	1.158E7	212	23.3	8.97
H0Y9K7	Splicing factor, proline- and glutamine-rich (Fragment) OS=Homo sa	0.00	6.28	2	1	1	1	1.489E7	223	26.4	5.16
A0A087WUJ2	Aspartyl/asparaginyl beta-hydroxylase (Fragment) OS=Homo sapien	0.00	3.23	6	1	1	1		186	21.3	4.35
H3BPL5	SAGA-associated factor 29 OS=Homo sapiens GN=SGF29 PE=1 SV=	0.00	9.85	2	1	1	1	8.733E6	132	14.6	6.07
H0YEU5	Histone-binding protein RBBP4 (Fragment) OS=Homo sapiens GN=R	0.00	4.79	8	1	1	1	4.994E6	167	19.0	5.53
M0QXY9	snRNA-activating protein complex subunit 2 (Fragment) OS=Homo s	0.00	11.47	2	1	1	1	6.980E7	279	29.5	9.66
E9PNF3	L-aminoadipate-semialdehyde dehydrogenase-phosphopantetheinyl	0.00	16.25	3	2	2	2	1.000E7	160	18.7	7.50
Q5W011	Splicing factor 45 (Fragment) OS=Homo sapiens GN=RBM17 PE=1 S	0.00	4.25	3	1	1	1	7.931E6	212	24.2	6.19
E9PPQ0	Mitochondrial fission regulator 1-like (Fragment) OS=Homo sapiens	0.00	22.64	7	1	1	1	6.158E6	53	5.8	9.70
H0YHZ5	GPN-loop GTPase 3 (Fragment) OS=Homo sapiens GN=GPN3 PE=1	0.00	15.00	2	1	1	1		120	13.8	4.61
Q5SZU1	D-3-phosphoglycerate dehydrogenase OS=Homo sapiens GN=PHGD	0.00	1.60	2	1	1	1	4.361E6	499	53.1	6.92
H7BYY4	L-seryl-tRNA(Sec) kinase (Fragment) OS=Homo sapiens GN=PSTK P	0.00	2.23	1	1	1	1	1.071E7	359	41.0	6.84
H7C5R5	Eukaryotic translation initiation factor 2A (Fragment) OS=Homo sapi	0.00	5.38	3	1	1	1	1.876E7	316	35.3	8.50
E9PNU1	Nucleoside diphosphate kinase 7 OS=Homo sapiens GN=NME7 PE=	0.00	3.52	3	1	1	1	1.650E7	256	28.9	8.09
A0A0A0MS89	Oral-facial-digital syndrome 1 protein OS=Homo sapiens GN=OFD1	0.00	2.07	3	1	1	1	1.264E7	338	40.2	5.86
F8VR84	UPF0160 protein MYG1, mitochondrial OS=Homo sapiens GN=C12or	0.00	4.69	3	1	1	1	1.399E8	213	24.0	5.39

A0A1B0GW11	Rho GTPase-activating protein 21 (Fragment) OS=Homo sapiens GN	0.00	18.02	1	1	1	1		172	19.3	5.63
E9PK89	Guanine nucleotide exchange factor for Rab-3A (Fragment) OS=Hom	0.00	4.94	2	1	1	1		263	28.5	5.83
E9PLA6	Serpin H1 (Fragment) OS=Homo sapiens GN=SERPINH1 PE=1 SV=8	0.00	30.59	10	1	1	1	1.888E7	85	8.8	5.24
G3V5L5	Protein arginine N-methyltransferase 5 (Fragment) OS=Homo sapien	0.00	5.33	4	1	1	1	7.353E6	169	19.6	6.89
K7EJP1	ATP synthase subunit alpha, mitochondrial (Fragment) OS=Homo sa	0.00	4.86	5	1	1	1		144	15.3	5.66
Q5JSB5	Transcription factor Dp-1 (Fragment) OS=Homo sapiens GN=TFDP1	0.00	6.38	2	1	1	1	7.952E6	235	26.0	9.66

AGS

Accession	Description	Score	Coverage	# Proteins	# Unique Peptides	# Peptides	# PSMs	Area	# AAs	MW [kDa]	calc. pI
Q02878	60S ribosomal protein L6 OS=Homo sapiens GN=RPL6 PE=1 SV=3 -	281.74	61.46	7	32	32	102	6.891E9	288	32.7	10.58
P05388	60S acidic ribosomal protein P0 OS=Homo sapiens GN=RPLP0 PE=1 S	251.65	64.35	13	19	19	86	9.801E9	317	34.3	5.97
P22626	Heterogeneous nuclear ribonucleoproteins A2/B1 OS=Homo sapiens (	235.77	67.71	2	26	31	97	2.264E9	353	37.4	8.95
F8W6I7	Heterogeneous nuclear ribonucleoprotein A1 OS=Homo sapiens GN=I	211.70	60.91	8	20	25	86	1.555E9	307	33.1	9.13
P07355	Annexin A2 OS=Homo sapiens GN=ANXA2 PE=1 SV=2 - [ANXA2_HU	208.44	69.62	24	31	31	84	1.358E9	339	38.6	7.75
P51991	Heterogeneous nuclear ribonucleoprotein A3 OS=Homo sapiens GN=I	206.06	47.35	2	24	26	78	1.444E9	378	39.6	9.01
P04264	Keratin, type II cytoskeletal 1 OS=Homo sapiens GN=KRT1 PE=1 SV=	164.42	54.04	12	30	36	67	1.004E9	644	66.0	8.12
Q6ZMU5	Tripartite motif-containing protein 72 OS=Homo sapiens GN=TRIM72	147.81	53.46	1	22	22	54	1.272E9	477	52.7	6.48
Q96AG4	Leucine-rich repeat-containing protein 59 OS=Homo sapiens GN=LRR	139.79	56.03	2	19	19	47	1.323E9	307	34.9	9.57
P04406	Glyceraldehyde-3-phosphate dehydrogenase OS=Homo sapiens GN=(	137.93	81.49	3	21	21	56	1.455E9	335	36.0	8.46
P08727	Keratin, type I cytoskeletal 19 OS=Homo sapiens GN=KRT19 PE=1 S\	119.81	79.25	18	25	33	49	4.037E8	400	44.1	5.14
B2R5W2	Heterogeneous nuclear ribonucleoproteins C1/C2 OS=Homo sapiens (	117.21	53.79	14	1	19	47	2.306E9	290	31.9	5.24
P13645	Keratin, type I cytoskeletal 10 OS=Homo sapiens GN=KRT10 PE=1 S\	115.66	51.71	20	29	33	49	6.171E8	584	58.8	5.21
P07910	Heterogeneous nuclear ribonucleoproteins C1/C2 OS=Homo sapiens (	109.50	55.23	11	1	19	46	2.306E9	306	33.6	5.08
P46777	60S ribosomal protein L5 OS=Homo sapiens GN=RPL5 PE=1 SV=3 -	109.42	51.18	2	20	20	44	9.741E8	297	34.3	9.72
Q99988	Growth/differentiation factor 15 OS=Homo sapiens GN=GDF15 PE=1	108.32	51.62	2	13	13	41	2.640E9	308	34.1	9.66
P35527	Keratin, type I cytoskeletal 9 OS=Homo sapiens GN=KRT9 PE=1 SV=	93.90	57.46	2	24	25	35	5.736E8	623	62.0	5.24
Q9Y295	Developmentally-regulated GTP-binding protein 1 OS=Homo sapiens	84.67	62.13	3	18	18	32	4.860E8	367	40.5	8.90
O15372	Eukaryotic translation initiation factor 3 subunit H OS=Homo sapiens	83.09	55.97	9	19	19	35	3.055E8	352	39.9	6.54
P06748	Nucleophosmin OS=Homo sapiens GN=NPM1 PE=1 SV=2 - [NPM_HU	81.02	39.80	3	12	12	30	8.333E8	294	32.6	4.78
Q12904	Aminoacyl tRNA synthase complex-interacting multifunctional protein	80.74	62.18	2	16	16	29	5.827E8	312	34.3	8.43
P51665	26S proteasome non-ATPase regulatory subunit 7 OS=Homo sapiens	76.61	49.38	4	12	12	28	2.705E8	324	37.0	6.77
A0A0C4DGB6	Serum albumin OS=Homo sapiens GN=ALB PE=1 SV=1 - [A0A0C4DG	70.24	27.32	8	13	13	42	2.420E8	604	69.2	6.37
O43684	Mitotic checkpoint protein BUB3 OS=Homo sapiens GN=BUB3 PE=1 S	69.97	58.54	3	16	16	30	6.091E8	328	37.1	6.84
Q13347	Eukaryotic translation initiation factor 3 subunit I OS=Homo sapiens (	69.80	63.08	2	16	16	29	3.121E8	325	36.5	5.64
P62136	Serine/threonine-protein phosphatase PP1-alpha catalytic subunit OS=	69.66	56.36	7	5	18	30	2.725E8	330	37.5	6.33
P05198	Eukaryotic translation initiation factor 2 subunit 1 OS=Homo sapiens (	66.45	67.94	3	21	21	30	3.084E8	315	36.1	5.08
P62995	Transformer-2 protein homolog beta OS=Homo sapiens GN=TRA2B F	64.93	52.08	3	13	16	27	1.163E9	288	33.6	11.25
Q13247	Serine/arginine-rich splicing factor 6 OS=Homo sapiens GN=SRSF6 PI	64.51	33.43	5	14	15	28	8.171E8	344	39.6	11.43
P35908	Keratin, type II cytoskeletal 2 epidermal OS=Homo sapiens GN=KRT2	64.11	48.36	8	14	22	31	5.218E8	639	65.4	8.00
D6R9P3	Heterogeneous nuclear ribonucleoprotein A/B OS=Homo sapiens GN=	62.59	40.00	4	14	15	28	4.961E8	280	30.3	7.91
E7EU96	Casein kinase II subunit alpha OS=Homo sapiens GN=CSNK2A1 PE=1	61.76	53.25	6	15	16	28	2.256E8	385	45.3	7.94
A0A087WUK2	Heterogeneous nuclear ribonucleoprotein D-like OS=Homo sapiens GI	61.55	32.51	2	14	16	30	6.084E8	363	40.0	9.96
P22087	rRNA 2'-O-methyltransferase fibrillarin OS=Homo sapiens GN=FBL PE	60.78	61.68	10	16	16	27	2.852E8	321	33.8	10.18
Q13151	Heterogeneous nuclear ribonucleoprotein A0 OS=Homo sapiens GN=I	57.41	43.28	1	11	13	26	4.230E8	305	30.8	9.29
Q7L2H7	Eukaryotic translation initiation factor 3 subunit M OS=Homo sapiens	57.18	44.39	5	12	12	24	2.146E8	374	42.5	5.63
P62140	Serine/threonine-protein phosphatase PP1-beta catalytic subunit OS=	55.78	44.65	6	4	14	25	2.183E8	327	37.2	6.19
P40937	Replication factor C subunit 5 OS=Homo sapiens GN=RFC5 PE=1 SV=	54.19	53.24	6	15	15	23	1.751E8	340	38.5	7.20
F8VYE8	Serine/threonine-protein phosphatase OS=Homo sapiens GN=PPP1CC	53.88	48.03	8	1	14	23	2.309E8	304	34.9	5.26
Q9NYK5	39S ribosomal protein L39, mitochondrial OS=Homo sapiens GN=MRI	53.55	47.04	2	15	15	24	2.426E8	338	38.7	7.65
Q13243	Serine/arginine-rich splicing factor 5 OS=Homo sapiens GN=SRSF5 PI	53.22	31.62	4	10	11	22	1.176E9	272	31.2	11.59
Q9BQE3	Tubulin alpha-1C chain OS=Homo sapiens GN=TUBA1C PE=1 SV=1 -	51.98	34.52	22	3	11	22	2.090E8	449	49.9	5.10
Q9Y3F4	Serine-threonine kinase receptor-associated protein OS=Homo sapien	51.16	46.00	2	13	13	20	1.635E8	350	38.4	5.12
Q9H9J2	39S ribosomal protein L44, mitochondrial OS=Homo sapiens GN=MRI	50.00	34.34	1	12	12	24	1.659E8	332	37.5	8.40
P68366	Tubulin alpha-4A chain OS=Homo sapiens GN=TUBA4A PE=1 SV=1 -	49.53	30.36	11	2	10	19	1.991E8	448	49.9	5.06
P60709	Actin, cytoplasmic 1 OS=Homo sapiens GN=ACTB PE=1 SV=1 - [ACT	49.45	42.40	21	5	11	20	2.326E8	375	41.7	5.48
Q9UKM9	RNA-binding protein Raly OS=Homo sapiens GN=RALY PE=1 SV=1 -	48.47	46.08	6	13	13	21	3.072E8	306	32.4	9.17
K7ES61	39S ribosomal protein L4, mitochondrial (Fragment) OS=Homo sapien	47.31	59.00	7	12	12	19	2.341E8	300	33.8	9.83

Q13155	Aminoacyl tRNA synthase complex-interacting multifunctional protein	47.29	59.06	3	13	13	23	5.003E8	320	35.3	8.22
Q14103	Heterogeneous nuclear ribonucleoprotein D0 OS=Homo sapiens GN=	46.64	35.49	6	5	11	21	5.344E8	355	38.4	7.81
P48729	Casein kinase I isoform alpha OS=Homo sapiens GN=CSNK1A1 PE=1	45.38	45.10	8	3	12	24	2.393E8	337	38.9	9.57
P68104	Elongation factor 1-alpha 1 OS=Homo sapiens GN=EEF1A1 PE=1 SV=	44.37	23.38	7	9	9	15	3.412E8	462	50.1	9.01
F5H265	Polyubiquitin-C (Fragment) OS=Homo sapiens GN=UBC PE=1 SV=1 -	44.04	63.09	23	5	5	20	1.538E9	149	16.8	6.58
Q96DV4	39S ribosomal protein L38, mitochondrial OS=Homo sapiens GN=MRP	42.49	30.00	1	11	11	22	1.641E8	380	44.6	7.53
P19784	Casein kinase II subunit alpha' OS=Homo sapiens GN=CSNK2A2 PE=	42.26	48.29	4	13	14	20	1.264E8	350	41.2	8.56
A0A0G2JPF8	Uncharacterized protein OS=Homo sapiens PE=4 SV=1 - [A0A0G2JPF	42.21	23.55	5	1	8	19	1.690E9	293	32.0	5.68
Q5JRI1	Serine/arginine-rich-splicing factor 10 OS=Homo sapiens GN=SRSF10	42.09	44.77	5	10	10	15	2.187E8	172	20.9	10.48
P09001	39S ribosomal protein L3, mitochondrial OS=Homo sapiens GN=MRPL	42.06	41.09	5	12	12	15	1.397E8	348	38.6	9.48
Q13595	Transformer-2 protein homolog alpha OS=Homo sapiens GN=TRA2A	41.92	37.94	1	10	13	22	4.699E8	282	32.7	11.27
G5E9W7	28S ribosomal protein S22, mitochondrial OS=Homo sapiens GN=MRP	40.93	54.55	7	16	16	21	9.568E7	319	36.8	6.81
P29966	Myristoylated alanine-rich C-kinase substrate OS=Homo sapiens GN=	40.42	31.02	1	9	9	17	1.218E9	332	31.5	4.45
Q15366	Poly(rC)-binding protein 2 OS=Homo sapiens GN=PCBP2 PE=1 SV=1	39.68	56.71	21	1	13	16	3.107E8	365	38.6	6.79
Q15014	Mortality factor 4-like protein 2 OS=Homo sapiens GN=MORF4L2 PE=	38.74	55.56	5	12	15	19	1.461E8	288	32.3	9.72
Q15365	Poly(rC)-binding protein 1 OS=Homo sapiens GN=PCBP1 PE=1 SV=2	38.51	50.00	13	8	12	17	2.871E8	356	37.5	7.09
H3BRU6	Poly(rC)-binding protein 2 (Fragment) OS=Homo sapiens GN=PCBP2	36.65	69.10	21	1	13	16	3.107E8	301	31.7	8.44
P40938	Replication factor C subunit 3 OS=Homo sapiens GN=RFC3 PE=1 SV=	36.23	46.91	2	14	14	19	1.111E8	356	40.5	8.34
C9J9K3	40S ribosomal protein SA (Fragment) OS=Homo sapiens GN=RPSA P	36.18	38.40	4	9	9	12	2.635E8	263	29.4	5.25
O95983	Methyl-CpG-binding domain protein 3 OS=Homo sapiens GN=MBD3 P	36.07	36.77	7	13	13	15	1.081E8	291	32.8	5.34
P02533	Keratin, type I cytoskeletal 14 OS=Homo sapiens GN=KRT14 PE=1 S	35.08	33.05	17	5	15	18	2.916E8	472	51.5	5.16
P52907	F-actin-capping protein subunit alpha-1 OS=Homo sapiens GN=CAPZ	34.54	55.94	1	8	10	17	3.493E8	286	32.9	5.69
O60506	Heterogeneous nuclear ribonucleoprotein Q OS=Homo sapiens GN=S	33.72	25.84	5	15	15	16	9.598E7	623	69.6	8.59
P13647	Keratin, type II cytoskeletal 5 OS=Homo sapiens GN=KRT5 PE=1 SV=	32.76	21.86	10	6	16	19	1.839E8	590	62.3	7.74
Q9NYT0	Pleckstrin-2 OS=Homo sapiens GN=PLEK2 PE=1 SV=1 - [PLEK2_HUM	31.90	49.29	4	14	14	15	2.441E8	353	39.9	9.41
P47755	F-actin-capping protein subunit alpha-2 OS=Homo sapiens GN=CAPZ	31.61	52.10	4	7	9	14	2.101E8	286	32.9	5.85
Q9UNQ2	Probable dimethyladenosine transferase OS=Homo sapiens GN=DIMT	31.42	37.38	3	9	9	13	1.176E8	313	35.2	9.99
P25685	DnaJ homolog subfamily B member 1 OS=Homo sapiens GN=DNAJB1	31.42	33.24	8	11	12	14	1.766E8	340	38.0	8.63
D6REM4	Casein kinase I isoform alpha OS=Homo sapiens GN=CSNK1A1 PE=1	31.08	55.51	2	1	10	19	2.057E8	236	27.7	9.44
Q96DI7	U5 small nuclear ribonucleoprotein 40 kDa protein OS=Homo sapiens	30.80	40.90	2	9	9	13	8.750E7	357	39.3	8.10
E9PB51	RNA-binding protein 4 (Fragment) OS=Homo sapiens GN=RBM4 PE=	30.71	62.50	8	12	12	14	1.804E8	240	26.3	7.55
P41091	Eukaryotic translation initiation factor 2 subunit 3 OS=Homo sapiens	30.67	23.73	4	9	9	12	1.491E8	472	51.1	8.40
C9J9W2	LIM and SH3 domain protein 1 (Fragment) OS=Homo sapiens GN=LA	30.41	74.70	4	9	9	12	2.586E8	166	19.0	9.01
E9PKG1	Protein arginine N-methyltransferase 1 OS=Homo sapiens GN=PRMT1	30.23	31.69	10	8	8	13	7.447E7	325	37.7	6.15
B3KTM8	Mortality factor 4-like protein 1 OS=Homo sapiens GN=MORF4L1 PE=	29.80	44.25	10	11	14	17	9.939E7	348	40.0	9.11
O95793	Double-stranded RNA-binding protein Staufen homolog 1 OS=Homo s	29.31	25.65	16	15	15	16	8.635E7	577	63.1	9.44
P0DN76	Splicing factor U2AF 35 kDa subunit-like protein OS=Homo sapiens G	29.20	39.17	5	7	7	13	2.925E8	240	27.9	8.81
D6RBQ9	Heterogeneous nuclear ribonucleoprotein D0 (Fragment) OS=Homo s	28.93	40.65	1	1	7	15	3.997E8	155	15.6	4.91
P51398	28S ribosomal protein S29, mitochondrial OS=Homo sapiens GN=DAP	28.76	31.66	11	11	11	13	1.124E8	398	45.5	8.88
P28482	Mitogen-activated protein kinase 1 OS=Homo sapiens GN=MAPK1 PE	28.55	43.33	7	12	12	16	1.294E8	360	41.4	6.98
P35249	Replication factor C subunit 4 OS=Homo sapiens GN=RFC4 PE=1 SV=	28.41	42.70	9	13	13	21	1.452E8	363	39.7	8.02
Q7L5D6	Golgi to ER traffic protein 4 homolog OS=Homo sapiens GN=GET4 PE	28.17	25.38	4	7	7	17	2.099E8	327	36.5	5.41
Q9BTV4	Transmembrane protein 43 OS=Homo sapiens GN=TMEM43 PE=1 SV	27.51	39.25	2	10	10	14	1.129E8	400	44.8	8.13
P69905	Hemoglobin subunit alpha OS=Homo sapiens GN=HBA1 PE=1 SV=2	27.30	42.96	3	6	6	12	2.804E8	142	15.2	8.68
Q9UH17	DNA dC->dU-editing enzyme APOBEC-3B OS=Homo sapiens GN=APO	27.21	30.89	3	8	9	14	1.442E8	382	45.9	6.06
O00487	26S proteasome non-ATPase regulatory subunit 14 OS=Homo sapiens	27.08	53.23	2	9	9	12	2.924E8	310	34.6	6.52
P08779	Keratin, type I cytoskeletal 16 OS=Homo sapiens GN=KRT16 PE=1 S	27.02	24.10	17	3	11	14	2.916E8	473	51.2	5.05
P07195	L-lactate dehydrogenase B chain OS=Homo sapiens GN=LDHB PE=1	26.59	35.33	4	10	10	12	2.405E8	334	36.6	6.05
P01023	Alpha-2-macroglobulin OS=Homo sapiens GN=A2M PE=1 SV=3 - [A2	26.42	3.46	1	3	5	10	3.648E8	1474	163.2	6.46
P02538	Keratin, type II cytoskeletal 6A OS=Homo sapiens GN=KRT6A PE=1 S	26.29	20.92	7	3	14	17	1.876E8	564	60.0	8.00
P61962	DDB1- and CUL4-associated factor 7 OS=Homo sapiens GN=DCAF7 P	26.25	34.50	2	10	10	13	1.429E8	342	38.9	5.52

A0A0A0MRV0	Ribosome-binding protein 1 OS=Homo sapiens GN=RRBP1 PE=1 SV=	26.17	24.54	4	11	11	14	1.080E8	1410	152.4	8.60
P05787	Keratin, type II cytoskeletal 8 OS=Homo sapiens GN=KRT8 PE=1 SV	26.12	27.12	19	9	15	17	1.175E8	483	53.7	5.59
P62714	Serine/threonine-protein phosphatase 2A catalytic subunit beta isofor	25.98	35.60	8	7	8	10	1.734E8	309	35.6	5.43
Q9UNM6	26S proteasome non-ATPase regulatory subunit 13 OS=Homo sapien	25.69	35.90	8	13	13	16	1.254E8	376	42.9	5.81
Q9NZJ7	Mitochondrial carrier homolog 1 OS=Homo sapiens GN=MTCH1 PE=1	25.42	19.28	3	7	7	10	1.418E8	389	41.5	9.32
P50750	Cyclin-dependent kinase 9 OS=Homo sapiens GN=CDK9 PE=1 SV=3	25.12	31.72	66	10	11	12	8.104E7	372	42.8	8.79
Q9H190	Syntenin-2 OS=Homo sapiens GN=SDCBP2 PE=1 SV=2 - [SDCB2_HU	25.10	21.92	1	7	7	7	2.284E8	292	31.6	9.07
P82933	28S ribosomal protein S9, mitochondrial OS=Homo sapiens GN=MRP	25.07	32.83	1	11	11	13	8.109E7	396	45.8	9.51
Q00577	Transcriptional activator protein Pur-alpha OS=Homo sapiens GN=PU	25.07	41.30	3	10	11	18	3.631E8	322	34.9	6.44
P20742	Pregnancy zone protein OS=Homo sapiens GN=PZP PE=1 SV=4 - [P	24.41	2.29	2	1	3	10	2.367E8	1482	163.8	6.38
F8VZJ2	Nascent polypeptide-associated complex subunit alpha OS=Homo sa	24.31	47.06	9	6	6	9	2.824E8	136	15.0	4.91
P35250	Replication factor C subunit 2 OS=Homo sapiens GN=RFC2 PE=1 SV	23.84	37.29	10	10	10	11	9.986E7	354	39.1	6.44
P67809	Nuclease-sensitive element-binding protein 1 OS=Homo sapiens GN=	23.40	34.88	4	3	7	10	1.612E8	324	35.9	9.88
O75367	Core histone macro-H2A.1 OS=Homo sapiens GN=H2AFY PE=1 SV=4	23.34	39.25	5	10	10	10	3.685E7	372	39.6	9.79
O96008	Mitochondrial import receptor subunit TOM40 homolog OS=Homo sa	22.68	22.99	3	7	7	9	7.831E7	361	37.9	7.25
P16989	Y-box-binding protein 3 OS=Homo sapiens GN=YBX3 PE=1 SV=4 - [	22.48	16.13	3	1	5	8	1.068E8	372	40.1	9.77
E9PK01	Elongation factor 1-delta (Fragment) OS=Homo sapiens GN=EEF1D	22.33	49.04	22	9	9	13	1.570E8	261	28.8	5.02
P05412	Transcription factor AP-1 OS=Homo sapiens GN=JUN PE=1 SV=2 - [	22.24	23.87	1	4	4	8	1.346E8	331	35.7	8.76
A0A140T933	HLA class I histocompatibility antigen, A-3 alpha chain (Fragment) O	22.20	39.13	25	8	9	10	1.792E8	299	34.2	6.71
P36578	60S ribosomal protein L4 OS=Homo sapiens GN=RPL4 PE=1 SV=5 -	21.99	25.06	4	10	10	12	7.333E7	427	47.7	11.06
P61160	Actin-related protein 2 OS=Homo sapiens GN=ACTR2 PE=1 SV=1 - [	21.85	23.35	4	7	7	9	9.186E7	394	44.7	6.74
P36957	Dihydrolipoyllysine-residue succinyltransferase component of 2-oxogl	21.66	22.30	5	8	8	8	5.814E7	453	48.7	8.95
P0DMV8	Heat shock 70 kDa protein 1A OS=Homo sapiens GN=HSPA1A PE=1	21.47	16.54	7	6	8	10	5.244E7	641	70.0	5.66
P31942	Heterogeneous nuclear ribonucleoprotein H3 OS=Homo sapiens GN=	21.30	29.48	5	8	8	10	1.303E8	346	36.9	6.87
P23396	40S ribosomal protein S3 OS=Homo sapiens GN=RPS3 PE=1 SV=2 -	21.18	51.44	12	10	10	10	1.150E8	243	26.7	9.66
Q5JP53	Tubulin beta chain OS=Homo sapiens GN=TUBB PE=1 SV=1 - [Q5JP	20.72	22.77	19	6	8	10	6.413E7	426	47.7	4.81
Q99496	E3 ubiquitin-protein ligase RING2 OS=Homo sapiens GN=RNF2 PE=1	20.46	19.35	3	5	5	7	5.291E7	336	37.6	6.84
P63267	Actin, gamma-enteric smooth muscle OS=Homo sapiens GN=ACTG2	20.43	22.07	14	1	7	11	1.420E8	376	41.8	5.48
Q9UN81	LINE-1 retrotransposable element ORF1 protein OS=Homo sapiens G	20.40	34.02	1	10	10	11	1.021E8	338	40.0	9.51
O43709	Probable 18S rRNA (guanine-N(7))-methyltransferase OS=Homo sapi	20.27	30.60	9	7	7	9	1.414E8	281	31.9	8.73
Q70UQ0	Inhibitor of nuclear factor kappa-B kinase-interacting protein OS=Ho	19.96	25.43	1	9	9	9	4.319E7	350	39.3	9.17
O43837	Isocitrate dehydrogenase [NAD] subunit beta, mitochondrial OS=Hon	19.92	24.42	4	8	8	9	4.592E7	385	42.2	8.46
Q9BRQ8	Apoptosis-inducing factor 2 OS=Homo sapiens GN=AIFM2 PE=1 SV=	19.69	25.74	1	7	7	10	3.426E7	373	40.5	9.11
Q96QR8	Transcriptional activator protein Pur-beta OS=Homo sapiens GN=PUF	19.30	41.99	4	9	10	14	2.514E8	312	33.2	5.43
Q92665	28S ribosomal protein S31, mitochondrial OS=Homo sapiens GN=MR	19.21	28.61	1	8	8	10	6.275E7	395	45.3	9.29
P35637	RNA-binding protein FUS OS=Homo sapiens GN=FUS PE=1 SV=1 - [	18.98	18.82	4	5	5	7	1.361E8	526	53.4	9.36
Q9UMY1	Nucleolar protein 7 OS=Homo sapiens GN=NOL7 PE=1 SV=2 - [NOL7	18.96	21.40	2	7	7	8	1.306E8	257	29.4	9.67
A6NG10	WW domain-binding protein 2 OS=Homo sapiens GN=WBP2 PE=1 S	18.94	43.51	9	8	8	8	1.207E8	239	25.8	5.99
Q9UBS4	DnaJ homolog subfamily B member 11 OS=Homo sapiens GN=DNAJ	18.75	22.35	2	7	7	7	5.901E7	358	40.5	6.18
A0A1W2PRV5	Survival motor neuron protein OS=Homo sapiens GN=SMN2 PE=1 S	18.33	21.28	8	5	5	9	1.852E8	282	30.4	5.73
O60547	GDP-mannose 4,6 dehydratase OS=Homo sapiens GN=GMDS PE=1	18.24	28.76	1	9	9	11	5.169E7	372	41.9	7.31
Q96N66	Lysophospholipid acyltransferase 7 OS=Homo sapiens GN=MBOAT7	18.23	18.22	6	6	6	7	6.803E7	472	52.7	8.97
P78345	Ribonuclease P protein subunit p38 OS=Homo sapiens GN=RPP38 P	18.10	34.28	3	8	8	9	6.068E7	283	31.8	9.92
P61247	40S ribosomal protein S3a OS=Homo sapiens GN=RPS3A PE=1 SV=	17.65	31.06	12	9	9	10	3.869E7	264	29.9	9.73
P08754	Guanine nucleotide-binding protein G(k) subunit alpha OS=Homo sa	17.57	27.68	23	3	7	8	1.045E8	354	40.5	5.69
P11142	Heat shock cognate 71 kDa protein OS=Homo sapiens GN=HSPA8 P	17.26	19.35	14	7	9	10	3.863E7	646	70.9	5.52
Q9P031	Thyroid transcription factor 1-associated protein 26 OS=Homo sapien	17.13	27.39	2	8	8	8	3.919E7	241	28.7	9.88
Q9NQ29	Putative RNA-binding protein Luc7-like 1 OS=Homo sapiens GN=LUC	17.08	23.45	7	5	8	8	4.150E7	371	43.7	9.92
P00338	L-lactate dehydrogenase A chain OS=Homo sapiens GN=LDHA PE=1	17.05	29.82	13	9	9	11	1.867E8	332	36.7	8.27
P46736	Lys-63-specific deubiquitinase BRCC36 OS=Homo sapiens GN=BRCC3	17.00	22.15	6	6	6	7	5.927E7	316	36.0	5.92
Q13190	Syntaxin-5 OS=Homo sapiens GN=STX5 PE=1 SV=2 - [STX5_HUMAN	16.72	27.04	5	6	6	8	4.724E7	355	39.6	9.16

O00622	Protein CYR61 OS=Homo sapiens GN=CYR61 PE=1 SV=1 - [CYR61_H	16.67	17.06	1	6	6	9	1.399E8	381	42.0	8.21
Q9Y3A4	Ribosomal RNA-processing protein 7 homolog A OS=Homo sapiens G	16.47	36.43	2	8	8	9	6.452E7	280	32.3	9.58
Q96L58	Beta-1,3-galactosyltransferase 6 OS=Homo sapiens GN=B3GALT6 PE-	16.41	19.76	1	5	5	7	5.752E7	329	37.1	9.66
P40926	Malate dehydrogenase, mitochondrial OS=Homo sapiens GN=MDH2 F	16.39	23.96	2	6	6	7	5.317E7	338	35.5	8.68
P29084	Transcription initiation factor IIE subunit beta OS=Homo sapiens GN=	16.16	44.67	4	11	11	12	6.648E7	291	33.0	9.66
H3BQN4	Fructose-bisphosphate aldolase OS=Homo sapiens GN=ALDOA PE=1	16.06	26.04	17	9	9	10	5.953E7	361	39.3	8.40
Q9BU76	Multiple myeloma tumor-associated protein 2 OS=Homo sapiens GN=	15.97	27.76	1	6	6	6	3.595E7	263	29.4	10.02
Q6PK04	Coiled-coil domain-containing protein 137 OS=Homo sapiens GN=CCI	15.96	25.26	2	6	6	8	4.308E7	289	33.2	10.93
Q07820	Induced myeloid leukemia cell differentiation protein Mcl-1 OS=Homo	15.87	21.14	3	6	6	6	1.057E8	350	37.3	5.66
Q16795	NADH dehydrogenase [ubiquinone] 1 alpha subcomplex subunit 9, m	15.81	31.56	3	9	9	12	4.626E7	377	42.5	9.80
P04899	Guanine nucleotide-binding protein G(i) subunit alpha-2 OS=Homo sa	15.79	25.92	23	3	7	8	9.226E7	355	40.4	5.54
P28562	Dual specificity protein phosphatase 1 OS=Homo sapiens GN=DUSP1	15.56	22.34	1	6	6	8	4.020E7	367	39.3	7.20
Q96C36	Pyrroline-5-carboxylate reductase 2 OS=Homo sapiens GN=PYCR2 PE	15.53	19.06	6	5	5	6	3.221E7	320	33.6	7.77
P56470	Galectin-4 OS=Homo sapiens GN=LGALS4 PE=1 SV=1 - [LEG4_HUMA	15.51	25.08	2	6	6	8	3.774E7	323	35.9	9.16
Q15024	Exosome complex component RRP42 OS=Homo sapiens GN=EXOSC7	15.48	34.71	1	7	7	9	7.113E7	291	31.8	5.19
P11177	Pyruvate dehydrogenase E1 component subunit beta, mitochondrial (	15.47	24.23	3	8	8	9	5.754E7	359	39.2	6.65
P13995	Bifunctional methylenetetrahydrofolate dehydrogenase/cyclohydrolase	15.47	33.14	6	8	8	8	8.781E7	350	37.9	8.73
P49411	Elongation factor Tu, mitochondrial OS=Homo sapiens GN=TUFM PE-	15.14	19.69	1	7	7	7	4.757E7	452	49.5	7.61
A0A087X1N3	Ribonuclease P 40kDa subunit, isoform CRA_c OS=Homo sapiens GN-	14.75	15.89	4	4	4	6	6.204E7	321	36.8	6.40
E9PIT3	Prothrombin OS=Homo sapiens GN=F2 PE=1 SV=1 - [E9PIT3_HUMA	14.65	6.69	2	3	3	9	4.931E7	583	65.4	5.71
A0A0A6YYF2	HCG1811249, isoform CRA_e OS=Homo sapiens GN=LAMA3 PE=1 SV	14.31	6.15	5	8	8	8	2.755E7	1724	190.3	8.15
A4D1E9	GTP-binding protein 10 OS=Homo sapiens GN=GTPBP10 PE=1 SV=1	14.30	25.58	4	8	8	8	6.273E7	387	42.9	9.03
J3KTA4	Probable ATP-dependent RNA helicase DDX5 OS=Homo sapiens GN=	14.10	14.01	13	6	7	8	4.152E7	614	69.0	8.85
A0A0G2JLR5	Ras association domain-containing protein 7 OS=Homo sapiens GN=F	13.93	23.74	7	6	6	6	5.572E7	337	36.3	6.06
Q9BYG3	MKI67 FHA domain-interacting nucleolar phosphoprotein OS=Homo s	13.90	17.75	4	5	5	6	4.829E7	293	34.2	9.88
P82673	28S ribosomal protein S35, mitochondrial OS=Homo sapiens GN=MRF	13.88	31.27	2	8	8	10	4.786E7	323	36.8	8.24
P07900	Heat shock protein HSP 90-alpha OS=Homo sapiens GN=HSP90AA1 F	13.75	11.20	8	4	8	9	5.069E7	732	84.6	5.02
Q14493	Histone RNA hairpin-binding protein OS=Homo sapiens GN=SLBP PE-	13.71	22.59	5	5	5	6	5.277E7	270	31.3	7.47
O00154	Cytosolic acyl coenzyme A thioester hydrolase OS=Homo sapiens GN-	13.69	14.21	2	4	4	5	6.275E7	380	41.8	8.54
Q9NXS2	Glutaminyl-peptide cyclotransferase-like protein OS=Homo sapiens GI	13.67	15.18	2	6	6	7	6.747E7	382	42.9	9.82
E7ER27	Peroxisomal multifunctional enzyme type 2 OS=Homo sapiens GN=HS	13.63	14.60	5	6	6	6	5.794E7	500	53.9	6.33
P15880	40S ribosomal protein S2 OS=Homo sapiens GN=RPS2 PE=1 SV=2 -	13.53	25.60	9	6	6	7	3.866E7	293	31.3	10.24
B4DY09	Interleukin enhancer-binding factor 2 OS=Homo sapiens GN=ILF2 PE	13.51	25.00	4	6	6	6	3.706E7	352	38.9	4.94
M0R3B2	Nitric oxide synthase-interacting protein (Fragment) OS=Homo sapier	13.46	27.80	6	5	5	7	3.837E7	241	26.8	8.82
Q9Y383	Putative RNA-binding protein Luc7-like 2 OS=Homo sapiens GN=LUC	13.32	15.56	2	3	6	6	4.282E7	392	46.5	10.01
Q9UBP9	PTB domain-containing engulfment adapter protein 1 OS=Homo sapi	13.32	22.37	3	7	7	7	6.590E7	304	34.5	7.90
P62333	26S proteasome regulatory subunit 10B OS=Homo sapiens GN=PSMC	13.23	23.39	6	6	6	6	5.834E7	389	44.1	7.49
Q9NYL9	Tropomodulin-3 OS=Homo sapiens GN=TMOD3 PE=1 SV=1 - [TMOD	13.11	27.84	5	8	9	9	6.800E7	352	39.6	5.19
A0A0B4J1Z1	Serine/arginine-rich-splicing factor 7 OS=Homo sapiens GN=SRSF7 PI	12.90	45.26	6	7	7	8	2.331E8	137	15.8	9.80
A0A1W2PPZ5	Transcription elongation factor A protein 1 OS=Homo sapiens GN=TC	12.90	20.60	13	6	6	7	4.086E7	301	33.9	8.38
Q96QD9	UAP56-interacting factor OS=Homo sapiens GN=FYTTD1 PE=1 SV=3	12.89	24.53	5	7	7	7	4.239E7	318	35.8	11.78
H0YLE8	Ras GTPase-activating-like protein IQGAP1 OS=Homo sapiens GN=IQ	12.69	8.11	2	6	6	6	4.836E7	1085	124.7	8.76
O14908	PDZ domain-containing protein GIPC1 OS=Homo sapiens GN=GIPC1	12.67	31.23	7	7	7	8	4.496E7	333	36.0	6.28
F8VZY9	Keratin, type I cytoskeletal 18 OS=Homo sapiens GN=KRT18 PE=1 S	12.63	18.16	10	5	7	7	2.758E8	391	43.7	5.35
P18464	HLA class I histocompatibility antigen, B-51 alpha chain OS=Homo sa	12.57	28.73	36	4	6	6	1.350E8	362	40.5	6.39
F5GYH1	Adaptin ear-binding coat-associated protein 1 (Fragment) OS=Homo	12.56	32.92	14	5	5	5	8.743E7	240	25.9	8.35
P17535	Transcription factor jun-D OS=Homo sapiens GN=JUND PE=1 SV=3 -	12.51	21.33	2	4	4	6	6.981E7	347	35.2	7.37
O15143	Actin-related protein 2/3 complex subunit 1B OS=Homo sapiens GN=	12.45	16.94	7	5	5	7	3.442E7	372	40.9	8.35
Q9BWM7	Sideroflexin-3 OS=Homo sapiens GN=SFXN3 PE=1 SV=3 - [SFXN3_H	12.35	28.35	3	7	7	7	3.718E7	321	35.5	9.10
E7EP32	Guanine nucleotide-binding protein G(I)/G(S)/G(T) subunit beta-2 OS	12.03	16.89	17	5	5	6	9.732E7	296	32.4	6.15
A0A0G2JL54	Complement C4-B OS=Homo sapiens GN=C4B_2 PE=1 SV=1 - [A0A0	11.95	0.88	9	1	1	4	1.930E7	1698	187.6	7.33

Q9BW04	Specifically androgen-regulated gene protein OS=Homo sapiens GN=	11.90	18.64	1	8	8	8	6.253E7	601	63.9	8.62
E9PMI6	Methylosome subunit pICln OS=Homo sapiens GN=CLNS1A PE=1 SV	11.72	49.10	5	5	5	9	6.707E7	167	18.2	4.41
A0A087WXC5	NADH dehydrogenase [ubiquinone] 1 alpha subcomplex subunit 10, r	11.71	19.72	8	8	8	8	6.056E7	355	40.8	8.48
Q17RN3	Protein FAM98C OS=Homo sapiens GN=FAM98C PE=2 SV=1 - [FA98	11.68	22.92	3	5	5	6	2.954E7	349	37.3	7.18
Q9NXW2	DnaJ homolog subfamily B member 12 OS=Homo sapiens GN=DNAJE	11.59	19.20	3	5	6	7	5.333E7	375	41.8	8.53
P14618	Pyruvate kinase PKM OS=Homo sapiens GN=PKM PE=1 SV=4 - [KPY	11.44	18.27	12	6	6	6	4.754E7	531	57.9	7.84
Q5T6W2	Heterogeneous nuclear ribonucleoprotein K (Fragment) OS=Homo sa	11.38	24.01	3	6	6	6	4.455E7	379	41.8	5.59
P35237	Serpin B6 OS=Homo sapiens GN=SERPINB6 PE=1 SV=3 - [SPB6_HU	11.19	21.28	5	6	6	6	3.467E7	376	42.6	5.27
G3XAN4	Translocating chain-associated membrane protein 1 OS=Homo sapier	11.12	19.44	2	5	5	6	1.027E8	288	33.4	9.89
Q13283	Ras GTPase-activating protein-binding protein 1 OS=Homo sapiens G	11.08	20.17	1	7	7	9	8.311E7	466	52.1	5.52
Q9H4B7	Tubulin beta-1 chain OS=Homo sapiens GN=TUBB1 PE=1 SV=1 - [TE	11.07	8.87	1	1	3	4	6.164E7	451	50.3	5.17
P61964	WD repeat-containing protein 5 OS=Homo sapiens GN=WDR5 PE=1	10.99	20.96	4	5	5	6	1.045E8	334	36.6	8.27
P49770	Translation initiation factor eIF-2B subunit beta OS=Homo sapiens GN	10.94	17.66	4	5	5	5	2.440E7	351	39.0	6.16
Q00796	Sorbitol dehydrogenase OS=Homo sapiens GN=SORD PE=1 SV=4 - [	10.94	17.65	3	5	5	5	2.331E7	357	38.3	7.97
A0A1W2PP35	Heterogeneous nuclear ribonucleoprotein U (Fragment) OS=Homo sa	10.73	9.75	14	6	6	6	1.063E8	728	80.6	5.74
Q13751	Laminin subunit beta-3 OS=Homo sapiens GN=LAMB3 PE=1 SV=1 -	10.69	7.68	2	7	7	7	3.493E7	1172	129.5	7.21
Q5T440	Putative transferase CAF17, mitochondrial OS=Homo sapiens GN=IBA	10.65	18.54	1	4	4	4	6.117E7	356	38.1	9.83
P30740	Leukocyte elastase inhibitor OS=Homo sapiens GN=SERPINB1 PE=1	10.56	22.43	5	8	8	8	2.665E7	379	42.7	6.28
Q86W42	THO complex subunit 6 homolog OS=Homo sapiens GN=THOC6 PE=	10.12	23.17	1	5	5	6	1.989E7	341	37.5	7.43
Q9H9H4	Vacuolar protein sorting-associated protein 37B OS=Homo sapiens GN	10.02	29.82	3	6	6	6	3.231E7	285	31.3	7.34
P15407	Fos-related antigen 1 OS=Homo sapiens GN=FOSL1 PE=1 SV=1 - [F	9.98	8.12	1	2	2	3	4.268E7	271	29.4	8.02
E7EVY0	Mitochondrial inner membrane protein OXA1L OS=Homo sapiens GN=	9.91	15.20	5	7	7	7	2.740E7	408	45.1	9.17
C9J3L8	Translocon-associated protein subunit alpha OS=Homo sapiens GN=S	9.87	12.83	5	3	3	4	1.960E8	265	29.6	4.30
F5H3X6	Prohibitin-2 (Fragment) OS=Homo sapiens GN=PHB2 PE=1 SV=2 - [F	9.80	23.00	7	4	4	5	2.037E7	213	23.6	6.95
Q9UH62	Armadillo repeat-containing X-linked protein 3 OS=Homo sapiens GN=	9.73	20.32	2	6	6	7	3.858E7	379	42.5	8.37
Q9HBH5	Retinol dehydrogenase 14 OS=Homo sapiens GN=RDH14 PE=1 SV=1	9.64	13.99	1	4	4	4	1.716E7	336	36.8	8.79
B7Z4J8	Zinc finger protein 346 OS=Homo sapiens GN=ZNF346 PE=1 SV=1 -	9.60	21.94	6	3	3	4	3.898E7	196	21.7	8.66
P19387	DNA-directed RNA polymerase II subunit RPB3 OS=Homo sapiens GN	9.60	43.64	2	7	7	7	3.863E7	275	31.4	4.92
P50479	PDZ and LIM domain protein 4 OS=Homo sapiens GN=PDLIM4 PE=1	9.57	17.58	2	5	5	7	5.806E7	330	35.4	7.91
P39023	60S ribosomal protein L3 OS=Homo sapiens GN=RPL3 PE=1 SV=2 -	9.55	20.60	7	7	7	7	9.458E7	403	46.1	10.18
A0A087WZB5	Beta-parvin OS=Homo sapiens GN=PARVB PE=1 SV=1 - [A0A087WZ	9.54	17.99	5	5	5	5	2.643E7	289	33.2	5.90
Q5T8U3	60S ribosomal protein L7a (Fragment) OS=Homo sapiens GN=RPL7A	9.44	21.99	3	4	4	4	1.740E7	191	21.5	11.02
C9JQ42	Glycogenin-1 (Fragment) OS=Homo sapiens GN=GYG1 PE=1 SV=8 -	9.43	15.47	5	4	4	4	5.413E7	265	29.9	6.15
Q96B36	Proline-rich AKT1 substrate 1 OS=Homo sapiens GN=AKT1S1 PE=1 S	9.42	16.41	3	3	3	5	4.014E7	256	27.4	4.75
E9PCY7	Heterogeneous nuclear ribonucleoprotein H OS=Homo sapiens GN=H	9.40	13.99	22	4	4	4	5.523E7	429	47.1	6.34
P19525	Interferon-induced, double-stranded RNA-activated protein kinase OS	9.40	8.89	3	4	4	4	1.910E7	551	62.1	8.40
O75190	DnaJ homolog subfamily B member 6 OS=Homo sapiens GN=DNAJB6	9.35	31.29	10	6	6	6	7.884E7	326	36.1	9.16
P08238	Heat shock protein HSP 90-beta OS=Homo sapiens GN=HSP90AB1 P	9.34	6.49	7	1	5	6	4.092E7	724	83.2	5.03
P53004	Biliverdin reductase A OS=Homo sapiens GN=BLVRA PE=1 SV=2 - [B	9.33	16.89	2	4	4	4	2.696E7	296	33.4	6.44
P63092	Guanine nucleotide-binding protein G(s) subunit alpha isoforms short	9.31	8.63	19	2	3	4	5.579E7	394	45.6	5.82
Q8WWH5	Probable tRNA pseudouridine synthase 1 OS=Homo sapiens GN=TRU	9.27	12.03	1	4	4	4	1.875E7	349	37.2	8.25
G3V150	Galactosylgalactosylxylosylprotein 3-beta-glucuronosyltransferase OS=	9.21	18.18	3	5	5	5	1.862E7	319	34.9	7.85
Q7Z7K6	Centromere protein V OS=Homo sapiens GN=CENPV PE=1 SV=1 - [C	9.19	18.55	2	3	3	3	3.507E7	275	29.9	9.73
G3V438	Activator of 90 kDa heat shock protein ATPase homolog 1 (Fragment)	9.17	30.54	5	4	4	4	7.529E7	203	23.0	7.44
Q8NBN7	Retinol dehydrogenase 13 OS=Homo sapiens GN=RDH13 PE=1 SV=2	9.15	27.79	2	6	6	6	3.121E7	331	35.9	8.10
Q3ZCQ8	Mitochondrial import inner membrane translocase subunit TIM50 OS=	9.07	17.56	10	5	5	6	3.406E7	353	39.6	8.37
P20042	Eukaryotic translation initiation factor 2 subunit 2 OS=Homo sapiens	9.02	15.62	1	5	5	5	2.250E7	333	38.4	5.80
P55735	Protein SEC13 homolog OS=Homo sapiens GN=SEC13 PE=1 SV=3 -	8.99	21.43	2	5	5	5	7.076E7	322	35.5	5.48
P07996	Thrombospondin-1 OS=Homo sapiens GN=THBS1 PE=1 SV=2 - [TSP	8.95	4.70	1	5	5	5	2.412E7	1170	129.3	4.94
Q7L0Y3	Mitochondrial ribonuclease P protein 1 OS=Homo sapiens GN=TRMT1	8.88	25.06	2	10	10	10	2.434E7	403	47.3	9.36
Q8WVM0	Dimethyladenosine transferase 1, mitochondrial OS=Homo sapiens G	8.78	23.70	1	6	6	9	4.582E7	346	39.5	9.26

Q99848	Probable rRNA-processing protein EBP2 OS=Homo sapiens GN=EBNA	8.69	25.16	2	6	6	6	3.542E7	306	34.8	10.10
Q9Y617	Phosphoserine aminotransferase OS=Homo sapiens GN=PSAT1 PE=1	8.63	14.32	1	5	5	5	3.310E7	370	40.4	7.66
O00743	Serine/threonine-protein phosphatase 6 catalytic subunit OS=Homo s	8.57	6.89	1	2	2	3	4.159E7	305	35.1	5.69
M0R2P8	PIH1 domain-containing protein 1 (Fragment) OS=Homo sapiens GN=	8.56	31.58	10	3	3	3	9.912E6	114	13.2	5.26
O15160	DNA-directed RNA polymerases I and III subunit RPAC1 OS=Homo sa	8.51	22.83	4	6	6	6	3.301E7	346	39.2	5.50
J3KQL8	Apolipoprotein L2 OS=Homo sapiens GN=APOL2 PE=1 SV=2 - [J3KQ	8.32	11.14	3	4	4	4	2.216E7	449	48.9	6.00
Q96QE5	Transcription elongation factor, mitochondrial OS=Homo sapiens GN=	8.24	19.17	2	7	7	7	2.177E7	360	41.6	9.32
Q9UJZ1	Stomatin-like protein 2, mitochondrial OS=Homo sapiens GN=STOML	8.23	25.00	3	5	5	5	2.882E7	356	38.5	7.39
P10909	Clusterin OS=Homo sapiens GN=CLU PE=1 SV=1 - [CLUS_HUMAN]	8.20	11.58	5	3	3	4	3.867E7	449	52.5	6.27
Q00534	Cyclin-dependent kinase 6 OS=Homo sapiens GN=CDK6 PE=1 SV=1	8.12	21.47	60	5	6	6	3.962E7	326	36.9	6.46
Q6IN84	rRNA methyltransferase 1, mitochondrial OS=Homo sapiens GN=MRM	7.90	19.26	2	5	5	6	3.702E7	353	38.6	7.94
A6NLN1	Polypyrimidine tract binding protein 1, isoform CRA_b OS=Homo sapi	7.88	15.18	9	5	5	7	5.969E7	527	56.5	9.38
Q15717	ELAV-like protein 1 OS=Homo sapiens GN=ELAVL1 PE=1 SV=2 - [EL/	7.86	17.48	1	4	4	5	1.887E7	326	36.1	9.17
P17612	cAMP-dependent protein kinase catalytic subunit alpha OS=Homo sa	7.75	10.26	4	2	4	4	3.315E7	351	40.6	8.79
F5H1C6	Fermitin family homolog 3 (Fragment) OS=Homo sapiens GN=FERMT	7.71	10.49	2	3	3	3	1.889E7	286	33.1	7.87
F8VRH0	Poly(rC)-binding protein 2 (Fragment) OS=Homo sapiens GN=PCBP2	7.58	33.55	1	1	5	5	9.630E7	310	32.0	8.07
Q9ULR0	Pre-mRNA-splicing factor ISY1 homolog OS=Homo sapiens GN=ISY1	7.50	15.09	4	3	3	3	2.880E7	285	33.0	5.17
Q9Y394	Dehydrogenase/reductase SDR family member 7 OS=Homo sapiens G	7.45	16.52	4	4	4	4	2.619E7	339	38.3	8.32
Q9UDY4	DnaJ homolog subfamily B member 4 OS=Homo sapiens GN=DNAJB4	7.44	18.10	2	3	5	5	3.352E7	337	37.8	8.50
Q86YD1	Prostate tumor-overexpressed gene 1 protein OS=Homo sapiens GN=	7.44	16.11	6	5	5	5	2.889E7	416	46.8	10.54
Q14344	Guanine nucleotide-binding protein subunit alpha-13 OS=Homo sapie	7.36	5.57	17	1	2	3	1.406E8	377	44.0	8.00
A0A087WVC4	cAMP-dependent protein kinase catalytic subunit beta OS=Homo sapi	7.24	8.88	11	1	3	3	2.603E7	338	39.2	8.82
Q8TCE1	Antithrombin-III OS=Homo sapiens GN=SERPINC1 PE=1 SV=1 - [Q8	7.21	6.95	2	2	2	3	5.943E7	259	29.1	8.81
Q9NX31	Oxidative stress-responsive serine-rich protein 1 OS=Homo sapiens G	7.16	17.81	1	3	3	3	1.943E7	292	31.8	8.48
Q9Y2P8	RNA 3'-terminal phosphate cyclase-like protein OS=Homo sapiens GN	7.13	12.60	4	4	4	4	1.203E7	373	40.8	9.26
A0A087WTT1	Polyadenylate-binding protein OS=Homo sapiens GN=PABPC1 PE=1 S	7.04	8.05	12	3	3	3	1.922E7	522	58.5	9.26
E9PKZ0	60S ribosomal protein L8 (Fragment) OS=Homo sapiens GN=RPL8 PE	7.00	16.59	5	3	3	3	2.824E7	205	22.4	10.76
Q9UET6	Putative tRNA (cytidine(32)/guanosine(34)-2'-O)-methyltransferase O	6.95	8.81	2	2	2	3	6.262E7	329	36.1	5.69
P60842	Eukaryotic initiation factor 4A-I OS=Homo sapiens GN=EIF4A1 PE=1	6.86	14.78	17	5	5	5	1.615E7	406	46.1	5.48
Q8IUX4	DNA dC->dU-editing enzyme APOBEC-3F OS=Homo sapiens GN=APO	6.86	7.51	3	2	3	4	3.261E7	373	45.0	7.23
E9PLA9	Caprin-1 (Fragment) OS=Homo sapiens GN=CAPRIN1 PE=1 SV=1 - [	6.85	24.19	3	4	4	4	2.049E7	186	20.2	7.40
O00401	Neural Wiskott-Aldrich syndrome protein OS=Homo sapiens GN=WAS	6.83	9.70	1	4	4	6	2.973E7	505	54.8	7.93
Q15327	Ankyrin repeat domain-containing protein 1 OS=Homo sapiens GN=A	6.77	13.48	1	3	3	3	2.158E7	319	36.2	7.50
Q9NPH2	Inositol-3-phosphate synthase 1 OS=Homo sapiens GN=ISYNA1 PE=	6.77	5.38	1	3	3	3	2.649E7	558	61.0	5.76
Q9BYD6	39S ribosomal protein L1, mitochondrial OS=Homo sapiens GN=MRPL	6.73	13.54	2	4	4	4	2.450E7	325	36.9	8.78
Q15738	Sterol-4-alpha-carboxylate 3-dehydrogenase, decarboxylating OS=Ho	6.72	22.25	2	5	5	6	2.915E7	373	41.9	8.06
Q15785	Mitochondrial import receptor subunit TOM34 OS=Homo sapiens GN=	6.68	16.83	1	4	4	4	1.600E7	309	34.5	8.98
Q9H9L3	Interferon-stimulated 20 kDa exonuclease-like 2 OS=Homo sapiens G	6.64	10.48	1	3	3	4	2.727E7	353	39.1	9.94
K7ELV2	Nucleoporin SEH1 (Fragment) OS=Homo sapiens GN=SEH1L PE=1 S	6.60	16.35	3	3	3	3	2.167E7	263	29.5	7.80
P01024	Complement C3 OS=Homo sapiens GN=C3 PE=1 SV=2 - [CO3_HUMA	6.57	3.97	1	4	4	6	1.433E8	1663	187.0	6.40
Q8TDN6	Ribosome biogenesis protein BRX1 homolog OS=Homo sapiens GN=E	6.50	15.58	1	5	5	5	1.387E7	353	41.4	9.92
Q92890	Ubiquitin recognition factor in ER-associated degradation protein 1 O	6.47	12.05	4	3	3	3	4.325E7	307	34.5	6.70
Q9BY42	Protein RTF2 homolog OS=Homo sapiens GN=RTFDC1 PE=1 SV=3 -	6.44	12.75	4	3	3	3	2.243E7	306	33.9	8.59
Q86YZ3	Hornerin OS=Homo sapiens GN=HRNR PE=1 SV=2 - [HORN_HUMAN	6.43	3.61	1	2	2	3	8.652E6	2850	282.2	10.04
P12236	ADP/ATP translocase 3 OS=Homo sapiens GN=SLC25A6 PE=1 SV=4	6.40	10.74	4	3	3	4	2.282E7	298	32.8	9.74
Q5JPU0	Pyruvate dehydrogenase E1 component subunit alpha, somatic form,	6.40	16.67	5	3	3	3	2.830E7	180	19.8	9.14
Q9BXJ4	Complement C1q tumor necrosis factor-related protein 3 OS=Homo s	6.37	12.60	2	3	3	4	4.358E7	246	27.0	6.52
B4DHE8	RNA-binding protein Musashi homolog 2 OS=Homo sapiens GN=MSI2	6.35	14.20	5	3	3	3	2.877E7	324	34.8	8.88
Q9UBV7	Beta-1,4-galactosyltransferase 7 OS=Homo sapiens GN=B4GALT7 PE	6.30	8.87	3	3	3	3	1.678E7	327	37.4	8.98
Q96DP5	Methionyl-tRNA formyltransferase, mitochondrial OS=Homo sapiens G	6.15	9.00	2	3	3	3	2.167E7	389	43.8	9.66
E7EMC7	Sequestosome-1 OS=Homo sapiens GN=SQSTM1 PE=1 SV=1 - [E7EI	5.99	18.52	7	4	4	4	2.888E7	378	41.0	7.52

O75436	Vacuolar protein sorting-associated protein 26A OS=Homo sapiens G		5.98	17.43	3	4	4	4	2.035E7	327	38.1	6.57
P24385	G1/S-specific cyclin-D1 OS=Homo sapiens GN=CCND1 PE=1 SV=1 -		5.96	14.58	1	3	3	3	2.436E7	295	33.7	5.02
Q6UX07	Dehydrogenase/reductase SDR family member 13 OS=Homo sapiens		5.93	7.96	1	3	3	3	1.230E7	377	40.8	7.69
B1AKM8	Phosphatidylserine decarboxylase proenzyme, mitochondrial (Fragme		5.86	17.11	4	4	4	4	1.527E7	228	26.3	9.82
Q14676	Mediator of DNA damage checkpoint protein 1 OS=Homo sapiens GN		5.80	2.06	1	2	2	3	6.446E7	2089	226.5	5.47
P81605	Dermcidin OS=Homo sapiens GN=DCD PE=1 SV=2 - [DCD_HUMAN]		5.71	22.73	1	2	2	2	3.600E7	110	11.3	6.54
Q8NBU5	ATPase family AAA domain-containing protein 1 OS=Homo sapiens G		5.64	5.54	1	2	2	2	2.110E7	361	40.7	6.90
F5H6X0	General transcription factor IIH subunit 3 (Fragment) OS=Homo sapi		5.62	31.08	7	3	3	4	3.276E7	148	16.4	7.56
H0YE89	RalBP1-associated Eps domain-containing protein 1 (Fragment) OS=H		5.62	26.23	5	1	1	2	1.791E7	61	6.7	10.27
Q7Z2W4	Zinc finger CCCH-type antiviral protein 1 OS=Homo sapiens GN=ZC3		5.54	5.65	3	4	4	4	1.121E7	902	101.4	8.40
F8W696	Apolipoprotein A-I OS=Homo sapiens GN=APOA1 PE=1 SV=1 - [F8W		5.49	21.63	2	4	4	6	1.466E7	245	27.9	6.13
Q8TB36	Ganglioside-induced differentiation-associated protein 1 OS=Homo sa		5.48	7.26	1	2	2	2	1.645E7	358	41.3	8.34
Q9NUQ2	1-acyl-sn-glycerol-3-phosphate acyltransferase epsilon OS=Homo sap		5.47	11.54	2	3	3	3	4.034E7	364	42.0	9.10
Q8N5P1	Zinc finger CCCH domain-containing protein 8 OS=Homo sapiens GN=		5.45	16.49	2	4	4	6	2.817E7	291	33.6	8.28
B4DUR8	T-complex protein 1 subunit gamma OS=Homo sapiens GN=CCT3 PE		5.45	4.40	2	2	2	2	1.188E7	500	55.6	5.64
Q8TBM8	DnaJ homolog subfamily B member 14 OS=Homo sapiens GN=DNAJ		5.38	7.65	3	3	3	3	1.513E7	379	42.5	8.59
O75569	Interferon-inducible double-stranded RNA-dependent protein kinase a		5.33	12.14	4	3	3	3	2.848E7	313	34.4	8.41
Q07955	Serine/arginine-rich splicing factor 1 OS=Homo sapiens GN=SRSF1 P		5.26	13.31	3	2	2	2	3.647E7	248	27.7	10.36
E5RHW4	Erlin-2 (Fragment) OS=Homo sapiens GN=ERLIN2 PE=1 SV=1 - [E5R		5.26	9.17	4	1	3	3	2.495E7	338	37.7	5.62
Q96CB9	5-methylcytosine rRNA methyltransferase NSUN4 OS=Homo sapiens		5.22	11.72	2	3	3	3	2.012E7	384	43.1	8.18
P10412	Histone H1.4 OS=Homo sapiens GN=HIST1H1E PE=1 SV=2 - [H14_H		5.21	18.26	5	3	4	4	1.429E7	219	21.9	11.03
P13747	HLA class I histocompatibility antigen, alpha chain E OS=Homo sapie		5.21	11.17	9	2	3	3	2.743E7	358	40.1	5.95
P60891	Ribose-phosphate pyrophosphokinase 1 OS=Homo sapiens GN=PRPS		5.19	11.95	8	3	3	4	1.072E7	318	34.8	6.98
A0A0A0MS14	Immunoglobulin heavy variable 1-45 OS=Homo sapiens GN=IGHV1-4		5.15	9.40	1	1	1	2	5.942E7	117	13.5	9.10
P04004	Vitronectin OS=Homo sapiens GN=VTN PE=1 SV=1 - [VTNC_HUMAN		5.12	5.65	1	2	2	4	1.752E8	478	54.3	5.80
O75477	Erlin-1 OS=Homo sapiens GN=ERLIN1 PE=1 SV=1 - [ERLN1_HUMAN		5.08	9.54	2	1	3	3	2.368E7	346	38.9	7.87
K7EM91	Kunitz-type protease inhibitor 2 OS=Homo sapiens GN=SPINT2 PE=1		5.06	15.35	5	3	3	3	1.415E7	202	22.8	7.46
A6NP24	Quinone oxidoreductase (Fragment) OS=Homo sapiens GN=CRYZ PE		5.05	8.64	3	2	2	2	1.671E7	243	26.0	8.88
O76003	Glutaredoxin-3 OS=Homo sapiens GN=GLRX3 PE=1 SV=2 - [GLRX3_		5.00	16.42	1	4	4	5	1.471E7	335	37.4	5.39
Q9BT78	COP9 signalosome complex subunit 4 OS=Homo sapiens GN=COPS4		4.99	5.91	4	2	2	2	1.083E7	406	46.2	5.83
Q9H444	Charged multivesicular body protein 4b OS=Homo sapiens GN=CHMP		4.91	9.38	1	2	2	2	2.487E7	224	24.9	4.82
P24752	Acetyl-CoA acetyltransferase, mitochondrial OS=Homo sapiens GN=A		4.82	16.86	3	6	6	6	1.824E7	427	45.2	8.85
Q96IZ0	PRKC apoptosis WT1 regulator protein OS=Homo sapiens GN=PAWR		4.81	10.59	1	3	3	3	1.335E7	340	36.5	5.41
U3KQ69	Mitochondrial GTPase 1 OS=Homo sapiens GN=MTG1 PE=3 SV=1 - [		4.76	17.41	4	3	3	3	1.761E7	293	32.6	9.11
P46734	Dual specificity mitogen-activated protein kinase kinase 3 OS=Homo		4.75	13.54	3	3	3	3	3.772E7	347	39.3	7.43
P54619	5'-AMP-activated protein kinase subunit gamma-1 OS=Homo sapiens		4.74	23.26	10	6	6	6	1.332E7	331	37.6	6.92
Q96B26	Exosome complex component RRP43 OS=Homo sapiens GN=EXOSC8		4.73	13.77	1	3	3	3	2.708E7	276	30.0	5.30
K7ES69	Calponin-2 OS=Homo sapiens GN=CNN2 PE=1 SV=1 - [K7ES69_HUM		4.71	14.19	6	2	2	2	2.265E7	148	16.5	9.04
Q5JR95	40S ribosomal protein S8 OS=Homo sapiens GN=RPS8 PE=1 SV=1 -		4.69	10.64	2	2	2	2	1.911E7	188	21.9	10.36
F8W6G5	Aprataxin (Fragment) OS=Homo sapiens GN=APTX PE=1 SV=1 - [F8		4.64	14.65	10	2	2	2	1.078E7	198	22.7	9.79
Q92747	Actin-related protein 2/3 complex subunit 1A OS=Homo sapiens GN=		4.60	10.54	2	3	3	4	1.827E7	370	41.5	8.18
A8MUF7	Hemoglobin subunit epsilon (Fragment) OS=Homo sapiens GN=HBE1		4.59	11.49	10	1	1	2	1.488E8	87	9.5	9.13
A0A087X1K6	Krueppel-like factor 13 OS=Homo sapiens GN=KLF13 PE=1 SV=1 - [		4.49	12.94	2	2	2	2	2.738E7	286	31.2	9.95
Q96GY0	Zinc finger C2HC domain-containing protein 1A OS=Homo sapiens G		4.49	16.92	2	4	4	5	1.150E7	325	35.1	9.82
J3QT54	Cleavage and polyadenylation-specificity factor subunit 7 (Fragment)		4.47	17.16	14	3	3	3	1.061E7	204	22.6	5.00
Q96MG7	Non-structural maintenance of chromosomes element 3 homolog OS=		4.47	11.84	1	3	3	3	1.532E7	304	34.3	9.28
P01857	Immunoglobulin heavy constant gamma 1 OS=Homo sapiens GN=IG		4.45	16.36	6	4	4	7	1.534E8	330	36.1	8.19
A0A0C4DFV9	Protein SET OS=Homo sapiens GN=SET PE=1 SV=1 - [A0A0C4DFV9_		4.44	13.91	4	3	3	3	2.343E7	266	31.1	4.23
O00151	PDZ and LIM domain protein 1 OS=Homo sapiens GN=PDLIM1 PE=1		4.42	15.20	1	3	3	3	3.327E7	329	36.0	7.02
H0Y2V1	Microtubule-associated protein (Fragment) OS=Homo sapiens GN=M		4.41	6.05	6	2	2	2	1.167E7	463	48.6	10.11
P62753	40S ribosomal protein S6 OS=Homo sapiens GN=RPS6 PE=1 SV=1 -		4.37	8.03	3	2	2	3	5.185E7	249	28.7	10.84

Q9H1D9	DNA-directed RNA polymerase III subunit RPC6 OS=Homo sapiens G	4.37	7.28	1	2	2	2	2.392E7	316	35.7	6.11
Q9Y2S7	Polymerase delta-interacting protein 2 OS=Homo sapiens GN=POLDI	4.36	12.50	2	4	4	4	9.228E6	368	42.0	8.63
H7BY36	RNA-binding protein EWS (Fragment) OS=Homo sapiens GN=EWSR1	4.34	9.42	5	2	2	2	2.900E7	308	32.2	9.82
Q5TBH9	Chromosome 1 open reading frame 131, isoform CRA_a OS=Homo sa	4.33	8.84	3	3	3	3	1.173E7	249	27.9	9.54
H3BR27	RNA-binding motif protein, X chromosome OS=Homo sapiens GN=RB	4.27	28.21	8	2	2	2	2.469E7	78	8.6	5.49
H3BPZ1	Very-long-chain (3R)-3-hydroxyacyl-CoA dehydratase OS=Homo sapie	4.20	10.39	5	3	3	3	1.073E7	337	40.0	8.97
P19338	Nucleolin OS=Homo sapiens GN=NCL PE=1 SV=3 - [NUCL_HUMAN]	4.18	7.18	6	4	4	4	1.069E7	710	76.6	4.70
Q9BXW7	Haloacid dehalogenase-like hydrolase domain-containing 5 OS=Homo	4.18	6.15	1	2	2	2	1.111E7	423	46.3	8.13
Q96J01	THO complex subunit 3 OS=Homo sapiens GN=THOC3 PE=1 SV=1 -	4.15	9.69	4	4	4	5	3.231E7	351	38.7	6.09
O75822	Eukaryotic translation initiation factor 3 subunit J OS=Homo sapiens (	4.13	9.30	1	2	2	2	1.432E7	258	29.0	4.83
P35226	Polycomb complex protein BMI-1 OS=Homo sapiens GN=BMI1 PE=1	4.11	7.67	8	3	3	3	1.111E7	326	36.9	8.63
Q13303	Voltage-gated potassium channel subunit beta-2 OS=Homo sapiens G	4.10	14.44	12	4	4	4	2.413E7	367	41.0	9.00
A0A0D9SFS3	2-oxoglutarate dehydrogenase, mitochondrial OS=Homo sapiens GN=	4.09	2.30	7	3	3	3	7.285E6	1001	113.2	7.08
E7EW18	DNA polymerase beta (Fragment) OS=Homo sapiens GN=POLB PE=1	4.09	6.43	6	2	2	2	1.260E7	280	31.3	9.16
P35579	Myosin-9 OS=Homo sapiens GN=MYH9 PE=1 SV=4 - [MYH9_HUMAN	4.05	4.23	1	6	6	6	1.941E7	1960	226.4	5.60
C9JXB8	60S ribosomal protein L24 OS=Homo sapiens GN=RPL24 PE=1 SV=1	4.02	18.18	3	2	2	2	9.490E6	121	14.4	11.31
H7BXD8	Galectin (Fragment) OS=Homo sapiens GN=LGALS8 PE=1 SV=1 - [H	4.02	11.43	8	2	2	2	1.114E7	175	19.6	9.63
C9JEV6	N-acetyl-D-glucosamine kinase OS=Homo sapiens GN=NAGK PE=1 S\	4.00	9.90	8	2	2	2	1.385E7	293	32.0	6.99
Q7RTS7	Keratin, type II cytoskeletal 74 OS=Homo sapiens GN=KRT74 PE=1 S	3.95	6.62	9	2	4	5	3.951E7	529	57.8	7.71
P36551	Oxygen-dependent coproporphyrinogen-III oxidase, mitochondrial OS	3.90	4.41	2	2	2	2	1.404E7	454	50.1	8.25
C9JEU5	Fibrinogen gamma chain OS=Homo sapiens GN=FGG PE=1 SV=1 - [(	3.85	11.01	5	3	3	4	2.678E7	445	50.3	6.09
P07477	Trypsin-1 OS=Homo sapiens GN=PRSS1 PE=1 SV=1 - [TRY1_HUMAN	3.85	11.34	12	2	2	2	9.221E7	247	26.5	6.51
B3KPJ4	Polyhomeotic-like protein 2 OS=Homo sapiens GN=PHC2 PE=1 SV=1	3.85	6.25	3	3	3	3	1.048E7	464	50.4	8.05
F8VS81	Twinfilin-1 (Fragment) OS=Homo sapiens GN=TWF1 PE=1 SV=1 - [F	3.84	8.93	5	2	2	2	1.389E7	291	33.4	6.46
Q7Z4F1	Low-density lipoprotein receptor-related protein 10 OS=Homo sapiens	3.83	3.37	2	2	2	2	1.775E7	713	76.1	5.52
P30419	Glycylpeptide N-tetradecanoyltransferase 1 OS=Homo sapiens GN=NI	3.82	4.84	3	2	2	2	2.124E7	496	56.8	7.80
A0A087WTZ5	UBX domain-containing protein 1 OS=Homo sapiens GN=UBXN1 PE=	3.82	11.76	5	2	2	2	1.274E7	238	27.0	7.96
Q9NZL4	Hsp70-binding protein 1 OS=Homo sapiens GN=HSPBP1 PE=1 SV=1	3.82	15.75	6	4	4	5	2.572E7	362	39.4	5.21
O94907	Dickkopf-related protein 1 OS=Homo sapiens GN=DKK1 PE=1 SV=1 -	3.77	3.76	1	1	1	2	2.019E6	266	28.7	8.40
Q9UBM7	7-dehydrocholesterol reductase OS=Homo sapiens GN=DHCR7 PE=1	3.76	8.42	8	3	3	3	2.101E7	475	54.5	8.70
E9PQW0	Peroxisomal membrane protein PEX16 OS=Homo sapiens GN=PEX16	3.74	26.67	4	2	2	2	1.476E7	60	6.8	8.69
A2IDA3	DNA-3-methyladenine glycosylase (Fragment) OS=Homo sapiens GN=	3.74	9.56	2	2	2	2	2.853E7	251	27.3	9.03
H7C1W2	Isocitrate dehydrogenase [NAD] subunit gamma, mitochondrial (Frag	3.70	9.05	5	2	2	2	6.573E6	199	22.4	8.18
F5H8H2	Mevalonate kinase OS=Homo sapiens GN=MVK PE=1 SV=1 - [F5H8H	3.66	3.49	3	1	1	1	3.035E7	344	37.1	7.08
E7EM64	COP9 signalosome complex subunit 6 OS=Homo sapiens GN=COPS6	3.61	6.75	2	2	2	3	1.044E7	326	36.0	5.73
A0A1W2PQS1	26S proteasome regulatory subunit 7 OS=Homo sapiens GN=PSMC2	3.55	6.25	2	2	2	2	9.289E6	400	44.8	6.60
Q8N6M0	OTU domain-containing protein 6B OS=Homo sapiens GN=OTUD6B P	3.52	11.95	3	2	2	2	1.710E7	293	33.8	6.05
E7EQR4	Ezrin OS=Homo sapiens GN=EZR PE=1 SV=3 - [E7EQR4_HUMAN]	3.43	8.19	6	5	5	5	2.363E7	586	69.3	6.16
Q5T911	Mediator of RNA polymerase II transcription subunit 4 (Fragment) OS	3.37	7.14	2	1	1	2	3.066E7	238	27.1	5.63
P02671	Fibrinogen alpha chain OS=Homo sapiens GN=FGA PE=1 SV=2 - [FIE	3.20	1.96	1	1	1	1	3.854E7	866	94.9	6.01
B1AN99	Trypsin-3 (Fragment) OS=Homo sapiens GN=PRSS3 PE=1 SV=8 - [B	3.04	11.30	2	2	2	2	2.408E8	177	19.3	6.07
A0A0D9SFB3	ATP-dependent RNA helicase DDX3X OS=Homo sapiens GN=DDX3X F	2.95	5.31	13	2	3	3	1.694E7	640	70.8	7.36
P78406	mRNA export factor OS=Homo sapiens GN=RAE1 PE=1 SV=1 - [RAE	2.92	12.50	3	4	4	4	2.961E7	368	40.9	7.83
F5GWG3	Retinoic acid-induced protein 3 (Fragment) OS=Homo sapiens GN=GI	2.91	4.76	2	1	1	1	3.030E7	273	30.5	9.03
P48651	Phosphatidylserine synthase 1 OS=Homo sapiens GN=PTDSS1 PE=1	2.91	2.75	1	1	1	1	2.067E7	473	55.5	8.43
S4R3D5	Aldo-keto reductase family 1 member C3 (Fragment) OS=Homo sapie	2.82	5.81	11	2	2	2	1.474E7	155	17.6	6.54
P04003	C4b-binding protein alpha chain OS=Homo sapiens GN=C4BPA PE=1	2.82	2.01	1	1	1	1	4.876E7	597	67.0	7.30
B0V3J0	tRNA-splicing endonuclease subunit Sen34 (Fragment) OS=Homo sap	2.79	13.22	5	2	2	2	1.115E7	242	26.1	9.35
A0A087WXQ5	TAR DNA-binding protein 43 (Fragment) OS=Homo sapiens GN=TARI	2.79	16.50	18	3	3	3	6.298E6	200	22.9	6.55
Q9NR45	Sialic acid synthase OS=Homo sapiens GN=NANS PE=1 SV=2 - [SIAS	2.77	8.91	3	2	2	2	2.308E7	359	40.3	6.74
M0R0V2	Deoxyhypusine synthase (Fragment) OS=Homo sapiens GN=DHPS PE	2.76	8.18	3	1	1	1	1.072E7	159	17.9	9.83

Q9BWD1	Acetyl-CoA acetyltransferase, cytosolic OS=Homo sapiens GN=ACAT2	2.74	2.77	1	1	1	2	1.966E7	397	41.3	6.92
F8WE98	Filamin-A (Fragment) OS=Homo sapiens GN=FLNA PE=1 SV=2 - [F8W	2.73	1.82	7	1	1	1	1.713E7	604	66.6	8.95
Q8WUZ0	B-cell CLL/lymphoma 7 protein family member C OS=Homo sapiens G	2.72	10.14	8	2	2	2	1.961E7	217	23.5	5.20
Q15527	Surfeit locus protein 2 OS=Homo sapiens GN=SURF2 PE=1 SV=3 - [S	2.71	7.42	1	1	1	2	6.983E7	256	29.6	9.22
K7ELP0	Tropomyosin alpha-4 chain (Fragment) OS=Homo sapiens GN=TPM4	2.70	15.94	1	1	1	1	1.357E7	69	8.0	5.01
O14828	Secretory carrier-associated membrane protein 3 OS=Homo sapiens G	2.67	4.61	1	1	1	1	2.178E7	347	38.3	7.64
F5GZQ3	Trifunctional enzyme subunit beta, mitochondrial OS=Homo sapiens G	2.63	1.96	2	1	1	1	7.825E6	459	49.6	9.42
P50148	Guanine nucleotide-binding protein G(q) subunit alpha OS=Homo sap	2.61	9.19	3	1	3	3	1.609E7	359	42.1	5.68
P29992	Guanine nucleotide-binding protein subunit alpha-11 OS=Homo sapie	2.61	7.80	3	1	3	3	1.580E7	359	42.1	5.69
P50402	Emerin OS=Homo sapiens GN=EMD PE=1 SV=1 - [EMD_HUMAN]	2.61	13.78	2	3	3	3	9.242E6	254	29.0	5.50
G3V5M0	DNA-(apurinic or apyrimidinic site) lyase (Fragment) OS=Homo sapier	2.60	16.05	8	2	2	2	1.720E7	162	17.8	5.27
Q5VU10	Ribonuclease P protein subunit p30 (Fragment) OS=Homo sapiens GN	2.60	13.68	3	3	3	3	9.386E6	212	23.3	8.97
Q9H1X3	DnaJ homolog subfamily C member 25 OS=Homo sapiens GN=DNAJC	2.59	2.78	1	1	1	1	1.658E7	360	42.4	9.13
B1AHF3	NADH-cytochrome b5 reductase 3 (Fragment) OS=Homo sapiens GN=	2.56	7.48	2	1	1	1		147	16.7	9.73
P62701	40S ribosomal protein S4, X isoform OS=Homo sapiens GN=RPS4X PE	2.56	12.55	4	3	3	3	1.321E7	263	29.6	10.15
P53985	Monocarboxylate transporter 1 OS=Homo sapiens GN=SLC16A1 PE=1	2.55	6.40	3	2	2	2	2.811E7	500	53.9	8.66
O94992	Protein HEXIM1 OS=Homo sapiens GN=HEXIM1 PE=1 SV=1 - [HEXI1	2.53	8.64	1	2	2	2	1.734E7	359	40.6	4.89
Q96EY1	DnaJ homolog subfamily A member 3, mitochondrial OS=Homo sapie	2.53	10.83	1	3	3	3	3.810E7	480	52.5	9.26
H0YJH7	Lamina-associated polypeptide 2, isoforms beta/gamma (Fragment) O	2.51	6.75	3	1	1	1	4.493E6	237	26.9	9.31
Q9Y6Z7	Collectin-10 OS=Homo sapiens GN=COLEC10 PE=1 SV=2 - [COL10_H	2.50	3.61	1	1	1	1	6.433E7	277	30.7	7.33
P16144	Integrin beta-4 OS=Homo sapiens GN=ITGB4 PE=1 SV=5 - [ITB4_HU	2.49	0.66	1	1	1	1	1.377E7	1822	202.0	6.09
H0Y698	Acyl-coenzyme A thioesterase 8 (Fragment) OS=Homo sapiens GN=A	2.49	21.62	7	1	1	1	2.508E7	74	8.4	9.10
A6NCS6	Uncharacterized protein C2orf72 OS=Homo sapiens GN=C2orf72 PE=	2.49	8.14	1	1	1	1	1.330E7	295	30.5	8.34
Q12906	Interleukin enhancer-binding factor 3 OS=Homo sapiens GN=ILF3 PE	2.49	2.57	1	2	2	2	1.633E7	894	95.3	8.76
M0QX76	40S ribosomal protein S16 (Fragment) OS=Homo sapiens GN=RPS16	2.45	20.00	6	1	1	1	1.427E7	50	5.6	9.63
Q8IYB3	Serine/arginine repetitive matrix protein 1 OS=Homo sapiens GN=SR	2.45	3.76	2	1	1	1	2.723E7	904	102.3	11.84
P62851	40S ribosomal protein S25 OS=Homo sapiens GN=RPS25 PE=1 SV=1	2.45	11.20	1	1	1	1	2.014E7	125	13.7	10.11
Q8IVS2	Malonyl-CoA-acyl carrier protein transacylase, mitochondrial OS=Hom	2.42	2.82	1	1	1	1	1.741E7	390	42.9	8.72
P82675	28S ribosomal protein S5, mitochondrial OS=Homo sapiens GN=MRPS	2.40	4.65	1	2	2	2	1.383E7	430	48.0	9.92
F2Z2A4	Deoxynucleotidyltransferase terminal-interacting protein 1 (Fragment)	2.39	6.21	4	1	1	1	1.164E7	145	16.4	9.61
P46109	Crk-like protein OS=Homo sapiens GN=CRKL PE=1 SV=1 - [CRKL_HU	2.38	8.91	1	2	2	2	1.550E7	303	33.8	6.74
A0A0A0MRQ5	Peroxiredoxin-1 OS=Homo sapiens GN=PRDX1 PE=1 SV=1 - [A0A0A0	2.38	11.34	4	1	1	1	1.059E7	97	10.7	8.72
F8WBC0	Ras-related protein Rap-1b (Fragment) OS=Homo sapiens GN=RAP1B	2.38	34.38	17	1	1	1	1.054E7	32	3.5	9.42
D6RAD4	Cyclin-dependent kinase 7 OS=Homo sapiens GN=CDK7 PE=1 SV=1	2.36	9.39	4	2	2	2	9.661E6	309	34.6	8.28
P06733	Alpha-enolase OS=Homo sapiens GN=ENO1 PE=1 SV=2 - [ENOA_HU	2.36	6.68	2	3	3	3	6.809E6	434	47.1	7.39
O94776	Metastasis-associated protein MTA2 OS=Homo sapiens GN=MTA2 PE	2.33	1.50	1	1	1	1	6.593E6	668	75.0	9.66
Q6P087	RNA pseudouridylate synthase domain-containing protein 3 OS=Homo	2.32	6.84	5	2	2	2	2.052E7	351	38.4	10.32
Q07021	Complement component 1 Q subcomponent-binding protein, mitocho	2.32	20.92	3	4	4	4	1.923E7	282	31.3	4.84
Q9NXC2	Glucose-fructose oxidoreductase domain-containing protein 1 OS=Ho	2.32	3.08	1	1	1	1	6.407E6	390	43.1	5.92
Q9H4I3	TraB domain-containing protein OS=Homo sapiens GN=TRABD PE=1	2.31	2.66	2	1	1	1	1.377E7	376	42.3	8.00
A0A087WVI1	PDZ and LIM domain protein 2 OS=Homo sapiens GN=PDLIM2 PE=1	2.31	6.15	9	2	2	2	9.759E6	325	34.7	8.47
H0Y4R1	Inosine-5'-monophosphate dehydrogenase 2 (Fragment) OS=Homo s	2.30	2.34	2	1	1	1	7.769E6	470	51.0	8.18
Q5QP23	RNA-binding protein 39 (Fragment) OS=Homo sapiens GN=RBM39 PE	2.28	6.49	5	1	1	1	3.308E7	231	27.1	11.41
Q9Y3B9	RRP15-like protein OS=Homo sapiens GN=RRP15 PE=1 SV=2 - [RRP1	2.28	3.90	1	1	1	1	2.451E7	282	31.5	5.52
E9PHS0	LanC-like protein 1 (Fragment) OS=Homo sapiens GN=LANCL1 PE=1	2.27	15.82	3	3	3	3	1.875E7	196	22.0	8.15
D6RDK6	OCIA domain-containing protein 1 (Fragment) OS=Homo sapiens GN=	2.27	12.29	9	2	2	2	8.758E6	179	20.0	9.06
P67936	Tropomyosin alpha-4 chain OS=Homo sapiens GN=TPM4 PE=1 SV=3	2.26	7.26	22	1	2	2	3.469E7	248	28.5	4.69
K7ERG3	Tropomyosin alpha-4 chain (Fragment) OS=Homo sapiens GN=TPM4	2.26	14.12	22	1	2	2	3.347E7	170	19.3	4.79
C9JQD0	Quinone oxidoreductase-like protein 1 (Fragment) OS=Homo sapiens	2.26	11.21	8	1	1	1	9.314E6	107	11.7	6.05
Q13123	Protein Red OS=Homo sapiens GN=IK PE=1 SV=3 - [RED_HUMAN]	2.25	1.62	1	1	1	1	8.049E6	557	65.6	6.64
B9A008	U3 small nucleolar ribonucleoprotein protein IMP4 (Fragment) OS=Ho	2.25	5.81	4	1	1	1	1.113E7	172	19.6	9.70

F5H0V9	Germ cell-specific gene 1 protein (Fragment) OS=Homo sapiens GN=	2.24	7.20	2	1	1	1	4.085E8	236	26.1	6.93
A0A0U1RQL8	Gelsolin (Fragment) OS=Homo sapiens GN=GSN PE=1 SV=1 - [A0A0	2.23	4.74	5	1	1	1	1.146E7	232	26.3	7.08
Q9P258	Protein RCC2 OS=Homo sapiens GN=RCC2 PE=1 SV=2 - [RCC2_HUM	2.23	3.64	1	1	1	1	1.695E8	522	56.0	8.78
P14091	Cathepsin E OS=Homo sapiens GN=CTSE PE=1 SV=2 - [CATE_HUMA	2.23	2.00	1	1	1	1	3.621E7	401	43.3	4.86
H3BV22	Serine/threonine-protein phosphatase (Fragment) OS=Homo sapiens	2.22	16.26	4	2	3	3	2.925E7	203	23.1	5.12
H0Y6Y4	Ubiquitin carboxyl-terminal hydrolase isozyme L5 (Fragment) OS=Ho	2.21	5.05	5	1	1	1	9.715E6	218	25.4	5.71
Q9NWB6	Arginine and glutamate-rich protein 1 OS=Homo sapiens GN=ARGLU1	2.21	8.06	1	3	3	3	1.518E7	273	33.2	10.35
K7EK18	Septin-9 (Fragment) OS=Homo sapiens GN=SEPT9 PE=1 SV=1 - [K7	2.20	5.13	3	1	1	1	1.245E7	195	22.2	9.36
F5GX09	Protein FAM76B OS=Homo sapiens GN=FAM76B PE=1 SV=1 - [F5GX	2.20	3.25	2	1	1	1	2.159E7	338	38.6	9.25
Q9H5Q4	Dimethyladenosine transferase 2, mitochondrial OS=Homo sapiens G	2.20	8.08	1	3	3	3	1.718E7	396	45.3	9.19
P26373	60S ribosomal protein L13 OS=Homo sapiens GN=RPL13 PE=1 SV=4	2.19	19.91	3	4	4	4	1.390E7	211	24.2	11.65
P04818	Thymidylate synthase OS=Homo sapiens GN=TYMS PE=1 SV=3 - [TY	2.18	3.51	1	1	1	1	5.418E6	313	35.7	7.01
F5GYN4	Ubiquitin thioesterase OTUB1 OS=Homo sapiens GN=OTUB1 PE=1 S	2.17	9.96	6	2	2	2	1.772E7	241	28.0	5.29
Q96BK5	PIN2/TERF1-interacting telomerase inhibitor 1 OS=Homo sapiens GN=	2.16	7.01	4	2	2	2	1.070E7	328	37.0	9.60
O95900	Probable tRNA pseudouridine synthase 2 OS=Homo sapiens GN=TRU	2.14	3.63	1	1	1	1		331	36.7	8.98
Q5TDE7	E3 ubiquitin-protein ligase RNF220 (Fragment) OS=Homo sapiens GN	2.14	3.86	3	1	1	1	1.012E7	259	29.3	5.53
P17858	ATP-dependent 6-phosphofructokinase, liver type OS=Homo sapiens	2.14	2.44	2	1	1	1	1.315E7	780	85.0	7.50
G3V4X8	SNW domain-containing protein 1 OS=Homo sapiens GN=SNW1 PE=	2.14	2.67	3	1	1	1	8.798E6	374	43.3	9.70
E9PQX8	Mitochondrial fission factor (Fragment) OS=Homo sapiens GN=MFF P	2.13	15.94	4	1	1	1	1.028E7	69	7.6	5.91
P00558	Phosphoglycerate kinase 1 OS=Homo sapiens GN=PGK1 PE=1 SV=3	2.12	3.60	2	1	1	1	1.127E7	417	44.6	8.10
A0A087X1J2	UPF0472 protein C16orf72 (Fragment) OS=Homo sapiens GN=C16orf	2.12	12.00	2	1	1	1	2.082E7	150	17.1	10.27
J3QL05	Serine/arginine-rich-splicing factor 2 (Fragment) OS=Homo sapiens G	2.12	13.08	3	1	1	2	2.794E7	130	15.1	10.96
F8VVM2	Phosphate carrier protein, mitochondrial OS=Homo sapiens GN=SLC2	2.12	3.70	2	1	1	1	2.860E7	324	36.1	9.26
V9GYR2	Sodium/potassium-transporting ATPase subunit beta (Fragment) OS=	2.12	13.85	2	2	2	2	1.818E7	130	15.1	6.16
Q9H7B2	Ribosome production factor 2 homolog OS=Homo sapiens GN=RPF2	2.12	12.09	2	4	4	4	8.508E6	306	35.6	9.99
K7EML3	Chromosome 19 open reading frame 66 (Fragment) OS=Homo sapien	2.11	25.35	2	1	1	1	5.317E6	71	8.1	4.83
F2Z2Y4	Pyridoxal kinase OS=Homo sapiens GN=PDXK PE=1 SV=1 - [F2Z2Y4_	2.11	6.25	2	1	1	1	1.331E7	272	30.6	6.65
P51570	Galactokinase OS=Homo sapiens GN=GALK1 PE=1 SV=1 - [GALK1_H	2.10	3.32	1	1	1	1	3.119E7	392	42.2	6.46
E9PQA5	Zinc finger protein-like 1 (Fragment) OS=Homo sapiens GN=ZFPL1 P	2.10	4.65	4	1	1	1	1.283E7	172	18.9	5.49
O76075	DNA fragmentation factor subunit beta OS=Homo sapiens GN=DFFB	2.09	5.33	2	1	1	1	1.553E7	338	39.1	9.00
J3QL14	V-type proton ATPase subunit d 1 (Fragment) OS=Homo sapiens GN=	2.09	5.60	4	1	1	1	2.330E7	232	26.8	4.94
Q8N954	G patch domain-containing protein 11 OS=Homo sapiens GN=GPATC	2.07	3.47	2	1	1	1	1.109E7	259	30.2	5.24
M0QYK9	DNA-binding death effector domain-containing protein 2 (Fragment) (	2.06	9.40	3	1	1	1	6.035E6	117	13.2	5.52
Q8TF74	WAS/WASL-interacting protein family member 2 OS=Homo sapiens G	2.05	4.32	1	1	1	1	1.450E7	440	46.3	10.93
H3BLU7	Aflatoxin B1 aldehyde reductase member 2 (Fragment) OS=Homo sa	2.05	4.78	3	1	1	1	2.049E7	314	34.7	7.18
C9JE01	WD repeat domain phosphoinositide-interacting protein 4 (Fragment)	2.04	15.19	15	2	2	2	8.611E6	158	18.0	7.94
C9J1G2	DnaJ homolog subfamily B member 2 (Fragment) OS=Homo sapiens	2.03	9.84	6	1	1	1	9.702E6	122	13.8	5.39
H3BQQ6	Protein FAM192A (Fragment) OS=Homo sapiens GN=FAM192A PE=1	2.02	15.63	9	1	1	1	9.125E6	96	11.8	4.75
G3V1C1	1,5-anhydro-D-fructose reductase OS=Homo sapiens GN=AKR1E2 PE	2.02	3.94	3	1	1	1	1.233E7	203	23.1	6.20
M0QX71	Glutamate-rich WD repeat-containing protein 1 (Fragment) OS=Homo	2.02	3.95	2	1	1	1	4.571E6	228	25.5	4.86
I3L1Q5	Pre-rRNA-processing protein TSR1 homolog OS=Homo sapiens GN=T	2.01	4.08	2	1	1	1	1.606E7	368	41.4	9.50
Q13724	Mannosyl-oligosaccharide glucosidase OS=Homo sapiens GN=MOGS F	2.01	1.43	1	1	1	1	6.569E6	837	91.9	8.90
A0A0A0MRR5	Uracil phosphoribosyltransferase homolog OS=Homo sapiens GN=UP	2.01	10.32	4	3	3	3	1.782E7	281	30.7	6.15
P0C1Z6	TCF3 fusion partner OS=Homo sapiens GN=TFPT PE=1 SV=1 - [TFPT	2.00	5.93	1	1	1	1	1.326E7	253	28.3	5.21
A0A0M3HER1	LIM and senescent cell antigen-like-containing domain protein 1 (Frag	1.99	5.74	3	1	1	1	2.479E7	209	24.4	9.00
S4R3W8	Calcium uniporter protein, mitochondrial OS=Homo sapiens GN=MCU	1.99	16.67	4	1	1	1	5.936E6	54	5.3	11.82
P36542	ATP synthase subunit gamma, mitochondrial OS=Homo sapiens GN=	1.98	4.03	1	1	1	1	1.228E7	298	33.0	9.22
E9PPT8	Coiled-coil domain-containing protein 84 OS=Homo sapiens GN=CCD	1.98	6.76	3	1	1	1	1.237E7	148	17.2	9.13
Q9UJK0	Ribosome biogenesis protein TSR3 homolog OS=Homo sapiens GN=T	1.97	2.56	1	1	1	1	1.364E7	312	33.6	6.87
F5H6G4	Protein RMD5 homolog B OS=Homo sapiens GN=RMND5B PE=1 SV=	1.96	2.89	2	1	1	1	1.065E7	380	42.7	6.92
C9JRZ8	Aldo-keto reductase family 1 member B15 OS=Homo sapiens GN=AK	1.95	5.06	3	2	2	2	1.811E7	316	36.5	6.70

G3V4X1	26S proteasome regulatory subunit 4 (Fragment) OS=Homo sapiens (	1.95	14.29	10	1	1	1	1.985E7	84	9.1	4.61
H0YI20	2'-5'-oligoadenylate synthase 1 (Fragment) OS=Homo sapiens GN=O/	1.95	15.56	4	3	3	3	2.770E7	302	35.0	9.32
H0YH81	ATP synthase subunit beta (Fragment) OS=Homo sapiens GN=ATP5E	1.94	6.91	4	2	2	2	6.440E6	362	38.2	5.55
Q92522	Histone H1x OS=Homo sapiens GN=H1FX PE=1 SV=1 - [H1X_HUMAI	1.94	4.69	1	1	1	1	1.422E7	213	22.5	10.76
H7BZN1	Choline-phosphate cytidylyltransferase A (Fragment) OS=Homo sapie	1.94	8.76	6	1	1	1	8.105E6	137	15.8	6.28
Q8N5G2	Macoilin OS=Homo sapiens GN=TMEM57 PE=1 SV=1 - [MACOI_HUM	1.93	1.36	1	1	1	1	6.003E6	664	76.1	9.07
Q5TBH0	Serine/threonine-protein kinase Nek6 (Fragment) OS=Homo sapiens (	1.93	4.00	7	1	1	1	3.210E6	200	22.6	7.03
G5EA42	Tropomodulin 2 (Neuronal), isoform CRA_a OS=Homo sapiens GN=TI	1.93	5.21	3	1	2	2	2.258E7	307	34.5	6.86
Q9Y240	C-type lectin domain family 11 member A OS=Homo sapiens GN=CLE	1.93	3.41	1	1	1	1		323	35.7	5.16
H0Y711	Methylosome protein 50 (Fragment) OS=Homo sapiens GN=WDR77 F	1.92	15.46	2	3	3	3	1.934E7	291	31.1	4.89
H0YJN9	Legumain (Fragment) OS=Homo sapiens GN=LGMN PE=1 SV=1 - [H	1.92	18.68	4	1	1	1	4.830E7	91	9.8	8.51
Q5QPP9	UDP-glucose 4-epimerase (Fragment) OS=Homo sapiens GN=GALE P	1.91	9.26	4	1	1	1	8.667E6	108	12.0	6.54
Q5VZC3	Fructose-1,6-bisphosphatase 1 (Fragment) OS=Homo sapiens GN=FE	1.91	7.95	2	1	1	1	1.542E7	151	16.7	6.16
Q9UNE7	E3 ubiquitin-protein ligase CHIP OS=Homo sapiens GN=STUB1 PE=1	1.89	6.27	2	1	2	2	4.409E7	303	34.8	5.87
F5H282	T-complex protein 1 subunit alpha OS=Homo sapiens GN=TCP1 PE=1	1.88	3.31	3	1	1	1	4.836E6	332	36.4	7.02
Q96MX6	WD repeat-containing protein 92 OS=Homo sapiens GN=WDR92 PE=	1.88	8.96	3	2	2	2	1.371E7	357	39.7	8.09
A0A0G2JQ92	Phosphatidate cytidylyltransferase, mitochondrial (Fragment) OS=Hor	1.87	11.44	3	2	2	2	1.820E7	236	26.7	8.07
Q5T9P8	Pre-mRNA-splicing factor 18 (Fragment) OS=Homo sapiens GN=PRPF	1.87	6.59	3	1	1	1	1.812E7	167	19.4	4.91
K7EQ02	DAZ-associated protein 1 (Fragment) OS=Homo sapiens GN=DAZAP1	1.86	4.89	4	1	1	1	1.729E7	327	35.0	7.85
H0Y5R6	Uroporphyrinogen decarboxylase (Fragment) OS=Homo sapiens GN=	1.86	3.07	3	1	1	1	1.677E7	228	25.4	5.31
C9J840	Mitogen-activated protein kinase kinase kinase kinase 4 OS=Homo sa	1.85	2.03	15	2	2	2	4.526E6	987	113.5	6.80
F8W914	Reticulon OS=Homo sapiens GN=RTN4 PE=1 SV=1 - [F8W914_HUM/	1.85	4.06	1	1	1	1	8.700E6	345	37.1	4.77
Q9H939	Proline-serine-threonine phosphatase-interacting protein 2 OS=Homo	1.85	6.29	3	3	3	3	6.456E6	334	38.8	8.48
Q8NC51	Plasminogen activator inhibitor 1 RNA-binding protein OS=Homo sapi	1.84	1.96	1	1	1	1	6.268E6	408	44.9	8.65
P29279	Connective tissue growth factor OS=Homo sapiens GN=CTGF PE=1 S	1.83	7.45	1	2	2	2	6.011E6	349	38.1	8.00
H7BZK6	Ubiquitin carboxyl-terminal hydrolase 46 OS=Homo sapiens GN=USP4	1.82	7.67	3	2	2	3	1.586E7	339	39.5	7.36
Q6PK18	2-oxoglutarate and iron-dependent oxygenase domain-containing pro	1.82	6.27	3	2	2	2	5.091E6	319	35.6	8.18
D6R967	Inorganic pyrophosphatase 2, mitochondrial (Fragment) OS=Homo sa	1.82	6.81	3	1	1	1	1.151E7	191	21.4	6.79
A0A0A0MTD5	Calcium uptake protein 2, mitochondrial (Fragment) OS=Homo sapier	1.82	9.75	2	2	2	2	6.445E6	236	26.1	9.07
Q8N0Y2	Zinc finger protein 444 OS=Homo sapiens GN=ZNF444 PE=1 SV=1 -	1.81	4.59	1	1	1	1	1.245E7	327	35.2	8.56
P27169	Serum paraoxonase/arylesterase 1 OS=Homo sapiens GN=PON1 PE=	1.81	4.51	1	1	1	1		355	39.7	5.22
Q9Y2W1	Thyroid hormone receptor-associated protein 3 OS=Homo sapiens GN	1.80	1.05	1	1	1	1	5.833E6	955	108.6	10.15
O75509	Tumor necrosis factor receptor superfamily member 21 OS=Homo sa	1.78	2.14	1	1	1	1	4.937E6	655	71.8	7.83
A0A087WY55	Chromosome 6 open reading frame 55, isoform CRA_b OS=Homo sap	1.78	6.43	3	2	2	2	8.524E6	280	31.1	6.44
Q5T0A6	Dehydrodolichyl diphosphate synthase complex subunit DHDDS (Frag	1.77	6.51	2	1	1	1	4.322E6	169	19.6	9.32
P46781	40S ribosomal protein S9 OS=Homo sapiens GN=RPS9 PE=1 SV=3 -	1.77	5.67	1	1	1	1	1.071E7	194	22.6	10.65
R4GND3	Pleckstrin homology-like domain family A member 1 OS=Homo sapier	1.77	3.46	2	1	1	1	5.545E6	260	29.6	9.20
F2Z3J9	Prostaglandin reductase 1 (Fragment) OS=Homo sapiens GN=PTGR1	1.76	8.47	2	1	1	1	7.298E6	118	13.4	5.55
F5H3K2	Uncharacterized protein C12orf43 (Fragment) OS=Homo sapiens GN=	1.76	5.32	5	1	1	1	7.442E6	188	20.5	9.09
Q92604	Acyl-CoA:lysophosphatidylglycerol acyltransferase 1 OS=Homo sapien	1.75	9.46	1	2	2	2	6.558E6	370	43.1	8.92
D6RIU4	Vesicular integral-membrane protein VIP36 (Fragment) OS=Homo sa	1.75	5.24	4	1	1	2	1.911E7	191	21.8	6.52
F5H2Z7	Coiled-coil domain-containing protein 92 (Fragment) OS=Homo sapie	1.75	7.29	4	1	1	1	4.613E6	96	11.0	6.52
Q12796	Proline-rich nuclear receptor coactivator 1 OS=Homo sapiens GN=PN	1.75	11.31	2	3	3	4	2.338E7	327	35.2	10.51
H0Y6D8	TATA-box-binding protein (Fragment) OS=Homo sapiens GN=TBP PE	1.74	14.85	3	1	1	1	2.174E7	101	11.6	9.83
S4R3V8	Lipolysis-stimulated lipoprotein receptor OS=Homo sapiens GN=LSR F	1.74	1.83	2	1	1	1	3.353E6	601	66.1	7.02
E9PJW1	Syntaxin-17 OS=Homo sapiens GN=STX17 PE=1 SV=1 - [E9PJW1_HU	1.74	21.95	5	1	1	1	1.411E7	41	5.0	9.32
O43143	Pre-mRNA-splicing factor ATP-dependent RNA helicase DHX15 OS=H	1.74	3.14	1	2	2	2	6.104E6	795	90.9	7.46
Q08211	ATP-dependent RNA helicase A OS=Homo sapiens GN=DHX9 PE=1 S	1.74	0.63	1	1	1	1	2.096E7	1270	140.9	6.84
F5H6T0	Protein ABHD4 (Fragment) OS=Homo sapiens GN=ABHD4 PE=1 SV=	1.73	13.33	2	1	1	1	6.018E6	90	10.1	10.39
C9J168	Phosphoribosyl pyrophosphate synthase-associated protein 1 (Fragme	1.73	12.17	9	2	2	2	9.818E6	230	25.4	6.80
Q5TBG5	Proteasome subunit beta type (Fragment) OS=Homo sapiens GN=PS	1.71	5.24	2	1	1	1	4.948E6	210	22.8	7.39

Q5JVF3	PCI domain-containing protein 2 OS=Homo sapiens GN=PCID2 PE=1	1.69	4.01	1	1	1	1	1.142E7	399	46.0	8.53
H0YMJ6	Apoptosis-enhancing nuclease (Fragment) OS=Homo sapiens GN=AE	1.69	5.03	2	1	1	1	6.993E6	179	19.5	10.23
Q5VV87	Microsomal glutathione S-transferase 3 OS=Homo sapiens GN=MGST	1.69	10.08	3	1	1	1	2.785E6	129	14.4	9.70
E5RHH1	60S ribosomal protein L26-like 1 (Fragment) OS=Homo sapiens GN=F	1.68	23.68	10	1	1	1	9.987E6	38	4.5	11.85
Q96PK6	RNA-binding protein 14 OS=Homo sapiens GN=RBM14 PE=1 SV=2 -	1.68	2.24	1	1	1	1	8.895E6	669	69.4	9.67
Q5JRC6	PHD finger protein 6 OS=Homo sapiens GN=PHF6 PE=1 SV=1 - [Q5J	1.66	4.63	3	2	2	2	1.786E7	324	36.4	9.03
C9J0D1	Histone H2A OS=Homo sapiens GN=H2AFV PE=1 SV=1 - [C9J0D1_H	1.66	7.38	18	1	1	1	9.513E6	122	13.2	9.99
K7ER96	Thioredoxin-like protein 1 (Fragment) OS=Homo sapiens GN=TXNL1	1.66	7.83	3	2	2	2	1.175E7	281	31.4	4.83
C9IZL7	Non-POU domain-containing octamer-binding protein (Fragment) OS=	1.65	10.14	5	2	2	2	1.027E7	207	23.7	9.80
Q6DCA0	AMMECR1-like protein OS=Homo sapiens GN=AMMECR1L PE=1 SV=1	1.65	5.48	1	1	1	1	1.484E7	310	34.5	8.98
H3BPG5	RNA binding protein S1, serine-rich domain, isoform CRA_c OS=Homo	1.65	11.72	6	1	1	1	1.970E7	128	15.1	11.87
Q8NBS9	Thioredoxin domain-containing protein 5 OS=Homo sapiens GN=TXN	1.64	2.08	1	1	1	1	4.145E6	432	47.6	5.97
Q969F1	General transcription factor 3C polypeptide 6 OS=Homo sapiens GN=	1.63	7.04	1	1	1	1	2.483E7	213	24.0	4.21
S4R3M7	GPN-loop GTPase 3 (Fragment) OS=Homo sapiens GN=GPN3 PE=1 S	1.62	52.00	2	1	1	1	6.945E6	25	2.6	9.04
B7Z8R2	Pentatricopeptide repeat-containing protein 2, mitochondrial OS=Hon	1.62	4.35	2	1	1	1	6.259E6	161	18.3	9.17
P14868	Aspartate--tRNA ligase, cytoplasmic OS=Homo sapiens GN=DARS PE=	1.62	3.59	3	2	2	2	4.413E6	501	57.1	6.55
B4DSN5	Tyrosine-protein phosphatase non-receptor type OS=Homo sapiens G	1.61	3.04	2	1	1	1	4.320E6	362	41.2	5.97
C9J8E1	MAP kinase-activated protein kinase 3 (Fragment) OS=Homo sapiens	1.61	6.02	6	2	2	2	1.221E7	332	37.4	6.52
Q8WVY7	Ubiquitin-like domain-containing CTD phosphatase 1 OS=Homo sapie	0.00	4.40	1	1	1	1	3.671E6	318	36.8	6.46
Q9NQG5	Regulation of nuclear pre-mRNA domain-containing protein 1B OS=H	0.00	9.51	4	3	3	3	7.796E6	326	36.9	5.97
O60814	Histone H2B type 1-K OS=Homo sapiens GN=HIST1H2BK PE=1 SV=3	0.00	7.14	16	1	1	1	6.402E6	126	13.9	10.32
O00767	Acyl-CoA desaturase OS=Homo sapiens GN=SCD PE=1 SV=2 - [ACOI	0.00	1.67	1	1	1	1		359	41.5	9.00
Q9Y673	Dolichyl-phosphate beta-glucosyltransferase OS=Homo sapiens GN=A	0.00	3.70	1	1	1	1	7.309E6	324	36.9	9.28
Q8N5I2	Arrestin domain-containing protein 1 OS=Homo sapiens GN=ARRDC1	0.00	3.00	1	1	1	1	4.179E6	433	46.0	7.02
Q9Y679	Ancient ubiquitous protein 1 OS=Homo sapiens GN=AUP1 PE=1 SV=	0.00	3.15	1	1	1	1		476	53.0	8.09
Q4G0S4	Cytochrome P450 27C1 OS=Homo sapiens GN=CYP27C1 PE=1 SV=2	0.00	1.88	1	1	1	1		372	42.6	9.25
Q96FF9	Sororin OS=Homo sapiens GN=CDCA5 PE=1 SV=1 - [CDCA5_HUMAN	0.00	5.56	2	1	1	1	8.513E6	252	27.6	9.61
Q8IWX8	Calcium homeostasis endoplasmic reticulum protein OS=Homo sapier	0.00	1.31	2	1	1	1	3.384E6	916	103.6	9.04
O76071	Probable cytosolic iron-sulfur protein assembly protein CIAO1 OS=Ho	0.00	2.95	1	1	1	1	4.117E6	339	37.8	4.97
P53621	Coatomer subunit alpha OS=Homo sapiens GN=COPA PE=1 SV=2 - [	0.00	0.65	1	1	1	1	3.692E6	1224	138.3	7.66
Q92905	COP9 signalosome complex subunit 5 OS=Homo sapiens GN=COPS5	0.00	3.59	1	1	1	1		334	37.6	6.54
P31689	DnaJ homolog subfamily A member 1 OS=Homo sapiens GN=DNAJA1	0.00	8.06	1	2	2	2	1.288E7	397	44.8	7.08
P26641	Elongation factor 1-gamma OS=Homo sapiens GN=EEF1G PE=1 SV=	0.00	2.29	1	1	1	1	3.255E6	437	50.1	6.67
Q99613	Eukaryotic translation initiation factor 3 subunit C OS=Homo sapiens	0.00	1.10	2	1	1	1	9.451E6	913	105.3	5.68
O00303	Eukaryotic translation initiation factor 3 subunit F OS=Homo sapiens (	0.00	7.84	2	2	2	2	9.335E6	357	37.5	5.45
P29317	Ephrin type-A receptor 2 OS=Homo sapiens GN=EPHA2 PE=1 SV=2 -	0.00	1.33	1	1	1	1		976	108.2	6.23
Q9UN86	Ras GTPase-activating protein-binding protein 2 OS=Homo sapiens G	0.00	3.53	1	1	1	1	1.839E7	482	54.1	5.55
Q9Y5P6	Mannose-1-phosphate guanyltransferase beta OS=Homo sapiens GN=	0.00	5.56	1	1	1	1	1.154E7	360	39.8	6.61
Q9BYB4	Guanine nucleotide-binding protein subunit beta-like protein 1 OS=Ho	0.00	3.98	1	1	1	1	1.710E7	327	35.6	7.97
Q9H4A6	Golgi phosphoprotein 3 OS=Homo sapiens GN=GOLPH3 PE=1 SV=1 -	0.00	5.03	1	1	1	1	7.765E6	298	33.8	6.44
P16401	Histone H1.5 OS=Homo sapiens GN=HIST1H1B PE=1 SV=3 - [H15_H	0.00	9.29	1	1	2	2	1.017E7	226	22.6	10.92
Q6UVM3	Potassium channel subfamily T member 2 OS=Homo sapiens GN=KCI	0.00	1.23	1	1	1	1	3.809E7	1135	130.4	7.28
Q9NX58	Cell growth-regulating nucleolar protein OS=Homo sapiens GN=LYAR	0.00	3.69	1	1	1	1	1.654E7	379	43.6	9.54
O15264	Mitogen-activated protein kinase 13 OS=Homo sapiens GN=MAPK13	0.00	5.21	25	1	2	2	1.349E7	365	42.1	8.38
Q99836	Myeloid differentiation primary response protein MyD88 OS=Homo sa	0.00	7.09	4	1	1	1	1.459E7	296	33.2	6.15
Q9Y6K5	2'-5'-oligoadenylate synthase 3 OS=Homo sapiens GN=OAS3 PE=1 SV	0.00	1.56	1	2	2	2	5.893E6	1087	121.1	8.40
Q86YP4	Transcriptional repressor p66-alpha OS=Homo sapiens GN=GATAD2A	0.00	3.63	1	1	1	1	1.586E7	633	68.0	9.94
Q96AQ6	Pre-B-cell leukemia transcription factor-interacting protein 1 OS=Hom	0.00	1.23	1	1	1	1	3.989E6	731	80.6	5.33
P12004	Proliferating cell nuclear antigen OS=Homo sapiens GN=PCNA PE=1 S	0.00	7.28	1	1	1	1	2.461E7	261	28.8	4.69
Q96HE9	Proline-rich protein 11 OS=Homo sapiens GN=PRR11 PE=1 SV=1 - [F	0.00	5.28	1	1	1	1	1.548E7	360	40.1	10.11
P49207	60S ribosomal protein L34 OS=Homo sapiens GN=RPL34 PE=1 SV=3	0.00	5.98	1	1	1	1		117	13.3	11.47

Q9BZE1	39S ribosomal protein L37, mitochondrial OS=Homo sapiens GN=MRI	0.00	5.91	4	2	2	2	6.968E6	423	48.1	8.59
Q14684	Ribosomal RNA processing protein 1 homolog B OS=Homo sapiens G	0.00	1.45	1	1	1	1	2.385E6	758	84.4	9.76
P08621	U1 small nuclear ribonucleoprotein 70 kDa OS=Homo sapiens GN=SN	0.00	2.52	1	1	1	1	1.042E7	437	51.5	9.94
P23526	Adenosylhomocysteinase OS=Homo sapiens GN=AHCY PE=1 SV=4 -	0.00	1.85	1	1	1	1	4.691E6	432	47.7	6.34
Q9Y512	Sorting and assembly machinery component 50 homolog OS=Homo s	0.00	1.49	1	1	1	1	4.708E6	469	51.9	6.90
Q6P4A7	Sideroflexin-4 OS=Homo sapiens GN=SFXN4 PE=1 SV=1 - [SFXN4_H	0.00	4.45	1	1	1	1	1.384E7	337	38.0	9.19
O43765	Small glutamine-rich tetratricopeptide repeat-containing protein alpha	0.00	3.19	1	1	1	1	6.038E6	313	34.0	4.87
Q86XZ4	Spermatogenesis-associated serine-rich protein 2 OS=Homo sapiens	0.00	2.20	1	1	1	1	3.148E6	545	59.5	8.90
Q96GP6	Scavenger receptor class F member 2 OS=Homo sapiens GN=SCARF2	0.00	1.72	2	1	1	1	1.060E7	870	92.4	8.44
Q96SB4	SRSF protein kinase 1 OS=Homo sapiens GN=SRPK1 PE=1 SV=2 - [S	0.00	2.14	2	1	1	1	3.522E7	655	74.3	6.16
O60687	Sushi repeat-containing protein SRPX2 OS=Homo sapiens GN=SRPX2	0.00	3.44	1	1	1	1	4.760E7	465	52.9	7.25
Q12846	Syntaxin-4 OS=Homo sapiens GN=STX4 PE=1 SV=2 - [STX4_HUMAN	0.00	3.70	1	1	1	1	1.278E7	297	34.2	6.28
Q9Y490	Talin-1 OS=Homo sapiens GN=TLN1 PE=1 SV=3 - [TLN1_HUMAN]	0.00	0.43	1	1	1	1		2541	269.6	6.07
Q92995	Ubiquitin carboxyl-terminal hydrolase 13 OS=Homo sapiens GN=USP1	0.00	1.51	1	1	1	1	2.131E6	863	97.3	5.53
Q502W6	von Willebrand factor A domain-containing protein 3B OS=Homo sapi	0.00	1.16	1	1	1	1	1.210E7	1294	145.7	7.33
O75695	Protein XRP2 OS=Homo sapiens GN=RP2 PE=1 SV=4 - [XRP2_HUMA	0.00	2.29	1	1	1	1	1.334E7	350	39.6	5.12
Q8WU90	Zinc finger CCCH domain-containing protein 15 OS=Homo sapiens GN	0.00	6.34	2	2	2	2	1.143E7	426	48.6	5.31
G3V1B3	60S ribosomal protein L21 OS=Homo sapiens GN=RPL21 PE=1 SV=1	0.00	17.24	3	1	1	1	1.025E7	87	9.9	10.29
Q86VM6	MBNL1 protein OS=Homo sapiens GN=MBNL1 PE=1 SV=1 - [Q86VM6	0.00	6.12	4	1	1	1	1.851E7	343	36.5	9.03
Q9UDT1	Rhomboid domain-containing protein 2 OS=Homo sapiens GN=WUGS	0.00	20.25	2	1	1	1	1.154E7	79	8.4	7.87
J3JS69	40S ribosomal protein S18 OS=Homo sapiens GN=RPS18 PE=1 SV=1	0.00	9.76	2	1	1	1	2.278E6	82	9.8	11.41
B4E0K5	Mitogen-activated protein kinase OS=Homo sapiens GN=MAPK14 PE=	0.00	5.65	26	1	2	2	3.497E6	283	32.3	4.92
G3V5G2	Kinectin (Fragment) OS=Homo sapiens GN=KTN1 PE=1 SV=1 - [G3V	0.00	5.56	3	1	1	1	3.377E7	162	18.7	5.81
F5H1Y0	RAD51-associated protein 1 OS=Homo sapiens GN=RAD51AP1 PE=1	0.00	6.38	3	1	1	1	7.574E6	235	26.4	5.94
A0A0A0MRP0	MAP7 domain-containing protein 3 (Fragment) OS=Homo sapiens GN	0.00	1.50	2	1	1	1	5.666E6	802	90.1	9.29
Q5T532	Golgin-45 (Fragment) OS=Homo sapiens GN=BLZF1 PE=1 SV=1 - [Q	0.00	6.40	2	1	1	1	5.666E6	250	28.0	9.19
A0A087WSY5	Carboxypeptidase B2 OS=Homo sapiens GN=CPB2 PE=1 SV=1 - [A0A	0.00	1.81	2	1	1	1	2.999E6	386	44.0	7.71
A0A087WXS7	ATPase ASNA1 OS=Homo sapiens GN=ASNA1 PE=1 SV=1 - [A0A087	0.00	2.72	2	1	1	1	3.716E6	331	37.1	5.14
B5MCP9	40S ribosomal protein S7 OS=Homo sapiens GN=RPS7 PE=1 SV=1 -	0.00	6.42	2	1	1	1	9.335E6	187	21.3	10.27
E9PKD5	26S proteasome regulatory subunit 6A (Fragment) OS=Homo sapiens	0.00	9.00	6	2	2	2	6.371E6	311	34.6	5.34
X6RM00	ELKS/Rab6-interacting/CAST family member 1 OS=Homo sapiens GN	0.00	2.36	1	1	1	1	1.822E9	976	111.9	6.49
J3KNE2	INO80 complex subunit E OS=Homo sapiens GN=INO80E PE=1 SV=1	0.00	8.29	3	1	1	1	8.733E6	205	21.9	5.20
H7C053	Polyglutamine-binding protein 1 (Fragment) OS=Homo sapiens GN=F	0.00	12.00	2	1	1	1	2.155E7	150	16.6	4.77
H0Y9P0	Receptor of-activated protein C kinase 1 (Fragment) OS=Homo sapie	0.00	19.28	9	1	1	1	1.133E7	83	8.8	4.42
K7EP16	Eukaryotic translation initiation factor 3 subunit G (Fragment) OS=Ho	0.00	25.42	5	2	2	2	1.176E7	118	13.2	9.35
H0Y614	Ubiquitin-fold modifier 1 (Fragment) OS=Homo sapiens GN=UFM1 PE	0.00	18.52	2	1	1	1	4.283E7	81	8.7	9.47
A8MZF9	Developmentally-regulated GTP-binding protein 2 OS=Homo sapiens	0.00	9.62	5	2	2	2	1.470E7	343	38.1	8.91
H3BM30	Enoyl-[acyl-carrier-protein] reductase, mitochondrial (Fragment) OS=	0.00	6.60	2	1	1	1	1.266E7	212	22.9	5.91
A0A087WUD1	Zinc finger protein 787 OS=Homo sapiens GN=ZNF787 PE=1 SV=1 -	0.00	3.40	2	1	1	1	1.369E7	382	40.4	7.96
M0R248	Delta(3,5)-Delta(2,4)-dienoyl-CoA isomerase, mitochondrial (Fragmen	0.00	2.62	2	1	1	1		267	29.2	8.00
C9K0C3	Lysocardiolipin acyltransferase 1 (Fragment) OS=Homo sapiens GN=L	0.00	5.10	2	1	1	1	4.796E6	157	18.4	9.88
C9JQR6	Inactive tyrosine-protein kinase 7 (Fragment) OS=Homo sapiens GN=	0.00	4.28	3	1	1	1	4.047E6	257	28.6	8.25
C9J2I1	Armadillo repeat-containing protein 8 (Fragment) OS=Homo sapiens	0.00	5.40	5	1	1	1	9.914E6	352	39.9	7.85
M0QX52	Microtubule-associated protein RP/EB family member 2 (Fragment) O	0.00	14.96	4	2	2	2	1.309E7	127	14.9	7.87
K7EMW3	Solute carrier family 25 member 39 OS=Homo sapiens GN=SLC25A39	0.00	3.96	4	1	1	1	1.976E6	227	24.9	9.94
C9JV02	Septin-8 (Fragment) OS=Homo sapiens GN=SEPT8 PE=1 SV=1 - [C9	0.00	6.92	19	1	1	1	4.362E6	130	15.0	5.50
H0YLT6	Tight junction protein ZO-1 (Fragment) OS=Homo sapiens GN=TJP1	0.00	6.06	6	1	1	1	5.801E6	231	25.9	9.89
H3BMN1	Arginine/serine-rich protein 1 (Fragment) OS=Homo sapiens GN=RSR	0.00	16.67	4	1	1	1	1.137E7	84	9.4	10.87
F8WF16	Periphilin-1 OS=Homo sapiens GN=PPHLN1 PE=1 SV=1 - [F8WF16_H	0.00	6.27	5	2	2	2	3.877E6	303	34.8	8.02
B8ZZL6	Macrophage-capping protein (Fragment) OS=Homo sapiens GN=CAP	0.00	5.50	3	1	1	1	7.754E6	218	24.1	6.64
K7EJC1	26S proteasome non-ATPase regulatory subunit 8 OS=Homo sapiens	0.00	4.65	4	1	1	1	6.650E6	172	19.8	6.93

G3V249	Probable tRNA N6-adenosine threonylcarbamoyltransferase (Fragmen	0.00	14.41	3	1	1	1	9.060E6	111	11.9	6.92
Q5T712	Monoacylglycerol lipase ABHD12 (Fragment) OS=Homo sapiens GN=	0.00	8.56	2	2	2	2	8.167E6	187	21.2	8.25
C9K088	Motor neuron and pancreas homeobox protein 1 (Fragment) OS=Hor	0.00	7.32	70	1	1	1	1.910E7	82	9.3	10.58
H7C2Y0	Septin-2 (Fragment) OS=Homo sapiens GN=SEPT2 PE=1 SV=1 - [H7	0.00	9.04	3	1	1	1	1.650E7	188	21.6	6.54
E9PKL9	GDP-L-fucose synthase (Fragment) OS=Homo sapiens GN=TSTA3 PE	0.00	8.21	6	2	2	2	4.906E6	268	29.6	6.01
E7ENN3	Nesprin-1 OS=Homo sapiens GN=SYNE1 PE=1 SV=2 - [E7ENN3_HUN	0.00	0.21	3	1	1	1	5.420E6	8392	964.2	5.54
K7EJR8	SWI/SNF-related matrix-associated actin-dependent regulator of chro	0.00	6.52	3	1	1	1	7.885E6	184	20.5	10.05
H0YEU5	Histone-binding protein RBBP4 (Fragment) OS=Homo sapiens GN=RE	0.00	4.79	8	1	1	1	7.936E6	167	19.0	5.53
M0QXY9	snRNA-activating protein complex subunit 2 (Fragment) OS=Homo sa	0.00	2.51	2	1	1	1	7.971E6	279	29.5	9.66
M0R076	Uncharacterized protein (Fragment) OS=Homo sapiens PE=1 SV=1 -	0.00	9.65	4	1	1	1	1.162E7	114	13.1	5.26
Q5JY83	RNA-binding motif protein, X-linked 2 OS=Homo sapiens GN=RBMX2	0.00	13.79	3	1	1	1	6.753E6	58	6.6	4.72
B8A6G2	60S ribosomal protein L10 (Fragment) OS=Homo sapiens GN=RPL10	0.00	5.56	5	1	1	1	3.863E6	108	12.3	8.95
H7C547	Sodium/potassium-transporting ATPase subunit beta-3 (Fragment) O	0.00	32.56	3	1	1	1	1.176E7	43	4.9	9.77
H0YJV7	Transcriptional repressor protein YY1 (Fragment) OS=Homo sapiens	0.00	4.21	4	1	1	1		190	20.8	9.09
E9PNW0	Nucleosome assembly protein 1-like 4 (Fragment) OS=Homo sapiens	0.00	9.35	25	1	1	1	5.208E6	107	12.2	8.16
H0YAW4	Eukaryotic translation initiation factor 3 subunit E (Fragment) OS=Ho	0.00	12.82	4	1	1	1	1.187E7	156	18.1	7.58
E5RHF4	39S ribosomal protein L15, mitochondrial (Fragment) OS=Homo sapi	0.00	6.57	2	1	1	1		198	21.9	10.78
H0YJ95	Y+L amino acid transporter 1 (Fragment) OS=Homo sapiens GN=SL	0.00	4.17	6	1	1	1	2.807E6	168	18.5	5.80
J3QR09	Ribosomal protein L19 OS=Homo sapiens GN=RPL19 PE=1 SV=1 - [J	0.00	4.66	3	1	1	1	8.430E6	193	23.1	11.47
D6RE80	Macrophage erythroblast attacher (Fragment) OS=Homo sapiens GN	0.00	4.92	5	1	1	1	5.233E6	183	20.9	8.18
C9J2I2	Metabotropic glutamate receptor 3 (Fragment) OS=Homo sapiens GN	0.00	8.77	5	1	1	1	5.447E7	171	19.0	9.14
H7C002	Cilia- and flagella-associated protein 69 (Fragment) OS=Homo sapien	0.00	1.17	2	1	1	1	3.248E5	515	58.2	6.35
D6REL8	Fibrinogen beta chain OS=Homo sapiens GN=FGB PE=1 SV=1 - [D6R	0.00	3.31	2	1	1	1		272	31.2	7.25
Q5T4U8	Geranylgeranyl transferase type-2 subunit beta OS=Homo sapiens GN	0.00	7.24	2	1	1	1		152	17.3	5.50
H0YKS8	Lactadherin (Fragment) OS=Homo sapiens GN=MFGE8 PE=1 SV=1 -	0.00	5.60	4	1	1	1		268	29.1	7.90
F8VNT5	Phosphatidylinositol 5-phosphate 4-kinase type-2 gamma (Fragment)	0.00	8.75	3	1	1	1		160	17.9	8.43
H0Y3V8	Sphingosine-1-phosphate lyase 1 (Fragment) OS=Homo sapiens GN=	0.00	11.11	3	1	1	1	9.533E6	135	15.0	7.77
I3L234	Ribosomal L1 domain-containing protein 1 (Fragment) OS=Homo sap	0.00	13.27	5	1	1	1		98	11.6	9.74
H7C1L0	cAMP-dependent protein kinase type II-alpha regulatory subunit (Fra	0.00	4.50	2	1	1	1	7.356E6	200	23.0	6.55
C9JSQ1	Creatine kinase U-type, mitochondrial (Fragment) OS=Homo sapiens	0.00	4.15	3	1	1	1	8.414E6	241	26.7	7.85
E9PNW2	Transmembrane 9 superfamily member (Fragment) OS=Homo sapien	0.00	7.73	10	1	1	1		207	24.1	5.58
F8W6C2	SPATS2-like protein (Fragment) OS=Homo sapiens GN=SPATS2L PE=	0.00	6.42	3	1	1	1	4.547E6	265	29.9	9.04
Q5SZE2	Ceramide synthase 2 (Fragment) OS=Homo sapiens GN=CERS2 PE=	0.00	12.50	7	1	1	2	1.448E7	128	15.3	9.69

693 genes in AGS	745 genes in HCT116	409 genes overlaying
RL6	TRI72	RL6
RLA0	EIF3H	RLA0
ROA2	ROA2	ROA2
F8W6I7	RLA0	F8W6I7
ANXA2	F8W6I7	ANXA2
ROA3	EIF3I	ROA3
K2C1	RL6	K2C1
TRI72	EIF3M	TRI72
LRC59	K2C1	LRC59
G3P	E7EU96	G3P
K1C19	LRC59	K1C19
B2R5W2	IF2A	
K1C10	RL5	K1C10
HNRPC	ROA3	
RL5	CSK22	RL5
GDF15	ANXA2	GDF15
K1C9	G3P	K1C9
DRG1	ODO1	DRG1
EIF3H	PP1A	EIF3H
NPM	ODO2	NPM
AIMP1	GDF15	AIMP1
PSMD7	PCBP2	PSMD7
A0A0C4DGB6	H3BRU6	
BUB3	NPM	BUB3
EIF3I	K1C9	EIF3I
PP1A	PSMD7	PP1A
IF2A	K1C10	IF2A
TRA2B	K22E	TRA2B
SRSF6	ELAV1	SRSF6
K22E	F8VYE8	K22E
D6R9P3	C1QBP	D6R9P3
E7EU96	GAPD1	E7EU96
A0A087WUK2	D6R9P3	A0A087WUK2
FBRL	TRA2B	FBRL
ROA0	DCAF7	ROA0
EIF3M	MAGB2	EIF3M
PP1B	PP1B	PP1B
RFC5	STRAP	RFC5
F8VYE8	FBRL	F8VYE8
RM39	RS3A	RM39
SRSF5	A0A087WUK2	SRSF5
TBA1C	TBA1C	TBA1C
STRAP	PNRC1	STRAP
RM44	G3V2Q1	RM44
TBA4A	EF1A1	
ACTB	BUB3	ACTB
RALY	DRG1	
K7ES61	F8VZJ2	K7ES61
AIMP2	KC1A	AIMP2
HNRPD	K7ES61	
KC1A	RING2	KC1A
EF1A1	K1C16	EF1A1
F5H265	J3KPX7	F5H265

RM38	CDK9	RM38
CSK22	BRCC3	CSK22
A0A0G2JPF8	RM39	A0A0G2JPF8
Q5JRI1	ROA0	Q5JRI1
RM03	TBA1A	RM03
TRA2A	PABP1	TRA2A
G5E9W7	PCBP1	G5E9W7
MARCS	HNRPQ	MARCS
PCBP2	RM44	PCBP2
MO4L2	G5E9W7	MO4L2
PCBP1	E9PEX6	PCBP1
H3BRU6	K2C6B	H3BRU6
RFC3	B3KPJ4	RFC3
C9J9K3	Q5JP53	C9J9K3
MBD3	K2C6C	
K1C14	CAZA1	K1C14
CAZA1	H0YA96	CAZA1
HNRPQ	RM38	HNRPQ
K2C5	B3KTM8	K2C5
PLEK2	RT29	PLEK2
CAZA2	GLYG	CAZA2
DIM1	TBB4B	DIM1
DNJB1	EIF3J	DNJB1
D6REM4	PSDE	D6REM4
SNR40	K2C5	SNR40
E9PB51	RFC4	
IF2G	F5H265	IF2G
C9J9W2	RT09	C9J9W2
E9PKG1	MTDC	E9PKG1
B3KTM8	PSD13	B3KTM8
STAU1	A0A0G2JLR5	
U2AF5	RFC5	U2AF5
D6RBQ9	SRSF5	D6RBQ9
RT29	ACTB	RT29
MK01	RM03	MK01
RFC4	D6REM4	RFC4
GET4	AIMP1	GET4
TMM43	G3BP1	TMM43
HBA	Q5JRI1	HBA
ABC3B	HTRA2	ABC3B
PSDE	C9J9K3	PSDE
K1C16	DIM1	K1C16
LDHB	CAZA2	LDHB
A2MG	SRSF6	A2MG
K2C6A	B1ANR0	
DCAF7	K1C19	DCAF7
A0A0A0MRV0	ICLN	
K2C8	A2A2S5	K2C8
PP2AB	TFB1M	PP2AB
PSD13	YBOX1	PSD13
MTCH1	INO1	MTCH1
CDK9	PDIP2	CDK9
SDCB2	HS71A	
RT09	TPM2	RT09

PURA	WDR5	PURA
PZP	PP2AB	PZP
F8VZJ2	D6RBQ9	F8VZJ2
RFC2	K1C14	RFC2
YBOX1	TRA2A	YBOX1
H2AY	MO4L2	H2AY
TOM40	RT35	TOM40
YBOX3	F8VRH0	YBOX3
E9PK01	GET4	E9PK01
JUN	PURB	JUN
A0A140T933	ABC3B	A0A140T933
RL4	A0A0G2JPF8	RL4
ARP2	JUND	ARP2
ODO2	LORF1	ODO2
HS71A	B4DHE8	HS71A
HNRH3	HSP7C	HNRH3
RS3	TOM40	RS3
Q5JP53	MDHM	Q5JP53
RING2	YBOX3	RING2
ACTH	A6NLN1	
LORF1	OGDHL	LORF1
BUD23	EIF3C	BUD23
IKIP	MCL1	
IDH3B	TIM50	IDH3B
AIFM2	SFXN3	AIFM2
PURB	PURA	PURB
RT31	MK67I	RT31
FUS	RFC2	FUS
NOL7	LDHB	NOL7
A6NG10	RBM4	
DJB11	EIF3L	DJB11
A0A1W2PRV5	MFF	A0A1W2PRV5
GMDS	K2C8	GMDS
MBOA7	AKTS1	
RPP38	EDC4	RPP38
RS3A	RM15	RS3A
GNAI3	DUS1	
HSP7C	GIPC1	HSP7C
TAP26	RT05	
LUC7L	U2AF5	LUC7L
LDHA	A0A140T933	LDHA
BRCC3	PCNA	BRCC3
STX5	E7ER27	
CYR61	NDUA9	CYR61
RRP7A	P5CR2	RRP7A
B3GT6	K7EIJ0	B3GT6
MDHM	E7EMC7	MDHM
T2EB	IDH3B	T2EB
H3BQN4	HNRPK	
MMTA2	BMI1	MMTA2
CC137	RT31	CC137
MCL1	MK01	MCL1
NDUA9	H2AY	NDUA9
GNAI2	RM01	

DUS1	K1C17	DUS1
P5CR2	ALDOA	P5CR2
LEG4	F5H6M0	LEG4
EXOS7	DNJA3	EXOS7
ODPB	DNJB1	ODPB
MTDC	FUS	MTDC
EFTU	SNR40	EFTU
A0A087X1N3	Q5QPL9	
E9PIT3	EBP2	
A0A0A6YYF2	MP2K3	
GTPBA	DJB11	GTPBA
J3KTA4	A0A1W2PRV5	J3KTA4
A0A0G2JLR5	A0A087WWU8	A0A0G2JLR5
MK67I	RS3	MK67I
RT35	J3KPS0	RT35
HS90A	TTP	
SLBP	PPP6	SLBP
BACH	UFD1	BACH
QPCTL	B3GT6	QPCTL
E7ER27	EIF3G	E7ER27
RS2	GO45	RS2
B4DY09	MARCS	B4DY09
M0R3B2	HEM6	
LC7L2	CAVN3	LC7L2
GULP1	EFTU	GULP1
PRS10	ZN346	
TMOD3	QPCTL	
A0A0B4J1Z1	G3XAN4	A0A0B4J1Z1
A0A1W2PPZ5	G3V2D5	A0A1W2PPZ5
UIF	EMD	UIF
H0YLE8	RPP38	
GIPC1	A0A087WX29	GIPC1
F8VZY9	MTCH1	F8VZY9
1B51	RFC3	
F5GYH1	A0A0C4DFV9	
JUND	ERLN1	JUND
ARC1B	SIAH2	ARC1B
SFXN3	BRX1	SFXN3
E7EP32	RPB3	E7EP32
A0A0G2JL54	HPBP1	
SARG	SERC	SARG
E9PMI6	RS2	
A0A087WXC5	IF4A1	A0A087WXC5
FA98C	E9PK01	FA98C
DJB12	PISD	
KPYM	LUC7L	
Q5T6W2	SSRA	
SPB6	GGH	SPB6
G3XAN4	EIF3E	G3XAN4
G3BP1	AIMP2	G3BP1
TBB1	LDHA	
WDR5	TIAR	WDR5
EI2BB	CL043	EI2BB
DHSO	E7EVY0	

A0A1W2PP35	1B58	
LAMB3	GRP78	LAMB3
CAF17	NOSIP	CAF17
ILEU	SEN34	
THOC6	E9PKG1	THOC6
VP37B	E9PCY7	
FOSL1	F8VNY5	FOSL1
E7EVY0	SFXN4	E7EVY0
C9J3L8	J3KTA4	
F5H3X6	T2EB	
ARMX3	EXOS7	ARMX3
RDH14	ARMX3	RDH14
B7Z4J8	TMX4	
RPB3	FABD	RPB3
PDLI4	MMTA2	PDLI4
RL3	LC7L2	RL3
A0A087WZB5	G3V150	
Q5T8U3	MFTC	Q5T8U3
C9JQ42	DNJB6	
AKTS1	PRKRA	AKTS1
E9PCY7	DCD	E9PCY7
E2AK2	RL3	E2AK2
DNJB6	HS90B	DNJB6
HS90B	ABC3F	HS90B
BIEA	SLBP	BIEA
GNAS2	CRKL	
TRUB1	K7EIE8	TRUB1
G3V150	AKAP1	G3V150
CENPV	TMM43	CENPV
G3V438	K7EQ02	
RDH13	WASL	RDH13
TIM50	1C17	TIM50
IF2B	A0A0B4J1Z1	IF2B
SEC13	DHR13	SEC13
TSP1	IF2G	
MRRP1	STML2	
TFB1M	NOL7	TFB1M
EBP2	JUN	EBP2
SERC	CIAO1	SERC
PPP6	H3BV22	PPP6
M0R2P8	A0A087X271	M0R2P8
RPAC1	PZP	
J3KQL8	SPY4	J3KQL8
TEFM	A0A0G2JNZ2	TEFM
STML2	1B15	STML2
CLUS	PCID2	CLUS
CDK6	A2MG	CDK6
MRM1	PRS7	MRM1
A6NLN1	RAE1L	A6NLN1
ELAV1	A2IDA3	ELAV1
KAPCA	WIPF2	KAPCA
F5H1C6	TYSY	
F8VRH0	SARG	F8VRH0
ISY1	CDK6	ISY1

DHRS7	BUD23	
DNJB4	AHSA1	DNJB4
PTOV1	ARP2	
GNA13	A0A0C4DGR2	
A0A087WVC4	ODPB	A0A087WVC4
Q8TCE1	F8VYY9	Q8TCE1
OSER1	CAF17	OSER1
RCL1	DHCR7	RCL1
A0A087WTT1	FOSL1	
E9PKZ0	HNRH3	
TRM7	RUSD3	
IF4A1	CENPV	IF4A1
ABC3F	RL4	ABC3F
E9PLA9	PI42C	E9PLA9
WASL	K7EQL4	WASL
ANKR1	F8VVM2	ANKR1
INO1	ARC1B	INO1
RM01	E9PHS0	RM01
NSDHL	Q5JR04	
TOM34	K7ELV2	
I20L2	J3KT86	I20L2
K7ELV2	GNAI1	K7ELV2
CO3	RPF2	CO3
BRX1	TRABD	BRX1
UFD1	E7EP32	UFD1
RTF2	RO52	
HORN	PDLI4	HORN
ADT3	H7BY36	ADT3
Q5JPU0	GDAP1	
C1QT3	ADT3	
B4DHE8	IGHG1	B4DHE8
B4GT7	ADT2	B4GT7
FMT	NUFP2	FMT
E7EMC7	A0A087WXC5	E7EMC7
VP26A	RDH13	VP26A
CCND1	F5GYN4	CCND1
DHR13	THOC3	DHR13
B1AKM8	ACDSB	
MDC1	ACOD	
DCD	LEG4	DCD
ATAD1	PLCE	ATAD1
F5H6X0	A0A087WTZ5	F5H6X0
H0YE89	XRCC6	H0YE89
ZCCHV	EIF3D	ZCCHV
F8W696	MRM1	
GDAP1	H0YKB1	GDAP1
PLCE	K7EML3	PLCE
ZC3H8	F5H6G4	ZC3H8
B4DUR8	B4DY09	
DJB14	D6RAD4	DJB14
PRKRA	HNRPF	PRKRA
SRSF1	A0A087WVC4	
E5RHW4	OSER1	
NSUN4	C9J384	NSUN4

H14	F5GWT4	
HLAE	HAX1	HLAE
PRPS1	K7EMD6	
HV145	J3KQL8	
VTNC	KLF6	VTNC
ERLN1	EIF3B	ERLN1
K7EM91	RDH14	
A6NP24	ISY1	A6NP24
GLRX3	PNMA1	GLRX3
CSN4	VTNC	
CHM4B	SEC13	
THIL	E2AK2	THIL
PAWR	S10A9	PAWR
U3KQ69	CC137	
MP2K3	B4GT7	MP2K3
AAKG1	B4DQT1	
EXOS8	C9JRD2	EXOS8
K7ES69	TEFM	
Q5JR95	ABRX2	
F8W6G5	J3QL05	
ARC1A	KAPCA	ARC1A
A8MUF7	AUP1	A8MUF7
A0A087X1K6	GTPBA	
ZC21A	EI2BB	ZC21A
J3QT54	FA98C	
NSE3	GRHPR	NSE3
IGHG1	GULP1	IGHG1
A0A0C4DFV9	GMDS	A0A0C4DFV9
PDLI1	MOT4	
H0Y2V1	NONO	H0Y2V1
RS6	Q8TCE1	RS6
RPC6	F5H1F6	
PDIP2	SUCA	PDIP2
H7BY36	ZCCHV	H7BY36
Q5TBH9	H3BPZ1	Q5TBH9
H3BR27	WDR92	
H3BPZ1	CYR61	H3BPZ1
NUCL	BACH	
HDHD5	ARGL1	HDHD5
THOC3	H3BSK9	THOC3
EIF3J	HLAE	EIF3J
BMI1	ATAD1	BMI1
KCAB2	RCL1	KCAB2
A0A0D9SFS3	E7EX73	
E7EW18	TFB2M	
MYH9	H7C3X5	
C9JXB8	NSUN4	
H7BXD8	EIF3F	
C9JEV6	H0YMU3	
K2C74	CLUS	
HEM6	J3QKT4	HEM6
C9JEU5	RRP7A	
TRY1	C9JZR2	TRY1
B3KPJ4	BIEA	B3KPJ4

F8VS81	H0Y2N6	
LRP10	G3V2J8	
NMT1	E9PLA9	NMT1
A0A087WTZ5	C9JP00	A0A087WTZ5
HPBP1	F8VUC8	HPBP1
DKK1	GNB1L	DKK1
DHCR7	SPB6	DHCR7
E9PQW0	HNRPR	E9PQW0
A2IDA3	E5RIU6	A2IDA3
H7C1W2	TXND5	H7C1W2
F5H8H2	Q5JW28	F5H8H2
E7EM64	R4GND3	E7EM64
A0A1W2PQS1	M0R0I0	
OTU6B	LAT1	OTU6B
E7EQR4	PLEK2	
Q5T911	A6NP24	Q5T911
FIBA	FIBP	
B1AN99	PAWR	B1AN99
A0A0D9SFB3	H3BQQ6	A0A0D9SFB3
RAE1L	TRUB2	RAE1L
F5GWG3	C9J050	
PTSS1	Q5T3N1	PTSS1
S4R3D5	RCN1	
C4BPA	MOT1	
B0V3J0	F8VZY9	
A0A087WXQ5	H0YKU1	
SIAS	RL8	
M0R0V2	THRB	
THIC	RS6	THIC
F8WE98	GPAT3	
BCL7C	OGFD3	
SURF2	CBX4	
K7ELP0	VP26A	
SCAM3	H3BM30	SCAM3
F5GZQ3	TAM41	
GNAQ	ABHD4	
GNA11	I20L2	GNA11
EMD	THIL	EMD
G3V5M0	CAVN1	
Q5VU10	RRP15	Q5VU10
DJC25	NSE3	DJC25
B1AHF3	DHB7	
RS4X	X6RLL4	RS4X
MOT1	IER5	MOT1
HEXI1	EXOG	HEXI1
DNJA3	A0A0A0MRR5	DNJA3
H0YJH7	ZFPL1	H0YJH7
COL10	H7C1I0	
ITB4	A0A0B4J207	
H0Y698	THOC6	H0Y698
CB072	K7ER96	
ILF3	ZC21A	
M0QX76	NOLC1	
SRRM1	RAD51	SRRM1

RS25	ZC3HF	
FABD	ZC3H8	FABD
RT05	GNA11	RT05
F2Z2A4	HDHD5	F2Z2A4
CRKL	H0YE89	CRKL
A0A0A0MRQ5	A0A087X1D8	
F8WBC0	M0QX65	
D6RAD4	OTU6B	D6RAD4
ENOA	RED	
MTA2	MSI1H	
RUSD3	RADI	RUSD3
C1QBP	WDR82	C1QBP
GFOD1	EXOS8	
TRABD	CCD92	TRABD
A0A087WVI1	JUNB	
H0Y4R1	HBA	
Q5QP23	STX17	
RRP15	H7BZK6	RRP15
E9PHS0	MYD88	E9PHS0
D6RDK6	V9GYR2	
TPM4	H0YHC3	
K7ERG3	TRY1	
C9JQD0	H3BPG5	
RED	IF2B	RED
B9A008	E9PQW0	B9A008
F5H0V9	F5H6X0	
A0A0U1RQL8	UTP18	
RCC2	CCND1	RCC2
CATE	H3BRL9	CATE
H3BV22	H0YIZ6	H3BV22
H0Y6Y4	A0A0D9SFB3	
ARGL1	H7C0S9	ARGL1
K7EK18	PTSS1	
F5GX09	H0Y9P0	
TFB2M	H0YJ92	TFB2M
RL13	M0R1Z5	
TYSY	C9J9W2	TYSY
F5GYN4	H1X	F5GYN4
PINX1	TF2B	
TRUB2	B4DNK4	TRUB2
Q5TDE7	F8W733	Q5TDE7
PFKAL	SHLB1	
G3V4X8	NUP37	G3V4X8
E9PQX8	F8WJN3	
PGK1	B1AN99	
A0A087X1J2	Z3H7B	
J3QL05	DNJA1	J3QL05
F8VVM2	RS4X	F8VVM2
V9GYR2	H0Y698	V9GYR2
RPF2	G3V4X1	RPF2
K7EML3	LAMB3	K7EML3
F2Z2Y4	HEXI1	F2Z2Y4
GALK1	RS23	
E9PQA5	DUS4	

DFFB	M0R2P8	
J3QL14	I3L2R3	
GPT11	H1T	
M0QYK9	F2Z2Y4	M0QYK9
WIPF2	M0QYK9	WIPF2
H3BLU7	PEX3	H3BLU7
C9JE01	A0A087WWT3	C9JE01
C9J1G2	F2Z3J9	
H3BQQ6	CDX2	H3BQQ6
G3V1C1	Q5JPU2	G3V1C1
M0QX71	H3BSQ0	
I3L1Q5	H3BNC1	I3L1Q5
MOGS	H7C1W2	
A0A0A0MRR5	F5H8H2	A0A0A0MRR5
TFPT	A0A0C4DGC5	
A0A0M3HER1	A0A1W2PQ16	A0A0M3HER1
S4R3W8	FMT	S4R3W8
ATPG	Q5TBH0	
E9PPT8	J3KSR8	E9PPT8
TSR3	ESIP1	TSR3
F5H6G4	GLRX3	F5H6G4
AK1BF	ARC1A	
G3V4X1	E9PH82	G3V4X1
H0YI20	Q5T8U3	
H0YH81	SRSF8	
H1X	B9A035	H1X
H7BZN1	SRRM1	
MACOI	C9J2I1	
Q5TBH0	OSGI1	Q5TBH0
G5EA42	E9PHA9	
CLC11	A0A1B0GW95	
H0Y711	G3V4X8	
H0YJN9	SCAM3	H0YJN9
Q5QPP9	SGPP1	
Q5VZC3	K7EJR8	
CHIP	K7EII7	CHIP
F5H282	D6RF48	F5H282
WDR92	B9A008	WDR92
A0A0G2JQ92	B1AP15	
Q5T9P8	TOB2	
K7EQ02	DDX6	K7EQ02
H0Y5R6	PBDC1	
C9J840	S4R3W8	
F8W914	Q5T911	
PPIP2	H0Y2V1	
PAIRB	GMPPB	
CTGF	H7BY16	CTGF
H7BZK6	Q5TDE7	H7BZK6
OGFD3	TRUB1	OGFD3
D6R967	KCAB2	
A0A0A0MTD5	PIR	
ZN444	A6NHN2	
PON1	K7ELB8	
TR150	THIC	

TNR21	TRADD	
A0A087WY55	D6R918	
Q5T0A6	E9PJD9	
RS9	NMT1	
R4GND3	MKRN1	R4GND3
F2Z3J9	I3L387	F2Z3J9
F5H3K2	ANKR1	
LGAT1	E5RIL5	LGAT1
D6RIU4	LGAT1	D6RIU4
F5H2Z7	PPR35	
PNRC1	A0A0D9SEU5	PNRC1
H0Y6D8	E9PIZ4	H0Y6D8
S4R3V8	EF2	
E9PJW1	H0YJH7	
DHX15	A0A0M3HER1	
DHX9	H0YJN9	DHX9
F5H6T0	F8WE04	
C9J168	G3PT	
Q5TBG5	RAB10	
PCID2	Q9UDT1	PCID2
H0YMJ6	BI2L1	
Q5VV87	G3V1C1	
E5RHH1	E7EX54	
RBM14	DJB14	RBM14
Q5JRC6	CATE	
C9J0D1	F8WFC6	
K7ER96	A0A0A0MSI0	K7ER96
C9IZL7	ATD3C	
AMERL	RMD5A	AMERL
H3BPG5	FILA2	H3BPG5
TXND5	I3L1Q5	TXND5
TF3C6	A2A2L6	
S4R3M7	ZCHC3	
B7Z8R2	F8I2	B7Z8R2
SYDC	PAK2	
B4DSN5	C9JUG1	
C9J8E1	SAM50	
UBCP1	CTU1	UBCP1
RPR1B	H7BY94	
H2B1K	HSDL1	
ACOD	RANB9	ACOD
ALG5	PSPC1	
ARRD1	K7EP82	
AUP1	S10A8	AUP1
C27C1	IGBP1	
CDCA5	E9PPT8	
CHERP	CF132	
CIAO1	UIF	CIAO1
COPA	H0YJE9	
CSN5	C9IYF5	
DNJA1	A0A1W2PPZ5	DNJA1
EF1G	M0R248	EF1G
EIF3C	STK39	EIF3C
EIF3F	H3BQC6	EIF3F

EPHA2	KAP2	
G3BP2	CTGF	G3BP2
GMPPB	AIFM2	GMPPB
GNB1L	RIR2B	GNB1L
GOLP3	Q5T457	
H15	H7BXR2	
KCNT2	K7EPP7	
LYAR	A0A1W2PRW5	
MK13	B5MCP9	
MYD88	AMERL	MYD88
OAS3	MSX2	
P66A	RBM14	
PBIP1	H7C547	
PCNA	F2Z2A4	PCNA
PRR11	H7C5D5	
RL34	J3QKY4	
RM37	F5GY15	RM37
RRP1B	Q6P0N6	
RU17	H0Y894	
SAHH	B4DLR8	
SAM50	B7Z8R2	SAM50
SFXN4	Q5T712	SFXN4
SGTA	F5H282	
SPAS2	RM37	SPAS2
SREC2	UBCP1	
SRPK1	F110C	
SRPX2	DKK1	
STX4	E5RJG9	
TLN1	E9PNW8	
UBP13	E9PEK1	
VWA3B	M4A10	
XRP2	H0YLA4	
ZC3HF	DHX9	ZC3HF
G3V1B3	KLF2	
Q86VM6	E9PN19	
Q9UDT1	C9JBY0	Q9UDT1
J3JS69	ANX11	
B4E0K5	E7EU85	
G3V5G2	TSR3	
F5H1Y0	D6RHI7	
A0A0A0MRP0	K7ERU8	
Q5T532	LYRIC	
A0A087WSY5	A2RQR6	
A0A087WXS7	RDH10	
B5MCP9	K7EJC1	B5MCP9
E9PKD5	ADHX	
X6RM00	D6RIU4	
J3KNE2	H3BLU7	
H7C053	A0A0A0MRK6	
H0Y9P0	M0QZR0	H0Y9P0
K7EP16	CC50B	
H0Y614	NSUN3	
A8MZF9	H0YBM4	
H3BM30	DNJB4	H3BM30

A0A087WUD1	CA106	
M0R248	PI42B	M0R248
C9K0C3	RCC2	
C9JQR6	TCPW	
C9J2I1	E7EM64	C9J2I1
M0QX52	HORN	M0QX52
K7EMW3	C9JCN8	
C9JV02	RUSD4	
H0YLT6	Q5LJB0	
H3BMN1	A8MUF7	
F8WF16	LARP1	
B8ZZL6	A0A075B6F6	
K7EJC1	A0A0C4DGA2	K7EJC1
G3V249	B1AK81	
Q5T712	MEP50	Q5T712
C9K088	E5RGE1	
H7C2Y0	ARG39	
E9PKL9	ASAP2	
E7ENN3	ASPH1	
K7EJR8	BACHL	K7EJR8
H0YEU5	CHIP	H0YEU5
M0QXY9	CO3	M0QXY9
M0R076	DJC25	
Q5JY83	DUS11	
B8A6G2	EF1G	
H7C547	FA5	H7C547
H0YJV7	FIP1	
E9PNW0	FUBP3	
H0YAW4	G3BP2	
E5RHF4	GP119	
H0YJ95	KCMF1	
J3QR09	KLD10	
D6RE80	LRIQ3	
C9J2I2	MDN1	
H7C002	MTG2	
D6REL8	PAR6B	
Q5T4U8	PPID	
H0YKS8	RBM26	
F8VNT5	SPAS2	
H0Y3V8	GARS	
I3L234	TSK	
H7C1L0	TXLNA	
C9JSQ1	WBP11	
E9PNW2	XRCC5	
F8W6C2	ZN771	
Q5SZE2	E7ETY4	
	G5E9U9	
	J3KRL8	
	V9GYL1	
	Q5SQT6	
	A0A024R7W5	
	M0R1N9	
	Q5TBH9	
	A0A0A0MRE9	

K7EM90
I3L4G9
A0A087WU62
Q5SY74
A0A087WYC0
K7EJY5
H0YJR8
C9JE01
C9JDR0
H0Y9D9
E7EVK2
H0Y9T5
M0QX52
C9J7M8
H0Y641
E9PPH5
F5H2X8
C9J0E9
F2Z3K5
E5RGJ2
H0Y6D8
E9PLG2
Q5VU10
H0Y9K7
A0A087WUJ2
H3BPL5
H0YEU5
M0QXY9
E9PNF3
Q5W011
E9PPQ0
H0YHZ5
Q5SZU1
H7BYY4
H7C5R5
E9PNU1
A0A0A0MS89
F8VR84
A0A1B0GW11
E9PK89
E9PLA6
G3V5L5
K7EJP1
Q5JSB5

KOBAS analysis results

##Databases: KEGG PATHWAY, KEGG DISEASE, Gene Ontology
##Statistical test method: hypergeometric test / Fisher's exact test
##FDR correction method: Benjamini and Hochberg

#Term	Database	ID	Input num	Backgroun	P-Value	Corrected	Input	Hyperlink
RNA transport	KEGG PATHWAY	hsa03013	10	165	1.48E-09	1.42E-07	EIF3H|EIF3I|RPP38|SRRM1|SEC13|EIF3C|	http://www.genome.jp/kegg-bin/show_pathway?hsa03013/hsa:8667%09red/hsa:8665%09red/hsa:8663%09red/hsa:6396%09red/hsa:10250%09red/hsa:79228%09red/hsa:8668%09red/hsa:8669%09red/hsa:10557%09red/hsa:84321%09red
Mismatch repair	KEGG PATHWAY	hsa03430	5	23	6.63E-08	3.37E-06	RFC5|RFC4|PCNA|RFC3|RFC2	http://www.genome.jp/kegg-bin/show_pathway?hsa03430/hsa:5111%09red/hsa:5982%09red/hsa:5983%09red/hsa:5984%09red/hsa:5985%09red
Spliceosome	KEGG PATHWAY	hsa03040	8	135	8.01E-08	3.83E-06	SRSF5|SRSF6|DIM1|ISY1|PCBP1|THOC3|	http://www.genome.jp/kegg-bin/show_pathway?hsa03040/hsa:10907%09red/hsa:6434%09red/hsa:6431%09red/hsa:6430%09red/hsa:29896%09red/hsa:57461%09red/hsa:5093%09red/hsa:84321%09red
DNA replication	KEGG PATHWAY	hsa03030	5	36	4.86E-07	1.84E-05	RFC5|RFC4|PCNA|RFC3|RFC2	http://www.genome.jp/kegg-bin/show_pathway?hsa03030/hsa:5111%09red/hsa:5982%09red/hsa:5983%09red/hsa:5984%09red/hsa:5985%09red
Nucleotide excision repair	KEGG PATHWAY	hsa03420	5	47	1.63E-06	5.53E-05	RFC5|RFC4|PCNA|RFC3|RFC2	http://www.genome.jp/kegg-bin/show_pathway?hsa03420/hsa:5111%09red/hsa:5982%09red/hsa:5983%09red/hsa:5984%09red/hsa:5985%09red
Yersinia infection	KEGG PATHWAY	hsa05135	6	121	9.39E-06	0.000283	CRKL|JUN|WASL|WIPF2|ACTB|MYD88	http://www.genome.jp/kegg-bin/show_pathway?hsa05135/hsa:3725%09red/hsa:1399%09red/hsa:147179%09red/hsa:8976%09red/hsa:4615%09red/hsa:60%09red
Focal adhesion	KEGG PATHWAY	hsa04510	7	199	1.45E-05	0.000406	CRKL|CCND1|JUN|PP1B|PP1A|ACTB|LAI	http://www.genome.jp/kegg-bin/show_pathway?hsa04510/hsa:1399%09red/hsa:595%09red/hsa:3725%09red/hsa:5500%09red/hsa:3914%09red/hsa:5499%09red/hsa:60%09red
Measles	KEGG PATHWAY	hsa05162	5	138	0.000221	0.004612	EIF3H|CCND1|MYD88|JUN|CDK6	http://www.genome.jp/kegg-bin/show_pathway?hsa05162/hsa:8667%09red/hsa:595%09red/hsa:3725%09red/hsa:4615%09red/hsa:1021%09red
Salmonella infection	KEGG PATHWAY	hsa05132	4	83	0.000343	0.00632	ACTB|MYD88|JUN|WASL	http://www.genome.jp/kegg-bin/show_pathway?hsa05132/hsa:3725%09red/hsa:8976%09red/hsa:4615%09red/hsa:60%09red
Oxytocin signaling pathway	KEGG PATHWAY	hsa04921	5	153	0.00035	0.006333	CCND1|ACTB|JUN|PP1B|PP1A	http://www.genome.jp/kegg-bin/show_pathway?hsa04921/hsa:3725%09red/hsa:595%09red/hsa:5499%09red/hsa:5500%09red/hsa:60%09red
Hippo signaling pathway	KEGG PATHWAY	hsa04390	5	154	0.00036	0.006447	CCND1|ACTB|CTGF|PP1B|PP1A	http://www.genome.jp/kegg-bin/show_pathway?hsa04390/hsa:595%09red/hsa:5499%09red/hsa:5500%09red/hsa:1490%09red/hsa:60%09red
Propanoate metabolism	KEGG PATHWAY	hsa00640	3	34	0.000375	0.006635	LDHA|LDHB|THIL	http://www.genome.jp/kegg-bin/show_pathway?hsa00640/hsa:38%09red/hsa:3939%09red/hsa:3945%09red

IL-17 signaling pathway	KEGG PATHWAY	hsa04657	4	93	0.000518	0.008274	FOSL1|JUN|JUND|ELAV1	http://www.genome.jp/kegg-bin/show_pathway?hsa04657/hsa:8061%09red/hsa:1994%09red/hsa:3725%09red/hsa:3727%09red
Pyruvate metabolism	KEGG PATHWAY	hsa00620	3	39	0.000546	0.008636	LDHA|LDHB|THIL	http://www.genome.jp/kegg-bin/show_pathway?hsa00620/hsa:38%09red/hsa:3939%09red/hsa:3945%09red
Tight junction	KEGG PATHWAY	hsa04530	5	170	0.000558	0.008733	PCNA|CCND1|ACTB|JUN|WASL	http://www.genome.jp/kegg-bin/show_pathway?hsa04530/hsa:5111%09red/hsa:3725%09red/hsa:8976%09red/hsa:595%09red/hsa:60%09red
Epstein-Barr virus infection	KEGG PATHWAY	hsa05169	5	201	0.001159	0.015089	CCND1|PSMD7|MYD88|JUN|CDK6	http://www.genome.jp/kegg-bin/show_pathway?hsa05169/hsa:595%09red/hsa:5713%09red/hsa:3725%09red/hsa:4615%09red/hsa:1021%09red
Proteoglycans in cancer	KEGG PATHWAY	hsa05205	5	203	0.001209	0.015599	CCND1|ACTB|PLCE|PP1B|PP1A	http://www.genome.jp/kegg-bin/show_pathway?hsa05205/hsa:51196%09red/hsa:595%09red/hsa:5499%09red/hsa:5500%09red/hsa:60%09red
**Cell cycle**	**KEGG PATHWAY**	**hsa04110**	**4**	**124**	**0.00146**	**0.01733**	**PCNA|CCND1|BUB3|CDK6**	**http://www.genome.jp/kegg-bin/show_pathway?hsa04110/hsa:9184%09red/hsa:5111%09red/hsa:595%09red/hsa:1021%09red**
Regulation of actin cytoskeleton	KEGG PATHWAY	hsa04810	5	214	0.001517	0.017773	CRKL|ACTB|PP1B|PP1A|WASL	http://www.genome.jp/kegg-bin/show_pathway?hsa04810/hsa:1399%09red/hsa:8976%09red/hsa:5499%09red/hsa:5500%09red/hsa:60%09red
Fatty acid metabolism	KEGG PATHWAY	hsa01212	3	57	0.00155	0.01802	FABD|RING2|THIL	http://www.genome.jp/kegg-bin/show_pathway?hsa01212/hsa:38%09red/hsa:27349%09red/hsa:7923%09red
Shigellosis	KEGG PATHWAY	hsa05131	3	68	0.002511	0.02555	ACTB|WASL|CRKL	http://www.genome.jp/kegg-bin/show_pathway?hsa05131/hsa:1399%09red/hsa:8976%09red/hsa:60%09red
Amphetamine addiction	KEGG PATHWAY	hsa05031	3	68	0.002511	0.02555	JUN|PP1B|PP1A	http://www.genome.jp/kegg-bin/show_pathway?hsa05031/hsa:3725%09red/hsa:5499%09red/hsa:5500%09red
Fatty acid biosynthesis	KEGG PATHWAY	hsa00061	2	18	0.002582	0.025631	FABD|RING2	http://www.genome.jp/kegg-bin/show_pathway?hsa00061/hsa:27349%09red/hsa:7923%09red
p53 signaling pathway	KEGG PATHWAY	hsa04115	3	72	0.002934	0.027772	AIFM2|CCND1|CDK6	http://www.genome.jp/kegg-bin/show_pathway?hsa04115/hsa:84883%09red/hsa:595%09red/hsa:1021%09red
Bacterial invasion of epithelial cells	KEGG PATHWAY	hsa05100	3	74	0.003161	0.028912	ACTB|WASL|CRKL	http://www.genome.jp/kegg-bin/show_pathway?hsa05100/hsa:1399%09red/hsa:8976%09red/hsa:60%09red
Cushing syndrome	KEGG PATHWAY	hsa04934	4	155	0.003202	0.028964	CCND1|WDR5|CDK6|GNA11	http://www.genome.jp/kegg-bin/show_pathway?hsa04934/hsa:2767%09red/hsa:595%09red/hsa:1021%09red/hsa:11091%09red
Chronic myeloid leukemia	KEGG PATHWAY	hsa05220	3	76	0.003399	0.030291	CCND1|CRKL|CDK6	http://www.genome.jp/kegg-bin/show_pathway?hsa05220/hsa:1399%09red/hsa:595%09red/hsa:1021%09red
Cellular senescence	KEGG PATHWAY	hsa04218	4	160	0.003577	0.030972	CCND1|CDK6|PP1B|PP1A	http://www.genome.jp/kegg-bin/show_pathway?hsa04218/hsa:595%09red/hsa:5499%09red/hsa:5500%09red/hsa:1021%09red
Wnt signaling pathway	KEGG PATHWAY	hsa04310	4	160	0.003577	0.030972	CCND1|FOSL1|JUN|DKK1	http://www.genome.jp/kegg-bin/show_pathway?hsa04310/hsa:8061%09red/hsa:595%09red/hsa:3725%09red/hsa:22943%09red

Influenza A	KEGG PATHWAY	hsa05164	4	167	0.004149	0.032788	ACTB|MYD88|TRY1|CDK6	http://www.genome.jp/kegg-bin/show_pathway?hsa05164/hsa:4615%09red/hsa:1021%09red/hsa:60%09red/hsa:5644%09red
mRNA surveillance pathway	KEGG PATHWAY	hsa03015	3	91	0.005529	0.041477	SRRM1|PP1B|PP1A	http://www.genome.jp/kegg-bin/show_pathway?hsa03015/hsa:5499%09red/hsa:5500%09red/hsa:10250%09red
Small cell lung cancer	KEGG PATHWAY	hsa05222	3	93	0.005861	0.042406	CCND1|LAMB3|CDK6	http://www.genome.jp/kegg-bin/show_pathway?hsa05222/hsa:595%09red/hsa:3914%09red/hsa:1021%09red
MicroRNAs in cancer	KEGG PATHWAY	hsa05206	5	299	0.006143	0.043338	CCND1|CRKL|BMI1|CDK6|MCL1	http://www.genome.jp/kegg-bin/show_pathway?hsa05206/hsa:1399%09red/hsa:595%09red/hsa:4170%09red/hsa:1021%09red/hsa:100532731%09red
AGE-RAGE signaling pathway in diabetic complications	KEGG PATHWAY	hsa04933	3	100	0.007117	0.049092	CCND1|PLCE|JUN	http://www.genome.jp/kegg-bin/show_pathway?hsa04933/hsa:51196%09red/hsa:595%09red/hsa:3725%09red
Chagas disease (American trypanosomiasis)	KEGG PATHWAY	hsa05142	3	103	0.0077	0.052198	MYD88|JUN|GNA11	http://www.genome.jp/kegg-bin/show_pathway?hsa05142/hsa:2767%09red/hsa:3725%09red/hsa:4615%09red
Fructose and mannose metabolism	KEGG PATHWAY	hsa00051	2	33	0.007791	0.052198	GMDS|GMPPB	http://www.genome.jp/kegg-bin/show_pathway?hsa00051/hsa:29925%09red/hsa:2762%09red
Ribosome biogenesis in eukaryotes	KEGG PATHWAY	hsa03008	3	105	0.008104	0.053413	RPP38|RCL1|RRP7A	http://www.genome.jp/kegg-bin/show_pathway?hsa03008/hsa:10557%09red/hsa:27341%09red/hsa:10171%09red
Metabolic pathways	KEGG PATHWAY	hsa01100	12	1433	0.009004	0.057713	IDH3B|PLCE|INO1|RING2|GMPPB|FABD	http://www.genome.jp/kegg-bin/show_pathway?hsa01100/hsa:29920%09red/hsa:29925%09red/hsa:3945%09red/hsa:7923%09red/hsa:3420%09red/hsa:2762%09red/hsa:1717%09red/hsa:38%09red/hsa:3939%09red/hsa:51196%09red/hsa:27349%09red/hsa:51477%09red
Human immunodeficiency virus 1 infection	KEGG PATHWAY	hsa05170	4	212	0.009344	0.059657	MYD88|JUN|CRKL|GNA11	http://www.genome.jp/kegg-bin/show_pathway?hsa05170/hsa:1399%09red/hsa:2767%09red/hsa:3725%09red/hsa:4615%09red
cAMP signaling pathway	KEGG PATHWAY	hsa04024	4	214	0.009642	0.060842	PLCE|JUN|PP1B|PP1A	http://www.genome.jp/kegg-bin/show_pathway?hsa04024/hsa:3725%09red/hsa:51196%09red/hsa:5499%09red/hsa:5500%09red
Human T-cell leukemia virus 1 infection	KEGG PATHWAY	hsa05166	4	219	0.010413	0.06403	CCND1|FOSL1|JUN|BUB3	http://www.genome.jp/kegg-bin/show_pathway?hsa05166/hsa:9184%09red/hsa:8061%09red/hsa:595%09red/hsa:3725%09red
Ferroptosis	KEGG PATHWAY	hsa04216	2	40	0.011082	0.066326	PCBP2|PCBP1	http://www.genome.jp/kegg-bin/show_pathway?hsa04216/hsa:5094%09red/hsa:5093%09red
Thyroid hormone signaling pathway	KEGG PATHWAY	hsa04919	3	119	0.01128	0.067265	CCND1|ACTB|PLCE	http://www.genome.jp/kegg-bin/show_pathway?hsa04919/hsa:51196%09red/hsa:595%09red/hsa:60%09red
Human cytomegalovirus infection	KEGG PATHWAY	hsa05163	4	225	0.01139	0.067675	CCND1|CRKL|CDK6|GNA11	http://www.genome.jp/kegg-bin/show_pathway?hsa05163/hsa:1399%09red/hsa:2767%09red/hsa:595%09red/hsa:1021%09red
Platelet activation	KEGG PATHWAY	hsa04611	3	124	0.012564	0.071997	ACTB|PP1B|PP1A	http://www.genome.jp/kegg-bin/show_pathway?hsa04611/hsa:5499%09red/hsa:5500%09red/hsa:60%09red
Osteoclast differentiation	KEGG PATHWAY	hsa04380	3	128	0.01365	0.076101	FOSL1|JUN|JUND	http://www.genome.jp/kegg-bin/show_pathway?hsa04380/hsa:8061%09red/hsa:3725%09red/hsa:3727%09red

Vascular smooth muscle contraction	KEGG PATHWAY	hsa04270	3	132	0.014787	0.080735	PP1B|PP1A|GNA11	http://www.genome.jp/kegg-bin/show_pathway?hsa04270/hsa:2767%09red/hsa:5499%09red/hsa:5500%09red
Amino sugar and nucleotide sugar metabolism	KEGG PATHWAY	hsa00520	2	48	0.015459	0.082789	GMDS|GMPPB	http://www.genome.jp/kegg-bin/show_pathway?hsa00520/hsa:29925%09red/hsa:2762%09red
Apoptosis	KEGG PATHWAY	hsa04210	3	136	0.015977	0.085117	ACTB|JUN|MCL1	http://www.genome.jp/kegg-bin/show_pathway?hsa04210/hsa:3725%09red/hsa:4170%09red/hsa:60%09red
Cysteine and methionine metabolism	KEGG PATHWAY	hsa00270	2	49	0.016051	0.085117	LDHA|LDHB	http://www.genome.jp/kegg-bin/show_pathway?hsa00270/hsa:3939%09red/hsa:3945%09red
Insulin signaling pathway	KEGG PATHWAY	hsa04910	3	137	0.016282	0.085785	CRKL|PP1B|PP1A	http://www.genome.jp/kegg-bin/show_pathway?hsa04910/hsa:1399%09red/hsa:5499%09red/hsa:5500%09red
Pathways in cancer	KEGG PATHWAY	hsa05200	6	530	0.016607	0.087215	CRKL|CCND1|JUN|CDK6|GNA11|LAMB3	http://www.genome.jp/kegg-bin/show_pathway?hsa05200/hsa:2767%09red/hsa:1021%09red/hsa:1399%09red/hsa:595%09red/hsa:3725%09red/hsa:3914%09red
Breast cancer	KEGG PATHWAY	hsa05224	3	147	0.019518	0.095994	CCND1|JUN|CDK6	http://www.genome.jp/kegg-bin/show_pathway?hsa05224/hsa:595%09red/hsa:3725%09red/hsa:1021%09red
Pathogenic Escherichia coli infection	KEGG PATHWAY	hsa05130	2	55	0.019798	0.095994	ACTB|WASL	http://www.genome.jp/kegg-bin/show_pathway?hsa05130/hsa:8976%09red/hsa:60%09red
Legionellosis	KEGG PATHWAY	hsa05134	2	55	0.019798	0.095994	MYD88|EF1G	http://www.genome.jp/kegg-bin/show_pathway?hsa05134/hsa:1937%09red/hsa:4615%09red
Viral myocarditis	KEGG PATHWAY	hsa05416	2	60	0.023172	0.095994	CCND1|ACTB	http://www.genome.jp/kegg-bin/show_pathway?hsa05416/hsa:595%09red/hsa:60%09red
Hepatitis B	KEGG PATHWAY	hsa05161	3	163	0.025382	0.095994	PCNA|MYD88|JUN	http://www.genome.jp/kegg-bin/show_pathway?hsa05161/hsa:5111%09red/hsa:3725%09red/hsa:4615%09red
Protein processing in endoplasmic reticulum	KEGG PATHWAY	hsa04141	3	165	0.026174	0.095994	UFD1|CHIP|SEC13	http://www.genome.jp/kegg-bin/show_pathway?hsa04141/hsa:10273%09red/hsa:7353%09red/hsa:6396%09red
cGMP-PKG signaling pathway	KEGG PATHWAY	hsa04022	3	167	0.02698	0.097628	PP1B|PP1A|GNA11	http://www.genome.jp/kegg-bin/show_pathway?hsa04022/hsa:2767%09red/hsa:5499%09red/hsa:5500%09red
MAPK signaling pathway	KEGG PATHWAY	hsa04010	4	295	0.027262	0.097628	MYD88|JUN|JUND|CRKL	http://www.genome.jp/kegg-bin/show_pathway?hsa04010/hsa:1399%09red/hsa:3727%09red/hsa:3725%09red/hsa:4615%09red
Hepatocellular carcinoma	KEGG PATHWAY	hsa05225	3	168	0.027387	0.097628	CCND1|ACTB|CDK6	http://www.genome.jp/kegg-bin/show_pathway?hsa05225/hsa:595%09red/hsa:1021%09red/hsa:60%09red
Non-small cell lung cancer	KEGG PATHWAY	hsa05223	2	66	0.027508	0.097628	CCND1|CDK6	http://www.genome.jp/kegg-bin/show_pathway?hsa05223/hsa:595%09red/hsa:1021%09red
Long-term potentiation	KEGG PATHWAY	hsa04720	2	67	0.02826	0.097628	PP1B|PP1A	http://www.genome.jp/kegg-bin/show_pathway?hsa04720/hsa:5499%09red/hsa:5500%09red
Glycolysis / Gluconeogenesis	KEGG PATHWAY	hsa00010	2	68	0.029021	0.097628	LDHA|LDHB	http://www.genome.jp/kegg-bin/show_pathway?hsa00010/hsa:3939%09red/hsa:3945%09red
Central carbon metabolism in cancer	KEGG PATHWAY	hsa05230	2	69	0.029789	0.097628	LDHA|LDHB	http://www.genome.jp/kegg-bin/show_pathway?hsa05230/hsa:3939%09red/hsa:3945%09red
Renal cell carcinoma	KEGG PATHWAY	hsa05211	2	69	0.029789	0.097628	JUN|CRKL	http://www.genome.jp/kegg-bin/show_pathway?hsa05211/hsa:1399%09red/hsa:3725%09red
Adherens junction	KEGG PATHWAY	hsa04520	2	72	0.032142	0.099902	ACTB|WASL	http://www.genome.jp/kegg-bin/show_pathway?hsa04520/hsa:8976%09red/hsa:60%09red

Melanoma	KEGG PATHWAY	hsa05218	2	72	0.032142	0.099902	CCND1|CDK6	http://www.genome.jp/kegg-bin/show_pathway?hsa05218/hsa:595%09red/hsa:1021%09red
Leishmaniasis	KEGG PATHWAY	hsa05140	2	74	0.033751	0.099902	MYD88|JUN	http://www.genome.jp/kegg-bin/show_pathway?hsa05140/hsa:3725%09red/hsa:4615%09red
Inositol phosphate metabolism	KEGG PATHWAY	hsa00562	2	74	0.033751	0.099902	PLCE|INO1	http://www.genome.jp/kegg-bin/show_pathway?hsa00562/hsa:51196%09red/hsa:51477%09red
Pancreatic cancer	KEGG PATHWAY	hsa05212	2	75	0.034566	0.101577	CCND1|CDK6	http://www.genome.jp/kegg-bin/show_pathway?hsa05212/hsa:595%09red/hsa:1021%09red
Glioma	KEGG PATHWAY	hsa05214	2	75	0.034566	0.101577	CCND1|CDK6	http://www.genome.jp/kegg-bin/show_pathway?hsa05214/hsa:595%09red/hsa:1021%09red
Biosynthesis of amino acids	KEGG PATHWAY	hsa01230	2	75	0.034566	0.101577	IDH3B|P5CR2	http://www.genome.jp/kegg-bin/show_pathway?hsa01230/hsa:3420%09red/hsa:29920%09red
Kaposi sarcoma-associated herpesvirus infection	KEGG PATHWAY	hsa05167	3	186	0.035286	0.102621	CCND1|JUN|CDK6	http://www.genome.jp/kegg-bin/show_pathway?hsa05167/hsa:595%09red/hsa:3725%09red/hsa:1021%09red
Transcriptional misregulation in cancer	KEGG PATHWAY	hsa05202	3	186	0.035286	0.102621	CDK9|FUS|BMI1	http://www.genome.jp/kegg-bin/show_pathway?hsa05202/hsa:2521%09red/hsa:1025%09red/hsa:100532731%09red
Pertussis	KEGG PATHWAY	hsa05133	2	76	0.03539	0.102621	MYD88|JUN	http://www.genome.jp/kegg-bin/show_pathway?hsa05133/hsa:3725%09red/hsa:4615%09red
Arrhythmogenic right ventricular cardiomyopathy (ARVC)	KEGG PATHWAY	hsa05412	2	77	0.036221	0.102621	EMD|ACTB	http://www.genome.jp/kegg-bin/show_pathway?hsa05412/hsa:60%09red/hsa:2010%09red
Synthesis and degradation of ketone bodies	KEGG PATHWAY	hsa00072	1	10	0.040833	0.106193	THIL	http://www.genome.jp/kegg-bin/show_pathway?hsa00072/hsa:38%09red
Herpes simplex virus 1 infection	KEGG PATHWAY	hsa05168	5	492	0.04108	0.106494	SRSF5|SRSF6|MYD88|PP1B|PP1A	http://www.genome.jp/kegg-bin/show_pathway?hsa05168/hsa:6431%09red/hsa:5499%09red/hsa:5500%09red/hsa:6430%09red/hsa:4615%09red
Viral carcinogenesis	KEGG PATHWAY	hsa05203	3	201	0.042669	0.109013	CCND1|JUN|CDK6	http://www.genome.jp/kegg-bin/show_pathway?hsa05203/hsa:595%09red/hsa:3725%09red/hsa:1021%09red
ErbB signaling pathway	KEGG PATHWAY	hsa04012	2	85	0.043133	0.109013	JUN|CRKL	http://www.genome.jp/kegg-bin/show_pathway?hsa04012/hsa:1399%09red/hsa:3725%09red
Colorectal cancer	KEGG PATHWAY	hsa05210	2	86	0.044029	0.109013	CCND1|JUN	http://www.genome.jp/kegg-bin/show_pathway?hsa05210/hsa:595%09red/hsa:3725%09red
PD-L1 expression and PD-1 checkpoint pathway in cancer	KEGG PATHWAY	hsa05235	2	89	0.046758	0.11118	MYD88|JUN	http://www.genome.jp/kegg-bin/show_pathway?hsa05235/hsa:3725%09red/hsa:4615%09red
Rap1 signaling pathway	KEGG PATHWAY	hsa04015	3	210	0.04744	0.11118	ACTB|PLCE|CRKL	http://www.genome.jp/kegg-bin/show_pathway?hsa04015/hsa:1399%09red/hsa:51196%09red/hsa:60%09red
PI3K-Akt signaling pathway	KEGG PATHWAY	hsa04151	4	354	0.04755	0.11118	CCND1|LAMB3|MCL1|CDK6	http://www.genome.jp/kegg-bin/show_pathway?hsa04151/hsa:595%09red/hsa:4170%09red/hsa:3914%09red/hsa:1021%09red
Hypertrophic cardiomyopathy (HCM)	KEGG PATHWAY	hsa05410	2	90	0.047681	0.11118	EMD|ACTB	http://www.genome.jp/kegg-bin/show_pathway?hsa05410/hsa:60%09red/hsa:2010%09red
GnRH signaling pathway	KEGG PATHWAY	hsa04912	2	93	0.050491	0.112932	JUN|GNA11	http://www.genome.jp/kegg-bin/show_pathway?hsa04912/hsa:2767%09red/hsa:3725%09red
Fc gamma R-mediated phagocytosis	KEGG PATHWAY	hsa04666	2	94	0.051441	0.112932	WASL|CRKL	http://www.genome.jp/kegg-bin/show_pathway?hsa04666/hsa:1399%09red/hsa:8976%09red

Amoebiasis	KEGG PATHWAY	hsa05146	2	95	0.052397	0.11404	LAMB3|GNA11	http://www.genome.jp/kegg-bin/show_pathway?hsa05146/hsa:2767%09red/hsa:3914%09red
Dilated cardiomyopathy (DCM)	KEGG PATHWAY	hsa05414	2	96	0.053359	0.115278	EMD|ACTB	http://www.genome.jp/kegg-bin/show_pathway?hsa05414/hsa:60%09red/hsa:2010%09red
Endocrine resistance	KEGG PATHWAY	hsa01522	2	98	0.055302	0.115278	CCND1|JUN	http://www.genome.jp/kegg-bin/show_pathway?hsa01522/hsa:595%09red/hsa:3725%09red
Choline metabolism in cancer	KEGG PATHWAY	hsa05231	2	99	0.056283	0.117173	JUN|WASL	http://www.genome.jp/kegg-bin/show_pathway?hsa05231/hsa:3725%09red/hsa:8976%09red
Inflammatory mediator regulation of TRP channels	KEGG PATHWAY	hsa04750	2	100	0.057271	0.118924	PP1B|PP1A	http://www.genome.jp/kegg-bin/show_pathway?hsa04750/hsa:5499%09red/hsa:5500%09red
Toll-like receptor signaling pathway	KEGG PATHWAY	hsa04620	2	104	0.061279	0.121814	MYD88|JUN	http://www.genome.jp/kegg-bin/show_pathway?hsa04620/hsa:3725%09red/hsa:4615%09red
Glucagon signaling pathway	KEGG PATHWAY	hsa04922	2	106	0.063318	0.123009	LDHA|LDHB	http://www.genome.jp/kegg-bin/show_pathway?hsa04922/hsa:3939%09red/hsa:3945%09red
Parathyroid hormone synthesis, secretion and action	KEGG PATHWAY	hsa04928	2	106	0.063318	0.123009	JUND|GNA11	http://www.genome.jp/kegg-bin/show_pathway?hsa04928/hsa:3727%09red/hsa:2767%09red
Insulin resistance	KEGG PATHWAY	hsa04931	2	108	0.06538	0.123697	PP1B|PP1A	http://www.genome.jp/kegg-bin/show_pathway?hsa04931/hsa:5499%09red/hsa:5500%09red
HIF-1 signaling pathway	KEGG PATHWAY	hsa04066	2	109	0.066419	0.124146	LDHA|LDHB	http://www.genome.jp/kegg-bin/show_pathway?hsa04066/hsa:3939%09red/hsa:3945%09red
Endocytosis	KEGG PATHWAY	hsa04144	3	244	0.067691	0.126232	WIPF2|DRG1|WASL	http://www.genome.jp/kegg-bin/show_pathway?hsa04144/hsa:51534%09red/hsa:147179%09red/hsa:8976%09red
2-Oxocarboxylic acid metabolism	KEGG PATHWAY	hsa01210	1	18	0.069486	0.12682	IDH3B	http://www.genome.jp/kegg-bin/show_pathway?hsa01210/hsa:3420%09red
Toxoplasmosis	KEGG PATHWAY	hsa05145	2	113	0.070632	0.128192	LAMB3|MYD88	http://www.genome.jp/kegg-bin/show_pathway?hsa05145/hsa:3914%09red/hsa:4615%09red
Steroid biosynthesis	KEGG PATHWAY	hsa00100	1	19	0.073007	0.129051	DHCR7	http://www.genome.jp/kegg-bin/show_pathway?hsa00100/hsa:1717%09red
Carbon metabolism	KEGG PATHWAY	hsa01200	2	117	0.074929	0.132304	IDH3B|THIL	http://www.genome.jp/kegg-bin/show_pathway?hsa01200/hsa:3420%09red/hsa:38%09red
Neurotrophin signaling pathway	KEGG PATHWAY	hsa04722	2	119	0.077108	0.133687	JUN|CRKL	http://www.genome.jp/kegg-bin/show_pathway?hsa04722/hsa:1399%09red/hsa:3725%09red
AMPK signaling pathway	KEGG PATHWAY	hsa04152	2	120	0.078205	0.135301	CCND1|ELAV1	http://www.genome.jp/kegg-bin/show_pathway?hsa04152/hsa:595%09red/hsa:1994%09red
Terpenoid backbone biosynthesis	KEGG PATHWAY	hsa00900	1	22	0.083492	0.139267	THIL	http://www.genome.jp/kegg-bin/show_pathway?hsa00900/hsa:38%09red
Oocyte meiosis	KEGG PATHWAY	hsa04114	2	128	0.087155	0.143758	PP1B|PP1A	http://www.genome.jp/kegg-bin/show_pathway?hsa04114/hsa:5499%09red/hsa:5500%09red
Dopaminergic synapse	KEGG PATHWAY	hsa04728	2	131	0.090588	0.148069	PP1B|PP1A	http://www.genome.jp/kegg-bin/show_pathway?hsa04728/hsa:5499%09red/hsa:5500%09red
Apelin signaling pathway	KEGG PATHWAY	hsa04371	2	137	0.097569	0.155575	CCND1|CTGF	http://www.genome.jp/kegg-bin/show_pathway?hsa04371/hsa:595%09red/hsa:1490%09red
Fluid shear stress and atherosclerosis	KEGG PATHWAY	hsa05418	2	139	0.099929	0.159019	ACTB|JUN	http://www.genome.jp/kegg-bin/show_pathway?hsa05418/hsa:3725%09red/hsa:60%09red
Biosynthesis of unsaturated fatty acids	KEGG PATHWAY	hsa01040	1	27	0.100705	0.159019	BACH	http://www.genome.jp/kegg-bin/show_pathway?hsa01040/hsa:11332%09red
Fatty acid elongation	KEGG PATHWAY	hsa00062	1	27	0.100705	0.159019	BACH	http://www.genome.jp/kegg-bin/show_pathway?hsa00062/hsa:11332%09red
Butanoate metabolism	KEGG PATHWAY	hsa00650	1	28	0.104109	0.161573	THIL	http://www.genome.jp/kegg-bin/show_pathway?hsa00650/hsa:38%09red
Citrate cycle (TCA cycle)	KEGG PATHWAY	hsa00020	1	30	0.110879	0.16683	IDH3B	http://www.genome.jp/kegg-bin/show_pathway?hsa00020/hsa:3420%09red

Glyoxylate and dicarboxylate metabolism	KEGG PATHWAY	hsa00630	1	30	0.110879	0.16683	THIL	http://www.genome.jp/kegg-bin/show_pathway?hsa00630/hsa:38%09red
Adrenergic signaling in cardiomyocytes	KEGG PATHWAY	hsa04261	2	149	0.111961	0.168149	PP1B|PP1A	http://www.genome.jp/kegg-bin/show_pathway?hsa04261/hsa:5499%09red/hsa:5500%09red
RNA polymerase	KEGG PATHWAY	hsa03020	1	31	0.114244	0.168928	RPB3	http://www.genome.jp/kegg-bin/show_pathway?hsa03020/hsa:5432%09red
Hepatitis C	KEGG PATHWAY	hsa05160	2	155	0.119353	0.173928	CCND1|CDK6	http://www.genome.jp/kegg-bin/show_pathway?hsa05160/hsa:595%09red/hsa:1021%09red
Base excision repair	KEGG PATHWAY	hsa03410	1	33	0.120938	0.173928	PCNA	http://www.genome.jp/kegg-bin/show_pathway?hsa03410/hsa:5111%09red
Jak-STAT signaling pathway	KEGG PATHWAY	hsa04630	2	162	0.128126	0.180284	CCND1|MCL1	http://www.genome.jp/kegg-bin/show_pathway?hsa04630/hsa:595%09red/hsa:4170%09red
Human papillomavirus infection	KEGG PATHWAY	hsa05165	3	330	0.132643	0.185358	CCND1|LAMB3|CDK6	http://www.genome.jp/kegg-bin/show_pathway?hsa05165/hsa:595%09red/hsa:3914%09red/hsa:1021%09red
Primary immunodeficiency	KEGG PATHWAY	hsa05340	1	37	0.134174	0.186061	ARP2	http://www.genome.jp/kegg-bin/show_pathway?hsa05340/hsa:57379%09red
African trypanosomiasis	KEGG PATHWAY	hsa05143	1	37	0.134174	0.186061	MYD88	http://www.genome.jp/kegg-bin/show_pathway?hsa05143/hsa:4615%09red
Thyroid cancer	KEGG PATHWAY	hsa05216	1	37	0.134174	0.186061	CCND1	http://www.genome.jp/kegg-bin/show_pathway?hsa05216/hsa:595%09red
Bladder cancer	KEGG PATHWAY	hsa05219	1	41	0.147213	0.19922	CCND1	http://www.genome.jp/kegg-bin/show_pathway?hsa05219/hsa:595%09red
NOD-like receptor signaling pathway	KEGG PATHWAY	hsa04621	2	178	0.148705	0.201072	MYD88|JUN	http://www.genome.jp/kegg-bin/show_pathway?hsa04621/hsa:3725%09red/hsa:4615%09red
Tryptophan metabolism	KEGG PATHWAY	hsa00380	1	42	0.150442	0.201745	THIL	http://www.genome.jp/kegg-bin/show_pathway?hsa00380/hsa:38%09red
Alcoholism	KEGG PATHWAY	hsa05034	2	180	0.151323	0.20276	PP1B|PP1A	http://www.genome.jp/kegg-bin/show_pathway?hsa05034/hsa:5499%09red/hsa:5500%09red
Fatty acid degradation	KEGG PATHWAY	hsa00071	1	44	0.156863	0.207452	THIL	http://www.genome.jp/kegg-bin/show_pathway?hsa00071/hsa:38%09red
Proteasome	KEGG PATHWAY	hsa03050	1	45	0.160056	0.210308	PSMD7	http://www.genome.jp/kegg-bin/show_pathway?hsa03050/hsa:5713%09red
Chemokine signaling pathway	KEGG PATHWAY	hsa04062	2	190	0.164546	0.214648	WASL|CRKL	http://www.genome.jp/kegg-bin/show_pathway?hsa04062/hsa:1399%09red/hsa:8976%09red
Hedgehog signaling	KEGG PATHWAY	hsa04340	1	47	0.166405	0.215863	CCND1	http://www.genome.jp/kegg-bin/show_pathway?hsa04340/hsa:595%09red
Calcium signaling pathway	KEGG PATHWAY	hsa04020	2	193	0.168553	0.218474	PLCE|GNA11	http://www.genome.jp/kegg-bin/show_pathway?hsa04020/hsa:51196%09red/hsa:2767%09red
Valine, leucine and isoleucine degradation	KEGG PATHWAY	hsa00280	1	48	0.169562	0.218738	THIL	http://www.genome.jp/kegg-bin/show_pathway?hsa00280/hsa:38%09red
Cocaine addiction	KEGG PATHWAY	hsa05030	1	49	0.172707	0.221392	JUN	http://www.genome.jp/kegg-bin/show_pathway?hsa05030/hsa:3725%09red
Intestinal immune network for IgA production	KEGG PATHWAY	hsa04672	1	49	0.172707	0.221392	ARP2	http://www.genome.jp/kegg-bin/show_pathway?hsa04672/hsa:57379%09red
Malaria	KEGG PATHWAY	hsa05144	1	49	0.172707	0.221392	MYD88	http://www.genome.jp/kegg-bin/show_pathway?hsa05144/hsa:4615%09red
Vibrio cholerae infection	KEGG PATHWAY	hsa05110	1	50	0.17584	0.223997	ACTB	http://www.genome.jp/kegg-bin/show_pathway?hsa05110/hsa:60%09red
Arginine and proline metabolism	KEGG PATHWAY	hsa00330	1	50	0.17584	0.223997	P5CR2	http://www.genome.jp/kegg-bin/show_pathway?hsa00330/hsa:29920%09red
Amyotrophic lateral sclerosis (ALS)	KEGG PATHWAY	hsa05014	1	51	0.178962	0.227085	TOM40	http://www.genome.jp/kegg-bin/show_pathway?hsa05014/hsa:10452%09red
Endometrial cancer	KEGG PATHWAY	hsa05213	1	58	0.200485	0.245037	CCND1	http://www.genome.jp/kegg-bin/show_pathway?hsa05213/hsa:595%09red
Lysine degradation	KEGG PATHWAY	hsa00310	1	59	0.203513	0.247623	THIL	http://www.genome.jp/kegg-bin/show_pathway?hsa00310/hsa:38%09red
Steroid hormone biosynthesis	KEGG PATHWAY	hsa00140	1	60	0.206531	0.249061	RING2	http://www.genome.jp/kegg-bin/show_pathway?hsa00140/hsa:7923%09red
Long-term depression	KEGG PATHWAY	hsa04730	1	60	0.206531	0.249061	GNA11	http://www.genome.jp/kegg-bin/show_pathway?hsa04730/hsa:2767%09red
Cortisol synthesis and secretion	KEGG PATHWAY	hsa04927	1	65	0.221447	0.261433	GNA11	http://www.genome.jp/kegg-bin/show_pathway?hsa04927/hsa:2767%09red
Mitophagy - animal	KEGG PATHWAY	hsa04137	1	65	0.221447	0.261433	JUN	http://www.genome.jp/kegg-bin/show_pathway?hsa04137/hsa:3725%09red
Inflammatory bowel disease (IBD)	KEGG PATHWAY	hsa05321	1	65	0.221447	0.261433	JUN	http://www.genome.jp/kegg-bin/show_pathway?hsa05321/hsa:3725%09red

Acute myeloid leukemia	KEGG PATHWAY	hsa05221	1	66	0.224397	0.263387	CCND1	http://www.genome.jp/kegg-bin/show_pathway?hsa05221/hsa:595%09red
Prolactin signaling pathway	KEGG PATHWAY	hsa04917	1	70	0.236085	0.273168	CCND1	http://www.genome.jp/kegg-bin/show_pathway?hsa04917/hsa:595%09red
Epithelial cell signaling in Helicobacter pylori infection	KEGG PATHWAY	hsa05120	1	70	0.236085	0.273168	JUN	http://www.genome.jp/kegg-bin/show_pathway?hsa05120/hsa:3725%09red
Gastric acid secretion	KEGG PATHWAY	hsa04971	1	75	0.25045	0.287338	ACTB	http://www.genome.jp/kegg-bin/show_pathway?hsa04971/hsa:60%09red
B cell receptor signaling pathway	KEGG PATHWAY	hsa04662	1	82	0.270111	0.3058	JUN	http://www.genome.jp/kegg-bin/show_pathway?hsa04662/hsa:3725%09red
Taste transduction	KEGG PATHWAY	hsa04742	1	83	0.272877	0.308075	HBA	http://www.genome.jp/kegg-bin/show_pathway?hsa04742/hsa:6326%09red
ECM-receptor interaction	KEGG PATHWAY	hsa04512	1	86	0.281115	0.315189	LAMB3	http://www.genome.jp/kegg-bin/show_pathway?hsa04512/hsa:3914%09red
Insulin secretion	KEGG PATHWAY	hsa04911	1	86	0.281115	0.315189	GNA11	http://www.genome.jp/kegg-bin/show_pathway?hsa04911/hsa:2767%09red
Gap junction	KEGG PATHWAY	hsa04540	1	88	0.286555	0.319967	GNA11	http://www.genome.jp/kegg-bin/show_pathway?hsa04540/hsa:2767%09red
Protein digestion and absorption	KEGG PATHWAY	hsa04974	1	90	0.291954	0.324659	TRY1	http://www.genome.jp/kegg-bin/show_pathway?hsa04974/hsa:5644%09red
Rheumatoid arthritis	KEGG PATHWAY	hsa05323	1	91	0.294638	0.326974	JUN	http://www.genome.jp/kegg-bin/show_pathway?hsa05323/hsa:3725%09red
Th1 and Th2 cell differentiation	KEGG PATHWAY	hsa04658	1	92	0.297312	0.328821	JUN	http://www.genome.jp/kegg-bin/show_pathway?hsa04658/hsa:3725%09red
TGF-beta signaling pathway	KEGG PATHWAY	hsa04350	1	94	0.302631	0.333119	BRX1	http://www.genome.jp/kegg-bin/show_pathway?hsa04350/hsa:5308%09red
Prostate cancer	KEGG PATHWAY	hsa05215	1	97	0.310533	0.339978	CCND1	http://www.genome.jp/kegg-bin/show_pathway?hsa05215/hsa:595%09red
Pancreatic secretion	KEGG PATHWAY	hsa04972	1	98	0.313147	0.34215	TRY1	http://www.genome.jp/kegg-bin/show_pathway?hsa04972/hsa:5644%09red
Aldosterone synthesis and secretion	KEGG PATHWAY	hsa04925	1	98	0.313147	0.34215	GNA11	http://www.genome.jp/kegg-bin/show_pathway?hsa04925/hsa:2767%09red
Phosphatidylinositol signaling system	KEGG PATHWAY	hsa04070	1	99	0.315752	0.344303	PLCE	http://www.genome.jp/kegg-bin/show_pathway?hsa04070/hsa:51196%09red
NF-kappa B signaling pathway	KEGG PATHWAY	hsa04064	1	100	0.318347	0.346436	MYD88	http://www.genome.jp/kegg-bin/show_pathway?hsa04064/hsa:4615%09red
T cell receptor signaling pathway	KEGG PATHWAY	hsa04660	1	103	0.326072	0.353191	JUN	http://www.genome.jp/kegg-bin/show_pathway?hsa04660/hsa:3725%09red
C-type lectin receptor signaling pathway	KEGG PATHWAY	hsa04625	1	104	0.328628	0.355486	JUN	http://www.genome.jp/kegg-bin/show_pathway?hsa04625/hsa:3725%09red
Th17 cell differentiation	KEGG PATHWAY	hsa04659	1	107	0.336238	0.362754	JUN	http://www.genome.jp/kegg-bin/show_pathway?hsa04659/hsa:3725%09red
Cholinergic synapse	KEGG PATHWAY	hsa04725	1	112	0.348731	0.373755	GNA11	http://www.genome.jp/kegg-bin/show_pathway?hsa04725/hsa:2767%09red
TNF signaling pathway	KEGG PATHWAY	hsa04668	1	112	0.348731	0.373755	JUN	http://www.genome.jp/kegg-bin/show_pathway?hsa04668/hsa:3725%09red
Leukocyte transendothelial migration	KEGG PATHWAY	hsa04670	1	112	0.348731	0.373755	ACTB	http://www.genome.jp/kegg-bin/show_pathway?hsa04670/hsa:60%09red
Lysosome	KEGG PATHWAY	hsa04142	1	123	0.3754	0.397367	CATE	http://www.genome.jp/kegg-bin/show_pathway?hsa04142/hsa:1510%09red
Relaxin signaling pathway	KEGG PATHWAY	hsa04926	1	130	0.391803	0.413118	JUN	http://www.genome.jp/kegg-bin/show_pathway?hsa04926/hsa:3725%09red
FoxO signaling pathway	KEGG PATHWAY	hsa04068	1	132	0.39641	0.417166	CCND1	http://www.genome.jp/kegg-bin/show_pathway?hsa04068/hsa:595%09red
Ubiquitin mediated proteolysis	KEGG PATHWAY	hsa04120	1	137	0.407778	0.427196	CHIP	http://www.genome.jp/kegg-bin/show_pathway?hsa04120/hsa:10273%09red
Estrogen signaling pathway	KEGG PATHWAY	hsa04915	1	138	0.410026	0.428723	JUN	http://www.genome.jp/kegg-bin/show_pathway?hsa04915/hsa:3725%09red
Signaling pathways regulating pluripotency of stem cells	KEGG PATHWAY	hsa04550	1	140	0.414496	0.43201	BMI1	http://www.genome.jp/kegg-bin/show_pathway?hsa04550/hsa:100532731%09red
Non-alcoholic fatty liver disease (NAFLD)	KEGG PATHWAY	hsa04932	1	149	0.4342	0.450241	JUN	http://www.genome.jp/kegg-bin/show_pathway?hsa04932/hsa:3725%09red
Gastric cancer	KEGG PATHWAY	hsa05226	1	149	0.4342	0.450241	CCND1	http://www.genome.jp/kegg-bin/show_pathway?hsa05226/hsa:595%09red
Phagosome	KEGG PATHWAY	hsa04145	1	152	0.440621	0.456317	ACTB	http://www.genome.jp/kegg-bin/show_pathway?hsa04145/hsa:60%09red
mTOR signaling pathway	KEGG PATHWAY	hsa04150	1	153	0.442745	0.458225	SEC13	http://www.genome.jp/kegg-bin/show_pathway?hsa04150/hsa:6396%09red
Tuberculosis	KEGG PATHWAY	hsa05152	1	179	0.495244	0.509967	MYD88	http://www.genome.jp/kegg-bin/show_pathway?hsa05152/hsa:4615%09red
Huntington disease	KEGG PATHWAY	hsa05016	1	193	0.521446	0.534918	RPB3	http://www.genome.jp/kegg-bin/show_pathway?hsa05016/hsa:5432%09red
Thermogenesis	KEGG PATHWAY	hsa04714	1	231	0.585952	0.596579	ACTB	http://www.genome.jp/kegg-bin/show_pathway?hsa04714/hsa:60%09red

Ras signaling pathway	KEGG PATHWAY	hsa04014	1	232	0.587527	0.597809	PLCE	http://www.genome.jp/kegg-bin/show_pathway?hsa04014/hsa:51196%09red
Cytokine-cytokine receptor interaction	KEGG PATHWAY	hsa04060	1	294	0.674412	0.681106	GDF15	http://www.genome.jp/kegg-bin/show_pathway?hsa04060/hsa:9518%09red
#Term	Database	ID	Input num	Backgroun	P-Value	Corrected	Input	Hyperlink

----

HCT116

Accession	Description	Score	Coverage	# Proteins	# Unique Peptides	# Peptides	# PSMs	Area	# AAs	MW [kDa]	calc. pI						
P24385	G1/S-specific cyclin-D1	3.27	6.44	1	2	2	2	3.378E7	295	33.7	5.02						
	A2	Sequence	# PSMs	# Proteins	# Protein Groups	Protein Group Accessions	Modifications	ΔCn	Area	q-Value	PEP	XCorr	Charge	MH+ [Da]	ΔM [ppm]	RT [min]	# Missed Cleavages
	High	SPNNFLSYYR	1	1	1	P24385		0	45175488.5	0	9.185E-05	3.274598837	2	1260.603807	2.40048805	29.60381964	0
	High	FLSLEPVKK	1	1	1	P24385		0	22389299.4	0.004	0.05219	1.435350895	2	1060.643236	2.898972254	23.14352007	1
Q00534	Cyclin-dependent kinase 6	10.98	13.50	56	3	4	6	1.613E8	326	36.9	6.46						
	A2	Sequence	# PSMs	# Proteins	# Protein Groups	Protein Group Accessions	Modifications	ΔCn	Area	q-Value	PEP	XCorr	Charge	MH+ [Da]	ΔM [ppm]	RT [min]	# Missed Cleavages
	High	LADFGLAR	1	66	3	P50750;Q00534;E5RIU6		0.0000	4.148E8	0	0.003093	2.55	2	862.47942	1.43	26.78	0
	High	LFDVcTVSR	2	1	1	Q00534	C5(Carbamidomethy	0.0000	3.110E7	0	0.0009943	2.26	2	1096.54790	2.09	27.45	0
	High	HLETFEHPNVVR	2	1	1	Q00534		0.0000	2.196E7	0	0.003176	1.99	3	1477.75797	2.22	20.27	0
	High	ILDVIGLPGEEDWPR	1	1	1	Q00534		0.0000	3.804E7	0.001	0.007249	2.13	2	1708.89287	1.35	43.26	0

AGS

Accession	Description	Score	Coverage	# Proteins	# Unique Peptides	# Peptides	# PSMs	Area	# AAs	MW [kDa]	calc. pI						
P24385	G1/S-specific cyclin-D1	5.963283539	14.58	1	3	3	3	24355509	295	33.70690284	5.0209961						
	A2	Sequence	# PSMs	# Proteins	# Protein Groups	Protein Group Accessions	Modifications	ΔCn	Area	q-Value	PEP	XCorr	Charge	MH+ [Da]	ΔM [ppm]	RT [min]	# Missed Cleavages
	High	FISNPPSmVAAGSVVAAVQGLNLR	1	1	1	P24385	M8(Oxidation)	0	29722507.8	0	0.002006	2.881889343	3	2414.291172	2.069157074	42.86086553	0
	High	SPNNFLSYYR	1	1	1	P24385		0	28749022.5	0.001	0.009496	3.081394196	2	1260.603319	2.013149646	31.05147177	0
	High	FLSLEPVKK	1	1	1	P24385		0	14594998	0.001	0.07364	1.402794242	2	1060.642015	1.748065946	24.03278257	1
Q00534	Cyclin-dependent kinase 6	8.12	21.47	60	5	6	6	3.962E7	326	36.9	6.46						
	A2	Sequence	# PSMs	# Proteins	# Protein Groups	Protein Group Accessions	Modifications	ΔCn	Area	q-Value	PEP	XCorr	Charge	MH+ [Da]	ΔM [ppm]	RT [min]	# Missed Cleavages
	High	LADFGLAR	1	66	2	Q00534;P50750		0.0000	6.930E7	0.001	0.02289	2.43	2	862.47911	1.08	28.11	0
	High	ILDVIGLPGEEDWPR	1	1	1	Q00534		0.0000	1.551E7	0.001	0.08025	2.28	2	1708.89324	1.57	44.84	0
	High	DLKPQNILVTSSGQIK	1	1	1	Q00534		0.0000	1.313E7	0.001	0.03255	1.81	3	1740.98911	2.07	30.27	0
	High	GSSDVDQLGK	1	1	1	Q00534		0.0000	6.889E6	0.001	0.0297	1.62	2	1005.48613	1.34	18.72	0
	High	HLETFEHPNVVR	1	1	1	Q00534		0.0000	7.472E6	0.001	0.06258	1.07	2	1477.75823	2.39	20.88	0
	High	LFDVcTVSR	1	1	1	Q00534	C5(Carbamidomethy	0.0000	3.404E7	0.003	0.1439	1.78	2	1096.54766	1.87	28.83	0
